# Abstract Supplement Abstracts from AIDS 2024, the 25th International AIDS Conference, 22 – 26 July, Munich, Germany & Virtual

**DOI:** 10.1002/jia2.26279

**Published:** 2024-07-23

## ORAL ABSTRACT

### OAA0202 Consecutive analytical treatment interruption enhances CD8 T‐cell functionality during virological control

G. Duette ^1,2^, S. Cronin^1,2^, K. Fisher^1^, S. G. Deeks^3^, A. D. Kelleher^4^, S. Palmer^1,2^

^1^The Westmead Institute for Medical Research, Centre for Virus Research, Westmead, Australia, ^2^The University of Sydney, Faculty of Medicine and Health, Sydney, Australia, ^3^University of California, San Francisco, Department of Medicine, San Francisco, United States, ^4^The Kirby Institute, University of New South Wales, Sydney, Australia

**Background: **The PULSE clinical trial is one of the first studies where 68 people living with HIV underwent three consecutive analytical treatment interruptions (ATIs). During this trial, we observed that 10% of the participants transiently controlled HIV rebound after the second and/or third ATI. In this study, we investigated whether consecutive ATIs result in an improved antiviral CD8 T‐cell immune response during delayed viral rebound.

**Methods: **We obtained peripheral blood mononuclear cells from four participants enrolled in the PULSE clinical trial: two non‐controllers (NCs) who experienced rapid viral rebound during all ATIs and two transient controllers (TCs) who exhibited virological control for up to 6 months during the second and/or third ATI. To evaluate CD8 T‐cell functionality during the consecutive ATIs, CD8 T cells were stimulated with Gag/Pol/Nef peptides to assess cytokine production (IFN‐γ and TNF‐α) and stained with CellTrace Far Red to quantify proliferation by flow cytometry. To compare the CD8 T‐cell cytolytic capacity across the ATIs, participant‐derived CD4 T cells were infected with HIV‐NL4‐3 and cocultured with autologous CD8 T cells from each ATI timepoint. At day 3 of coculture, the levels of p24 were measured by ELISA.

**Results: **For TC participants, we observed an increase in both CD8 T‐cell proliferation and cytokine production (3‐ to 34‐fold) during the transient viral control timepoints. In contrast, the capacity for CD8 T cells to proliferate and produce cytokines did not increase across the ATI timepoints for the NCs. Moreover, TC‐derived CD8 T cells from the third ATI, where viral rebound is delayed, eliminated HIV‐infected CD4 T cells more efficiently, as reflected by a two‐ to five‐fold decrease in p24, when compared to CD8 T cells from earlier ATI timepoints. In contrast, within NCs, we did not observe a consistent enhancement of CD8 T‐cell cytolytic response across the ATI timepoints.

**Conclusions: **Our findings indicate that in some participants from the PULSE study, repeated exposure to viral antigens during consecutive ATIs results in a potential “vaccinal effect” improving CD8 T‐cell proliferation, cytokine production and cytotoxicity. Importantly, these results provide insight into the immunological mechanisms contributing to viral control indicating that enhanced host immunity is pivotal for achieving viral remission after an ATI.

### OAA0203 HIV reservoir characteristics in spontaneous viral controllers and individuals treated during both acute and chronic HIV‐1 infection in South Africa

T. J. B. Chikowore ^1,2^, N. Reddy^1,3^, G. Q. Lee^4^, K. Dong^5,6^, K. Reddy^1^, T. Ndung'u^1,2,3,5,6^

^1^Africa Health Research Institute, Durban, South Africa, ^2^University College London, London, United Kingdom, ^3^University of KwaZulu Natal, Durban, South Africa, ^4^Weill Cornell Medicine, New York, United States, ^5^University of KwaZulu Natal, HIV Pathogenesis Programme, Doris Duke Medical Research Institute, Durban, South Africa, ^6^MIT and Harvard University, Ragon Institute of MGH, Cambridge, United States

**Background: **The HIV reservoir is the main barrier to cure efforts. Individuals who control the virus without antiretroviral therapy (ART) represent a model of functional cure, but few studies have characterized reservoir dynamics and mechanisms of persistence in these individuals. In this study, we measured and characterized the reservoir size and genetic landscape before and after ART in spontaneous controllers versus viraemic individuals who initiated ART in acute or chronic phases of infection.

**Methods: **Participants were from a natural controller and the FRESH acute infection cohorts in Durban, South Africa. At enrolment, all participants were ART‐naïve and included 16 elite controllers (EC, viral load [VL] <100 copies/ml); 9 viraemic controllers (VC, viral load [VL] <2000 copies/ml); 12 with viraemic chronic HIV infection (CHI, VL >2000 copies/ml) and 35 with acute HIV infection (AHI, median of 1 [1−3] day post detection). A subset from each group initiated ART during follow up and were assessed prior to treatment; 1, 3 and 5 years after treatment. HIV DNA was measured in peripheral blood mononuclear cells (PBMCs) by droplet digital polymerase chain reaction (PCR). Proviral genome characteristics were analysed by near full‐length individual proviral sequencing.

**Results: **In untreated infection, there was no significant difference in total HIV‐1 DNA between the study groups. Notably, elite controllers showed a lower proportion (9.68%) of intact genomes in comparison to both individuals with untreated AHI (62.04%) and CHI (31.18%). ECs and individuals treated in AHI showed a significant reduction in proviral load (*p* = 0.035 and <0.0001, respectively) in contrast to VCs and CHI‐treated participants who showed no significant change after 5 years of ART. Additionally, after 1 year of treatment, no intact genomes were detectable in AHI‐treated individuals, whereas in ECs and CHI‐treated individuals, 14.06% and 3.85% of genomes were intact.

**Conclusions: **In untreated HIV‐1 subtype C infection, proviral load is similar between spontaneous controllers and individuals with AHI or CHI. ECs and individuals initiating treatment in AHI showed a significant reduction in reservoir size after treatment. Our results suggest that reservoir quality and not size may, in part, explain ART‐free viral control and that the timing of ART initiation might play a key role in a potential post‐treatment functional cure.

### OAA0204 Characterization of the HIV‐1 reservoir in early treated individuals from the Zurich Primary HIV Infection Study with low‐level viraemia

J. Tschumi ^1,2^, L. Jörimann^1,2^, K. Neumann^1,2^, M. Zeeb^1,2^, P. Frischknecht^1^, D. Braun^1,2^, R. D. Kouyos^1,2^, K. J. Metzner^1,2^, H. F. Günthard^1,2^, Swiss HIV Cohort Study

^1^University Hospital Zurich, Department of Infectious Diseases and Hospital Epidemiology, Zurich, Switzerland, ^2^University of Zurich, Institute of Medical Virology, Zurich, Switzerland

**Background: **In most people living with HIV (PWH), antiretroviral therapy (ART) reduces viral loads to undetectable levels. However, in some individuals, viraemia remains detectable despite very early initiation of ART during primary HIV infection, adherence to drug regimens and absence of drug resistance mutations. The causes of such residual viraemia remain unclear. We conducted a longitudinal analysis of the HIV‐1 reservoir in early treated individuals with low‐level viraemia to investigate potential HIV‐1 evolution.

**Methods: **Near full‐length HIV‐1 proviral next‐generation sequencing (NGS) in bulk was applied to three longitudinal PBMC samples from well‐characterized participants enrolled in the Zurich Primary HIV Infection study with low‐level viraemia between 50 and 500 copies/ml plasma (*n* = 7) for at least 4 years and matched controls with viral loads <50 copies/ml plasma (*n* = 14). All individuals initiated ART during primary HIV‐1 infection and reported highest levels of adherence. We included a baseline sample prior to, and a sample 2 and 4 years after ART initiation. After removing hypermutated reads, genetic distances and diversities were calculated and drug resistance mutations were assessed. Total, intact and unintegrated HIV‐1 DNA and HIV‐1 transcript were quantified using digital PCR.

**Results: **We observed similar frequencies of intact HIV‐1 proviruses in individuals with and without low‐level viraemia. While the numbers of unspliced HIV‐1 RNA in PBMCs of individuals with low‐level viraemia was higher than of those without, numbers of HIV‐1 multiply spliced RNA and 2‐LTR circles showed no significant differences between individuals with and without low‐level viraemia. After removing hypermutated reads, no increase in genetic distance or diversity of proviral sequences over time was detected in individuals with low‐level viraemia. While minor variants of proviral drug resistance mutations with frequencies <15% appeared and vanished over time, major drug resistance mutations with frequencies >15% were not detected in proviral DNA in any individual with low‐level viraemia.

**Conclusions: **Our results show no evidence of evolution of the HIV‐1 reservoir or drug resistance development, despite years of low‐level viraemia. Thus, treatment intensification of individuals with low‐level viraemia may not be needed in PWH who initiated ART with a high resistance barrier during primary HIV‐1 infection, given optimal adherence and frequent monitoring.

### OAA0205 Experimental macaque model of post‐treatment SIV control presents distinct features of viral reservoirs in blood and tissues, the pVISCONTI study

C. Charre ^1,2^, A. Melard^1^, A. Chaillon^3^, E. Gardiennet^1^, D. Desjardins^4^, C. Passaes^5^, V. Monceaux^5^, M. Muller‐Trutwin^5^, C. Rouzioux^6^, R. Le Grand^4^, A. Saez‐Cirion^5^, V. Avettand‐Fenoël^1,2^

^1^Université Paris Cité; INSERM, U1016; CNRS, UMR8104, Paris, France, ^2^APHP Hôpital Cochin, Virology Department, Paris, France, ^3^University of California, Department of Medicine/Division of Infectious Diseases, San Diego, United States, ^4^Université Paris‐Saclay, CEA, INSERM, UMR1184, Immunology of Viral, Auto‐Immune, Hematological and Bacterial Diseases (IMVA‐HB/IDMIT Department), Fontenay‐aux‐Roses/Le Kremlin‐Bicêtre, France, ^5^Institut Pasteur, HIV Inflammation et Persistance Unit, Paris, France, ^6^Université Paris Cité/APHP Hôpital Necker—Enfants Malades, Paris, France

**Background: **The pVISCONTI study confirmed in a non‐human primate model of simian immunodeficiency virus (SIV) infection that post‐treatment control is favoured by antiretroviral treatment (ART) initiation at day (D)28 (ET) versus 6 months post‐infection (LT). However, intrinsic viral features of reservoirs in such control remain elusive. We performed a large analysis of tissue and blood reservoirs in the cynomolgus macaques (CyMs) of this study.

**Methods: **SIVmac251‐infected CyMs were classified as post‐treatment controllers (PTCs, *n* = 11) or non‐PTCs (*n* = 11) depending on their capacity to control viraemia <400 copies/ml for >6 months after analytical treatment interruption (ATI) after 24 months of ART. Necropsy was performed 6−12 months after ATI. From antemortem serial blood and lymph nodes (LN) specimens and postmortem tissues from 12 anatomical sites, we quantified total SIV‐DNA, cell‐associated SIV‐RNA (caRNA) and evaluated reservoir integrity by Intact Provirus DNA Assay. Near‐full‐length SIV genome nanopore sequencing was performed in five PTCs (3ET, 2LT) and five non‐PTCs (2ET, 3LT).

**Results: **SIV‐DNA was lower in PTCs versus non‐PTCs from D14 post‐ATI in circulating CD4^+^ T cells (*p* = 0.009). caRNA in PBMCs was lower in PTCs than non‐PTCs from D28 post‐ATI (*p* = 0.004). No difference was noticed before ATI. At necropsy, SIV‐DNA and caRNA were lower in PTCs than non‐PTCs in thymus, LN, spleen, ileum, colon, rectum and PBMCs (*p*<0.05). Intact proviruses were less abundant in PTCs versus non‐PTCs in LN before ATI (*p*<0.001) and in LN, spleen and colon at necropsy (*p*<0.015); investigation in other tissues is ongoing. Regardless time of treatment initiation, post‐mortem tissue proviruses showed: (i) individual‐related evolution in non‐PTCs in contrast to sequences intermingled between PTCs; (ii) lower pairwise genetic distance to the inoculum in PTCs versus non‐PTCs (*p* = 0.008). In non‐PTCs, but not in PTCs, tissue proviruses at necropsy were genetically closer to circulating viruses at the time of ART initiation than to those at primary infection (*p* = 0.014).

**Conclusions: **PTC status was associated with lower levels of intact proviruses in LN before ATI and with limited reservoir size, transcriptional activity and viral evolution. This study might help to better understand which factors in addition to early treatment initiation are important for post‐treatment control.

### OAA0602 Antibody signatures associated with HIV transmission during breastfeeding

N. Nziza ^1^, W. Jung^1^, F. Li^2^, J. Hsiao^1,3^, T. Chen^1^, C. Atyeo^1^, R. McNamara^1^, G. Alter^1^, L. Kuhn^4^, N. Tobin^2^, G. Aldrovandi^2^, B. Juelg^1^

^1^Ragon Institute of MGH, MIT and Harvard, Cambridge, United States, ^2^David Geffen School of Medicine at University of California, Los Angeles, United States, ^3^MIT, Cambridge, United States, ^4^Columbia University Irving Medical Center, New York, United States

**Background: **In the absence of antiretroviral therapy, up to 15% of mothers living with HIV transmit the virus to their children during pregnancy, delivery and breastfeeding. Antibodies have largely been studied for their impact on HIV transmission to children *in utero*, but their presence in the milk and the mechanisms by which they might affect HIV transmission to the newborn during breastfeeding is less well understood.

**Methods: **In this study, we comprehensively profiled humoral immune responses in breastmilk of cohorts from the Zambia Exclusive Breastfeeding Study. All 34 mother/child dyads included in our analysis (17 without and 17 with HIV breastfeeding‐associated transmission) were ART naïve. We applied our System's Serology platform, an approach that interrogates subclass/isotype/Fc‐receptor binding across different antigen‐specificities and functional assays to measure antibody effector functions in parallel; all linked to systems biology/machine learning algorithms.

**Results: **We demonstrated that antibody responses in the breastmilk of transmitting mothers are higher after HIV transmission compared to non‐transmitting women, despite similar viral loads in plasma and breastmilk as well as CD4 T‐cell counts between the two groups. Particularly, higher IgG levels and FcgR2A binding against gp41 and gp140 were detected in the transmitting group (*p* < 0.05), in addition to higher FcgR2B and ADCD against gp41 (*p* < 0.05). We also observed an increase in humoral response over time in transmitting mothers, while antibody response progressively decreased in non‐transmitting mothers.

**Conclusions: **This study suggests that a strong immune activation in the milk of HIV‐infected mothers is seen with breastfeeding‐associated HIV transmission and highlights particular antibody Fc‐effector profiles that might be involved in this transmission. These data contribute to a more comprehensive understanding of Fc‐mediated antibody response in the breastmilk, which is crucial for advancing therapeutic strategies aimed at eliminating this route of mother‐to‐child transmission.

### OAA0603 Increased cardiovascular risk in perinatally HIV‐acquired adolescents may linked to proinflammatory NK cells

N. Liyanage ^1^, M. A. Alles^2^, M. Gunasena^2^, V. Musiime^3^, C. Kityo^4^, B. Tamilselvan^4^, B. Richardson^4^, W. Ching‐Wen L^4^, S. Sun^5^, C. Cameron^4^, M. Cameron^4^, S. Dirajlal‐Fargo^5^, N. Funderburg^1^

^1^The Ohio State University, College of Medicine, Columbus, United States, ^2^The Ohio State University, Columbus, United States, ^3^Joint Clinical Research Center, Kampala, Uganda, ^4^Case Western Reserve University School of Medicine, Cleveland, United States, ^5^Northwestern University Feinberg School of Medicine, Chicago, United States

**Background: **Our initial findings and literature suggest a potential reprogramming of innate immune cells in adolescents acquiring HIV perinatally and undergoing antiretroviral therapy. This reprogramming may accelerate ageing, increasing the risk of future complications, particularly cardiovascular disease (CVD). Natural killer (NK) cells, with diverse functions, play a crucial role in HIV pathogenesis and are implicated in comorbidities like CVD through immune crosstalk. Despite this understanding, the specific involvement of NK cells in HIV‐related cardiovascular risks remains unclear. Here, we studied NK cell subsets and their potential role in cardiovascular risk in adolescents with and without HIV.

**Methods: **In this cross‐sectional study, using high‐dimensional flow cytometry, plasma biomarker profiling and transcriptomics, we compared cardiovascular risk factors and immune signatures in cryopreserved peripheral blood mononuclear cells, as well as cardiovascular biomarkers (carotid intima‐media thickness and pulse wave velocity—PWV) in Ugandan adolescents with perinatally acquired HIV on antiretroviral therapy and virally suppressed (*n* = 18) and age/sex‐matched HIV‐unexposed and uninfected adolescents (*n* = 20). At baseline, the median age was 14 years, and 50% were females.

**Results: **In the PHIV, we found elevated activation, maturation, memory and pro‐inflammatory/migration markers in most NK subsets compared to HIV negative (*p*<0.05). Oxidized low density lipoprotein (LDL) levels were significantly lower in the plasma of PHIVs (*p*<0.05). Further, negative correlations were found between all activated CCR5+NK subsets and plasma oxLDL among PHIVs. This was confirmed by in vitro studies which revealed increased uptake of oxLDL by macrophages in the presence of activated NK cells (*p*<0.05). Bulk‐RNA sequencing data revealed differential expression of genes associated with immune cell migration, cholesterol uptake into tissue and vascular remodelling, and enrichment of pathways associated with NK activation and epigenetic regulation in the PHIV group (*p*<0.05). Interestingly, the dysregulated NK subsets showed significant correlations with carotid intima‐media thickness and PVW.

**Conclusions: **Our data, for the first time, reveal an increase in several activated, mature NK subsets capable of homing to vascular tissue. This correlates with increased plasma oxLDL uptake by macrophages. Dysregulated NK subsets exhibit significant correlations with carotid intima‐media thickness and PVW, suggesting a potential link between NK cells and cardiovascular risk in adolescents with PHIV.

### OAA0604 Comparative analysis of the HIV reservoir localization and cellular function in paediatric and adult tonsillar tissues

F. Shaik Abdool ^1,2^, K. Gopee^1^, S. Mfusi^1^, N. Mthabela^3^, F. Karim^3^, A. L. Sibiya^4^, W. Kuhn^5^, J. Z. Porterfield^1,6^, A. Leslie^1,7^, H. N. Kløverpris^1,8^

^1^Africa Health Research Institute, Basic and Translation Science (Immunology), Durban, South Africa, ^2^University of KwaZulu‐Natal, School of Clinical & Laboratory Medicine, Durban, South Africa, ^3^Africa Health Research Institute, Clinical Core, Durban, South Africa, ^4^King Edward VIII Hospital, University of KwaZulu‐Natal, Department of Otorhinolaryngology, Durban, South Africa, ^5^General Justice Gizenga Mpanza Regional Hospital, University of KwaZulu‐Natal, Department of Ear, Nose and Throat, Durban, South Africa, ^6^University of South Florida, Division of Infectious Disease and International Medicine, Tampa, United States, ^7^University College London, Division of Infection and Immunity, London, United Kingdom, ^8^University of Copenhagen, Department of Immunology and Microbiology, Copenhagen, Denmark

**Background: **Anti‐retroviral treatment in paediatric HIV infection is challenging with low ART adherence throughout childhood and adolescence, particularly in under resourced populations. Therefore, non‐ART strategies are important to achieve HIV remission during paediatric HIV. Viral reservoir seeding occurs rapidly within lymphoid tissues after transmission and remains the major source of viral rebound following ART cessation. However, the size, localization and mechanisms of viral silencing and reactivation within viral reservoirs in lymphoid tissues from children born with HIV remain unknown.

**Methods: **We hypothesize that differences in CD4^+^ T‐follicular helper cell (TFH) frequencies within germinal centres (GCs) in children and adults contribute to variations in the viral reservoir landscape. To study the paediatric HIV lymphoid tissue reservoir, we used flow cytometry, single‐cell RNA sequencing (scRNAseq) and fluorescent microscopy to study the viral reservoirs from resected tonsils from children (<12 years) born with HIV from our larger cohort of adult and paediatric participants within South Africa.

**Results: **We found higher frequencies of TFH (PD‐1^++^/CXCR5^+^) in paediatric participants compared to adults (*p* = 0.004), suggesting elevated levels of HIV susceptible cells in paediatric lymphoid tissues. Immunofluorescent microscopy showed that HIV‐p24 antigens were localized within both GC and extrafollicular localization in viraemic paediatric samples, whereas plasma viral suppressed individuals overall showed lower frequencies of GC HIV localization. scRNAseq (10X Genomics) and HIV sequencing from tonsils of people living with HIV is underway to determine the transcriptional profiles, viral diversity and frequency of intact virus between paediatric and adult participants.

**Conclusions: **This study presents a unique opportunity to investigate the viral reservoir location in paediatric lymphoid tissues that is important to tailor HIV cure strategies for children born with HIV.

### OAA0605 Altered immune response to tetanus paediatric vaccines among HIV‐exposed and negative infants

S. Osawe ^1^, C. Co Chukwu^2^, J. A. Okopi^3^, A. Abimiku^1^

^1^Institute of Human Virology Nigeria, International Research Center of Excellence, Abuja, Nigeria, ^2^National Veterinary Research Institute, Medical Laboratory Science, Vom, Nigeria, ^3^Federal University of Health Sciences, Department of Microbiology, Otukpo, Nigeria

**Background: **Evidence suggests that the developing immune system of HIV‐exposed but uninfected (HEU) infants early in life, differs from their HIV‐unexposed (HU) peers. More so, reports show that HEU infants do not respond optimally to paediatric vaccinations, thereby increasing their vulnerability to infectious diseases and raising the potential for elevated rates of morbidity and mortality. This study documented the impact of HIV exposure on how infants respond to a paediatric vaccination.

**Methods: **We documented the immune responses in HEU and HU infants and their moms by quantifying the total IgG antibodies against Tetanus toxoid (TT), one of the prescribed paediatric vaccines, in a Nigerian birth cohort. Plasma samples collected from each mother‐infant pair at Birth (mother and infant) and Week 15 (infants only) were tested to quantify specific anti‐TT IgG titres.

**Results: **A total of 200 pregnant women were enrolled with their infants, 140 living with HIV and 60 living without HIV. Mean maternal age was similar for both groups (*p* = 0.24) as were the number of pregnancies, number of live births and mode of delivery (*p* = 0.19, *p* = 0.38 and *p* = 0.77). A total of 205 infants were enrolled at birth with 144 HEU infants and 61 HU infants with significant differences in weight (*p* = 0.00), height (*p* = 0.03) and head circumference (*p* = 0.016). There was also a 12% decrease in the median transfer of anti‐TT IgG antibodies in mothers living with HIV (*p* = 0.02). HU infants maintained significantly higher anti‐TT IgG titres at Week 15 compared to HEU infants at birth (*p* = 0.018) and Week 15 (*p* = 0.001).

**Conclusions: **Our data show that HEU infants present with altered immune responses compared to HU infants. Exposure to HIV in these infants may alter the responses to infectious diseases and how well they respond to childhood immunization highlighting the need for focused strategies to improve responses to vaccine‐preventable diseases and the possibility of including additional booster shots.

### OAA1308 TCR repertoire diversity allows for expansion of HIV‐specific CD8 T‐cells following anti‐PD1 in people with HIV and cancer on ART

C. Gubser ^1^, L. Rajdev^2^, C. Chiu^3^, S. Li^4^, C. Tumpach^5^, R.D. Pascoe^5^, C. Durand^6^, J.J. Chang^5^, A. Solomon^5^, R. Cao^5^, L. Martelotto^7^, J. Bethony^8^, D. Utzschneider^5^, A. Kallies^5^, M. Davenport^9^, J.L. Anderson^4^, T.A. Rasmussen^10^, J. Schroeder^4^, S.R. Lewin^5^, this Project Was Partially Funded with an AMC Grant: Number UM1 CA121947, PI; Dr. Joseph Sparano; and the American Foundation for AIDS Research (amfAR) Impact Grant (109226‐58‐RGRL) (SRL and CD) and from the National Institutes of Health Delaney AIDS Research Enterprise to Find a Cure Collaboratory (Grant UM1AI126611‐01) and from the Australian Centre for HIV and Hepatitis Virology Research (CG, TAR, SRL) and the National Health and Medical Research Council (NHMRC) of Australia APP 1149990 (SRL, CG, TAR) and 1135851 (SRL); and from the J and M Wright Foundation (JS, JA)

^1^Peter Doherty Institute, Univsersity of Melbourne, Infectious Diseases, Melbourne, Australia, ^2^Mount Sinai Hospital, New York, United States, ^3^St. Vincent's Institute, Melbourne, Australia, ^4^Peter Doherty Institute, University of Melbourne, Computational Science Initiative, Melbourne, Australia, ^5^Peter Doherty Institute, University of Melbourne, Infectious Diseases, Melbourne, Australia, ^6^John Hopkins University, Baltimore, United States, ^7^University of Adelaide, South Australia, Australia, ^8^AMC, George Washington University, Washington, United States, ^9^Kirby Institute, Sydney, Australia, ^10^Aarhus University Hospital, Aarhus, Denmark

**Background: **In people living with HIV (PWH), immune dysfunction persists with elevated expression of the exhaustion marker programmed death (PD1) despite suppressive antiretroviral therapy (ART). Anti‐PD1 therapy in people with cancer can enhance tumor‐specific T‐cell responses through the proliferative burst of exhausted effector T‐cells (T_EX_). We aimed to determine the effects of anti‐PD1 in vivo on the T‐cell receptor (TCR) repertoire of HIV‐specific CD8 T‐cells.

**Methods: **As part of a prospective longitudinal clinical trial of PWH on ART with cancer (AIDS Malignancy Consortium‐095 Study), participants received anti‐PD1 (nivolumab) every 3 weeks. Blood was collected prior to and following the first, fourth and subsequent infusions. We sorted HIV‐tetramer+ CD8 T‐cells from six participants and performed single cell RNA sequencing and TCR repertoire computational analysis. We used a data‐based frequency cut‐off and considered a clonotype as expanded when a particular TCR sequence constituted > 2% of the overall TCR sequences.

**Results: **Out of 1828 TCR clonotypes, 55 expanded, totalling 913 cells, with 78.2% identified as T_EM_ or T_EX_ cells. All cells with expanded clonotypes showed differentially expressed genes upregulated for effector functions and antigen recognition. Baseline TCR diversity positively correlated with expanded clonotypes after a single anti‐PD1 dose (r=0.57). In 3 participants, high diversity in HIV tetramer+ CD8 T‐cells led to rapid expansion and subsequent contraction with additional doses. In the other 3 participants, lower diversity also resulted in clonotype expansion, but only after multiple anti‐PD1 doses. Pairwise TCR distance analysis demonstrated that expanded clones were biochemically diverse and therefore likely recognise a diverse range of HIV epitopes.

**Conclusions: **PWH on ART and cancer with a diverse HIV‐specific TCR repertoire at baseline exhibit rapid clonotype expansion after a single anti‐PD1 dose, while a less diverse TCR repertoire requires multiple doses of anti‐PD1 for expansion. Whether these expanded clonotypes can control HIV replication once ART is stopped remains to be determined in future clinical trials of anti‐PD1 in PWH on ART.

### OAA1303 The HIV‐1 Tat‐based therapeutic vaccine to intensify or replace antiretroviral therapy: results of extended (8 years) follow up of Phase‐II clinical trials in italy and South Africa

C. Sgadari^1^, A. Tripiciano^1^, O. Picconi^1^, A. Cafaro^1^, S. Moretti^1^, V. Francavilla^1^, M. R. Pavone Cossut^1^, M. Campagna^1^, I. Schietroma^1^, F. Mancini^1^, R. Belli^1^, M. T. Maggiorella^1^, P. Monini^1^, B. Ensoli ^1^

^1^Istituto Superiore di Sanità (ISS), National HIV/AIDS Research Center (CNAIDS), RM, Italy

**Background: **Although antiretroviral therapy (cART) effectively suppresses HIV replication, it is unable to eradicate the virus, which persists in cART‐resistant reservoirs. New therapeutic interventions are, therefore, needed to improve cART effectiveness, and, possibly, to eradicate HIV. Preclinical and observational studies indicated that naturally occurring anti‐Tat antibodies block HIV replication, and are associated with slower progression to AIDS and better response to therapy.

**Methods: **One phase‐I (ISS T‐001) and 2 phase‐II (ISS T‐002 and ISS T‐003) Tat‐based therapeutic vaccine trials were conducted in naïve and cART‐treated people living with HIV (PLWH) in Italy and South Africa (395 volunteers). Each trial was followed by an extended observational study (up to 8 years) to evaluate the persistence of anti‐Tat immune responses and to monitor immunological and virological parameters.

**Results: **The Tat vaccine is safe in PLWH and promotes a long‐lasting anti‐Tat immunity, associated with significant improvements of immune system functions, even after long‐term cART (median of 6 years). These include increases of CD4^+^ T cells and CD4^+^/CD8^+^ T‐cell ratios, re‐equilibration of CD4^+^ and CD8^+^ T‐cell memory subsets with reduction of effector cells, improvement of T‐cell responses, and B‐cell and NK‐cell gains. This suggests a return to immune homeostasis that cART alone fails to restore. Strikingly, this was accompanied by a sustained proviral DNA decay, with a drastic reduction of the virus reservoirs (up to 90%) in blood after 8 years from vaccination with an average speed —four to seven times greater than observed in PLWH treated with cART alone. We are now investigating correlates of protection (functional anti‐Tat antibodies) and the long‐term effects of vaccination.

**Conclusions: **Results indicate that Tat is a key virulence factor, which plays a pivotal role in virus spreading and persistence, and that the induction of anti‐Tat immune responses represents a pathogenetic intervention to intensify cART efficacy, and to attack the ART‐resistant virus reservoir. Phase III studies are planned for vaccine registration.

### OAA1304 V2‐specific responses rescues SIV vaccine efficacy decreased by mucosal immunization with nanoparticles

M. A. Rahman ^1^, H. Scinto^1^, M. Bissa^1^, S. E. Howe^1^, S. Sarkis^1^, X. Jiang^2^, C. C. Luo^2^, A. Gutowska^1^, L. Schifanella^1^, R. Moles^1^, I. Silva de Castro^1^, M. Becerra Flores^2^, S. Basu^3,4^, K. F. N. N'guessan^3,4^, L. D. Williams^5,6^, X. Shen^5^, M. Doster^1^, T. Hoang^1^, E. Woode^1^, Y. Sui^1^, G. D. Tomaras^5,6^, D. Paquin Proulx^3,4^, M. Rao^3^, J. D. Talton^7^, X.‐P. Kong^2^, T. Cardozo^2^, S. Zolla Pazner^2^, J. A. Berzofsky^1^, G. Franchini^1^

^1^National Institutes of Health, Vaccine Branch, CCR, NCI, Bethesda, United States, ^2^New York University School of Medicine, New York, United States, ^3^Walter Reed Army Institute of Research, United States Military HIV Research Program, Silver Spring, United States, ^4^Henry M. Jackson Foundation for the Advancement of Military Medicine, Bethesda, United States, ^5^Duke University School of Medicine, Department of Surgery, Durham, United States, ^6^Duke University School of Medicine, Duke Human Vaccine Institute, Durham, United States, ^7^Alchem Laboratories, Alachua, United States

**Background: **Deletion of Env‐V1 region of the DNA/ALVAC/gp120/alum vaccine regimen improves efficacy compared to envelope‐replete immunogens and efficacy is linked to antibody responses to the envelope V2 region. We hypothesized that mucosal vaccination with V2‐peptide CKFNMTGLKRDKTKEYNETWYSTDLVCEQGNSTDNESRCYMNHC scaffolded as pentamers with Typhoid Toxin B subunit (V2‐TTB) might further improve efficacy, and poly(DL‐lactic‐co‐glycolic‐acid) nanoparticles (NPs) (<100 nm) was administered orally to deliver V2‐TTB‐NPs to the colon.

**Methods: **Rhesus macaques (*n* = 44) were immunized intramuscularly with the systemic ∆V1/DNA/ALVAC/gp120/alum vaccine regimen at weeks 0, 4, 8 and 12. At weeks 0, 4 and 16, one group (*n* = 12) also received oral immunizations with V2‐TTB‐NPs, while other groups (*n* = 9) received TTB‐NPs or empty‐NPs (*n* = 9). All animals were given 11 weekly intrarectal challenges with SIV_mac251_. Virological and immunological responses were analysed to understand their role in vaccine efficacy.

**Results: **Addition of V2‐TTB‐NP to the vaccine did not significantly reduce acquisition risk compared to the systemic vaccine, although the peak viral load and area‐under‐curve VL were both significantly reduced in animals that acquire SIV. Surprisingly, no vaccine efficacy was observed in animals vaccinated with the systemic vaccine plus either TTB‐NPs or empty NPs. V2‐TTB‐NP group exhibited higher antibody‐dependent cellular cytotoxicity (ADCC_ titres, deltaV1‐specific IgA rectal plasmablasts and plasmacytes, and IgG in rectal secretions after the last boost. Conversely, TTB‐NP or empty‐NP‐treated animals showed increased plasmacytoid dendritic cells (*p* = 0.056 and *p*<0.0001, respectively) and IFN‐γ^+^NKp44^−^NKG2A^−^ cells (*p*<0.0001 and *p*<0.0001, respectively) compared to that of V2‐TTB‐NP group. These cells were associated with increased acquisition in TTB NP group. On the other hand, V2‐specific ADCC (*p* = 0.01 and *p* = 0.01, respectively), mucosal CD14^+^ cells (*p* = 0.02 and *p*<0.0001, respectively) and NKp44^+^ cells (*p* = 0.004 and *p*<0.0001, respectively) were decreased in the TTB‐NP or empty‐NP groups versus V2‐TTB‐NP group and correlating with protection from acquisition in V2‐TTB‐NP group.

**Conclusions: **Collectively, the data indicate that despite some stronger mucosal V2‐ or deltaV1‐specific immune responses in V2‐TTB‐NP group, empty‐NPs and TTB‐NPs shifted innate mucosal immunity towards a “non‐protective” immune environment for ALVAC‐based HIV vaccine. Furthermore, the presence of V2 increases the “protective” responses compared to Nanoparticle alone groups. These data underscore the importance of both quality of innate responses and antibodies to V2 in protection against SIV/HIV acquisition.

### OAA1305 Safety and immunogenicity of a polyvalent DNA/polyvalent protein HIV vaccine with matched Env immunogens delivered as a prime‐boost regimen or co‐administered in adults without HIV

I. Frank ^1^, S. Li^2^, N. Grunenberg^2^, E. T. Overton^3^, S. T. Robinson^2^, H. Zheng^2^, K. E. Seaton^4^, J. R. Heptinstall^4^, M. A. Allen^5^, K. H. Mayer^6,7^, D. A. Culver^8^, M. C. Keefer^9^, S. Edupuganti^10^, M. N. Pensiero^5^, V. L. Mehra^5^, S. C. De Rosa^2^, D. E. Morris^2^, S. Wang^11^, M. S. Seaman^12^, D. C. Montefiori^13^, G. Ferrari^13,14^, G. D. Tomaras^4^, J. G. Kublin^2^, L. Corey^2^, S. Lu^11^, HVTN 124 Study Team

^1^University of Pennsylvania, Medicine/Infectious Diseases, Philadelphia, United States, ^2^Fred Hutchinson Cancer Center, Vaccine and Infectious Disease Division, Seattle, United States, ^3^University of Alabama, Division of Infectious Diseases, Birmingham, United States, ^4^Duke University, Center for Human Systems Immunology, Departments of Surgery, Immunology, and Molecular Genetics and Microbiology and Duke Human Vaccine Institute, Durham, United States, ^5^National Institutes of Health, Vaccine Research Program, Division of AIDS, National Institute of Allergy and Infectious Diseases, Bethesda, United States, ^6^Beth Israel Deaconess Medical Center, Division of Infectious Diseases, Boston, United States, ^7^Fenway Health, The Fenway Institute, Boston, United States, ^8^Cleveland Clinic, Department of Pulmonary Medicine, Respiratory Institute, and Department of Pathobiology, Lerner Research Institute, Cleveland, United States, ^9^University of Rochester School of Medicine & Dentistry, Department of Medicine, Rochester, United States, ^10^Emory University, Hope Clinic of the Emory Vaccine Center, Division of Infectious Diseases, Department of Medicine, School of Medicine, Decatur, United States, ^11^University of Massachusetts Medical School, Department of Medicine, Worcester, United States, ^12^Beth Israel Deaconess Medical Center, Center for Virology and Vaccine Research, Boston, United States, ^13^Duke University Medical Center, Duke Human Vaccine Institute and Department of Surgery, Durham, United States, ^14^Duke University, Department of Molecular Genetics and Microbiology and Department of Immunology, Durham, United States

**Figure**. OAA1305

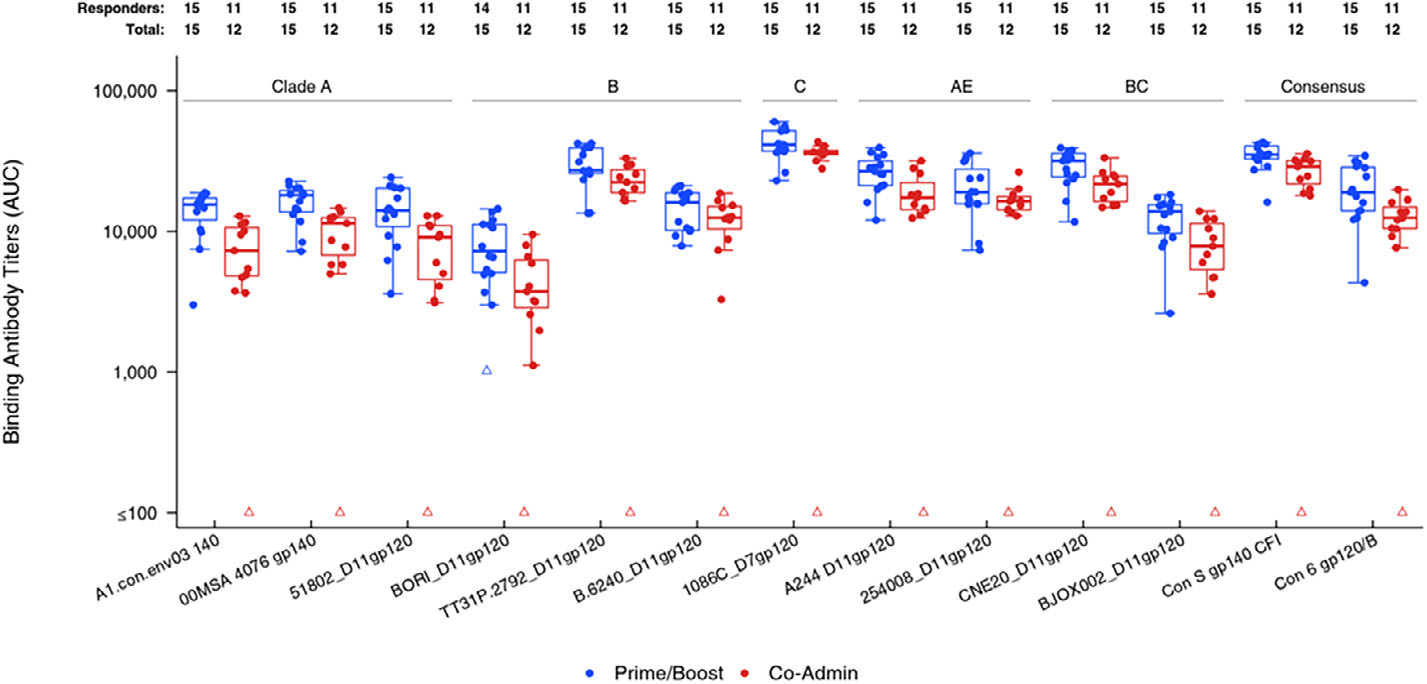

**Background: **An effective HIV vaccine will need to exhibit potent immunogenicity and broad cross‐subtype coverage. HVTN 124 evaluated the safety and immunogenicity of a unique polyvalent DNA‐protein HIV vaccine regimen with matching envelope (Env) immunogens.

**Methods: **HVTN 124 (NCT03409276) was a randomized, phase 1, placebo‐controlled, double‐blind study, including participants without HIV aged 18−50 years with low likelihood for HIV acquisition. The DNA vaccine comprised five plasmids: four copies expressing Env gp120 (clades A/B/C/AE) and one *gag* p55 (clade C). The protein vaccine included four DNA vaccine‐matched gp120 proteins adjuvanted with glucopyranosyl lipid adjuvantstable emulsion (GLA‐SE). Participants were randomized to placebo or one of two vaccine groups (prime‐boost or co‐administration). Vaccines were delivered via intramuscular injection. Part A (*N* = 12) evaluated the safety of the protein/adjuvant vaccine. Part B (*N* = 48) evaluated the safety and immunogenicity of two regimens: DNA prime (Months 0/1/3) with protein boost (Months 6/8), and DNA/protein co‐administration (Months 0/1/3/6/8). Part B per‐protocol participants were included in the immunogenicity analysis using samples collected 2 weeks after the last injection. All participants (intent‐to‐treat) were included in the safety analysis.

**Results: **Sixty participants (26 male, 34 female) were enrolled. Both vaccines were safe. Both regimens in Part B elicited IgG response rates >90% of high titre against a panel of heterologous gp120/gp140 antigens from diverse viral subtypes (Figure 1). High‐titre IgG responses were observed in 67−100% of participants against gp70‐V1V2 antigens from subtypes A, B, C and AE. Neutralizing antibody activity was detected against Tier 1A and Tier 1B viruses (clades B, C, AE and AG). Antibody‐dependent cellular cytotoxicity was detected in all of participants against AE CM235 and in ≥80% of prime‐boost recipients against other subtypes. Response rates and magnitudes for antibody‐mediated responses in the prime‐boost arm were similar to or exceeded those in the co‐administration arm. The prime‐boost regimen elicited Env‐specific CD4^+^ T‐cell responses in 15/15 participants versus 5/13 for co‐administration (*p* = 0.0002). IgG/IgG3 responses against gp70 V1V2, an established correlate of protection, were higher in the HVTN124 prime‐boost regimen than comparable regimens in the RV144, HVTN702 and HVTN705 trials.

**Conclusions: **This vaccine regimen was safe and highly immunogenic against multiple HIV clades, warranting evaluation in larger trials.

### OAA2802 Association of T‐resident memory and other CD4^+^ T‐cell subsets with HIV persistence across diverse tissue compartments from ART‐suppressed people with HIV

J. A. Neidleman ^1,2^, A. Chaillon^3^, A. F. George^1,2^, K. Yin^1,2^, X. Luo^1,2^, J. Frouard^1,2^, K. C. Young^1,2^, T. Ma^1,2^, A. Andrew^1,2^, I. Ezeonwumelu^1,2^, M. Calvet‐Mirabent^1,2^, S. Miliotis^1,2^, M. Porrachia^3^, B. Woodworth^3^, D. M. Smith^3^, S. Gianella^3^, N. R. Roan^1,2^

^1^J. David Gladstone Institutes, Gladstone Institute of Virology, San Francisco, United States, ^2^UCSF, Urology, San Francisco, United States, ^3^University of California, San Diego, Division of Infectious Diseases and Global Public Health, La Jolla, United States

**Background: **Memory CD4^+^ T cells are important long‐lived reservoirs for HIV. Yet, the phenotypic and functional features of these cells within tissue compartments of people with HIV (PWH) remain poorly characterized. Furthermore, the association of these features with HIV persistence remains unexplored.

**Methods: **We developed and applied a 40‐parameter CyTOF panel to deeply phenotype and quantitate subsets of memory CD4^+^ T cells from 26 different tissue specimens (including ileum, colon, rectum, para‐tracheal and aortic lymph nodes, spleen, liver, kidney, prostate, testes and bone marrow) from six ART‐suppressed PWH. Specimens were also analysed by digital droplet PCR for levels of cell‐associated total HIV DNA and unspliced RNA, which were associated with subset distribution using linear regression models. All participants were enrolled in the Last Gift Study, comprised of altruistic PWH who donate their bodies post‐mortem for research.

**Results: **Across tissue compartments, HIV DNA levels significantly associated with the frequencies of T‐resident memory (Trm, CD45RA‐CD45RO+CD69+) CD4^+^ T cells (*p* = 0.022), while a positive trend (*p* = 0.069) was observed for T follicular helper (Tfh) (CD45RA‐CD45RO+PD1+CXCR5+) CD4^+^ T cells. By contrast, HIV DNA levels trended negatively with the frequencies of T central memory (Tcm, CD45RA‐CD45RO+CCR7+CD27+) CD4^+^ T cells (*p* = 0.160). HIV RNA was more frequently detectable (Wilcoxon *t*‐test, *p* = 0.07) in tissue specimens with lower frequencies of Trm cells. Memory CD4^+^ T cells from distinct tissue compartments varied in expression of activation (HLADR, CD38), differentiation (CD127, CD57) and homing (CCR5, CCR6) markers.

**Conclusions: **Although Tcm from blood have previously been reported as a preferential reservoir for HIV, our data suggest that this may not be the case in tissues where Tcm frequencies negatively associated with HIV DNA levels. Instead, our data suggest that Tfh and especially Trm may be preferential tissue‐based reservoir cells among CD4^+^ T cells given their positive association with HIV DNA levels. Our observation that Trm frequencies associate positively with overall HIV DNA but negatively with detectable HIV gene expression (RNA) suggests that these cells may be a preferential latent tissue reservoir of HIV. Our data also revealed phenotypic features of memory CD4^+^ T cells specific to various tissue compartments.

### OAA2803 Concurrent depletion and dysfunction of tissue‐resident memory‐like CD4^+^ T cells in the small intestine coinciding with intestinal epithelial cell hyperproliferation in people living with HIV

O. Asowata^1^, S. Nyquist^2^, A. Langhave^3^, N. Mthabela^4^, L. Madziwa^4^, K. Chetty^4^, F. Karim^4^, V. Manzini^5^, F. Madela^5^, S. Khader^5^, A. Leslie^4^, A. Shalek^2^, H. Kløverpris ^4^

^1^AHRI, Durban, South Africa, ^2^Institute for Medical Engineering and Science, Department of Chemistry, Boston, United States, ^3^University of Copenhagen, Copenhagen, Denmark, ^4^Africa Health Research Institute, Basic and Translational Science, Durban, South Africa, ^5^Inkosi Albert Luthuli Central Hospital, Division Upper Gastrointestinal Tract and Colorectal Surgery, Durban, South Africa

**Background: **HIV infection impacts gut‐resident CD4^+^ T cells and gut homeostasis, both of which are central to the overall HIV‐associated pathology. Gut resident CD4^+^ T cells support epithelial cell renewal and differentiation in the intestinal crypt base. However, the differential impacts of HIV infection on CD4^+^ T‐cell levels and function in the small intestine versus the large intestine, as well as their effects on epithelial hyperproliferation, remain unknown.

**Methods: **Here, we studied the frequency and function of remaining tissue‐resident CD4^+^ T cells in the small and large intestine of PLWH in South Africa. Biopsies from >250 clinically indicated individuals from HIV endemic regions in South Africa were analysed for CD4^+^ T‐cell levels, and phenotyped by flow and mass cytometry and transcriptionally profiled after *ex vivo* live sorting. We used spatial profiling to identify HIV infection levels, CD4 T‐cell location and epithelial hyperproliferation by fluorescent microscopy.

**Results: **Tissue‐resident memory‐like (TRM‐like) CD69^+^CD103^+^CD4^+^ T cells expressed the HIV co‐receptor CCR5 and were enriched in the duodenum compared to the colon. In PLWH, these cells were preferentially depleted in the duodenum, but not in the colon. RNA sequencing of remaining duodenal CD4^+^ TRM‐like cells showed transcriptional downregulation of genes involved in proinflammatory cytokine signalling (IL‐8, IL‐6, IL‐2, GM‐CSF) and upregulation of genes involved in the IL‐17 and Th2 pathway in PLWH. In addition, integrin signalling was downregulated in the duodenum and consistent with loss of duodenal integrin CD103^+^ CD4^+^ T cells. In contrast, limited gene regulation was observed in the colon with limited CD4^+^ T‐cell depletion. In the non‐hemopoietic epithelial compartment within the same cohort, we found increased proliferation (Ki‐67) of duodenal crypt‐based cells colocalizing with OLFM4 expressing intestinal stem cells (ISCs), suggesting a direct link between CD4 TRM loss in the upper small intestine and ISC hyperproliferation.

**Conclusions: **HIV infection preferentially depletes TRM‐like CD4^+^ T cells in the small intestine, while the remaining cells show dysfunctional profiles. This may have implications for cytokine signalling to intestinal stem cells, a critical process for epithelial cell differentiation and gut barrier integrity in PLWH.

### OAA2804 Study of the transcriptional microenvironment and associated viral dynamics of the SIV tissue reservoirs during ART and post‐ATI

R. Lorenzo‐Redondo ^1,2^, M. S. Arif^3^, C. T. Thuruthiyil^3^, S. S. Pascoe^3^, M. A. Shaaban^1,2^, Y. Thomas^3^, J. M Hasson^1,2^, S. Samer^3^, M. R. Haque^3^, F. A. Engelmann^3^, M. D. McRaven^3^, M. Araínga^4^, L. Simons^1,2^, J. Hultquist^1,2^, E. Martinelli^1^, F. Villinger^4^, T. J. Hope^3^

^1^Northwestern University Feinberg School of Medicine, Department of Medicine (Infectious Diseases), Chicago, United States, ^2^Northwestern University Robert J. Havey, MD Institute for Global Health, Center for Pathogen Genomics and Microbial Evolution, Chicago, United States, ^3^Northwestern University Feinberg School of Medicine, Cell and Developmental Biology, Chicago, United States, ^4^University of Louisiana at Lafayette, New Iberia Research Center, New Iberia, United States

**Background: **Despite effective antiretroviral therapy (ART), HIV‐1 persistence is the major obstacle to a functional cure. To eliminate this viral reservoir, it is key to characterize reservoirs tissue microenvironment and associated viral population dynamics during ART, and after analytical treatment interruption (ATI).

**Methods: **We have developed immunoPET/CT‐guided bulk and spatial transcriptomics pipelines, combined with immunofluorescence detection of viral proteins and whole genome long‐read deep viral sequencing using the SIV/rhesus macaque model. For this study, rhesus macaques were intra‐venously challenged with SIVmac239, ART initiated at week 10 after infection and maintained for 54 weeks, followed by PET/CT‐guided necropsy after early ATI (4−7 days post‐ATI). ^64^Copper‐labelled probe against viral envelope efficiently detected infection sites as early as 4‐days post‐ATI and immunoPET/CT‐guided transcriptomics allowed the characterization of these PET/CT+ “hot” areas. Additionally, we performed viral long‐read deep sequencing and population dynamics analysis in the same tissue sections.

**Results: **SIV presence in early post‐ATI tissue reservoirs is associated with higher overall transcriptional levels and specific activation of genes associated with metabolic processes, cell localization and cytokine immune signalling. Moreover, we detect specific transcriptional clusters that are highly associated with active SIV rebounding foci. These foci are characterized by higher estimated presence of immune cells and upregulation of immune cell migration and immune activation. Analysis of intra‐host viral population dynamics from the same “hot” areas of the tissues indicates highly complex dynamics during ART and after ATI. Early after ATI, we observe similar viral quasispecies structure between different anatomical compartments indicating possible viral recirculation during ART or immediately after ATI. However, we observe significantly dissimilar patterns of viral diversification in different tissue reservoirs. Interestingly, the highest viral diversity levels are consistently detected in gut‐associated lymphoid tissue (GALT) reservoirs where our spatial transcriptomics show activation of genes associated with viral production and immune activation pathways.

**Conclusions: **Overall, we have demonstrated the extreme sensitivity of our newly developed methods to study the SIV reservoirs. We have identified various possible biomarkers of such reservoirs. The combination of these methods is providing a unique view of the virus‐host system that leads to viral persistence during ART and fast recovery of the viral populations immediately after ATI.

### OAA2805 Development of a novel approach to treat HIV reservoirs in gut‐associated lymphoid tissues using nanoformulation of cART drugs

D. Roy ^1^, A. Paul^1^, M. J. Haney^2^, M. M. Zarei^1^, H. Rodrigo^3^, E. V. Batrakova^2^, U. Roy^1^

^1^The University of Texas Rio Grande Valley, Health and Biomedical Science, Brownsville, United States, ^2^University of North Carolina, Chapel Hill, Department of Molecular Pharmaceutics, Chapel Hill, United States, ^3^The University of Texas Rio Grande Valley, Department of Mathematical & Statistical Sciences, Edinburg, United States

**Background: **Combination antiretroviral therapy (cART) has effectively controlled the viral load below the detection level and significantly extended the life of people with HIV. However, cART does not eliminate the virus from the host and certain cART drugs do not efficiently reach the HIV reservoirs of organs such as gut‐associated lymphoid tissue (GALT). Considering GALT is one of the major reservoirs of HIV, reducing the viral load within the tissue is important for a better outcome of the treatment. We propose that a formulation of three clinically available cART drugs, Emtricitabine, Tenofovir and Dolutegravir, into polymer‐based nanoparticles would significantly improve their accumulation in the GALT and increase drug therapeutic efficacy. Self‐assembling polymer micelles consisting of two block copolymers, Pluronic F127 and Pluyronic L61, were used as nanocontainers for cART drugs. Specifically, we hypothesized that cART nanoformulation targeted to Microfold cells (M cells) of the intestine would efficiently penetrate towards HIV reservoirs in GALT.

**Methods: **Various cART nanoformulations were manufactured and their accumulation in M cells was examined in *in vitro* and *in vivo* mouse models. The pharmacokinetics and therapeutic efficacy study in BALB/c mice and humanized mice have determined the targeted drug delivery and anti‐HIV activity of current formulations.

**Results: **The selected nanoformulations showed sustained drug release with no significant toxicity in different immune cells and *in vitro* M‐cell models. A single dose of cART nanoformulation had significantly higher retention in plasma compared to free drugs, showing a sustained release for 14 days. Finally, the selected nanoformulation of the cART combination was highly effective against HIV in GALT tissue.

**Conclusions: **Loading cART drugs into polymer‐based micelles provided significantly higher therapeutic efficacy against HIV in *in vitro* and *in vivo* models compared to free drugs. Thus, this approach may provide the next‐generation anti‐HIV medicine with targeted delivery to the GALT and better therapeutic efficacy with much fewer side effects with a once‐a‐week dosing schedule.

### OAA3502 Daily variations of CD4^+^ T‐cell counts and phenotype in relationship with residual viral transcription in the blood of people with HIV‐1 receiving antiretroviral therapy

A. Fert^1^, D. Chatterjee^1^, T. R. Wiche Salinas^1^, Y. Zhang^1^, D. Planas^1^, A. Cattin^1^, L. Raymond Marchand^2^, E. Moreira Gabriel^1^, C.‐D. Ngassaki‐Yoka^1^, J. Girouard^3^, A. Filali‐Mouhim^2^, N. Cermakian^4^, D. E. Kaufmann^1^, J.‐P. Routy^3^, P. Ancuta ^5^

^1^Université de Montréal and CHUM Research Centre, Montréal, Canada, ^2^CHUM Research Centre, Montréal, Canada, ^3^Chronic Viral Illness Service and Division of Hematology, Research Institute of the McGill University Health Centre, Montreal, QC, Canada, ^4^Douglas Research Centre, McGill University, Montreal, QC, Canada, ^5^Univrsité de Montréal and CHUM Research Centre, Microbiologie, infectiologie et immunologie, Montréal, Canada

**Background: **Biological functions exhibit circadian rhythms to align with day/night environmental changes. The circadian clock master regulators CLOCK/BMAL1 bind on E‐boxes in the promoters of clock‐regulated genes, including HIV‐1. Circadian variations in residual viral transcription were reported in people with HIV‐1 (PWH) receiving antiretroviral therapy (ART). Here, we investigated quantitative, phenotypic and transcriptional features of CD4^+^ T cells in relationship with daily variations in residual HIV‐1 transcription during ART.

**Methods: **Eleven male ART‐treated PWH were admitted at the CHUM Unit for Innovative Therapies for 36 hours. Blood was collected every 4 hours, before food intake, for 24 hours. Plasma levels of cortisol/melatonin and markers of intestinal permeability (FABP2, LBP) were measured by ELISA. Flow cytometry allowed the quantification/characterization of leukocytes. Sorted CD4^+^ T cells were used for cell‐associated integrated HIV‐DNA and Gag HIV‐RNA quantification by nested PCR/RT‐PCR and RNA‐Sequencing by Illumina technology. The impact of time on immunological/virological parameters was investigated using Cosinor and linear mixed models.

**Results: **Typical peaks in melatonin and cortisol levels were observed at 4:00 and 8:00, respectively. The highest FABP2 and lowest lipopolysaccharide binding protein (LBP) levels were observed at 4:00. CD4^+^ T‐cell counts were at nadir levels in the morning, but significantly increased at 20:00. This increase coincided with a superior expression of HIV‐1 co‐receptors CCR5/CXCR4, Th17 marker CCR6, gut‐homing ITGB7; follicular helper (CXCR5^+^PD‐1^+^) and effector memory (CD45RA^−^CCR7^−^) phenotype on memory CD4^+^ T cells; and robust changes in differential gene expression at 20:00 and 4:00 versus other timepoints. While integrated HIV‐DNA levels per million CD4^+^ T cells were stable, the proviral load normalized per ml of blood varied in a time‐dependent manner with the highest abundance at 20:00. The HIV‐RNA/DNA ratio (surrogate marker of HIV transcription) peaked at 4:00 and negatively correlated with levels of cortisol and LBP. Peak HIV‐RNA/DNA ratios coincided with unique transcriptional profiles encoding for regulators of T‐cell functions and viral transcription (SP6, Jun, BCL6, BCL3, SOCS3).

**Conclusions: **Our results reveal a massive influx of CD4^+^ T cells carrying proviral HIV‐DNA in the blood at 20:00, a peak in residual HIV‐1 transcription at 4:00 and a transcriptional signature associated with these daily immune/viral variations in ART‐treated PWH.

### OAA3503 Failure to achieve undetectable viral load caused by HIV expression from defective proviruses and large reservoir size

V. Hariharan ^1^, J. Siliciano^1^, R. Siliciano^1^, F. Simonetti^1^

^1^Johns Hopkins University, School of Medicine, Baltimore, United States

**Background: **Antiretroviral therapy (ART) halts viral replication and reduces plasma HIV RNA to undetectable levels in weeks to months in most PLWH. However, ART is not curative due to HIV persistence in memory CD4^+^ T cells. Some individuals experience non‐suppressible viraemia (NSV) despite adherence to ART, usually after years of undetectable viral load. Here, we characterized the source of NSV and reservoir composition in two PLWH who never reached undetectable viral load despite adherence and CD4 recovery.

**Methods: **We longitudinally sequenced plasma HIV RNA and proviral DNA in CD4^+^ T cells. We measured infected cell frequency using the intact proviral DNA assay (IPDA) and viral outgrowth assay. Additionally, we developed custom digital PCR assays to quantify lineage‐specific deletions, which we introduced into NL4‐3 to assess their impact on virion production and infectivity.

**Results: **Viral sequences contributing to NSV exhibited no known drug resistance mutations and showed no evolution. They were remarkably polyclonal, in contrast to previous reports of NSV. Furthermore, after 2 years on ART, total HIV DNA (29,000 and 11,000 proviruses/10^6^ CD4^+^ T cells; for P1 and P2, respectively) was >100‐fold greater than the median value from 400 ART‐suppressed PLWH (755 proviruses/10^6^ CD4^+^ T cells). For each participant, >90% of proviruses shared an identical deletion despite being diverse elsewhere in the genome, an unexpected finding given the lethal nature of the deletions. In Participant 1, we found two deletions affecting the open reading frames of *vif, vpr, tat, rev, vpu* and *env*; in participant 2, we found a 270‐nucleotide deletion in the first exon of *tat*. Proviruses with these deletions contributed between 40% and 70% to the NSV. These proviruses could be induced to make virions *ex vivo* but did not give rise to exponential outgrowth by qVOA. Finally, introducing these deletions into NL4‐3 resulted in >5‐log reduction in viral titre relative to the wild‐type.

**Conclusions: **Failure to achieve undetectable HIV RNA on ART can be caused by a large population of infected cells, including those carrying defective proviruses with deletions. We hypothesize that the expression, dissemination and diversification of these defective genomes is due to pre‐ART superinfection with intact proviruses.

### OAA3504 Modelling of ALT flares and liver inflammation in chronically SHIV/HBV co‐infected rhesus macaques

S. Biswas ^1^, S. Lutz^1^, S. Yusova^1^, J. Tran^1^, L. Rust^1^, S. Naldiga^1^, M. Fisher^1^, H. Crank^1^, J. Smedley^1^, J. Sacha^1^, B. Burwitz^1^

^1^Oregon Health & Science University, Pathobiology and Immunology, Beaverton, United States

**Background: **Twenty percent of individuals living with HIV also acquire hepatitis B virus (HBV) in their lifetime primarily due to common transmission routes. Rhesus macaques (RMs) have served as a model for HIV research for decades, but they have never been used to study HIV/HBV co‐infection. Our group has shown that RMs infected with SHIV_DH12 Clone 7_ and HBV_genotype D_ support chronic HIV/HBV co‐infection. A major advantage of the RM model is that we can obtain longitudinal biopsies and constantly monitor immune cell phenotyping and alanine aminotransferase (ALT) changes during the various stages of co‐infection; something that is impossible to obtain in clinical patients to understand co‐infection‐associated pathogenesis.

**Methods: **We infected RM intravenously with SHIV_DH12 Clone 7_ (5 × 10^3^ TCID_50_) followed by HBV (genotype D, 1 × 10^9^ virions, i.v) 3 weeks later. We next subcutaneously administered co‐infected RM with combination ART (cART) approximately 11 weeks post‐infection; consisting of Tenofovir (5.1 mg/kg), Emtricitabine (40 mg/kg) and Dolutegravir (2.5 mg/kg). Once the HBV viral loads went down to 10^2^ copies/ml, we replaced cART with Dolutegravir monotherapy (10 mg/kg). We collected weekly blood draws to monitor HBV/SHIV infection, and track CD4^+^ and CD8^+^ T cells, HBV surface antigens (HBsAg) and monitor ALT levels. We obtained longitudinal liver biopsies to monitor changes in liver immune cell population and investigate signs of fibrosis by immunohistochemistry.

**Results: **One RM from our pilot study exhibited co‐infection for >76 weeks making it the first ever animal to exhibit chronic HIV/HBV co‐infection. cART‐treated SHIV/HBV co‐infected RM suppressed HBV and SHIV viraemia with no effect on HBsAg levels. However, Dolutegravir monotherapy led to the rebound of HBV viraemia only. A major finding was the spike in ALT level during ART that was associated temporally with an increase in CD4^+^ T cells. Immunohistochemistry analysis is ongoing to detect signs of fibrosis in the chronically infected RM.

**Conclusions: **Thus, we present here the first ever RM model to exhibit long‐term SHIV/HBV co‐infection that exhibited similar outcomes of ART as observed in clinically co‐infected patients undergoing ART. Our data also show that we can model ALT flares during ART which would be crucial in understanding immune cell dynamics across the stages of co‐infection.

### OAA3505 Characterization of adipose tissue B‐cell antibodies in persons with diabetes and HIV

L. M. Obare ^1^, C. Wanjalla^1^, R. Gangula^1^, X. Zhang^1^, K. Nthenge^1^, W. J. McDonnell^2^, S. A. Smith^1^, S. Mallal^1^, J. R. Koethe^1^, S. Bailin^1^

^1^Vanderbilt University Medical Center, Infectious Diseases, Nashville, United States, ^2^Infinimmune, San Ramon, United States

**Background: **PLWH experience a 1.5‐ to 2‐fold higher incidence of cardiovascular disease not solely attributable to traditional risk factors. Changes in subcutaneous adipose tissue (SAT) immune cells contribute to inflammation and proatherogenic dyslipidaemia. B cells have been associated with cardiometabolic disease in the general population and are present in SAT. However, the role of B cells in cardiometabolic disease has not been studied in PLWH. We leveraged an existing cohort to investigate the changes in SAT B cells with cardiometabolic disease among PLWH.

**Methods: **SAT from PLWH with varying metabolic states and HIV‐negative participants with diabetes were obtained via liposuction. Single‐cell sequencing using cellular indexing of transcriptomes and epitopes (CITE‐seq) was employed to analyse immune and non‐immune cells in SAT. B‐cell receptor (BCR) sequences were analysed using the 10X/enclone genomics analysis. The top 20 clonal BCRs were synthesized as IgG isotypes, and enzyme‐linked immunosorbent assay against cytomegalovirus (CMV) antigens determined CMV specificity. Statistical analyses utilized GraphPad Prism software.

**Results: **We identified distinct B‐cell subsets, plasmablasts, naïve and memory B cells in SAT through CITE‐seq using well‐defined markers CD19, CD20, CD38 and CD27. Plasmablasts were significantly higher in prediabetic/diabetic PWH (Median 60 [IQR 20.3−88.9], *p* = 0.01), and diabetic HIV‐negative persons (50 [20.6−77.1], *p* = 0.003) compared to non‐diabetic PLWH (11.9 [5.3−33.3]). There was no difference in the proportion of memory B cells based on HIV or diabetes, while naïve cells were higher in non‐diabetic PWH (42.9 [21.9−63.4]) compared to diabetic PLWH (50 [0−28.6], *p* = 0.008) and trending towards significant in HIV negative with diabetes (15.48 [0−44.5], *p* = 0.06). BCR sequencing revealed that B cells expressing all four immunoglobulin isotypes (IgD, IgM, IgA and IgG) were present in SAT. Clonal B cells were more prevalent in the SAT of diabetic PWH compared with non‐diabetic PWH and diabetic HIV negative, suggesting differences in antigen‐driven responses within the SAT. Of the 20 clonal BCRs, only one clone among HIV‐positive individuals bound weakly to CMV.

**Conclusions: **Clonal B cells that accumulate in the SAT with diabetes, irrespective of HIV status, may be important in the pathogenesis of cardiometabolic disease.

### OAB0102 The effectiveness of different anal cancer screening strategies for people living with HIV/AIDS

Y. Liu^1^, A. Deshmukh^2^, K. Sigel^3^, M. Gaisa ^3^

^1^Icahn School of Medicine at Mount Sinai, New York, United States, ^2^College of Medicine, Medical University of South Carolina, Charleston, United States, ^3^Icahn School of Medicine at Mount Sinai, Department of Medicine, New York, United States

**Background: **People living with HIV/AIDS (PLWHA) have the highest incidence of human papillomavirus (HPV)‐associated anal cancer. The new anal cancer screening guidelines, published in 2024 by the International Anal Neoplasia Society, outline five screening strategies in high‐resource settings: anal cytology alone, high‐risk HPV (hrHPV) testing alone, cytology with hrHPV triage, hrHPV testing with cytology triage or cotesting. We aim to compare the effectiveness of these strategies in detecting anal cancer/precancer within a large cohort of PLWHA undergoing primary screening.

**Methods: **The study included 1620 PLWHA who underwent anal cytology, hrHPV testing and high‐resolution anoscopy (HRA)‐guided biopsy at our institution between 2012 and 2019. Using biopsy‐proven anal HSIL as an endpoint, we calculated sensitivity, specificity, positive predictive value (PPV), negative predictive value (NPV) and the number of HRA referrals triggered by each screening strategy.

**Results: **The median age was 45 years (range: 34−54), with 90% of the participants being men who have sex with men living with HIV/AIDS. Anal HSIL rate was 42%. The performance of each screening strategy is summarized in Table 1. All strategies showed comparable performance metrics. HrHPV testing alone demonstrated the highest sensitivity (96%), while hrHPV with cytology triage showed the highest specificity (48%). The approach of hrHPV with cytology triage, or vice versa, yielded the highest PPV (54%) and while hrHPV alone had the highest NPV (92%). The number of HRA referrals triggered by screening was highest for hrHPV alone (83%) followed by cytology alone (77%) and lowest for hrHPV with cytology triage (66%).

**Table 1:** OAB0102

Screening strategy	Results triggering HRA referral	Sensitivity (95% CI)	Specificity (95% CI)	PPV (95% CI)	NPV (95% CI)	# HRAs
**Cytology alone**	ASCUS or worse	88 (85−90)	30 (27−33)	48 (45−51)	77 (72−81)	1252 (77%)
**hrHPV alone**	hrHPV+	96 (95−97)	27 (25−30)	49 (47−52)	92 (88−95)	1341 (83%)
**Cytology with hrHPV triage**	ASCUS/hrHPV+	85 (82−88)	47 (44−50)	54 (51−57)	81 (78−84)	1080 (67%)
	LSIL/hrHPV+					
	ASC‐H/HSIL					
	All HPV16+					
**hrHPV with cytology triage**	hrHPV+/ASCUS or worse	85 (82−88)	48 (44−51)	54 (51−57)	81 (78−84)	1073 (66%)
	All HPV16+					
**Cotesting**	NILM/hrHPV+	89 (86−91)	40 (37−44)	52 (49−55)	83 (80−87)	1167 (72%)
	ASCUS/hrHPV+					
	LSIL/hrHPV+					
	ASC‐H/HSIL					
	All HPV16+					

**Conclusions: **All screening strategies outlined in the new guidelines demonstrate comparable effectiveness in detecting anal cancer and precancer among PLWHA. Nevertheless, the combined approach of cytology and hrHPV testing, whether utilized as cotesting or triage, proves more effective than cytology or hrHPV testing alone. Significantly, the incorporation of hrHPV testing increases specificity and results in a reduced number of HRA referrals, a critical consideration given the limited HRA capacity, even in high‐resource settings.

### OAB0103 Prevalence of high‐risk penile human papillomavirus in men who have sex with men and transgender women

D. Salusso ^1^, A. Gun^1^, A. Nava^1^, J. Garcia^1^, M. Abba^2^, M. E. Salas^2^, P. Delgado^1^, M. Golemba^1^, M. Moragas^1^, J. Naipauer^3^, C. F. Perez^1^, C. Cesar^1^, P. Cahn^1^, M. I. Figueroa^1^, V. Fink^1^

^1^Fundación Huésped, Research Department, Buenos Aires, Argentina, ^2^Facultad de Ciencias Médicas, Universidad de La Plata, Centro de Investigaciones Inmunológicas Básicas y Aplicadas, La Plata, Argentina, ^3^UBA‐CONICET, Instituto de Fisiología, Biología Molecular y Neurociencias, Buenos Aires, Argentina

**Background**: High‐risk HPV in anogenital locations can lead to preneoplastic and neoplastic lesions. HPV transmission occurs through genital‐genital or oral‐genital contact and penile HPV represents a potential reservoir. This study aims to evaluate the prevalence of penile HPV among men who have sex with men (MSM) and transgender women (TGW) populations.

**Methods**: A study on penile HPV was conducted at a Buenos Aires research centre, involving MSM and TGW, some participating in an ongoing anal and oral HPV study. Epidemiological and clinical data were collected and penile samples were obtained using a 600‐grit emery paper and a saline water‐imbibed Dacron swab. DNA was purified using the Biocomma Universal Genomic DNA Purification kit, and HPV was detected with ATILA (Genotyping High‐Risk HPV Real Time Fluorescent Detection).

**Results**: Between June 2022 and August 2023, 200 participants were enrolled. Median age: 33 (IQR 28−40); 98.5% (197) Hispanic‐Latino ethnicity; 13.5%(27) circumcised; 85%(171) had at least one previous sexually transmitted infection (STI): syphilis (63%), gonorrhoea (27.5%), chlamydiasis (21.5%), 36.5% (73) had HIV, median CD4 count: 417 cells/ml (IQR 315−664); 44% (88) had a history of anal or penile warts and 59% (69/113) anal intraepithelial lesions; 14.5% (29) had complete HPV vaccination. Baseline characteristics by group are shown in the table.

**Table:** Table. OAB0103

	MSM	TGW	
	(*n* = 164)	(*n* = 36)	*p* value
Tertiary education level	141 (86%)	10 (28%)	<0.001
History of STIs	137 (84%)	34 (94%)	0.092
HIV diagnosis	51 (31%)	22 (61%)	<0.001
Sexual work	31 (19%)	31 (86%)	<0.001
Lifetime insertive vaginal sex	89 (54%)	13 (36%)	0.048
Condom use in anal/vaginal insertive sex (more than 50%)	70 (47%)	21 (68%)	0.035
Condom use in oral insertive sex (less than 50%	156 (97%)	26 (77%)	<0.001

Penile HPV was detected in 69 participants (34.5%), with no differences between groups (MSM/TGW 33.5/38.9%). HPV genotypes distribution were: HPV‐33 (26%), HPV‐59 (17%), HPV‐35, HPV‐43, HPV‐45 (16%), HPV‐16 (15%), HPV‐56 (14%), HPV‐18, HPV‐31, HPV‐39 (10%), HPV‐52, HPV‐68 (9%), HPV‐58 (7%), HPV‐51 (4%), HPV‐66 (3%). Nineteen penile samples (10%) showed three or more concomitant genotypes. Among those with anal (*n* = 99) and oral (*n* = 103) samples, the HPV prevalence was 79% and 22%, respectively. HPV‐33 was the most frequent genotype in penile and anal samples. Participants with penile HPV had a higher frequency of anal infection (*p* = 0.02). No other statistically significant association was found.

**Conclusions: **One third of participants presented high‐risk HPV penile infection. An association between penile and anal HPV was found. These facts reinforce the potential role of the penis as a reservoir. Prevention strategies such as gender‐neutral vaccination and circumcision should be considered.

### OAB0104 Dynamic interplay of high‐risk human papillomavirus in women living with HIV: persistence, clearance, incidence and synergies with human T‐lymphotropic virus‐1 infections in a Kenyan teaching referal hospital

J. Kangethe ^1,2,3,4^, S. Gichuhi^5^, E. Odari^6^, J. Pintye^7^, K. Mutai^1^, M. Mureithi^8^

^1^Kenyatta National Hospital, Comprehensive Care Center for HIV, Nairobi, Kenya, ^2^Kenyatta National Hospital, Department of Medical Research, Training and Innovation, Nairobi, Kenya, ^3^University of Nairobi, Department of Medical Microbiology and Immunology, Nairobi, Kenya, ^4^Consortium for Advanced Research Training Research in Africa, Nairobikenya, Kenya, ^5^University of Nairobi, Department of Opthamology, Nairobi, Kenya, ^6^Jomo Kenyatta University of Agriculture and Technology, Nairobi, Kenya, ^7^University of Washington, Department of Biobehavioral Nursing and Health Informatics, Seattle, United States, ^8^University of Nairobi, Department of Medical Microbiology and Immunology, Nairobi, Kenya

**Background: **Cervical cancer (CC) is a major global health threat, especially in low‐ and middle‐income countries like Kenya. Women living with HIV (WLHIV) are more susceptible to high‐risk HPV (HR‐HPV) infections, increasing their risk of cervical cancer and co‐infections like HTLV‐1. Our study at Kenyatta National Hospital (KNH) explored HR‐HPV persistence, clearance and incidence, examining their interaction with HTLV‐1 in WLHIV on antiretroviral therapy (ART).

**Methods: **We conducted a prospective cohort study with 152 WLHIV, including 17 with HTLV‐1 co‐infections at KNH. After 12 months, cervical samples were retested for HR‐HPV using Gene Xpert® and HPV Genotypes 14 Real‐TM Quant. Data were analysed using SPSS 23.0, presenting outcomes as proportions, stratified by HR‐HPV genotypes and synergy with HTLV‐1. Descriptive comparisons and statistical tests assessed associations, with odds ratios reported for risk estimation.

**Results: **This prospective cohort study included 152 WLHIV who had initial HR‐HPV infections, among whom 17 were co‐infected with HTLV‐1 and provided consent. Participants had a mean age of 41.3 years (SD 8.7) and 29.6% had predictable virological failure (HIV 1 RNA ≥1000 copies/ml of plasma). The study revealed an overall HR‐HPV persistence rate of 89.5%, with a clearance rate of 10.5%. Notably, HR‐HPV 52 exhibited the highest persistence rate at 29.6%, followed by type 16 (22.4%) and 18 (19.1%), respectively. Statistical analysis demonstrated a significant association between age and HR‐HPV persistence, with rates of 86.8% and 13.2% for older and younger individuals, respectively (*p* < 0.001). HIV diagnosis at an older age (≥35 years) and a shorter duration of ART (<5 years) use were associated with HR‐HPV persistence, with rates of 60.3% versus 39.7% (*p* = 0.002) and 64.7% versus 35.3% (*p* = 0.004), respectively. Furthermore, co‐infection with HTLV‐1 was associated with a 100% HR‐HPV persistence rate, compared to an 88.8% rate among participants with HR‐HPV infections only.

**Conclusions: **The study revealed a significant 89.5% HR‐HPV persistence rate among WLHIV, with HR‐HPV 52, 16 and 18 showing elevated persistence. These findings underscore the importance of implementing the 9‐valent HPV vaccine in Kenya, particularly for WLHIV, and highlight a 100% type‐specific HR‐HPV persistence rate in the presence of HTLV‐1 co‐infection.

### OAB0105 Detection of *Treponema Pallidum* DNA for diagnosis, resistance identification and treatment outcome prediction in early syphilis among men who have sex with men

T.‐Y. Wu ^1^, L.‐H. Su^1^, H.‐Y. Sun^1^, W.‐D. Liu^1^, Y.‐S. Huang^1^, W.‐C. Liu^1^, K.‐Y. Lin^1^, S.‐Y. Chang^2^, C.‐C. Hung^1,3^

^1^National Taiwan University Hospital and National Taiwan University College of Medicine, Department of Internal Medicine, Taipei, Taiwan, Province of China, ^2^National Taiwan University Hospital and National Taiwan University College of Medicine, Department of Laboratory Medicine, Taipei, Taiwan, Province of China, ^3^National Taiwan University Hospital Yunlin Branch, Department of Internal Medicine, Yunlin, Taiwan, Province of China

**Background: **The study aimed to investigate the use of *Treponema pallidum* DNA (TP‐DNA) for diagnosis, resistance identification and treatment outcome prediction in early syphilis among men who have sex with men (MSM).

**Methods: **Adult MSM seeking care for sexually transmitted infections were prospectively enrolled from September 2021 to September 2023. The diagnosis of syphilis was made based on serologic testing and clinical presentations. Collected clinical samples, including oral rinse, rectal swab and urethral swab, were tested for TP‐DNA with the use of PCR assay targeting the 47 kDa gene. The cycle threshold (Ct) value of PCR assay was determined and the result was considered positive with a Ct value <38. Resistance‐associated mutations (RAMs) to macrolides and tetracyclines were identified. Serologic responses were compared between individuals with detected TP‐DNA and those without.

**Results: **During the study period, 570 MSM were enrolled, contributing to 386 tests for early syphilis, 169 treated syphilis and 87 without syphilis. TP‐DNA was detected in at least one clinical sample in 48.4% (187/386) of participants with early syphilis and 1.1% (1/87) of those without syphilis, resulting in a specificity of 98.9% and sensitivity 48.4%. TP‐DNA was most frequently detected in participants with secondary syphilis (71.8%), followed by those with primary and early latent syphilis (51.6% and 34.4%, respectively). The detection rate of TP‐DNA was higher in participants with rapid plasma regain (RPR) titres ≥1:32 compared with those with lower titres (54.0% vs. 19.4%, *p*<0.001). The rate of *T. pallidum* harbouring RAMs to macrolides (A2058G, A2058T and A2059G) was 55.6% (109/196), while the rate of RAMs to tetracyclines (G966T, C967T and C1192G) was 0.6% (1/156). Regardless of RPR titres and syphilis staging, participants with detected TP‐DNA had higher serologic responses compared to those without, with rates of 50.3% versus 35.2% at month 3 and 80.5% versus 66.5% at month 6.

**Conclusions: **Among MSM, the detection of TP‐DNA showed high specificity for early syphilis, which correlated with the stage of syphilis, RPR titres and treatment response. The prevalence of *T. pallidum* strains with RAMs to macrolides was >50%, warranting further close monitoring.

### OAB1702 Pharmacokinetics and HIV viral load suppression of 1 month‐daily rifapentine and isoniazid (1HP) for tuberculosis preventive therapy among adults with HIV taking standard dolutegravir‐based regimens

A. Avihingsanon ^1,2^, S. Jirajariyavej^3^, T. Palakawong Na Ayuthaya^4^, P. Treebupachatsakul^5^, J. Sophonphan^1^, J. Visuthranukul^6^, D. Danpornprasert^7^, W. Imsanguan^8^, P. Panarat^9^, S. Wiwatrojanagul^10^, P. Noopetch^11^, N. Natcha^12^, S. Gatechompol^1^, H. S. Lwin^1^, S. Thammapiwan^1^, P. Pingsusaen^1^, S. J. Kerr^1,13,14^, J.‐G. Shin​^15^, P. Chetchotisakd^16^, LTBI Study Group

^1^HIV‐NAT, Thai Red Cross AIDS Research Center, Bangkok, Thailand, ^2^Center of Excellence in Tuberculosis, Faculty of Medicine, Chulalongkorn University, Bangkok, Thailand, ^3^Taksin Hospital, Bangkok, Thailand, ^4^The Public Health Centre 28, Thon Buri, Thailand, ^5^Buddhachinnaraj Hospital, Pitsanulok, Thailand, ^6^Police General Hospital, Bangkok, Thailand, ^7^Klang Hospital, Bangkok, Thailand, ^8^Chiangrai Prachanukroh Hospital, Chiangrai, Thailand, ^9^Queen Savang Vadhana Memorial Hospital, Chonburi, Thailand, ^10^Maharat Nakhon Ratchasima Hospital, Nakhon Ratchasima, Thailand, ^11^Hatyai Hospital, Songkla, Thailand, ^12^Sisaket Hospital, Sisaket, Thailand, ^13^The Kirby Institute, University of New South Wales, Sydney, Australia, ^14^Biostatistics Excellence Centre, Faculty of Medicine, Chulalongkorn University, Bangkok, Thailand, ^15^Center for Personalized Precision Medicine of Tuberculosis, Inje University College of Medicine, Inje, the Republic of Korea, ^16^Faculty of Medicine, Srinagarind Hospital, Khon Kaen University, Khon Kaen, Thailand

**Background: **Ultrashort 1‐month of daily rifapentine 600/isoniazid 300 mg (1HP) is an effective and attractive Tuberculosis Preventive Therapy (TPT) regimen. However, co‐administration of 1HP and dolutegravir (DTG)‐based ART is limited mainly due to potential suboptimal DTG concentrations. Recent findings from A5372 and Taiwanese HIV cohort suggest the potential concomitant use of 1HP with standard dose DTG in Asian people with HIV (PWH). We, therefore, assessed safety, pharmacokinetics and HIV viral load suppression of once daily tenofovir disoproxil fumarate/lamivudine/DTG (TLD) when co‐administration with 1HP in antiretroviral (ARV) naïve and ARV experienced PWH.

**Methods: **We analysed data from an ongoing Phase 3 multicentre, randomized trial comparing 1HP and 3HP (weekly isoniazid 900 mg/rifapentine 900 mg for 12 weeks) among adult PWH (≥18 years) without evidence of active tuberculosis (TB) from 14 HIV clinics in Thailand. 1HP was initiated 2−4 weeks after starting TLD. Dolutegravir concentrations were collected at day 0 (before 1HP) and day 28. Dolutegravir concentrations were analysed at Center for Personized Precision Medicine of Tuberculosis, Inje University College of Medicine, South Korea.

**Results: **Two hundred and fifty‐two PWH (81% male, median age 32 [IQR: 26−41] years, BW 64.9 [IQR: 56.5−73.8] kg) on TLD and 1HP were analysed. Median CD4 cell count was 451 (IQR: 287−682) cells/mm^3^, 202 (80%) were ARV naïve (no baseline VL) and 20% participants were ARV experienced with pre‐1HP HIV VL < 50 copies/ml. A total of 99.2% participants completed the 1HP; two (0.8%) permanently discontinued treatment due to possible 1HP‐related adverse events. Hypersensitivity reaction occurred in 0.8%. Grade 3 and 4 asymptomatic hepatitis developed in 0.4%, and 1.2% serious adverse events (SAEs) were reported. HIV RNA < 50 copies/ml at 24 and 48 weeks were 95.2%and 97.7%, respectively. The geometric mean (95% CI) dolutegravir trough concentrations were 0.56 mg/dl (0.27−1.16) on day 0 versus 0.15 mg/dl (0.09−0.24) on day 28. Almost 92.6% had DTG Ctrough > 0.064 mg/dl.

**Conclusions: **Although there were substantial reductions in DTG Ctrough, almost 93% of participants had DTG C trough concentration above the protein‐binding‐adjusted IC90. Co‐administration of 1HP and TLD were well tolerated, with robust high rates of HIV virological suppression. These findings highlight the potential co‐administration of 1HP with standard dose TLD.

### OAB1703 High incidence of tuberculosis in young children living with HIV in the Western Cape, South Africa

K. Anderson (Melunsky) ^1^, H. Rabie^2^, B. S. Eley^3^, L. Frigati^2^, J. Nuttall^3^, E. Kalk^1^, A. Heekes^4^, M. Smith^4^, A. Boulle^1,4,5^, V. Mudaly^6^, M.‐A. Davies^1,4,5^

^1^University of Cape Town, Centre for Infectious Disease Epidemiology and Research, School of Public Health, Cape Town, South Africa, ^2^Tygerberg Hospital, Stellenbosch University, Department of Paediatrics and Child Health, Stellenbosch, South Africa, ^3^Red Cross War Memorial Children's Hospital, University of Cape Town, Department of Paediatrics and Child Health, Cape Town, South Africa, ^4^Western Cape Department of Health and Wellness, Health Intelligence, Cape Town, South Africa, ^5^University of Cape Town, Division of Public Health Medicine, School of Public Health, Cape Town, South Africa, ^6^Western Cape Department of Health and Wellness, Service Priorities Coordination, Cape Town, South Africa

**Background: **We examined tuberculosis (TB) trends among children living with HIV (CLHIV), age ≤5 years, in Western Cape, South Africa. Early infant HIV testing and early antiretroviral therapy (ART) initiation is implemented in this high HIV and TB setting.

**Methods: **We analysed routinely collected healthcare data for CLHIV born May 2018−October 2022 (database closure mid‐2023). We examined factors associated with TB diagnosis using Fine‐Gray competing risk models (death and loss to follow‐up as competing events), adjusted for sex, birth year and previous TB.

**Results: **We included 2219 CLHIV; 30% diagnosed with HIV at age ≤7 days, 41% at age 8−365 days and 29% at age >1 year. Median follow‐up from birth was 38 months (IQR 24−50); 90% of CLHIV started ART. TB was diagnosed in 28% of CLHIV (*n* = 626/2219); 62% first diagnosed before/within 3 months of ART start (“TB before ART”) and 38% >3 months after ART start (“TB after ART”). Of those with “TB before ART” (*n* = 390), median age at HIV diagnosis was 13 months (IQR 6−22) and median time from HIV diagnosis to TB diagnosis was 5 days (IQR 0−31). “TB before ART” was significantly associated with older age at HIV diagnosis and advanced/severe immunodeficiency. Of those with “TB after ART” (*n* = 258), median age at HIV diagnosis was 2 months (IQR 0−8) and median time from ART start to TB diagnosis was 12 months (IQR 7−21). “TB after ART” was associated with increased viral load and advanced/severe immunosuppression (time‐updated) but not age at ART start. Even low‐level viraemia (500−999 vs. <100 copies/ml) was associated with “TB after ART” (aSHR 2.75; 95% CI 1.05−7.18). Overall, 5% (*n* = 112/2219) of CLHIV died, 36% of whom were diagnosed with TB (median time from TB diagnosis to death: 58 days; IQR 17−191).

**Conclusions: **Young CLHIV in this setting have high TB‐associated morbidity and mortality. Susceptible groups include: CLHIV diagnosed with HIV and TB concurrently at an older age, associated with advanced/severe immunodeficiency; and CLHIV who, despite starting ART in early infancy, develop TB later, associated with advanced/severe immunodeficiency and elevated viral load. Efforts to improve early HIV diagnosis, viral suppression and TB preventive therapy are needed.

### OAB1704 Single‐dose liposomal amphotericin to prevent meningitis in HIV‐associated cryptococcal antigenemia with low serum Crag titers

E. K. Nalintya ^1^, D. B. Meya^2^, C. P. Skipper^3^, P. Kirumira^1^, P. Ayebare^1^, R. D. Naluyima^1^, T. Namuli^1^, F. Turya^1^, S. Walukaga^3^, A. Wele^4^, B. Dai^4^, D. R. Boulware^3^, R. Rajasingham^3^

^1^Infectious Diseases Institute, College of Health Sciences, Makerere University, Kampala, Uganda, ^2^Department of Medicine, School of Medicine, College of Health Sciences, Makerere University, Kampala, Uganda, ^3^Division of Infectious Diseases and International Medicine, Department of Medicine, University of Minnesota, Minneapolis, United States, ^4^Division of Biostatistics and Health Data Science, University of Minnesota, Minneapolis, United States

**Background: **Cryptococcal meningitis is a leading cause of AIDS‐related mortality. Cryptococcal antigen (CrAg) predicts the development of meningitis. Historically, despite standard‐of‐care fluconazole, 25−30% of asymptomatic CrAg‐positive persons develop breakthrough meningitis or death. Recently, single‐dose liposomal amphotericin (10 mg/kg) was recommended as first‐line therapy for the treatment of cryptococcal meningitis by the World Health Organization. We evaluated whether a single high dose of liposomal amphotericin could prevent cryptococcal meningitis and death in persons with asymptomatic cryptococcal antigenemia.

**Methods: **Participants with HIV and asymptomatic cryptococcal antigenemia in Uganda were randomized to one dose of liposomal amphotericin (10 mg/kg) with fluconazole or fluconazole alone through 24 weeks (clinicaltrials.gov NCT03945448). Participants were enrolled from two sites in Uganda from April 2019 to April 2022. Twenty‐four‐week meningitis‐free survival was compared between treatment groups using a log‐rank test. Hazard ratio was estimated using a univariate Cox proportional hazard model. Upon review of the interim data, the data safety and monitoring board (DSMB) recommended no further enrolment of participants with low serum CrAg titres (≤1:80). Herein, we present the results among participants with a low serum CrAg titre.

**Results: **We enrolled 251 of 593 CrAg‐positive participants screened, of whom 168 had a low CrAg titre (≤1:80). Median CrAg titre and baseline characteristics were comparable between the two groups. During 24 weeks of follow up, clinical events occurred in 14.5% (12/83) of participants randomized to liposomal amphotericin with fluconazole versus 10.6% (9/85) assigned to fluconazole alone (ARR = 3.9%; 95% CI, −6.13% to 13.9%; *p* = 0.45) with a hazard ratio of 1.42 (95% CI, 0.60−3.36; *p* = 0.43). Clinical adverse events were more common in the intervention group; however, no specific adverse event was more notable in one group or the other. There was no difference between the occurrence of grade 3 and 4 laboratory adverse events across the two groups (liposomal amphotericin 24% [20] vs. standard‐of‐care 24% [20]; *p* = 0.93).

**Conclusions: **Among CrAg‐positive persons with low titres (≤1:80), the addition of single‐dose liposomal amphotericin to fluconazole as pre‐emptive therapy provided no additional clinical benefit.

### OAB1705 Prevalence of isolated cryptococcal antigenemia and efficacy of preemptive fluconazole treatment in people living with HIV in China

Y. Lu ^1^, Y.‐K. Chen^1^

^1^Chongqing Public Health Medical Center, Department of Infectious Diseases, Chongqing, China

**Background: **Studies have shown that isolated cryptococcal antigenemia (ICA) is a risk factor for death or cryptococcal meningitis (CM) in PLWH, and that pre‐emptive antifungal treatment for ICA is vital for the prevention of CM or death in this population. However, there is a dearth of published studies concerning the prevalence of ICA and pre‐emptive fluconazole utilization in PLWH in China.

**Methods: **This was a multicentre, retrospective study at eight hospitals in China. Data from Jan 2019 to Dec 2022 were retrospectively analysed. CrAg‐positive PLWH received extensive laboratory and radiological examinations to evaluate the presence of underlying cryptococcal disease. Those with CM, pulmonary cryptococcosis (PC) or cryptococcemia, and those having less than 1 year of follow‐up were excluded from the analysis. Only PLWH with ICA were enrolled in our study.

**Results: **A total of 14,678 PLWH underwent serum CrAg testing. Of these, 369 with CM, 101 with PC and 67 with cryptococcemia were excluded, and of the remaining 10,649 PLWH, the data of 433 with ICA were pooled and analysed. In our cohort, the overall prevalence of ICA was observed to be 4.1%, and the prevalence of ICA in patients with CD4^+^ T‐cell counts of <200 cells/µl was 5.2%. Additionally, the prevalence of ICA in patients with CD4^+^ T‐cell counts of <100 cells/µl was 6.6%. Interestingly, we observed five further PLWH with ICA in a subset of 1260 individuals having CD4^+^ T‐cell counts ranging from 200 to 500 cells/µl. When comparing one group without pre‐emptive fluconazole treatment and the other with pre‐emptive fluconazole treatment, we found that the cumulative incidence of CM and/or death in these groups was lower in the pre‐emptive fluconazole treatment group (8.6% vs. 19.0%, *p* = 0.144), and patients who did not receive pre‐emptive fluconazole treatment had a significantly higher risk of CM and/or death (aHR: 3.035, 95% CI, 1.067−8.635; *p* = 0.037).

**Conclusions: **Our results demonstrate that overall ICA prevalence in our PLWH cohort exceeded the threshold of 3%, indicative of a high endemic rate of cryptococcal infection in PLWH in China. Pre‐emptive fluconazole treatment is an effective strategy for reducing the risk of CM and death in China.

### OAB2102 Randomized placebo‐controlled trial of high‐dose vitamin D and low‐dose calcium supplementation to improve bone density in adolescents with perinatally acquired HIV in Southern Africa

R. Ferrand ^1,2^, N. Dzavakwa^1,2^, L. Kasonka^3^, H. Banda‐Mabuda^3^, T. Bandason^2^, M. Chisenga^3^, S. Filteau^1^, K. Kranzer^1,2^, H. Mujuru^4^, U. Schaible^5^, S. Rowland‐Jones^6^, V. Simms^1,2^, C. Gregson^2,7^, VITALITY Trial Group

^1^London School of Hygiene and Tropical Medicine, London, United Kingdom, ^2^Biomedical Research and Training Institute, Harare, Zimbabwe, ^3^University Teaching Hospital, Lusaka, Zambia, ^4^University of Zimbabwe, Harare, Zimbabwe, ^5^Research Centre Borstel, Leibniz Lung Centre, Borstel, Germany, ^6^University of Oxford, Oxford, United Kingdom, ^7^University of Bristol, Bristol, United Kingdom

**Background: **HIV adversely affects skeletal development in children despite antiretroviral therapy (ART). Vitamin D deficiency, highly prevalent among children with HIV in Africa, has a further adverse impact on bone health. We investigated whether adjuvant vitamin D_3_ and calcium carbonate supplementation improves bone density among adolescents with HIV.

**Methods: **We conducted a multi‐country individually randomized, double‐blinded placebo‐controlled trial of weekly high‐dose (20,000IU) vitamin D_3_ plus daily calcium carbonate (500 mg) supplementation for 48 weeks. Adolescents with HIV aged 11−19 years taking ART for ≥6 months were recruited from HIV clinics in Zimbabwe and Zambia. The primary outcome was total body less‐head bone mineral density (TBLH‐BMD) Z‐score using a UK reference population, measured by dual‐energy X‐ray absorptiometry (DXA). Lumbar spine bone mineral apparent density (LS‐BMAD) Z‐score was a secondary outcome. Linear regression was used to compare arms adjusting for site and baseline value of the measure. Pre‐specified sub‐group analyses by age‐group, sex, pubertal stage and baseline vitamin D insufficiency (defined as 25(OH)D level <75 nmol/l) were performed.

**Results: **Eight hundred and forty‐two participants, 448 (53.21%) female, were enrolled. 75.9% were vitamin D insufficient. Baseline characteristics were balanced between arms; at 48 weeks, DXA outcomes were available for 751 (89.2%) participants.

There was no difference by arm in the primary outcome (mean 48‐week TBLH‐BMD Z‐score −1.56 [SD 1.12] in intervention arm vs. −1.53 [1.18] in the control arm, adjusted mean difference [AMD] −0.03 [95% CI −0.08, 0.02]). Results were similar for LS‐BMAD Z‐score (mean −0.71 [1.16] vs. −0.64 [1.19]; AMD −0.04 [95% CI −0.11, 0.02]).

However, among participants with vitamin D insufficiency, there was a significant difference by arm in both TBLH‐BMD‐ and LS‐BMAD‐ Z‐scores (Table 1). There was no evidence of effect in other subgroups. No drug‐related severe adverse events were observed.

**Table 1:** OAB2102: Outcomes by baseline vitamin D_3_ level

		TBLH‐BMD Z‐score				LS‐BMAD Z‐score			
Baseline 25(OH)D level	*N*	Control arm mean (SD)	Intervention arm mean (SD)	Adjusted mean difference (95% CI)	*p*‐value	Control arm mean (SD)	Intervention arm mean (SD)	Adjusted mean difference (95% CI)	*p*‐value
<75 nmol/l	562	−1.53 (1.22)	−1.61 (1.13)	−0.06 (−0.11, −0.01)	0.027	−0.67 (1.19)	−0.72 (1.13)	−0.09 (−0.17, −0.02)	0.016
≥75 nmol/l	189	−1.52 (1.03)	−1.45 (1.12)	0.05 (−0.07, 0.16)	0.44	−0.51 (1.08)	−0.70 (1.24)	0.10 (−0.03, 0.23)	0.13

**Conclusions: **High‐dose vitamin D_3_ and low‐dose calcium supplementation, a safe, easily available and cheap intervention, during adolescence (a period of rapid growth) may promote bone accrual and mineralization towards maximizing peak bone mass, which may reduce the risk of fractures among children growing up with HIV.

### OAB2103 Neurocognitive trajectories among a young adult cohort from four African countries: associations with HIV and food insecurity

S. Frndak ^1,2^, E. Tsoy^3^, N. Dear^1,2^, H. Kibuuka^4^, J. Owuoth^5,6^, V. Sing'oei^5,6^, J. Maswai^1,7^, E. Bahemana^1,8^, V. Anyebe^1,9^, Z. Parker^1,2^, J. S. Cavanaugh^1^, N. Shah^1^, T. A. Crowell^1,2^, J. A. Ake^1^, V. Valcour^3^

^1^U.S. Military HIV Research Program, Walter Reed Army Institute of Research, Silver Spring, United States, ^2^Henry M. Jackson Foundation for the Advancement of Military Medicine, Inc., Bethesda, United States, ^3^University of California San Francisco, Department of Neurology: Memory and Aging Center, San Francisco, United States, ^4^Makerere University Walter Reed Project, Kampala, Uganda, ^5^U.S. Army Medical Research Directorate—Africa, Kisumu, Kenya, ^6^HJF Medical Research International, Kisumu, Kenya, ^7^U.S. Army Medical Research Directorate—Africa, Kericho, Kenya, ^8^HJF Medical Research International, Mbeya, the United Republic of Tanzania, ^9^HJF Medical Research International, Abuja, Nigeria

**Background: **HIV and food insecurity (FI) compound cognitive impairment in adulthood. It is unclear how HIV and FI may impact neurodevelopment in young adults.

**Methods: **The African Cohort Study (AFRICOS) prospectively enrols people with (LWH) and without (LWoH) HIV, aged ≥15 years, in care at 12 PEPFAR‐supported facilities in Kenya, Nigeria, Tanzania and Uganda. Participants were administered neuropsychological tests annually including the WHO/NIMH Auditory‐Verbal Learning (AVLT) total recall (verbal memory), Trail Making (TMT) time (processing speed) and Grooved Pegboard (GP) time (motor speed and dexterity). FI was self‐reported as not enough to eat or <3 meals per day. We restricted the sample to participants <26 years old at first neuropsychological test and on antiretroviral therapy ≥6 months if LWH. Linear mixed models (LMMs) assessed baseline HIV status and time‐varying FI on cognition longitudinally across age. Interactions included: FI*HIV, gender*FI, gender*HIV, FI*age, HIV*age. Age interactions assessed differences in rate of cognitive score change. All models controlled for gender, household income, education and prior tuberculosis infection.

**Results: **Young adults (*n* = 902) were primarily female (56%), LWH (74%) and 29% were FI, with four visits on average. Overall, increasing age was associated with better scores. Longitudinally, those LWoH scored better than those LWH (total recall β = 1.03; TMT time β = −5.55; GP time β = −0.12; all *p*≤0.05). Females scored better than males LWH on total recall longitudinally (interaction *p*<0.01), with no gender difference for those LWoH. For FI*age, FI was associated with a greater positive rate of change in total recall (interaction *p* = 0.002) (Figure). No FI*HIV interactions were significant and rates of change for TMT and GP were not different by FI or HIV status.

**Figure**. OAB2103

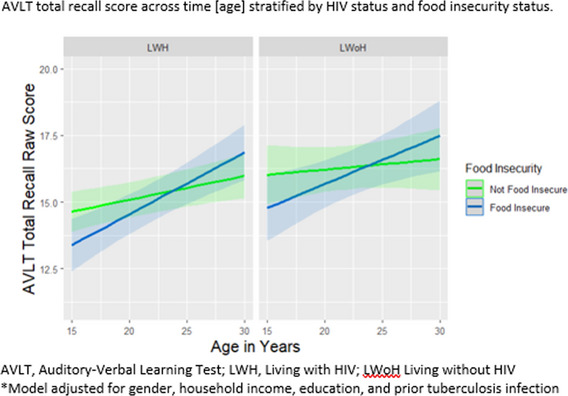

**Conclusions: **FI was associated with a greater positive rate of change in total recall among both treatment‐experienced young adults LWH and LWoH, possibly reflecting developmental catch‐up resulting from clinical care.

### OAB2104 Increased biomarkers of cardiovascular disease in a long‐term survivor cohort of young adults living with perinatal HIV with virologic non‐suppression or metabolic syndrome

L. Aurpibul ^1^, P. Lumbiganon^2^, P. Ounchanum^3^, K. Chokephaibulkit^4^, T. Sudjaritruk^5,6^, P. Kosalaraksa^2^, S. Rungmaitree^4^, W. N. Songtaweesin^7,8^, T. Suwanlerk^9^, S. Kawichai^7^, J. L. Ross^9^, T. Puthanakit^7^

^1^Chiang Mai University, Research Institute for Health Sciences, Chiang Mai, Thailand, ^2^Khon Kaen University, Department of Pediatrics, Faculty of Medicine, Srinagarind Hospital, Khon Kaen, Thailand, ^3^Chiangrai Prachanukroh Hospital, Department of Pediatrics, Chiang Rai, Thailand, ^4^Mahidol University, Department of Pediatrics, Faculty of Medicine Siriraj Hospital, Bangkok, Thailand, ^5^Chiang Mai University, Department of Pediatrics, Faculty of Medicine, Chiang Mai, Thailand, ^6^Chiang Mai University, Clinical and Molecular Epidemiology of Emerging and Re‐emerging Infectious Diseases Research Cluster, Faculty of Medicine, Chiang Mai, Thailand, ^7^Chulalongkorn University, Department of Pediatrics, Faculty of Medicine, Bangkok, Thailand, ^8^Chulalongkorn University, Bangkok, Thailand, School of Global Health, Faculty of Medicine, BangkokThai, Thailand, ^9^TREAT Asia/amfAR—The Foundation for AIDS Research, Bangkok, Thailand

**Background: **With age and duration of antiretroviral therapy (ART), young adults with perinatally acquired HIV (YA‐PHIV) are at risk for cardiovascular disease. Inflammatory biomarkers including high sensitivity C‐reactive protein (hs‐CRP), interleukin (IL)‐18 and soluble CD163 (sCD163) have been associated with increased cardiovascular morbidity and mortality. We assessed biomarker levels and their association with HIV viraemia and metabolic syndrome in a long‐term cohort of YA‐PHIV.

**Methods: **YA‐PHIV, who started ART in paediatric clinics at five sites in Thailand aged 18−25 years, were recruited from November 2020 to July 2021. Clinical assessments and blood sampling for hs‐CRP, IL‐18 and sCD163 levels were performed. Metabolic syndrome was defined using National Cholesterol Education Program‐Adult Treatment Panel III criteria. The hs‐CRP levels between 1.0 to < 3.0 and ≥ 3 mg/l were defined as intermediate and high risk for cardiovascular disease, respectively. Biomarker association with HIV viraemia and metabolic syndrome were assessed using Mood's median non‐parametric test.

**Results: **Of the total 347 YA‐PHIV, 187 (54%) were biological females. At enrolment, median age and duration on ART were 21.8 years (interquartile range, IQR 20.1−23.5) and 16.7 years (IQR 13.4−18.4), respectively. Their median CD4 was 564 cells/mm^3^ (IQR 356−753), 14% had CD4 count <200 cells/mm^3^, 19% had HIV‐RNA >1000 copies/ml and 7.8% had metabolic syndrome.

Overall, 25% had hs‐CRP levels associated with intermediate and 26% with high risk for cardiovascular disease; levels were similar (24% and 23%) in those with HIV‐RNA <1000 copies/ml. The median serum IL‐18 was 82.4 pg/ml (IQR 33.8−151.9) and sCD163 was 53.6 ng/ml (IQR 31.1−90.1). YA‐PHIV with HIV‐RNA >1000 had significantly higher hs‐CRP (*p* = 0.001), IL‐18 (*p* <0.001) and sCD‐163 (*p* = 0.003) than those with HIV‐RNA <1000 copies/ml. YA‐PHIV with metabolic syndrome were more likely to have higher median hs‐CRP (*p* = 0.008) and a trend towards higher sCD163 (*p* = 0.07) than those without, but there was no difference in IL‐18 levels. Higher median IL‐18 was observed in males than females.

**Conclusions: **YA‐PHIV with HIV‐RNA >1000 copies/ml had increased biomarkers of cardiovascular disease, and those with metabolic syndrome had increased hs‐CRP and sCD163. Further research on the predictive value of these biomarkers on cardiovascular outcomes in ageing YA‐PHIV is warranted.

### OAB2105 Biomarker‐confirmed, unhealthy alcohol use among adolescents and young adults with HIV is associated with viral non‐suppression in Uganda and Kenya in the SEARCH‐Youth study

F. Mwangwa ^1^, S. Puryear^2^, M. Nyabuti^3^, D. Black^4^, J. Peng^5^, E. Bukusi^3^, L. B. Balzer^6^, C. Camlin^7^, J. Ayieko^8^, T. Ruel^9^, G. Chamie^9^, M. R. Kamya^10^, D. V. Havlir^9^, J. Hahn^2^

^1^Infectious Diseases Research Collaboration, Kampala, Uganda, ^2^University of California San Francisco, Division of HIV, ID and Global Medicine, San Francisco, United States, ^3^Kenya Medical Research Institute, Research, Nairobi, Kenya, ^4^University of California San Francisco, Division of HIV, San Francisco, United States, ^5^University of Washington, School of Public Health, Department of Biostatistics, Seattle, United States, ^6^University of Massachusetts, Amherst, School of Public Health and Health Sciences, Amherst, United States, ^7^University of California San Francisco, Center for AIDS Prevention Studies, San Francisco, United States, ^8^Kenya Medical Research Institute, Adult and Adolescent Studies, Nairobi, Kenya, ^9^University of California San Francisco, School of Medicine, San Francisco, United States, ^10^Makerere University, Department of Medicine, Kampala, Uganda

**Background: **We previously demonstrated in a clinic‐cluster randomized trial that the multilevel SEARCH‐Youth intervention, which included life‐stage assessments, person‐centred care and rapid viral load feedback, improved HIV outcomes among adolescents and young adults with HIV (AYAH) aged 15−24 in Uganda and Kenya. In a cross‐sectional study, we evaluated whether alcohol use predicted viral non‐suppression.

**Methods: **We conducted a survey on alcohol use and tested for phosphatidylethanol (PEth), a blood‐based biomarker of prior month alcohol consumption, among intervention participants. We defined unhealthy alcohol use as Alcohol Use Disorders Identification Test—Consumption (AUDIT‐C, prior 3 months) positive (≥3 for women, ≥4 for men) OR PEth≥50 ng/ml, and explored the association between unhealthy alcohol use and viral non‐suppression (≥400 copies/ml) using logistic regression, adjusting for gender, age and country.

**Results: **Unhealthy alcohol use was assessed in 718 AYAH: 409 (57%) in Uganda and 309 (43%) in Kenya. Eighty percent were female, median age 24 years, and 95% were virally suppressed at the time of the survey and PEth measures, 30−36 months after enrolment. The prevalence of unhealthy alcohol use was 24.8% and more common among males, older AYAH and participants in Uganda (Table). Participants with unhealthy alcohol use had 2.8 times higher odds of viral non‐suppression (adjusted odds ratio = 2.80, 95% CI: 1.19−6.58, *p* = 0.02).

**Table:** Table. OAB2105

	No or low‐risk alcohol use, *n* (row %)	Unhealthy alcohol use^a^, *n* (row %)
All	538 (75.2)	177 (24.8)
Male	92 (64.3)	51 (35.7)
Female	446 (78.0)	126 (22.0)
Aged 15−19	98 (94.2)	6 (5.8)
Aged 20−24	245 (75.6)	79 (24.4)
Aged 25−29	139 (68.4)	60 (31.6)
Ugandan	251 (62.2)	155 (38.2)
Kenyan	287 (92.9)	22 (7.1)

^a^ Alcohol Use Disorders Identification Test—Consumption (AUDIT‐C, prior 3 months) positive (≥3 for women, ≥4 for men) OR PEth≥50 ng/ml.

**Conclusions: **Unhealthy alcohol use was more common in males and increased with age, suggesting there may be a window for intervention. After adjusting for gender, age and country, unhealthy alcohol use was highly predictive of non‐suppression among AYAH receiving the intensive, multi‐level SEARCH Youth intervention, suggesting alcohol use may play a role in ongoing HIV transmission among AYAH.

### OAB2602 Efficacy and safety of bictegravir plus lenacapavir: 48‐week outcomes in virologically suppressed people with HIV‐1 on complex antiretroviral regimens at baseline

K. Mounzer^1^, J. Slim^2^, M. Ramgopal^3^, M. Hedgcock^4^, M. Bloch^5^, J. Santana^6^, I. Mendes^7^, X. Zhang^7^, P. Sklar^7^, J. M. Montezuma‐Rusca^7^, S. Segal‐Maurer ^8^

^1^Philadelphia FIGHT, Philadelphia, United States, ^2^New York Medical College, Valhalla, United States, ^3^Midway Immunology and Research Center, Fort Pierce, United States, ^4^Spectrum Health, Vancouver, Canada, ^5^Holdsworth House Medical Practice, Darlinghurst, Australia, ^6^University of Puerto Rico, San Juan, Puerto Rico, ^7^Gilead Sciences, Inc., Foster City, United States, ^8^New York‐Presbyterian Queens, Flushing, United States

**Background: **The combination of bictegravir (BIC), an integrase strand transfer inhibitor, and lenacapavir (LEN), a first‐in‐class capsid inhibitor, could consolidate treatment in virologically suppressed (VS) people with HIV‐1 (PWH) who are otherwise unable to take a single‐tablet regimen (STR). The 24‐week primary efficacy and safety outcomes for BIC + LEN in VS PWH on a complex regimen have been presented; here, we report longer‐term data through Week 48.

**Methods: **In this Phase 2, randomized, open‐label, multicentre study (ARTISTRY‐1, NCT05502341), PWH on a stable baseline regimen (SBR) (≥6 months prior to screening) were randomized 2:2:1 to receive once‐daily oral BIC 75 mg + LEN 25 mg, oral BIC 75 mg + LEN 50 mg or to continue SBR. The proportion of participants with HIV‐1 RNA <50 copies/ml (missing = excluded), change in CD4 cell count and treatment‐emergent adverse events (TEAEs) were assessed.

**Results: **At baseline (*N* = 128), 19% of participants were female, 31% were Black and 16% were Hispanic/Latinx; median (Q1, Q3) age was 60 (56, 65) years and participants were taking a median (range) of 3 (2–9) tablets per day. One hundred and twenty‐one participants completed the Week 48 visit (BIC 75 mg + LEN 25 mg: *N* = 49; BIC 75 mg + LEN 50 mg: *N* = 47; SBR: *N* = 25). At Week 48, all participants in the BIC 75 mg + LEN 50 mg group had HIV‐1 RNA <50 copies/ml; changes in CD4 counts were comparable among groups (Table). One participant on BIC 75 mg + LEN 50 mg had HIV‐1 RNA ≥50 copies/ml at Week 36 and resuppressed after switching treatment. BIC + LEN was well tolerated, with few TEAEs leading to premature treatment discontinuation (Table).

**Table:** Table. OAB2602

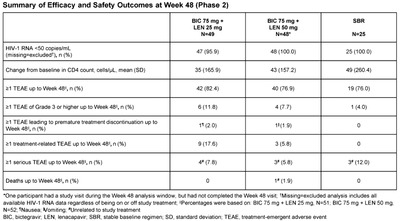

**Conclusions: **These findings support continued evaluation of the combination of BIC and LEN to simplify treatment in VS PWH who are receiving complex regimens. A BIC/LEN STR will be assessed in Phase 3.

### OAB2603 Phase 1 study of VH4524184 (VH‐184), a new third‐generation integrase strand transfer inhibitor with a unique resistance profile

L. Rogg ^1^, M. Underwood^1^, N. Hanan^2^, J. R. Castillo‐Mancilla^1^, L. Kahl^3^, F. Halliday^4^, J. L. Jeffrey^1^, S. Byrne^5^, T. Onodera^5^, J. Horton^6^, M. Lataillade^7^, M. Gartland^1^

^1^ViiV Healthcare, Durham, United States, ^2^GSK, Collegeville, United States, ^3^ViiV Healthcare, Brentford, United Kingdom, ^4^GSK, Brentford, United Kingdom, ^5^Shionogi & Co., LTD., Osaka, Japan, ^6^Parexel International, Durham, United States, ^7^ViiV Healthcare, Branford, United States

**Background: **Due to the widespread use, new integrase strand transfer inhibitors (INSTIs) that can overcome second‐generation INSTI‐related resistance may be needed. VH4524184 (VH‐184) is a third‐generation INSTI in development for HIV‐1 treatment. We present the pharmacokinetics, safety and in vitro resistance profile of VH‐184.

**Methods: **A double‐blind, randomized, placebo‐controlled, phase 1, first‐time‐in‐human (FTIH) study evaluated oral VH‐184 in healthy adults administered as single ascending doses (10−460 mg; part 1), multiple ascending doses (160−480 mg) for 14 days with concomitant midazolam (part 2), and as a single dose (100 mg) under fasted and fed conditions (part 3). Additionally, resistance to VH‐184 was evaluated in vitro against a panel of HIV‐1 clinical isolates from the phase 3 SAILING and DAWNING studies.

**Results: **In the FTIH study, 84 participants were included (placebo, *n* = 21; VH‐184, *n* = 63; 5 female, 34 Black, 28 Hispanic). Geometric mean VH‐184 plasma concentrations increased in a dose‐proportional manner after single doses of 10−300 mg, without further increase after 460‐mg single or 480‐mg multiple doses; geometric mean half‐life was ∼24 hours. Accumulation in exposures ranging from 1.3‐ to 1.9‐fold was observed after repeat VH‐184 dosing of 480 and 160 mg, respectively. Results suggested VH‐184 had minimal impact on the pharmacokinetics of CYP3A substrates and a moderate positive food effect. Generally, adverse events (AEs) were mild (*n* = 44 in 29 participants); none were serious. Laboratory AEs were asymptomatic and unrelated to VH‐184. VH‐184 demonstrated potent in vitro antiviral activity against dolutegravir‐selected INSTI‐resistant isolates (Figure).

**Figure**. OAB2603

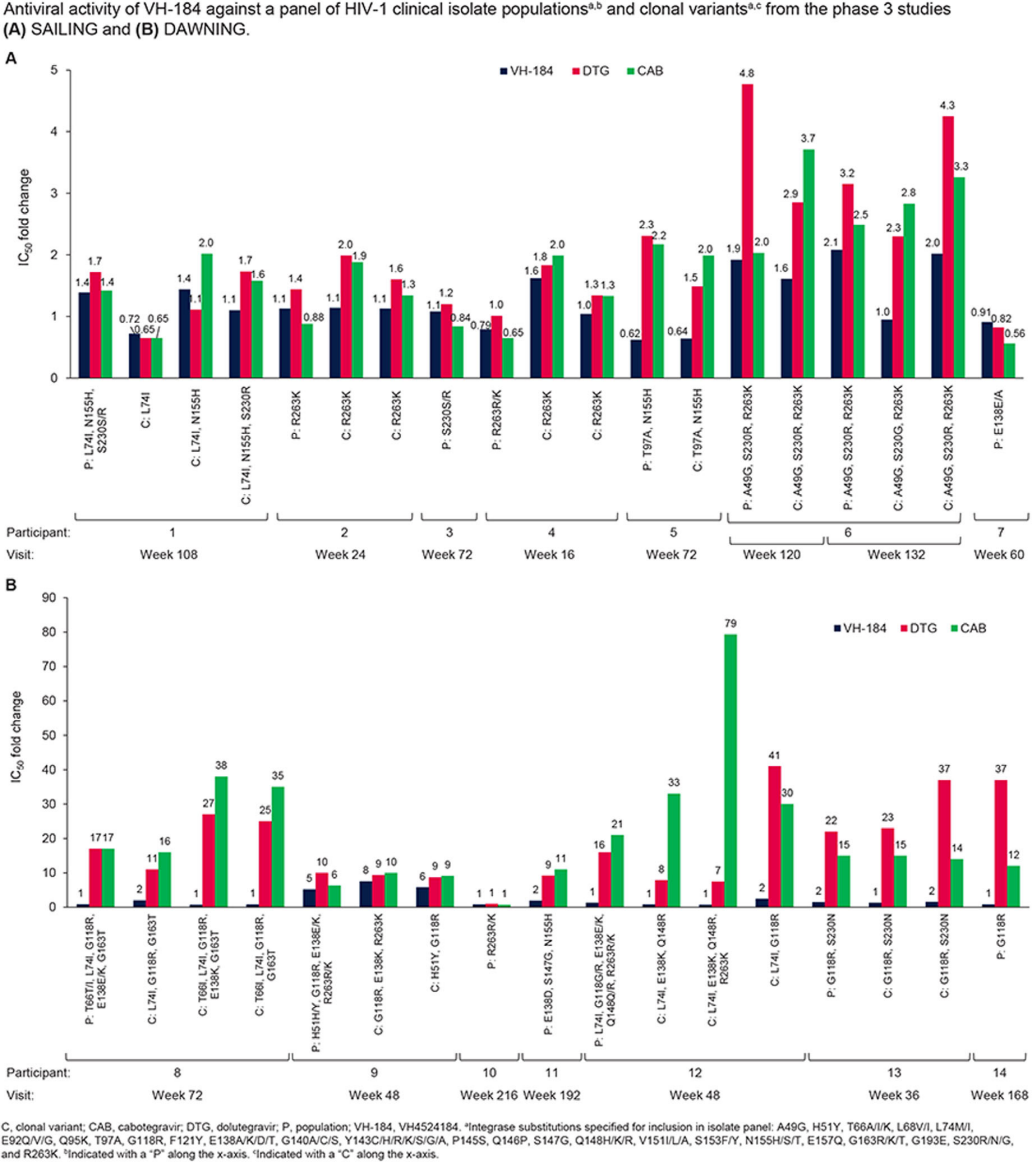

**Conclusions: **FTIH study results helped characterize VH‐184 pharmacokinetics and indicate that VH‐184 does not inhibit or induce CYP3A4 and has a moderate positive food effect. The in vitro resistance profile of VH‐184 is distinct from prior INSTIs, retaining antiviral activity against second‐generation INSTI‐resistant clinical isolates. Early data support the safety and further development of VH‐184 as a third‐generation INSTI for HIV‐1 treatment.

### OAB2604 Subcutaneous injections of cabotegravir + rilpivirine in virally suppressed adults with HIV‐1: a substudy of the Phase 3 FLAIR study

R. D'Amico ^1^, K. Han^2^, S. Min^1^, R. DeMoor^2^, M. St Clair^1^, S. L. Ford^3^, C. Harrington^1^, E. Bernal Morell^4^, J. Lombaard^5^, H. Crauwels^6^, I. Kolobova^1^, N. Patel^7^, N. Jamil^7^, R. Van Solingen‐Ristea^6^, C. Acuipil^1^, W. Spreen^1^

^1^ViiV Healthcare, Durham, United States, ^2^GSK, Collegeville, United States, ^3^GSK, Durham, United States, ^4^Sección de Enfermedades Infecciosas, Hospital General Universitario Reina Sofía, Murcia, Spain, ^5^Josha Research, Bloemfontein, South Africa, ^6^Janssen Research & Development, Beerse, Belgium, ^7^GSK, London, United Kingdom

**Background: **Cabotegravir+rilpivirine (CAB+RPV) administered via intramuscular gluteal (IM) injections is approved for maintaining HIV‐1 virologic suppression. Subcutaneous (SC) injection may enable self‐administration at home. We present outcomes from the Phase 3 FLAIR (NCT02938520) substudy evaluating SC anterior abdominal injections of CAB+RPV in participants with >3 years’ experience with IM injections.

**Methods: **FLAIR participants who consented for the substudy received IM injections during screening, 3 SC injections from Day 1 to Week (W) 8, and IM injections at W12. All injections were administered by study staff using the FLAIR study regimen and schedule (Q4W, CAB+RPV [400 mg/600 mg]). Outcomes assessed included pharmacokinetics, safety, tolerability, efficacy and patient reported outcomes (PROs).

**Results: **Ninety‐three participants enrolled into the substudy and received SC injections; 19% were female sex at birth, 23% were ≥50 years and 20% were Black. CAB and RPV plasma exposures were generally comparable between SC and IM injections, with 90% CIs of geometric liver stiffness measure (LSM) ratios all within 0.80–1.25 bioequivalence limits (Table). Pain, nodules and erythema were the most commonly reported SC‐related injection site reactions (ISRs), occurring with 48%, 34% and 26% of injections. Median ISR duration was 10 days; however, median induration and nodule durations were longer with SC versus IM administration (33 vs. 26 days and 39 vs. 9 days, respectively). Five (5%) participants withdrew due to an SC‐related ISR. At W12, 90% (*n* = 84/93) of participants maintained HIV‐1 RNA <50 copies/ml, and 2% (*n* = 2/93) had HIV‐1 RNA ≥50 copies/ml; no participants had confirmed virologic failure during the substudy. At W9, most participants preferred IM injections (59%; *n* = 50/85), most commonly citing less injection site swelling (58%; *n* = 29/50) and fewer nodules (58%); 34% (*n* = 29/85) preferred SC, most commonly citing convenience (86%; *n* = 25/29). Fifty‐nine percent (*n* = 51/87) of participants were “extremely” or “very” interested in self‐administration.

**Table:** Table. OAB2604: Geometric LSM ratios and 90% CIs for CAB and RPV PK parameters following SC (test) and gluteal (reference) administration by injection interval (paired data)

Analyte	Parameter	SC injection 1	SC injection 2	SC injection 3	Return to gluteal (assessed *post hoc*)
CAB	Cmax	0.942	0.911	0.910	1.003
		(0.897, 0.990)	(0.860, 0.965)	(0.857, 0.966)	(0.938, 1.074)
		(*n* = 89)	(*n* = 86)	(*n* = 83)	(*n* = 82)
	Ctau	0.948	0.935	0.934	0.999
		(0.901, 0.998)	(0.877, 0.995)	(0.872, 1.000)	(0.943, 1.059)
		(*n* = 63)	(*n* = 74)	(*n* = 70)	(*n* = 76)
RPV	Cmax	0.938	0.918	0.905	0.937
		(0.895, 0.982)	(0.876, 0.963)	(0.861, 0.952)	(0.891, 0.986)
		(*n* = 88)	(*n* = 85)	(*n* = 82)	(*n* = 80)
	Ctau	1.002	0.961	0.928	1.003
		(0.952, 1.056)	(0.921, 1.002)	(0.885, 0.973)	(0.955, 1.053)
		(*n* = 63)	(*n* = 73)	(*n* = 68)	(*n* = 74)

*Note*: Values displayed are geometric LSM ratios (90% CI) between each injection versus the IM gluteal injection immediately prior to the first SC injection. Return to gluteal: the IM gluteal injection immediately following the third (final) SC injection versus the IM gluteal injection immediately prior to the first SC injection.

Abbreviations: CAB, cabotegravir; CI, confidence interval; Cmax, plasma concentration approximately 1 week post injection; Ctau, plasma concentration at end of dosing interval; IM, intramuscular; LSM, least squares mean; PK, pharmacokinetics; RPV, rilpivirine; SC, subcutaneous.

**Conclusions: **Equivalent pharmacokinetics and similar efficacy were established for CAB+RPV at the SC administration site. Most participants favoured IM, but a majority were still interested in the option of self‐administration.

### OAB2605 PedMAb1 clinical trial: Safety assessment OF CAP256V2LS and VRC07‐523LS to prevent breastmilk HIV transmission in HIV‐1 exposed and negative neonates

G. Scarlatti ^1^, L. Naidoo^2^, M. Matlou^2^, T. Ramraj^2^, Y. Singh^2^, B. Daniels^2^, T. Chetty^2^, R. Dassaye^2^, N. Ngandu^2^, L. Galli^1^, M. Reddy^2^, Q. September^2^, N. Ngcobo^2^, T. Reddy^2^, T. Cafun‐Naidoo^2^, K. Woeber^2^, N. Jeenarain^2^, R. Imamdin^2^, K. Maharajh^2^, A. Ramjeth^2^, T. Bhengu^2^, L. Gama^3^, P. Van de Perre^4^, T. Tylleskar^5^, N. Nagot^4^, Y. Cazaboun^4^, J. P. Moles^4^, P. Moore^6^, S. Balla^6^, N. N. Mkhize^6^, P. Biswas^1^, S. Dispinseri^1^, A. Goga^7^, on behalf of the PedMAb1 clinical trial team

^1^IRCCS Ospedale San Raffaele srl, Viral Evolution and Transmission, Milan, Italy, ^2^SA‐MRC, Durban, South Africa, ^3^National Institute of Health, Vaccine Research Center, Bethesda, United States, ^4^University Montpellier/INSERM, Montpellier, France, ^5^University of Bergen, Bergen, Norway, ^6^Wits Health Consortium/NICD, Johannesburg, South Africa, ^7^SA‐MRC, Pretoria, South Africa

**Background: **Breastmilk transmission of HIV‐1 contributes to residual vertical HIV transmission. PedMAb1 aims to define the optimal doses, ideal combination and timing of subcutaneous (SC) administration of two broadly neutralizing antibodies, VRC07‐523LS and CAP256V2LS, to prevent vertical HIV transmission. Here, we first report on reactogenicity and safety of CAP256V2LS, for the first time in infants, and VRC07‐523LS.

**Methods: **Since study start on 1st September 2022, eight eligible HIV‐exposed uninfected infants in each study arm received 5, 10 or 20 mg/kg CAP256V2LS (arms 1−3) or 20 or 30 mg/kg VRC07‐523LS (arms 4 and 5), SC within 96 hours of birth. All infants were observed for 4 hours post‐dose, and followed up face‐to‐face at days 3, 14 and 28, then monthly until 6 months for safety and pharmacokinetic assessments. In a pictorial study, diary mothers documented reactogenicity and early adverse events (AEs). An internal study safety committee reviewed safety data 2 weekly. Here, we report on reactogenicity and AEs until 19 January 2024. The Division of AIDS Table, version 2.1. July 2017, was used to grade AEs.

**Results: **Reactogenicity grade 1 events observed at 4 hours or over the first 3 days post‐dose were recorded in 3/8 and 2/8 infants receiving CAP256V2LS at 10 and 20 mg/kg, respectively; and in 4/8 and 3/8 infants receiving VRC07‐523LS at 20 and 30 mg/kg, respectively. AEs were mostly common illnesses (except for low absolute neutrophils, a palatal cyst and an uncomplicated umbilical hernia). Ninety‐nine AEs were reported in 24 infants receiving CAP256V2LS, but only two (grade 1), one infant with raised AST and one with irritability in the 10 and 20 mg/kg arms, respectively, were related to study product. Sixty‐one AEs were reported in 16 infants receiving VRC07‐523LS, and only two (raised AST and ALT) grade 1 and 2, in one infant were related to the 20 mg/kg administration. All related AEs were asymptomatic and resolved within 2 weeks.

**Conclusions: **CAP256V2LS and VRC07‐523LS at the doses here administered SC to infants within max 96 hours of birth are safe and showed overall low reactogenicity. Reported product‐related AEs were rare and expected. Analysis of the pharmacokinetics of the study products is ongoing.

### OAB3402 Detailed modelling of viraemia exposure does not independently predict cardiovascular disease in people with HIV

O. Elvstam ^1^, on behalf of the RESPOND Study Group

^1^Lund University, Department of Translational Medicine, Malmö, Sweden

**Background: **People with HIV have increased risk of cardiovascular disease (CVD), and prolonged viraemia has been considered a CVD risk factor. Still, many previous studies have had insufficient data on potential confounders. We explored the association between viraemia and CVD after adjusting for established risk factors.

**Methods: **Adults from RESPOND (data from 2012 to 2021) without prior CVD were followed from the first date with complete data until the first of CVD (myocardial infarction, stroke or invasive cardiovascular procedures), loss to follow‐up or death. We fitted Cox models including the variables in the D:A:D score (age, gender, smoking, family history, diabetes, current abacavir, CD4 count, blood pressure, cholesterol, high‐density lipoprotein, stavudine, didanosine, indinavir, lopinavir and darunavir; all time‐updated). We analysed the associations between six measures of viraemia (detection limit, 200 copies/ml) and CVD after adjusting for the D:A:D variables. We compared model performance with and without viraemia with Harrell's C in five‐fold internal cross‐validation.

**Results: **Five hundred and forty‐seven events (39% myocardial infarctions and 31% strokes) were observed in 17,497 persons (median follow‐up, 6.8 years). Median age at inclusion was 45 years, 76% were male and median total viraemia‐copy‐years was 2.7 log_10_copy × years/ml. While several viraemia variables were associated with CVD in univariable analyses, there were no statistically significant associations when adjusting for the D:A:D variables, neither for measures of current or pre‐ART viral load, highest viraemia category during ART or cumulative viraemia. None of the viraemia variables improved prediction capacity (weighted mean, 0.70 for D:A:D variables without viraemia and 0.70 for all models combining D:A:D and viraemia variables).

**Conclusions: **In this large cohort, HIV viraemia was not associated with CVD after adjusting for established risk factors. Although we are unable to analyse the impact of viraemia before HIV diagnosis, our results provide evidence against a role for viral load in predicting CVD among people with HIV.

**Figure**. OAB3402

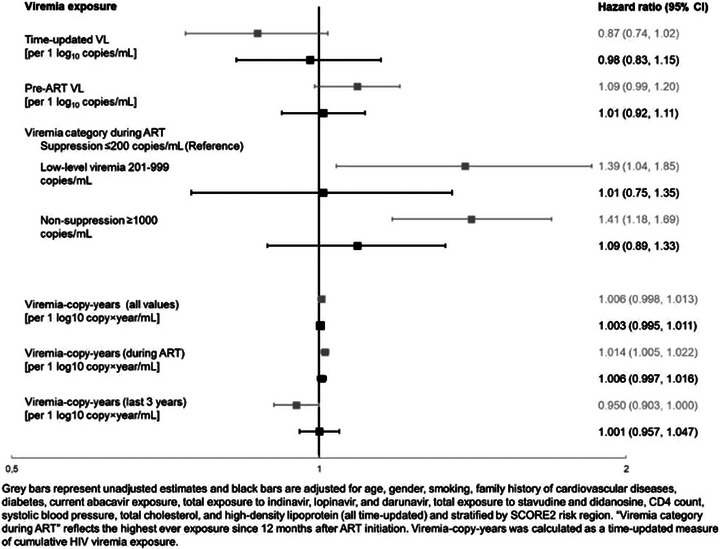

### OAB3403 Risk of major cardiovascular events with dolutegravir versus efavirenz‐based antiretroviral therapy: retrospective cohort analyses using routine, de‐identified data from South Africa

J. Dorward ^1,2^, X. Masombuka^3^, L. Lewis^2^, C. Pastellides^3^, J. van der Molen^2^, K. Asare^2^, C. Bottomley^4^, D. Jacobs^3^, S. Collie^3^, N. Garrett^2,5^

^1^University of Oxford, Nuffield Department of Primary Care Health Sciences, Oxford, United Kingdom, ^2^Centre for the AIDS Programme of Research in South Africa (CAPRISA), Durban, South Africa, ^3^Discovery Health, Johannesburg, South Africa, ^4^London School of Hygiene & Tropical Medicine, London, United Kingdom, ^5^University of KwaZulu‐Natal, Discipline of Public Health Medicine, School of Nursing and Public Health, Durban, South Africa

**Background: **Integrase inhibitors such as dolutegravir may be associated with increased cardiovascular disease (CVD) but there are few data from low‐ and middle‐income countries (LMICs), where dolutegravir has largely replaced efavirenz.

**Methods: **We used de‐identified, routinely collected data from a South African managed healthcare organization to conduct a retrospective cohort study among people with HIV (PWH) ≥18 years old, without known CVD, and initiating tenofovir disoproxil fumarate, emtricitabine and efavirenz (TEE), or tenofovir disoproxil fumarate, lamivudine and dolutegravir (TLD). We used a multivariable Cox regression to evaluate associations between TLD and the primary outcome of CVD admissions/deaths, adjusted for baseline characteristics. In a sensitivity, “as‐treated” analysis, we censored people if they changed their ART regimen during follow‐up.

**Results: **Between April 2020 and December 2022, 5107 PWH initiated TLD (*n* = 2204) and TEE (*n* = 2903). Median (IQR) age was 37 (32−44) years, 56.8% were female and 9.8% had a risk factor for CVD at baseline (Table). Median follow‐up was 535 days (IQR 308−582). There were 33 CVD events after a median 487 (IQR 245−844) days. Twenty‐five occurred in the TEE group (observed rate 4.91/1000 person‐years, 95% CI 3.17−7.24) and eight in the TLD group (2.49/1000 person‐years, 95%CI 1.08−4.91). In the multivariable Cox regression model, there was no evidence of a higher risk of CVD events with TLD versus TEE (hazard ratio 0.49, 95% CI 0.22−1.09, *p* = 0.080). In the TLD group, 3.4% PWH changed their ART regimen after a median 268 days, and in the TEE group, 13.1% changed ART after median 314 days, and were censored in the “as‐treated” analysis with similar results to the main model.

**Table:** Table. OAB3403

Variable	Levels	TEE *n*, %	TLD *n*, %	Total
Age (years)	Median (IQR)	37.0 (32.0−44.0)	38.0 (32.0−44.0)	37.0 (32.0−44.0)
Gender	Female	1733 (59.9)	1170 (52.8)	2903 (56.8)
TB at ART initiation	Yes	86 (3.0)	74 (3.3)	160 (3.1)
Hypercholesterolaemia	Yes	45 (1.6)	40 (1.8)	85 (1.7)
Hypertension	Yes	231 (8.0)	208 (9.4)	439 (8.6)
Diabetes mellitus	Yes	80 (2.8)	44 (2.0)	124 (2.4)
CVD risk factor	Yes	269 (9.3)	234 (10.6)	503 (9.8)
Statin use	Yes	85 (2.9)	79 (3.6)	164 (3.2)

**Conclusions: **We found no evidence of increased risk of CVD in people who initiated TLD versus TLE in this large South African cohort with medium term follow‐up, supporting the decision to replace TEE with TLD at ART initiation in South Africa.

### OAB3404 Dolutegravir‐containing antiretroviral therapy and incident hypertension: findings from a prospective cohort in Kenya, Nigeria, Tanzania and Uganda

M. Romo ^1,2^, S. Frndak^1,2^, N. Dear^1,2^, H. Kibuuka^3^, J. Owuoth^4,5^, V. Sing'oei^4,5^, J. Maswai^6,7^, E. Bahemana^8,9^, V. Anyebe^10,11^, Z. Parker^2,12^, J. S. Cavanaugh^1^, N. Shah^1^, J. Ake^1^, T. Crowell^1,2^, African Cohort Study (AFRICOS) Group

^1^U.S. Military HIV Research Program, CIDR, Walter Reed Army Institute of Research, Silver Spring, United States, ^2^Henry M. Jackson Foundation for the Advancement of Military Medicine, Inc., Bethesda, United States, ^3^Makerere University Walter Reed Project, Kampala, Uganda, ^4^U.S. Military HIV Research Program, Walter Reed Army Institute of Research, Walter Reed Army Institute of Research Africa, Kisumu, Kenya, ^5^HJF Medical Research International, Kisumu, Kenya, ^6^U.S. Military HIV Research Program, Walter Reed Army Institute of Research, Walter Reed Army Institute of Research Africa, Kericho, Kenya, ^7^HJF Medical Research International, Kericho, Kenya, ^8^U.S. Military HIV Research Program, Walter Reed Army Institute of Research, Walter Reed Army Institute of Research Africa, Mbeya, the United Republic of Tanzania, ^9^HJF Medical Research International, Mbeya, the United Republic of Tanzania, ^10^U.S. Military HIV Research Program, Walter Reed Army Institute of Research, Walter Reed Army Institute of Research Africa, Abuja, Nigeria, ^11^HJF Medical Research International, Abuja, Nigeria, ^12^U.S. Military HIV Research Program, Walter Reed Army Institute of Research, Walter Reed Army Institute of Research Africa, Lagos, Nigeria

**Background: **Randomized trials in African countries have suggested that people with HIV (PWH) initiating antiretroviral therapy (ART) with dolutegravir may have increased risk of hypertension compared with efavirenz. This risk needs to be assessed in real‐world populations.

**Methods: **The ongoing African Cohort Study enrols PWH and people without HIV (PWoH) aged ≥15 years in care at 12 PEPFAR‐supported facilities in Kenya, Nigeria, Tanzania and Uganda. For these analyses, observation time began at ART initiation or cohort enrolment (whichever later) for PWH and at cohort enrolment for PWoH. We defined hypertension as blood pressure ≥140/90 mmHg at ≥2 consecutive 6‐monthly visits or receipt of any anti‐hypertensive medication. We excluded participants who met this definition prior to the start of observation time (i.e. prevalent hypertension) and study visits with pregnancy. We used Cox proportional hazards models to examine associations between time‐varying ART anchor drug (or PWoH) and incident hypertension, changing reference groups to allow for all comparisons with dolutegravir. We ran an unadjusted model, a model adjusted for socio‐demographic characteristics only and a model additionally adjusted for time‐varying body mass index (BMI).

**Results: **Between 01/2013 and 08/2023, 2935 participants meeting inclusion criteria for these analyses were enrolled and followed for a median of 5.4 years; 2477 (84%) were PWH, 1691 (58%) were female and mean (SD) age was 36 (12) years. During follow‐up, 423 (14%) participants had incident hypertension. In unadjusted and adjusted models (Figure), dolutegravir was not significantly associated with incident hypertension compared with efavirenz, protease inhibitors and PWoH. Dolutegravir was associated with significantly reduced hazards of incident hypertension compared with nevirapine (adjusted hazard ratio 0.45, 95% CI: 0.32–0.63). Adjusting for time‐varying BMI did not have a major impact on comparisons.

**Figure**. OAB3404

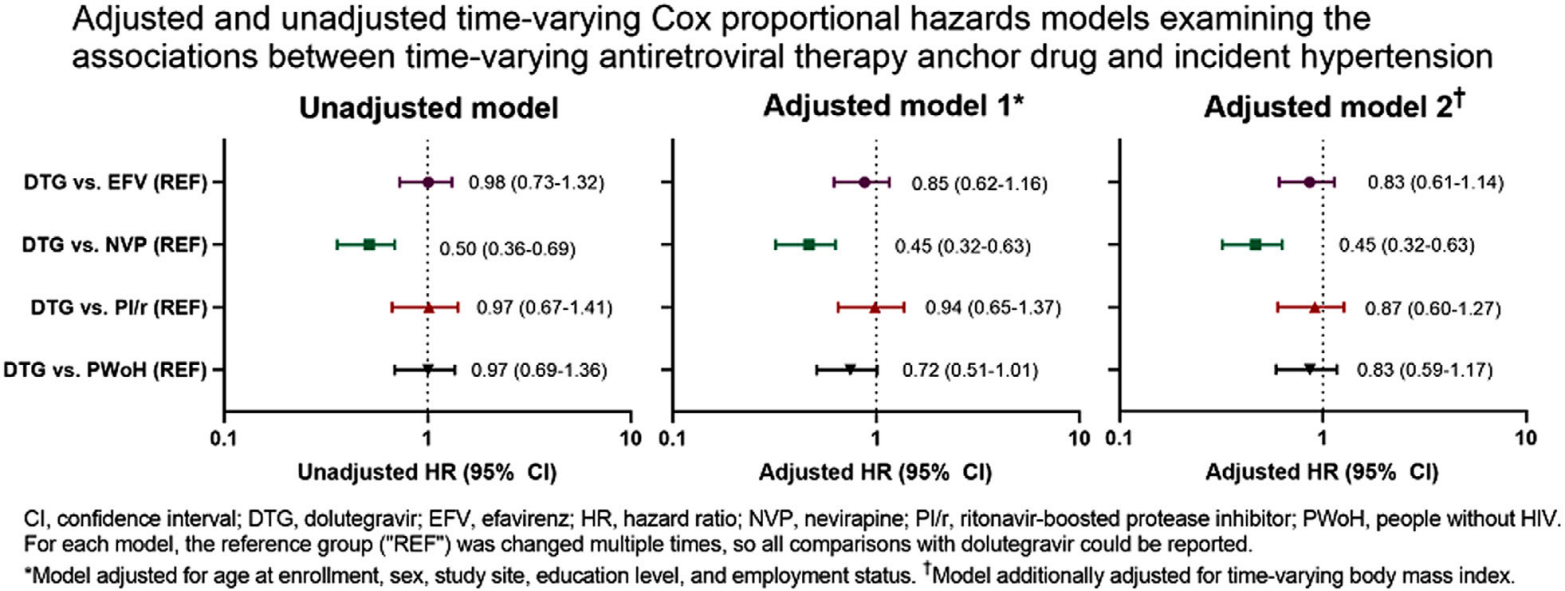

**Conclusions: **Dolutegravir was not associated with any significant deleterious impact on incident hypertension compared with other ART anchor drugs.

### OAB3405 Integrating non‐communicable disease screening and treatment in antiretroviral clinics: insights from Malawi

S. Kawonga Dunga ^1^, A. Yemane Berhan^2^, L. Kalitera^1^, S. Fatch^1^, A. Maida^3^, T. Maphosa^1^

^1^Elizabeth Glaser Pediatric Aids Foundation, Lilongwe, Malawi, ^2^Elizabeth Glaser Pediatric AIDS Foundation, Washington, DC, United States, ^3^U.S. Centers for Disease Control, Division of Global HIV & TB, Lilongwe, Malawi

**Background: **Increased access to antiretroviral therapy (ART) has improved the life expectancy of people living with HIV (PLHIV), resulting in an ageing cohort at high risk of developing non‐communicable diseases (NCDs). Integrating NCD care within HIV clinics is pivotal for comprehensive healthcare. We share the outcomes of integrating NCD screening with ART services in Malawi.

**Description: **The initiative by the Elizabeth Glaser Pediatric AIDS Foundation involved using lay health workers (LHWs) to implement hypertension (HTN) and diabetes mellitus (DM) screening among PLHIV aged > 30 and > 40 years, respectively, across 46 health facilities. We analysed routinely collected data from screening registers for all ART clients screened for HTN or DM between January and September 2023. We used descriptive analysis and logistic regression based on two outcome variables: HTN, defined as at least two blood pressure readings of > 140/90 mmHg, and DM, defined as a random blood sugar level of ≥ 200 mg/dl or fasting blood sugar level of ≥ 163 mg/dl.

**Lessons learned: **A total of 106,880 PLHIV on ART were eligible for HTN screening, while 60,572 were eligible for DM screening. Of those eligible, 23,841 (22%) underwent HTN screening, and 9835 (16%) DM screening. The screening cohorts predominantly consisted of women (67% and 63% within the HTN and DM cohorts, respectively). The prevalence of HTN and DM was 3815 (16%) and 197 (2%), respectively. The prevalence of HTN was higher in males (16.8%) than females (15.5%) (*p* = 0.013). Individuals aged > 60 years were more likely to have HTN (adjusted odds ratio [AOR] = 2.84, 95% CI: 2.50−3.21) or DM (AOR = 2.04, 95% CI: 1.43−2.9, *p* < 0.001) than those aged 40–60 years. A high percentage diagnosed with HTN (91.7%) and DM (89%) were treated within the HIV clinic. Severe HTN requiring hospitalization was noted in nine PLHIV.

**Conclusions/Next steps: **The findings show the significance of addressing the healthcare needs of patients on ART, particularly in integrating NCD care into HIV services. Enhancing NCD screening rates and optimizing treatment within ART clinics can better manage HIV comorbidities.

### OAB3602 Time is of the essence: clinical frailty among a cohort of indigenous peoples ageing with HIV in Ontario, Canada

N. Bauer ^1,2^, K. O'Brien^2^, L. Light^2^, L. Sirisegaram^3^, R. Jackson^4^, M. Young^5^, A. Kroch^2^, S. Hillier^6^

^1^University of Toronto, Temerty Faculty of Medicine, Toronto, Canada, ^2^Ontario HIV Treatment Network (OHTN), Toronto, Canada, ^3^University Health Network and Sinai Health Systems, Division of General Internal Medicine and Geriatric Medicine, Department of Medicine, Toronto, Canada, ^4^McMaster University, Department of Health & Aging and the School of Social Work, Hamilton, Canada, ^5^Ontario Aboriginal HIV/AIDS Strategy (OAHAS), Barrie, Canada, ^6^York University, School of Health Policy & Management, Toronto, Canada

**Background: **As the cohort of people living with HIV (PLHIV) grows older on average, geriatric and HIV care must co‐evolve. Within this cohort, Indigenous Peoples remain overrepresented. The ageing experience for Indigenous older adults living with HIV (IOALWH) involves the intersecting effects of systemic colonial oppression, intergenerational trauma, continued racist violence, service inaccessibility and socio‐economic disadvantage. These factors have contributed to increasing health disparities within Indigenous communities, translating to higher co‐morbidity burden and worse health outcomes. Owing to both their HIV status and Indigenous identity, IOALWH may face disproportionate burdens while ageing. This study aims to characterize clinical frailty in a cohort of IOALWH.

**Methods: **The OHTN Cohort Study (OCS) is an open longitudinal cohort of PLHIV at 15 clinical sites in Ontario, Canada, with currently over 5000 people under active follow‐up. The study includes data abstraction from clinical records, laboratory reports and an annually administered questionnaire. We assessed clinical frailty using the modified frailty index (mFI), approximated with aggregations of ICD‐10 codes from diagnostic records. Presentation of a frailty‐related condition contributes to a frailty score. A score of 0 represents no clinical frailty, 1−2 is pre‐frailty and ≥3 is clinical frailty.

**Results: **Data from 6582 participants (*n* = 330 Indigenous) and diagnostic reports from 1940 to 2018 were included. IOALWH faced greater rates of pre‐frailty (49.7% vs. 41.1%) and clinical frailty (8.8% vs. 5.8%). IOALWH acquired all clinical indicators at earlier median ages, including HIV (31 vs. 34), AIDS (36 vs. 40), pre‐frailty (40 vs. 44) and clinical frailty (51 vs. 56). Certain frailty indicators were overrepresented among IOALWH compared to non‐Indigenous OALWH, including impaired sensorium (+21.1% greater prevalence), chronic inflammatory lung disease (COPD)/pneumonia (+7.6%), diabetes (+2.3%) and non‐independent functional status (+2.3%). In a multivariate logistic model, intravenous drug use (IVDU; OR 1.97, *p*<.0001), AIDS (OR 1.57, *p*<.0001) and Indigenous identity (OR 1.57, *p*<.0001) were found to be independent predictors of pre‐frailty and clinical frailty.

**Conclusions: **IOALWH face disproportionate burdens of ageing‐related co‐morbidities and acquire clinical frailty at earlier ages compared to non‐Indigenous OALWH, independent of IVDU and AIDS. Healthcare delivery must address underlying inequities by co‐evolving HIV and geriatric care to respond to earlier and more complex care needs.

### OAB3603 Association of PNPLA3 rs738409 genotype with hepatic steatosis among non‐obese people living with HIV

T. Apornpong^1^, N. Chuaypen^2^, H. M. S. Lwin^1^, A. Hiransuthikul^1,3^, S. Gatechompol^1^, S. Ubolyam^1^, S. Kerr^1,4,5^, P. Tangkijvanich^2^, A. Avihingsanon^1^, W. M. Han ^1,5^, HIVNAT 006

^1^HIV‐NAT, Thai Red Cross AIDS Research Centre, Bangkok, Thailand, ^2^Center of Excellence in Hepatitis and Liver Cancer, Chulalongkorn University, Department of Biochemistry, Faculty of Medicine, Bangkok, Thailand, ^3^Chulalongkorn University, Department of Preventive and Social Medicine, Faculty of Medicine, Bangkok, Thailand, ^4^Biostatistics Excellence Centre/Chulalongkorn University, Faculty of Medicine, Bangkok, Thailand, ^5^The Kirby Institute, UNSW, Sydney, Australia

**Background: **In addition to the well‐established association of obesity, there is a growing recognition of genetic influences on susceptibility to liver diseases. We investigated genetic determinants of steatosis liver disease (SLD) among Thai people with HIV (PWH).

**Methods: **A cross‐sectional study was conducted at the HIV Netherlands Australia Thailand (HIV‐NAT), Bangkok, Thailand. PWH aged ≥18 years without excessive alcohol consumption who underwent controlled attenuation parameter (CAP) between July 2013 and June 2023 and had tested with single nucleotide polymorphism (SNPs) including PNPLA3 rs738409 and HSD178B rs6834314 were analysed. SLD was defined as CAP ≥248 dB/m. Multivariable logistic regression investigated associations between SNPs and SLD, and interactions between genotypes and obesity (BMI>25 kg/mm^2^).

**Results: **Of 764 PWH (35% female, median age 45 [IQR 36−52] years) analysed, SLD was observed in 270 (35.3%) participants and 136 (50%) were not obese. The median duration of ART and median CD4 count was 11 (5−18) years and 581 (422−753) cells/mm^3^, respectively. Seventy‐four (35%) were on integrase strand transfer inhibitors. Participants with SLD had higher BMI (24.9 [22.8−28.1] vs. 22.1 [IQR 20.3−23.9] kg/m^2^) and a higher proportion diabetes mellitus (25% vs. 16%), hypertension (83% vs. 60%), dyslipidaemia (88% vs. 76%) and liver fibrosis (LSM≥7.5 kPa, 22% vs. 12%), compared to those without SLD. Overall, PNPLA3 rs738409 CC, CG and GG genotypes were present in 50%, 39% and 11%, and HSD178B rs6834314 AA, AG and GG genotypes in 36%, 51% and 13%, respectively. In the multivariable model, the male sex (aOR = 1.98, 95% CI 1.23−3.18, *p* = 0.005), higher BMI (aOR = 1.32, 95% CI 1.24−1.41, *p*<0.001) and PNPLA3 rs738409 G allele (aOR = 1.49, 95% CI 1.003−2.22, *p* = 0.049) were associated with an increased risk of SLD. There was a significant interaction between PNPLA3 rs738409 genotype and BMI (*p* = 0.014). After subgroup analysis using BMI, PNPLA3 rs738409 G allele was significantly associated among non‐obese individuals (aOR = 1.79, 95% CI 1.18−2.72, *p* = 0.006).

**Conclusions: **Nearly half of the participants with SLD in our cohort were not obese. PNPLA3 rs738409 genotype was associated with SLD among non‐obese Thai individuals. This underscores that genetic factors can influence liver disease susceptibility in PWH, independent of established risk factors.

### OAB3604 Additional time post–integrase inhibitor to protease inhibitor switch shows trend to weight loss: DEFINE 48‐week results

D. Anderson ^1^, M. Ramgopal^2^, D. Hagins^3^, J. Lee^1^, R. B. Simonson^1^, T.‐H. Hsu^1^, P. Xu^4^, N. Ahmad^1^, W. R. Short^5^

^1^Johnson & Johnson, Infectious Diseases & Vaccines, Titusville, United States, ^2^Midway Immunology and Research Center, Fort Pierce, United States, ^3^Chatham CARE Center, Savannah, United States, ^4^Johnson & Johnson, Janssen Research & Development, Titusville, United States, ^5^Perelman School of Medicine, University of Pennsylvania, Philadelphia, United States

**Background: **Integrase inhibitor (INI)–based antiretroviral (ARV) therapies are associated with greater weight gain than non‐nucleoside reverse transcriptase inhibitor– or boosted protease inhibitor (PI)–based regimens. The primary analysis of DEFINE found no significant difference in percent body weight change from baseline to Week 24 when switching to darunavir/cobicistat/emtricitabine/tenofovir alafenamide (D/C/F/TAF) versus continuing INI+TAF/emtricitabine (FTC). Longer‐term weight/metabolic data through Week 48 post–ARV switch are reported here.

**Methods: **DEFINE (ClinicalTrials.gov: NCT04442737) is a randomized (1:1), prospective, 48‐week, active‐controlled, open‐label, multicentre phase 4 study evaluating a switch to D/C/F/TAF (immediate switch [IS]) versus continuance of INI+TAF/FTC (for Weeks 0−24, then delayed switch [DS] to D/C/F/TAF for Weeks 24−48) in virologically suppressed adults with HIV‐1 acquisition and ≥10% weight gain within the preceding 36‐month period on the INI‐based regimen. Percent weight change from baseline was analysed in the intent‐to‐treat (ITT) population. Outcomes are reported in the IS arm (with no comparator).

**Results: **One hundred and three adults were randomized (IS arm: *n* = 53; DS arm: *n* = 50); 61% were Black/African American and 30% were female. Versus 24 weeks, weight loss and DEXA changes were observed at Week 48 (median [IQR] percent change, −0.84% [−4.10, 1.69]; Table). Participants were increasingly likely to experience any weight loss (63.0% vs. 44.9%) and achieve ≥3% weight loss (30.4% vs. 14.3%) at Week 48 versus 24, respectively. With extended time post‐switch, minor improvements in BMI (57.1% with BMI ≥30 kg/m^2^ at Week 24 vs. 50.0% at Week 48) and waist circumference were also seen. D/C/F/TAF was safe and well‐tolerated, with high levels of virologic suppression.

**Table:** Table. OAB3604

	Week 24	Week 48
**Weight change**, %, median (interquartile range; [IQR])	0.52 (−1.80, 2.91)	−0.84 (−4.10, 1.69)
	[*n* = 49]	[*n* = 46]
**Experienced ≥3% weight loss**, *n* (%)	7 (14.3)	14 (30.4)
	[*n* = 49]	[*n* = 46]
**Weight change, %, Black/African American subgroup**, median (IQR)	−0.15 (−2.39, 2.91)	−1.11 (−5.60, 1.70)
	[*n* = 30]	[*n* = 28]
**Weight change, %, female subgroup**, median (IQR)	0.73 (−2.18, 3.49)	−1.75 (−4.05, 1.70)
	[*n* = 14]	[*n* = 12]
**Weight change, %, Black/African American female subgroup**, median (IQR)	0.03 (−2.29, 3.32)	−2.81 (−5.19, 2.17)
	[*n* = 12]	[*n* = 11]
**Weight change, %, baseline body mass index [BMI] ≥30 kg/m^2^ subgroup**, median (IQR)	−0.34 (−1.77, 2.43)	−1.07 (−3.55, 1.80)
	[*n* = 26]	[*n* = 24]
**Weight change, %, baseline BMI ≥40 kg/m^2^ subgroup**, median (IQR)	−0.58 (−2.39, 1.38)	−2.81 (−2.91, 0.96)
	[*n* = 11]	[*n* = 9]

**Conclusions: **The trajectory of weight change after INI to PI switch appeared different in the first 24 weeks post‐switch (no weight loss observed) versus Weeks 24−48, where a trend to weight loss emerged. Secondary metabolic endpoints remained stable or paralleled weight loss. Longer time post–ARV switch suggests medication management may be an important component to address this issue.

### OAB3605 A pilot study assessing changes in cerebral function parameters in persons with insomnia switching integrase inhibitors

M. Henderson ^1^, K. Alford^2^, N. Doyle^1^, A. Busza^3^, S. Vundavalli^4^, A. Winston^1^, J. Vera^2^

^1^Imperial College London, Department of Infectious Disease, London, United Kingdom, ^2^Brighton and Sussex Medical School, Department of Global Health and Infection, Brighton, United Kingdom, ^3^Imperial College London, Department of Brain Sciences, London, United Kingdom, ^4^Brighton and Sussex University Hospitals NHS Trust, Department of Imaging and Nuclear Medicine, Brighton, United Kingdom

**Background: **Sleep disturbances are frequently reported in persons with HIV and have been associated with the use of integrase strand transfer inhibitors (INSTIs). This exploratory study assessed changes in cerebral function parameters in individuals with insomnia switching INSTIs.

**Methods: **Individuals with an insomnia severity index (ISI) above 8 and virologically suppressed on a dolutegravir‐containing ART regimen (DTG‐ART) were randomized 1:1 to either continue DTG‐ART or switch to tenofovir‐alafenamide/emtricitabine/bictegravir (BIC‐ART) for 120 days. Cerebral function parameters were measured at baseline and day 120 and included PROs assessing sleep (ISI, Epworth Sleepiness Scale [ESS]) and quality of life (QoL) (Short Form 36‐Physical Function [SF36‐PF]), resting‐state functional cerebral MRI (RS fMRI) examining functional connectivity (FC) networks previously associated with DTG use and plasma soluble inflammatory biomarkers (sCD14, IL‐6 and IP‐10). Between group analyses of PRO change scores and the impact of covariates were analysed using Mann‐Whitney U and linear regression modelling. RS fMRI were examined by independent component analysis using Matlab's CONN toolbox.

**Results: **Of 19 individuals (12 DTG‐ART, 7 BIC‐ART), median age was 55 years (range 28−83), all were male and 17 of White ethnicity. Over 120 days, improvements in sleep and QoL in those randomized to BIC‐ART versus DTG‐ART were observed. Median change in ISI score −9 (−14 to −2) versus −1 (−10 to −4), *p* = 0.0247, ESS −3.0 (−6 to −1) versus 2 (−3 to 6), *p* = 0.005 and SF36‐PF −5 (−40 to 5) versus 0 (−5 to 15), *p* = 0.025, respectively. BIC‐ART was also associated with increased FC in the default mode network (*p*<0.005), most pronounced in the middle temporal and superior frontal gyri. No significant changes in soluble biomarkers were observed.

**Conclusions: **In this exploratory study, individuals with insomnia who switched to BIC‐ART had improvements in self‐reported sleep, QoL and RS networks associated with sleep, when compared to those continued on DTG‐ART.

**Figure**. OAB3605

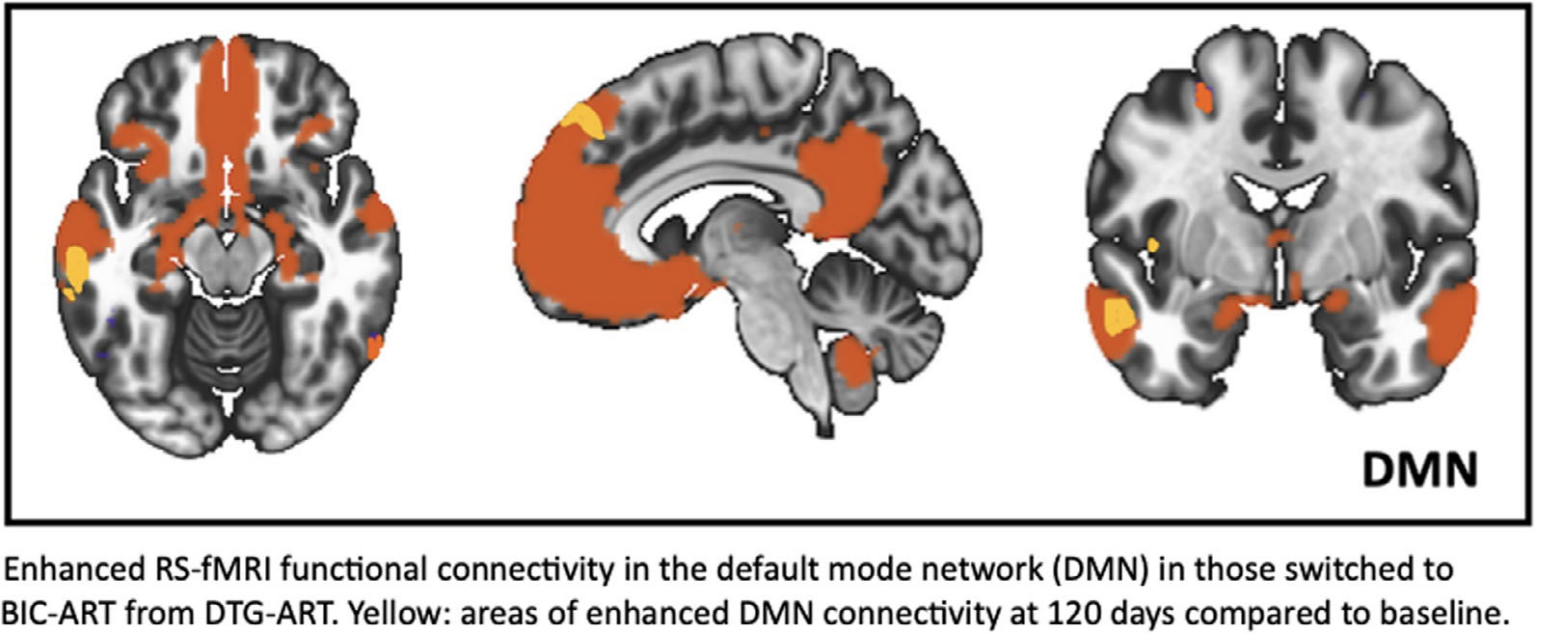

### OAB3802 Analysis of dolutegravir scaleup and HIV‐1 treatment outcomes in Uganda from 2021 to 2023

K. Huang ^1^, P. Solis Reyes^1^, A. Ayitewala^2^, F. Kyeyune^2^, I. Nankia^2^, E. Arts^1^

^1^University of Western Ontario, Microbiology and Immunology, London, Canada, ^2^Joint Clinical Research Centre, Center for AIDS Research Laboratories, Kampala, Uganda

**Background: **As the rapid DTG scale‐up in sub‐Saharan Africa nears completion, assessment on the impact of this major transition is necessary. Critically, little is known about the suppression rates of DTG‐based regimens in sub‐Saharan Africa following mass implementation. The Joint Clinical Research Centre (JCRC) is headquartered in Kampala, Uganda, and houses one of the largest HIV clinics and research facilities in Africa. Together with its regional facilities, the JCRC provides care to over one million HIV clients yearly. Here, we report on the clinical outcomes of DTG and non‐nucleoside reverse transcriptase inhibitor (NNRTI)‐based regimens seen in clients receiving care at the JCRC between January 2021 and September 2023.

**Methods: **The treatment regimen and outcome (suppression or failure) was recorded for clients treated at the JCRC in Kampala, Uganda, between 2021 and 2023. Frequency and suppression/failure rates of each regimen were determined.

**Results: **The clinic provided care for 1,258,368 clients (95.2% viral suppression) in January−September 2023 (2023n), up from 975,622 clients in 2021 (95.9% suppression). The use of DTG‐based regimens increased from 876,872 (89.9%) in 2021 to 1,250,188 (99.4%) in 2023n. This corresponded with an increase in DTG failure rate from 3.5% (30,944 clients) in 2021 to 4.8% (59,677 clients) in 2023n. The two most common DTG‐based cocktails were TDF/3TC/DTG (TLD) and ABC/3TC/DTG (1,195,722 and 41,761 clients in 2023n, respectively). Strikingly, virologic failure was 3.4‐fold higher for ABC/3TC/DTG compared to TLD (0.14% vs. 0.04%) in 2023n. The most common DTG‐based salvage therapy was DRV/RTV/TDF/3TC/DTG (417 clients in 2023n), with a virologic suppression of 78.9% (329/417 clients) in 2023n. The highest rate of suppression among DTG‐based salvage therapies (>10 clients) was observed for DRV/RTV/DTG/ETV (28/34 clients, 82.4%) in 2023n. NNRTI‐based regimen use decreased from 98,746 clients in 2021 to <10,000 clients in 2023n. Average virologic failure on an NNRTI‐based regimen was 8.9% from 2021 to 2023.

**Conclusions: **Thus, in one of the largest reports on the clinical outcomes of the DTG scale‐up to date, our findings support the continued widespread use of TLD in sub‐Saharan Africa to achieve UNAIDS’ 95% suppression target. However, as a consequence of observed rates of virologic failure >5%, we emphasize continued caution in the use of ABC/3TC/DTG and NNRTI‐based regimens.

### OAB3803 Viral suppression, viral failure and safety outcomes in children and adolescents on dolutegravir in Europe and Thailand

K. Scott ^1^, S. Crichton^1^, J. O'Rourke^1^, E. Chappell^1^, L. Ene^2^, L. Galli^3^, T. Goetghebuer^4^, C. Henegar^5^, T. U. Hoffmann^6^, C. Koenigs^7^, M. Marczyńska^8^, L. Naver^9,10^, A. Noguera‐Julian^11^, P. Paioni^12^, J. T. Ramos^13,14,15^, W. N. Songtaweesin^16^, V. Spoulou^17^, N. Tantawarak^18^, V. Vannappagari^5^, A. Volokha^19^, A. Turkova^1^, A. Judd^1^, I. J. Collins^1^

^1^MRC Clinical Trials at University College London, London, United Kingdom, ^2^“Dr. Victor Babes” Hospital for Infectious and Tropical Diseases, Bucharest, Romania, ^3^Infectious Disease Unit, Meyer Children's Hospital, IRCCS, Department of Health Sciences, University of Florence, Florence, Italy, ^4^Hospital St Pierre Cohort, Brussels, Belgium, ^5^ViiV Healthcare, Epidemiology and Real World Evidence, Durham, United States, ^6^Hvidovre University Hospital, Department of Pediatrics, Copenhagen, Denmark, ^7^Goethe University, University Hospital Frankfurt, Department of Paediatrics and Adolescent Medicine, Frankfurt, Germany, ^8^Medical University of Warsaw, Department of Children's Infectious Diseases, Warsaw, Poland, ^9^Karolinska University Hospital, Department of Pediatrics, Stockholm, Sweden, ^10^Karolinska Institutet, Department of Clinical Science, Intervention and Technology (CLINTEC), Stockholm, Sweden, ^11^Hospital Sant Joan de Deu, Universitat de Barcelona, Servei de Malalties Infeccioses, Barcelona, Spain, ^12^University Children's Hospital Zurich, Division of Infectious Diseases and Hospital Epidemiology, Zurich, Switzerland, ^13^Hospital 12 de Octubre Instituto de Investigación Sanitaria (i+12), Madrid, Spain, ^14^CIBERINFEC, ISCIII, Madrid, Spain, ^15^Universidad Complutense, Madrid, Spain, ^16^Chulalongkorn University, Department of Pediatrics, and School of Global Health, Faculty of Medicine, Bangkok, Thailand, ^17^“Agia Sofia” Children's Hospital, First Dept of Paediatrics, Athens, Greece, ^18^Khon Kaen University, Khon Kaen, Thailand, ^19^Shupyk National Healthcare, University of Ukraine, Kyiv, Ukraine

**Background: **There are limited data on DTG safety and effectiveness in children and adolescents living with HIV (CALWHIV) in routine‐care settings.

**Methods: **Data on CALWHIV aged <18 years at DTG start were pooled from 15 cohorts in the European Pregnancy and Paediatric Infections Cohort Collaboration. Effectiveness outcomes were proportion virally suppressed <50 copies/ml at 24/48/96/144/192 (±12) weeks after DTG start; and cumulative incidence of viral failure (≥2 viral load [VL] ≥400 c/ml or 1 VL≥400 c/mlfollowed by DTG discontinuation), overall and by age, ART/VL status and weight‐band at DTG start. Safety outcomes were frequency of clinical adverse events (AEs) and serious AEs (SAEs) causally associated with DTG, incidence of laboratory abnormalities and DTG discontinuation.

**Results: **Of 1231 CALWHIV ever on DTG, characteristics at DTG start were: median [IQR] age 14 [11, 16] years, 50% male, 95% perinatal HIV, 42% Black ethnicity; 10% ART‐naïve, 49% ART‐experienced/suppressed (VL<200 c/ml), 13% ART‐experienced/viraemic (VL≥200 c/ml) and 28% ART‐experienced/unknown VL. Median duration on DTG was 93 [49, 163] weeks. Viral suppression was 88−91% throughout time on DTG; highest among those ART‐experienced/suppressed at DTG start (92−94%), lowest among ART‐experienced/viraemic at DTG start (72−83%).

Overall cumulative incidence (95% CI) of viral failure by 96 and 144‐weeks was 4% (3−6) and 8% (6−11), respectively. Incidence varied by ART/VL status at DTG start: lowest in ART‐experienced/suppressed and highest among ART‐experienced/viraemic (*p*<0.001, Figure).

**Figure**. OAB3803

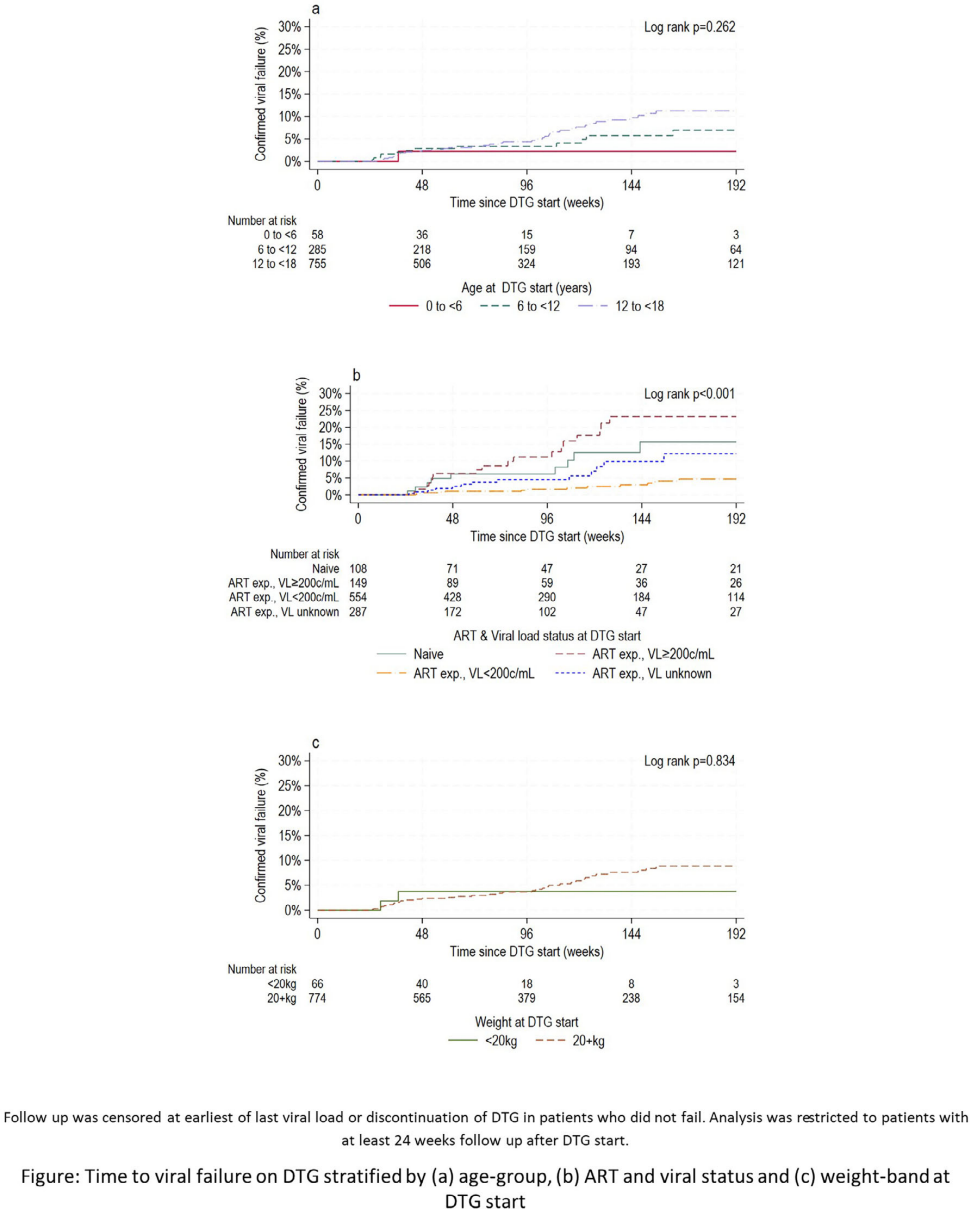

Of 1146/1231 (93%) with clinical data, 26 (2%) experienced 52 AEs causally related to DTG, including 5 SAEs (no deaths). Among 936/1231 (76%) with laboratory data, 49 (5%) experienced 57 DAIDS grade ≥3 events; a rate of <1 per 100 person‐years. Overall, 95 (8%) discontinued DTG at median 90 [36, 138] weeks: 5 (5%) due to viral failure, 17 (18%) toxicity and 73 (77%) treatment simplification/other reasons. Cumulative incidence of discontinuation was 5% (4−7) and 10% (8−12) by 96 and 144 weeks, respectively.

**Conclusions: **DTG was generally well tolerated with high viral suppression; overall incidence of viral failure was low but significantly higher among those ART‐experienced/viraemic at DTG start.

### OAB3804 Extended efficacy and safety of dolutegravir and darunavir containing regimens at week 96 in the international randomized clinical trial: D^2^EFT

N. Kumarasamy ^1^, on behalf of D2EFT Study Group

^1^Voluntary Health Services, Chennai Antiviral Research and Treatment (CART), Chennai, India

**Background: **D^2^EFT is an international randomized trial comparing dolutegravir (DTG) with ritonavir boosted darunavir (DRV/r) versus DTG with fixed tenofovir and lamivudine or emtricitabine (TDF/XTC) versus standard of care (SOC:DRV/r+2NRTIs with a rotation of nucleosides or adaptation to HIV genotype) in adult people living with HIV‐1 whose first‐line NNRTI‐based therapy has failed. At the 48‐week primary endpoint, both intervention arms demonstrated non‐inferiority to SOC in terms of HIV‐RNA<50 copies/ml with the DRV/r+DTG arm also showing superiority.

**Methods: **Week 96 data are here presented as modified intent to treat analysis including all available data.

**Results: **Eight hundred and twenty‐six participants from 14 resource‐constrained countries were randomized: DTG+DRV/r (*n* = 271), DTG+TDF/XTC (*n* = 294), SOC:DRV/r+2NRTIs (*n* = 261). Median age 39 years, 55% female and 69%, 25% and 2% of Black, Asian and White ethnicity, respectively. Median CD4 was 206 cells/mm^3^ and median HIV‐RNA was 15,400 copies/ml. Median BMI was 23 kg/m^2^.

By week 96, proportions remaining on original randomized regimen were 98%, 97% and 91% for DTG+DRV/r, DTG+TDF/XTC and DRV/r+2NRTIs, respectively. At 96 weeks when compared to the SOC, the percentage with HIV‐RNA<50 copies/ml was significantly higher for both DTG+DRV/r (85.6% vs. 76.0% [difference 9.6% (95% CI: 2.7−16.4), *p* = 0.01]) and DTG+TDF/XTC (81.6 vs. 72.6% [difference 9.0% (95% CI 1.4, 16.6)], *p* = 0.02). In a snapshot analysis in which response at week 96 was defined as remaining on randomized regimen and HIV‐RNA <50 copies/ml, the proportions were 68.6% SOC, 77.9% DTG+DRV/r and 76.5% DTG+TDF/XTC (*p*<0.05 for both intervention arms against SOC).

Overall mean CD4 gain to week 48 was 156 cells/mm^3^ and by week 96 was 204 cells/mm^3^. At week 96, CD4 count gain was on average 53 cells greater in the DTG+DRV/r arm and 35 cells greater in the DTG+TDF/XTC arm compared to SOC (*p*<0.003 and 0.06, respectively).

Mean weight gain over 96 weeks was 4.1 kg (SD 7.2), 7.4 kg (SD 7.9) and 5.8 kg (SD 8.1) in SOC, DTG+DRV/r and DTG+TDF/XTC arms, respectively. Trajectory of weight gain in DTG+TDF/XTC versus SOC plateaued after week 48 but continued in DTG+DRV/r.

**Conclusions: **Extended week 96 analysis in the D^2^EFT study confirms the efficacy and tolerability of DTG‐containing regimens with both arms reaching superiority against DRV/r+2NRTI for virological suppression to <50 copies/ml.

### OAB3805 Switching from a second‐line ritonavir‐boosted protease inhibitor‐based regimen to bictegravir/emtricitabine/tenofovir alafenamide: results of a randomized clinical trial

S. Pierre ^1^, J. B. Marc^1^, F. Homeus^1^, G. R. Bernadin^1^, L. Trevisi^2^, E. Jean^1^, E. Dumont^1^, S. Sundaresan^3^, V. Rivera^1^, D. Israelski^4^, S. E. Collins^4^, J. W. Pape^5^, B. Liautaud^1^, P. Severe^1^, P. E. Sax^6^, S. Koenig^6^

^1^GHESKIO, Medicine, Port‐au‐Prince, Haiti, ^2^Harvard Medicine School, Global Health and Social Medicine, Boston, United States, ^3^Analysis Group, Inc, Boston, United States, ^4^Gilead Sciences, Inc, Foster City, United States, ^5^GHESKIO, Medicine, Port‐au‐Prince, Haiti, Haiti, ^6^Brigham and Women's Hospital, Medicine, Boston, United States

**Background: **Patients on ritonavir‐boosted protease inhibitor (PI/r)‐based regimens in resource‐limited settings have high rates of nucleoside reverse transcriptase inhibitor (NRTI) resistance, but testing is rarely available. This study compared continuing PI/r + NRTIs versus B/F/TAF in PWH on second‐line ART with no prior drug resistance testing.

**Methods: **This prospective, open‐label trial conducted at GHESKIO in Haiti, randomized adults (>/ = 18 years) with viral suppression on second‐line PI/r‐based ART to continue current regimen versus switch to B/F/TAF. The primary endpoint was the proportion of participants with HIV‐1 RNA >/ = 200 copies/ml at week 48 using the food and drug administration (FDA) snapshot algorithm; difference between groups was assessed with non‐inferiority margin of 4%.

**Results: **Between October 2020 and March 2023, 310 participants were randomized and treated (B/F/TAF: 153; PI/r: 148). Median age was 50 years (IQR 42, 58) and 173 (57%) were women. At enrolment, 180 (59.8%) were taking lopinavir/r and 121 (40.2%) atazanavir/r; 234 (77.7%) were taking TDF, 54 (17.9%) zidovudine and 13 (4.34%) abacavir; all were taking lamivudine or emtricitabine. Median time on PI/r was 3.7 years (IQR 2.2, 5.7). At week 48, the proportion with HIV‐1 RNA >/ = 200 copies/ml was 0.7% (1/153) and 3.4% (5/148) in the B/F/TAF and PI/r groups, respectively: difference −2.7 (95% CI: −6.7 to 1.2), meeting non‐inferiority for B/F/TAF compared to PI/r (Table 1). One hundred and forty‐four (94.1%) and 135 (91.2%), respectively, had 48‐week HIV‐1 RNA <200 copies/ml. There were no drug discontinuations due to adverse events in either group. Baseline archived proviral DNA (B/F/TAF group) and genotypic resistance testing for virologic failures (both groups) are pending. This study was conducted during both COVID‐19 and severe civil unrest and gang‐related violence in Haiti. Follow‐up was enabled by community health workers and neighbourhood drug distribution.

**Table 1:** OAB3805: Primary end point—virologic outcomes at week 48

Week 48 outcome	B/F/TAF (*n* = 153)	Boosted PI (*n* = 148)
Primary end point: HIV‐1 RNA >/ = 200 copies/ml	**1 (0.7** **%)**	**5 (3.4%)**
HIV‐1 RNA >/ = 200 copies/ml in 48‐week window	0	3
Treatment discontinued before week 48 owing to lack of efficacy	0	0
Died or LTFU with last available HIV‐1 RNA value of >/ = 200 copies/ml	1	2
HIV‐1 RNA <200 copies/ml in 48‐week window	**144 (94.1%)**	**135 (91.2%)**
No data for final outcome (censored)	**8 (5.2%)**	**8 (5.2%)**

**Conclusions: **Switching virally suppressed adults on a second‐line PI/r regimen to B/F/TAF is non‐inferior to continuing PI/r‐based ART. Rates of viral suppression were high in both groups.

### OAC0802 Understanding barriers and facilitators to doxycycline post‐exposure prophylaxis adherence among young women in Western Kenya: a qualitative study

B. Kwach ^1^, Z. Kwena^1^, L. Violette^2^, J. B Odoyo^1^, K. Oware^1^, J. Simoni^3^, E. A. Bukusi^1,2,4^, J. M. Baeten^2^, J. Stewart^5,6^

^1^Kenya Medical Research Institute (KEMRI), Centre for Microbiology Research, Kisumu, Kenya, ^2^University of Washington, Department of Global Health, Seattle, United States, ^3^University of Washington, Department of Psychology, Seattle, United States, ^4^University of Washington, Department of Ob/Gyn, Seattle, United States, ^5^Hennepin Healthcare, Division of Infectious Diseases, Minneapolis, United States, ^6^University of Minnesota, Department of Medicine, Minneapolis, United States

**Background: **Sexually transmitted disease rates are high among women using pre‐exposure prophylaxis (PrEP) in sub‐Saharan Africa. Doxycycline post‐exposure prophylaxis (dPEP) effectively prevented STIs in trials among cisgender men and transgender women but not among cisgender women because the use of dPEP was low. Understanding the barriers to dPEP adherence in women is essential to interpreting trial results and to inform implementation.

**Methods: **We conducted a qualitative analysis nested in the dPEP Kenya study, an open‐label randomized controlled trial of dPEP (200 mg of doxycycline taken within 72 hours of exposure) among 449 cisgender women, aged 18−30 and taking HIV PrEP in Kisumu, Kenya. We completed serial, in‐depth interviews (*n* = 40) and four focus group discussions (FGDs) (*n* = 29) among women purposively sampled from 224 participants assigned to dPEP group between June 2021 and August 2023. In‐depth interviews (IDIs) and FGDs were audio‐recorded, transcribed and translated from preferred language to English for coding and analysis. An inductive content analysis approach was used to identify themes (Dedoose).

**Results: **Five main themes on barriers to dPEP adherence emerged (Table 1). We found that side effects, such as nausea from medication on an empty stomach, challenges in interpreting dosage instructions and pill burden were major barriers to dPEP adherence. Stigma associated with taking dPEP and fear of partner reaction also discouraged the use of dPEP. Three key themes emerged as facilitators to the use of dPEP in women, the perceived value of dPEP in preventing STI, familiarity with doxycycline and the use of a discrete pill case motivated some women to take dPEP.

**Table 1:** OAC0802: Facilitators and barriers to dPEP adherence

Factors	Themes	Category	Exemplary quotes
**Barriers to dPEP use**	Side effects (e.g. nausea/vomiting, fatigue, headache, photosensitivity, increased libido, smell of urine, taste in mouth, weight loss and interaction with menstrual cycle)	Challenges of taking dPEP on empty stomach	*“I am not used to doxy, so if I take it, I don't feel well. The first week I was given, I took them 6 times. Since it was giving me side effects like nausea, the following week I took it 4 times, so the following week I didn't use because of the side effects. I don't know how you will help me for the side effects”*
	Challenges in interpreting post‐exposure dosage instructions	Knowledge gap, trusting main partner	*“What I didn't know is to use doxy when I have sex with my boyfriend because he is someone I trust, and I can also say that he trusts me(laughter) its only that he doesn't know that I have other sex partners”*
	Pill burden	Challenges of taking dPEP with PrEP or other medication	*“This might discourage me if the number of pills that I am taking are many, if you think of adding dPEP on top of those you will feel discouraged. Let's say I am on anti‐malarial medication; I will have to finish this dose first in order for me to continue with the dPEP. If I am sick and I have been issued with medication from the hospital, this might make me discontinue the use of dPEP for a while.”*
	Fear of partner's reaction	Partner influence on decision to take dPEP and dosing time	*“There is main partner, and when he is around it is hard to take dPEP because I don't know what he will think of me.”*
	Stigma	Stigma associated with dPEP medication	*“The people I used to live with had no issues with me taking PrEP since they were also using it. But when it came to dPEP, I was the only one using it in that living space. So, they asked me if I intended to become more of a prostitute than them. They asked why I was using dPEP and said that it would make me increase sexual partners, which was somehow true. They felt that using dPEP made me a prostitute; a prostitute more than them. That was the challenge I had. Their words made me sit down at times and wonder whether I should keep taking the drug if my friends could tell me such things”*
**Facilitators to dPEP use**	Discrete pill case	AGYW desire private and concealed environment to take medication—at home and while travelling	*“What I do when traveling, there is a container that looks like that one for nail polish that I was given with dPEP. Whenever I remove it, nobody realizes that it is medicine because it may lead someone into thinking that I want to apply it, so that gives me an easy time”*
	STI risk awareness	Role of dPEP in STI prevention to continue with routine activities is valued	*“It's not hard for me to take it, for me to protect my life I must use it. With the way the situation is as at now, if you were to get an STI you will have to stay at home and your children will end up struggling. You have to leave your house and hustle”*
	Doxycycline familiarity	Perception of diverse uses of doxycycline as a motivation to take dPEP	*“I like doxycycline because it is not like PrEP. Some people will assume that you are using ARVs when they see PrEP. But with doxycycline, they may think that you are experiencing some stomach upset. They will not be able to understand why you are using it. That is why”*

**Conclusions: **Adherence to doxycycline prophylaxis could be better supported in this population by decreasing the frequency of dosing and urgency of dosing to allow for optimal location and timing of dosing. These findings are critical in contextualizing the null results of the dPEP Kenya study and informing future biomedical prevention interventions.

### OAC0803 Doxycycline PrEP prevents STIs without affecting vaginal bacterial flora in female sex workers

S. Abe ^1^, D. Shiojiri^2^, A. Kawashima^1^, H. Uemura^1^, N. Ando^1^, D. Mizushima^1^, H. Gatanaga^1^, S. Oka^1^

^1^National Center for Global Health and Medicine, Tokyo, Japan, ^2^Personal Health Clinic, Tokyo, Japan

**Background: **The efficacy and potential harm of doxycycline prophylaxis in women remain controversial. We investigated the impact of doxycycline pre‐exposure prophylaxis (DoxyPrEP) on reducing sexually transmitted infections (STIs) and its effect on the vaginal microbiome in female sex workers (FSWs).

**Methods: **Daily doxycycline (100 mg) was initiated in October 2022 at a private STI clinic in Tokyo, based on shared decision‐making. This analysis comprised a retrospective cohort study and a survey. Targeted STIs included *Chlamydia trachomatis*, *Neisseria gonorrhoeae* and syphilis. Routine clinical practice involved microscopic examination of vaginal smears every 1−3 months to monitor the vaginal microbiome. We compared incidence rates (per 100 person‐years) before and during DoxyPrEP, using incidence rate ratios (IRRs) with conditional fixed‐effects Poisson regression. Perturbations of the vaginal microbiome, defined by microscopic abnormalities and antimicrobial treatment, were also examined. Adherence to the regimen, adverse events and user satisfaction were monitored through surveys.

**Table:** Table. OAC0803

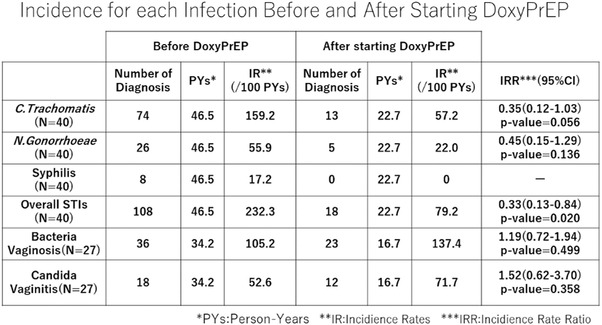

**Results: **Forty FSWs with a median age of 29 years (interquartile range 26−33.5) were analysed. Before DoxyPrEP, the overall STI incidence rate was 232.3 per 100 person‐years. After initiating DoxyPrEP, the overall STI incidence rate declined to 79.2 per 100 person‐years. There was a significant reduction in overall STIs (IRR = 0.33, *p* = 0.020) and a marginally significant reduction in *Chlamydia trachomatis* (IRR = 0.35, *p* = 0.056). The incidence of syphilis was reduced to zero. However, there was no significant change in *Neisseria gonorrhoeae* (IRR = 0.45, *p* = 0.136). Incidences of bacterial vaginosis and *Candida vaginitis* did not significantly increase (IRR = 1.19, *p* = 0.499 and IRR = 1.52, *p* = 0.358, respectively). Of the 40 participants, 22 completed surveys, almost all participants adhered strictly to DoxyPrEP. No severe doxycycline‐related adverse events were reported. Condom use remained constant in 95.4% of participants. Regarding satisfaction, 72.7% of participants reported reduced anxiety about acquiring STIs.

**Conclusions: **DoxyPrEP significantly reduced STI rates in FSWs without significantly increasing other vaginal infections. This supports the introduction of DoxyPrEP in high‐risk populations.

### OAC0804 Sex workers’ challenges with condom use and their perspectives on HIV PrEP—lessons from a participatory qualitative study of sex workers’ health needs in Germany

E. Willems ^1^, M. Ceres^2^, U. Probst^3^, N. Sarma^4^, J. Claass^5^

^1^Deutsche Aidshilfe, Berlin, Germany, ^2^Berufsverband erotische und sexuelle Dienstleistungen, Berlin, Germany, ^3^Freie Universität, Institut für Sozial‐ und Kulturanthropologie, Berlin, Germany, ^4^Robert Koch Institut, Berlin, Germany, ^5^Centrum für HIV und sexuell übertragbare Infektionen in Altona (CASAblanca), Hamburg, Germany

**Background: **Sex workers (SWs) are at increased risk of acquiring HIV globally. However, there is little data on the situation in Germany. In order to improve the understanding of the health needs of SW in Germany, we conducted a participatory qualitative study. One aim was to assess the potential of HIV pre‐exposure prophylaxis (PrEP) for SW. This topic highlighted an important interaction with condom use.

**Methods: **Between 10/2022 and 04/2023, 10 peer researchers conducted 11 focus group interviews across Germany in five languages. The 80 participants were female, trans and male sex workers in various fields of sex work (street, escort, brothels) from 23 countries. The sample was characterized by high diversity through the large proportion of SW with vulnerabilities such as illegal residence status or drug addiction. The data were analysed using qualitative content analysis.

**Results: **More than half of the participants had never heard of PrEP. Identified barriers to PrEP knowledge include the belief that PrEP is only suitable for people who practice condomless sex. Female SWs who were interested in PrEP described fear of stigmatization due to the association of PrEP with condomless sex. Many participants reported frequent clients’ demands for condomless sex and perceived this as a growing trend. Some described how this trend, combined with exacerbated financial precariousness, pressure them to offer condomless sex. Some female and trans SWs mentioned the fear that widespread PrEP use intensifies this pressure. For most participants, PrEP seems an attractive HIV prevention method, whether because they practice condomless sex or as an additional strategy in case of condom breakage or stealthing.

**Conclusions: **PrEP education for SW needs to be expanded in Germany, especially for non‐MSM. To prevent stigmatization and pressure on SW to offer condomless sex, the association “PrEP = condomless sex” must be countered. Study participants suggested that PrEP should not be presented as an alternative to condoms, but as a combined protection method. SWs need support in negotiating condom use and prices of sexual services. Support could be peer‐to‐peer empowerment, professionalization measures and information for clients.

### OAC0805 PrEP perception and PrEP initiation among adolescent men who have sex with men and transgender women in Brazil

S. Seixas ^1^, N. Galvão^1^, B. Oliveira Leite^2^, F. Soares^2^, L. Amorim^2^, J. A. Pinto Junior^3^, D. B. Greco^4^, A. Grangeiro^5^, I. Dourado^2^, L. Magno^1,2^

^1^Universidade do Estado da Bahia, Departamento de Ciências da Vida—Campus I, Salvador, Brazil, ^2^Universidade Federal da Bahia, Instituto de Saúde Coletiva (ISC), Salvador, Brazil, ^3^Universidade Federal Fluminense, Departamento de Estatística Instituito de Matemática e Estatísticatica, Rio De Janeiro, Brazil, ^4^Universidade Federal de Minas Gerais, Departamento de Clínica Médica, Belo Horizonte, Brazil, ^5^Universidade de São Paulo, Departamento de Medicina Preventiva, São Paulo, Brazil

**Background: **Perceptions about PrEP are multifaceted, shaped by influences from friends, family and social networks. These perceptions can impact the decision to start PrEP. We aimed to describe patterns of PrEP perceptions and their association with PrEP initiation among adolescent men who have sex with men (AMSM) and transgender women (ATGW).

**Methods: **PrEP1519 constituted a single arm, multicentric demonstration cohort study focusing on daily oral PrEP among AMSM and ATGW aged 15−19 years, in three Brazilian cities. Our analysis encompassed baseline data collected from February/2019 to February/2023. Latent class analysis (LCA) was used to identify patterns of PrEP perception based on eight observable binary indicators (agree or disagree). Logistic regression was conducted to estimate adjusted odds ratios (aOR) of the association between PrEP perception and the outcome PrEP initiation (PrEP dispensing within 30 days of starting in the cohort vs. not initiation PrEP).

**Results: **One thousand and four seventy‐seven adolescents enrolled in the study, with 91.0% identified as MSM,74.5% falling within the 18−19 age group, 72.0% self‐reported Black/Brown skin colour and the majority, 81.4% initiated PrEP. The prevalence of PrEP perception indicators were: 33.2% considered PrEP to be the same medication for treating HIV, 26.0% believed it had many side effects, 14.0% thought it was exclusively for MSM or TGW, 11.0% believed it was only for those with numerous partners, 9.0% were concerned about interactions with other medications like hormones, 24.6% thought it could negatively impact one's image, 8.0% found it inconvenient that PrEP was the same as HIV treatment and 10.0% perceived it as a hassle to take preventive medication. Classes were distinguished by “positive PrEP perception” (*N* = 1350; 93.2%), described by very low probabilities of agreement with specific indicators and “negative PrEP perception” (*N* = 98; 6.8%), represented by higher prevalences for the indicators. Adolescents with a positive PrEP perception were more likely to initiate PrEP (aOR: 2.46: 1.37–4.41) adjusted by potential confounders.

**Conclusions: **Our findings underscore the significance of the initiation decision for PrEP and highlight the influential role of perception in this process among adolescents. Moreover, it is crucial to foster a positive perception of this preventive strategy to enhance its adoption among adolescents.

### OAC1002 Multi‐level community‐based HIV prevention intervention for people who inject drugs on the US‐Mexico border: an effectiveness evaluation

G. Perez ^1^, N. Ludwig‐Barron^2^, V. Alan^3^, J. Puentes^4^, J. Salazar^5^, R. Maria Elena^6^, S. John^7^, J. Lechuga^1^

^1^Hunter College, Psychology, New York, United States, ^2^University of California San Francisco, Prevention Medicine, San Francisco, United States, ^3^The University of Texas at El Paso, Psychology, El Paso, United States, ^4^The University of Texas at El Paso, Public Health, El Paso, United States, ^5^University of California San Francisco, Medicine, San Francisco, United States, ^6^Maria Elena Ramos, Ciudad Juarez, Mexico, ^7^University of California San Francisco, Prevention Medicine, San Francisco, United States

**Background: **Prevalence of injection drug use on the US‐Mexico border is three times the national average. Border communities are places where the risk of drug use harms and HIV acquisition is augmented due to the confluence of factors operating across the physical, social, economic and policy environment. A theory informed multilevel, community‐based, behavioural intervention was implemented to promote engagement in harm reduction behaviours among people who inject drugs (PWID).

**Methods: **The behavioural intervention consisted of three components aimed at ameliorating the negative influence of factors operating at the structural, interpersonal and individual levels: (1) rapid HIV testing at community sites; (2) peer led multi‐session psychoeducational harm reduction sessions to promote self‐efficacy among small networks of PWID; and (3) community wide events to reduce HIV stigma. A quasi‐experimental design was employed consisting of time repeated survey assessments administered at 6 and 12 months after the rollout of intervention components. Participants were recruited through respondent driven‐sampling.

**Results: **Three hundred and fifty‐five PWID (70% male) with a mean age of 39.53 years (SD = 10.93) were recruited to participate in the cross‐sectional assessment surveys. Participants reported a mean of initiating injection drug use of 21 years and injecting a mean of 5.6 times daily. Three generalized estimating equations testing the influence of exposure to intervention components controlling for assessment time indicated that exposure to the three intervention components compared to exposure to no intervention components increased safe‐injection (M = 3.6 vs. M = 2.5), β = .65, *p* < .01), engagement in a greater number of preventative behaviours (M = 4.06 vs. M = 2.4, β = 1.65, *p* < .01), reduction in the number of condomless sex (M = 29.04 vs. M = 65.04, β = −.76, *p* < .05) and reduction in HIV stigma (M = 2.29 vs. M = 2.68, β = −.30, *p* < .05).

**Conclusions: **Findings suggest that the intervention was effective in reducing drug use harms and risk of HIV acquisition by promoting engagement in preventative behaviours and reducing stigma. Future directions include testing which combination of components is most effective under resource constraints to promote scalability.

### OAC1003 Leading the way: indigenous communities successfully advancing HIV care outcomes post‐pandemic

I. Khan ^1^, M. Andkhoie^1^, D. Bryant^2^, G. Cote^3^, C. Shingoose^4^, S. Key^5^, C. Roache^1^, L. Matz^1^, R. Ramsingh^1^, S. Skinner^6^, C. McArthur^5^, C. Wishnevetski^1^, L. Jagoe^1^, A. Wiebe^1^, T. Wong^7^

^1^Government of Canada, Indigenous Services Canada, Regina, Canada, ^2^Cote First Nation, Health, Cote First Nation, Canada, ^3^Cote First Nation, Chief, Cote First Nation, Canada, ^4^Keeseekoose First Nation, Health, Keeseekoose First Nation, Canada, ^5^The Key First Nation, Health, The Key First Nation, Canada, ^6^Wellness Wheel, Regina, Canada, ^7^Government of Canada, Indigenous Services Canada, Ottawa, Canada

**Background: **The COVID‐19 pandemic significantly affected timely access to healthcare, including access to HIV care in rural parts of Saskatchewan, Canada. In the southeast region, Indigenous PLWH have shared personal stories about encountering stigma and discrimination within the healthcare system. In response to these challenges, Indigenous leadership took a proactive role, focusing on improving testing and diagnosis for sexually transmitted and blood‐borne infections (STBBIs) and achieving notable milestones in HIV care since the region declared an HIV outbreak in 2016.

**Description: **Local Indigenous leaders made pivotal commitments to cultural approaches that emphasize trust‐building and normalized access to HIV prevention and care among equity‐denied populations. These strategies encompassed establishing convenient testing locations with culturally grounded, respectful, trauma‐informed and compassionate prevention and care services that provide comprehensive support, including transportation, family support, food incentives and land‐based therapies.

**Lessons learned: **Between 2018 and 2022, the area achieved a 100% timely linkage to care for new HIV cases and ensured treatment for all diagnosed individuals within 12 months. In 2022, 81% of the PLWH in the area achieved viral suppression. From 2018 to 2022, HIV and hepatitis C virus (HCV) screenings increased by 403% and 448%, respectively. During the same period, over 532,000 new syringes and 2700 safer inhalation kits were distributed. New HCV diagnosis decreased by 57% during this period, and new HIV diagnosis decreased from the rates of 45 cases per 100,000 population to zero cases. Timely care prevented mother‐to‐child transmission, resulting in seven HIV‐negative births among pregnant PLWH since 2020.

**Conclusions/Next steps: **These initiatives resulted in a significant rise in testing uptake, the establishment of on‐site infectious disease care clinics, prompt prescription linkages and pharmacy support. Active harm reduction and wellness outreach, along with rural‐to urban prenatal referrals, contributed to a decline in HCV and HIV cases. In addition, the measures resulted in achieving viral suppression for the majority of PWLH, and the adoption of “U = U” (Undetectable equals Untransmittable) by health professionals impacted and improved the STBBI outcomes in the area. The consistent and frequent support from local leadership, coupled with cultural approaches, played a crucial role in rebuilding trust and optimizing health outcomes among PLWH.

### OAC1004 Addressing substance use: decreased problematic substance use among young transgender women in Brazil—findings from a peer‐led intervention study

C. Coutinho ^1^, E. Jalil^1^, T. Wargas^1^, N. Bertoni^2^, C. Arsolino^1^, E. Peixoto^1^, C. Jalil^1^, M. St Silva^1^, R. Moreira^1^, M. Ramos^1^, S. W Cardoso^1^, V. Veloso^1^, L. Monteiro^1^, B. Grinsztejn^1^, E. Wilson^3^, BeT Study Group

^1^Instituto Nacional de Infectologia Evandro Chagas, Rio de janeiro, Brazil, ^2^INCA, Rio de janeiro, Brazil, ^3^Center for Public Health Research, San Francisco Department of Public Health, San Francisco, United States

**Background: **Substance use linked to challenging social conditions may increase HIV risk among young trans women (YTGW) in Brazil. This study aimed to evaluate substance use among Brazilian YTGW and identify factors associated with a high risk of problematic substance use (PSU).

**Methods: **Data from BeT, a 48‐week status‐neutral peer‐led HIV systems navigation intervention study in Rio de Janeiro, Brazil (February 2022−August 2023), targeting YTGW (18−24 years old) to improve HIV prevention/care outcomes were analysed. Baseline to follow‐up visits data (48‐week) were compared to assess changes in lower (0−10 for alcohol, 0−3 for others), moderate (11−26 for alcohol and 4−26 for others) and high‐risk PSU (27+) using ASSIST. Anxiety/depression screenings used PHQ4 (moderate/severe >5). Factors associated with moderate/high risk of any PSU (except tobacco/alcohol/marijuana) were identified using adjusted logistic regression models.

**Results: **Among 164 participants, most were aged 20−24 years (74%), identified as Black/Pardo (66%), higher than secondary education (66%) and 49% reported transactional sex. Substance use prevalence at baseline included alcohol (82%), binge drinking (74%), marijuana (66%), tobacco (65%), cocaine/crack (12%), inhalants (9%) and amphetamines (8%). Moderate/high‐risk of PSU decreased significantly from baseline to follow‐up for all substances, especially tobacco (57.7%−39.7%), alcohol (22.5%−0.9%), marijuana (62.9%−50.9%), crack/cocaine (8.6%−4.3%), amphetamines (3.4%−0.9%) and inhalants (8.6%−0.9%) (see Figure). No participant had high risk of PSU at follow‐up. High risk of any PSU (except tobacco/alcohol/marijuana) at baseline was associated with lower education (aOR: 4.12 [95% CI: 1.02−17.02]; *p*‐value = 0.046), binge drinking (aOR: 5.92 [95% CI: 1.55−39.36]; *p*‐value = 0.024) and moderate/severe anxiety/depression score (aOR: 4.13 [95% CI: 1.22−17.23]; *p*‐value = 0.032).

**Figure**. OAC1004

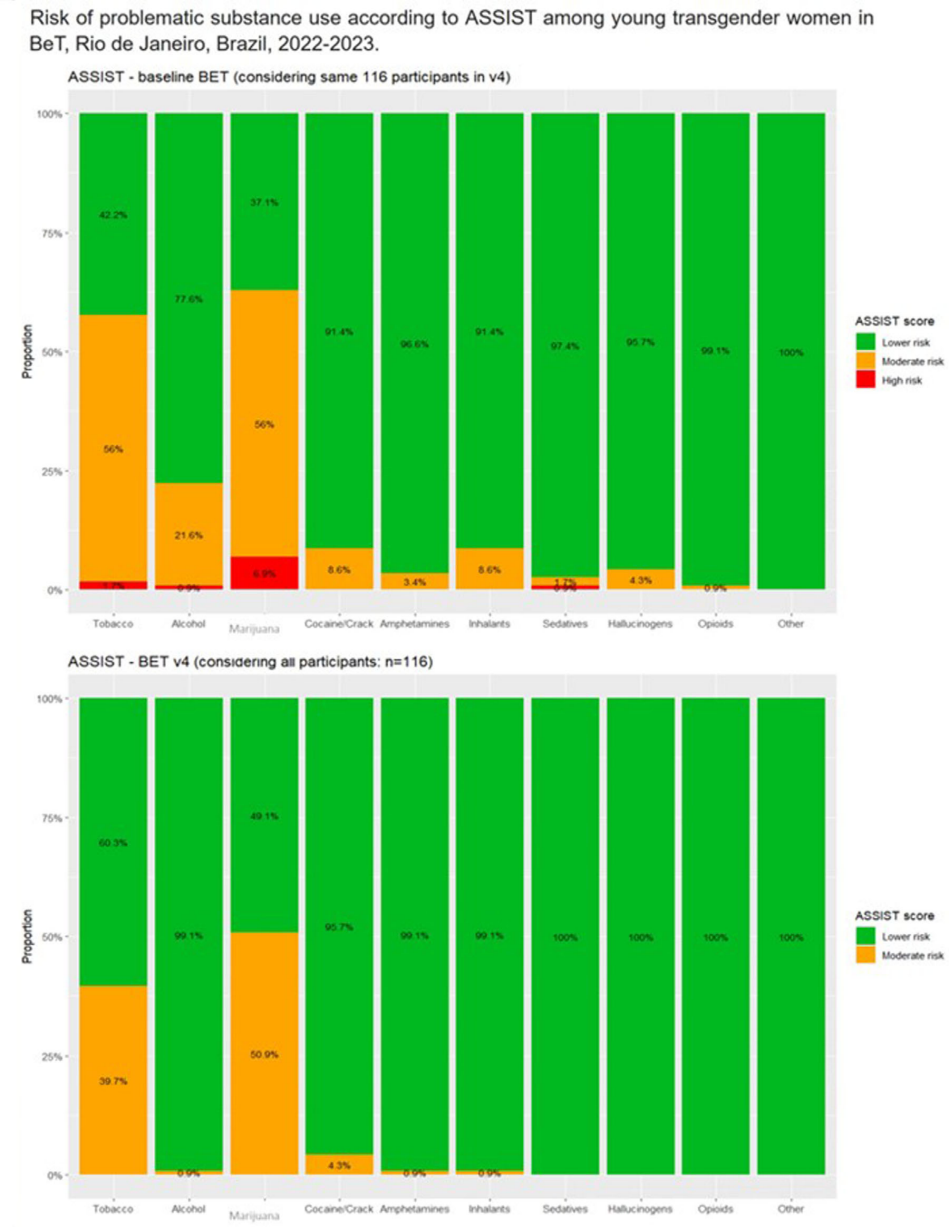

**Conclusions: **An unanticipated outcome of the intervention was a significant decrease in problematic substance among YTGW in Brazil, indicating the relevance of intervention components beyond HIV. PSU was highly correlated with lower education, binge drinking and mental distress among YTGW in our intervention. Intensive peer‐led systems navigation interventions may serve as models for substance use reduction with YTGW.

### OAC1005 Effect of training and clinical mentoring on prevention, linkage and retention to HIV care among key populations in Zambézia province, Mozambique

C. Orlando de Castro Rafael^1^, C. Belo^1^, J. Seleme^2^, G. Maibaze^3^, C. De Schacht^1^, C. W. Wester^4,5^, J. Tique ^1^

^1^Friends in Global Health (FGH), Maputo, Mozambique, ^2^Ministry of Health, National Directorate of Public Health, HIV Program, Maputo, Mozambique, ^3^US Centers for Disease Control and Prevention (CDC), Division of Global HIV & TB, Maputo, Mozambique, ^4^Vanderbilt University Medical Center (VUMC), Department of Medicine, Division of Infectious Diseases, Nashville, United States, ^5^Vanderbilt Institute for Global Health (VIGH), Nashville, United States

**Background: **Key populations (KPs), that is sex workers, men who have sex with men, transgender people, people who inject drugs and people in prisons and other closed settings, experience stigma, discrimination and structural barriers when accessing services for HIV prevention and care. We describe the effect of interventions implemented to strengthen the provision of KP‐friendly services at 128 health facilities (HFs) in Zambézia Province, Mozambique.

**Description: **We used a quality improvement conceptual framework to understand barriers to the provision of KP‐friendly services, design and implement interventions. Lack of appropriate provider training was the main barrier identified. Evaluation occurred over 2 years, including pre‐intervention (October 2021−September 2022) and during‐intervention (October 2022−September 2023) periods. Interventions included: (1) training 85 clinicians and 103 lay staff, some of whom self‐identify as KP, on providing KP‐friendly services; (2) performing 2245 one‐on‐one mentoring sessions to clinicians and lay staff from 128 HF (average six sessions per HF) on KP‐related skills using standardized tools; and (3) a monitoring strategy including data triangulation. Aggregated clinical data on pre‐exposure prophylaxis (PrEP), antiretroviral therapy (ART) linkage and uptake, viral load (VL) coverage and suppression were used to assess trends.

**Lessons learned: **Results show an increase in PrEP initiation among KP by 171%, with 5772 individuals initiating PrEP during‐implementation compared to 2130 pre‐implementation. Linkage to ART services increased 22.6% from 62% pre‐ to 76% during‐implementation. The number of individuals currently receiving ART increased by 39% from 5191 pre‐implementation to 7189 during‐implementation. Testing for VL coverage (77%) during‐implementation remained the same as in the pre‐implementation period (Table), while VL suppression (91%) increased compared to pre‐implementation (87%).

**Table:** Table. OAC1005: HIV services for key populations, pre‐ and during‐implementation in Zambézia Province

	Pre‐implementation	During‐implementation	Percentage change
**Tested for HIV^a^ (*n*)**	10,417	10,999	5.6%
**Negative test result (*n*)**	8331	8994	8.0%
Initiated PrEP (*n*)	2130	5772	170.9%
**Positive test result (*n*, %)**	2086 (20%)	2005 (18%)	−3.9%
Linkage to ART^b^ (%)	62%	76%	22.6%
Initiated ART (*n*)	1975	2091	5.9%
Currently receiving ART (*n*)	5191	7189	38.5%
Tested for VL in last 12 months (*n*)	3177	4720	48.6%
VL coverage^c^ (%)	77%	77%	0%
VL suppression^d^ (*n*, %)	2777 (87%)	4282 (91%)	54.2%

^a^Some persons could have been tested several times, as guidelines recommend HIV testing for KP 4x/year (in 2021/2022) or 2x/year (as of 2023).

^b^Linkage to care is calculated by dividing the total number of persons initiating ART with the total number of persons living with HIV identified during the specific period (may include individuals tested by other institutions/partners).

^c^VL coverage is a proxy indicator calculated by dividing the number of individuals with one VL result registered within the last 12 months and the number of individuals on ART 6 months prior.

^d^Viral suppression is calculated by dividing the number of individuals with a VL result less than 1000 copies/ml and the total number of individuals with a VL result registered.

**Conclusions/Next steps: **These findings highlight the favourable effect of training and clinical mentoring on key HIV prevention and care outcomes in the HF context in resource‐constrained settings. The use of clinical quality improvement methods can be a meaningful tool to identify barriers and develop solutions to improve the provision of KP‐friendly services.

### OAC1502 Unveiling a new path of hiv services in prison setting in Bangladesh

M. Akhtaruzzaman ^1^, F. Khatun^1^

^1^AIDS/STD Programme, Ministry of Health & Family Welfare, Dhaka, Bangladesh

**Background: **Bangladesh faced challenges in achieving the first 95 target for HIV, with a current standing at 73%. The AIDS/STD Programme (ASP) took a proactive role in enhancing HIV testing services, focusing on overlooked areas like prisons. Unfortunately, progress was hindered by the absence of established testing and prevention facilities, influenced by restrictive policies. Across the nation's 68 prisons, living 90,000 inmates. Programme data revealed 1110 people who inject drugs (PWID) in jail between 2018 and 2020, with 169 on opioid substitution therapy (OST) and 114 HIV positive. The lack of HIV services in prisons resulted in the discontinuation of OST and antiretroviral therapy (ART). In response, ASP launched a project in 2021 aiming to integrate HIV services within prison hospital settings.

**Description: **In June 2021, ASP engaged in advocacy with the prison directorate, conducting workshops to present data and evidence for approval from the Ministry of Home Affairs. On 21 September 2022, an MoU was signed, enabling ASP to intervene in nine selected prisons prioritized based on HIV prevalence districts. Human resources were deployed, prison staff were trained to identify and test inmates, especially key populations (KPs). Testing began in October 2022, with 7976 HIV and 5596 syphilis tests conducted by December 2023, identifying five new HIV and 140 syphilis cases, all enrolled in HIV and STI treatment. In three prisons, 144 PWID were identified, including 12 persons who were HIV positive, having entered jail for a short time. The project ensured antiretroviral drugs (ARVs) for short‐term cases, although 39 persons were OST recipients, but OST remained unavailable.

**Lessons learned: **Successful lessons included the establishment of testing facilities in high‐prevalence districts, with inmates showing willingness to undergo testing. Three ARV dispensing centres within prison hospitals improved access for HIV‐positive inmates.

**Conclusions/Next steps: **The imperative next step is to expand HIV services to all 68 prisons, as motivated by the Directorate of Prisons. While the pilot phase from 2021 to 2023 did not initiate OST in selected prisons, efforts are underway to introduce this service in districts where NGO facilities offer it. Valuable insights from the end‐of‐grant cycle survey results in February 2024 will guide future planning and improvements.

### OAC1503 Breaking the barriers: opportunities for integrating HIV testing with other services for incarcerated population. Results from Plan India's Prison and OCS intervention funded by GFATM in 13 Indian states

K. Biswas ^1^, R. Rana^1^, A. Rawat^1^, B. Borah^1^, M. Asif^1^

^1^Plan International (India Chapter), New Delhi, India

**Background: **Though estimated national adult prevalence remained low in India 0.20% (0.17−0.25%) in 2022, the observed HIV prevalence among inmates in jails remained very high. 1.93% (95% CI: 1.75−2.12). Complementing Govt. of India's effort to end AIDS by 2030, Plan India is implementing Prison and Other Close Settings (OCS) intervention in 13 priority Indian states through an integrated HIV prevention project funded by The Global Fund.

**Description: **Plan India in collaboration with state and central Govt. has introduced an Integrated HIV prevention service along with STI, Hep‐B, Hep‐C and TB for the incarcerated population in 357 prisons and 218 other closed settings since 22 September with a focus to undertrials. The project targeted to (A) mapping all the prison and OCS in project states, (B) expand and strengthen HIV and priority disease screening facility in the prison set up, and (C) capacitate prison peer volunteers (PPVs) among prison inmates towards sustainability.

**Lessons learned: **Eighty seven percent (*n* = 320,825) of the incarcerated population those received HIV testing during the period of 22 September−23 September were undertrial inmates. One thousand four hundred and seventy‐nine inmates identified as HIV positive, while positivity varied widely within the project states 0.44% (0.1−4.97). Seventy seven percent have been linked with ART treatment within 30 days. Eighty percent of newly identified HIV‐positive inmates (*n* = 1479) are from injecting drug use background. Out of the inmates tested for TB, 1.93% (*n* = 240,780) were identified as symptomatic and 0.91% (*n* = 4487) were diagnosed with TB. STI screening resulted in 0.57% (*n* = 38,642) cases diagnosed, and 1.19% (*n* = 78,642) inmates were identified as Hep‐C positive. Around 6000 prison peer volunteers have been identified and trained to carry out community‐based HIV screening and providing ART adherence support to HIV‐positive inmates.

**Conclusions/Next steps: **The result suggests a significant impact created within the prison setting with the integrated HIV and other priority disease screening among incarcerated population. Advocacy with Govt. departments and collaboration with prison officials has resulted in ensuring increase in identification and early linkage of HIV‐positive inmates. Plan India's Prison intervention shows the pathways to expand and saturate the incarcerated population with integrated package of screening services and beyond.

### OAC1504 AEGIDA: an intervention to support uptake of HIV self‐testing and PrEP among women who exchange sex and/or use substances in Kazakhstan

V. Frye ^1^, B. West^1^, M. Darisheva^2^, N. Zholnerova^3^, E. Grigochuk^4^, A. Terlikbayeva^2^, S. Primbetova^2^, T. McCrimmon^5^, L. Gilbert^1^, M. Chang^6^, N. El‐Bassel^1^

^1^Columbia University School of Social Work, Social Intervention Group, New York, United States, ^2^Global Health Research Center of Central Asia, Almaty, Kazakhstan, ^3^Amelia, Almaty, Kazakhstan, ^4^Independent Consultant, Almaty, Kazakhstan, ^5^Columbia University Mailman School of Public Health, New York, United States, ^6^Columbia University School of Social Works, New York, Virgin Islands, United States

**Background: **Women who exchange sex and/or use substances (WESUS) face stigma‐related barriers to HIV testing, the gateway to PrEP/PEP and ART. In Kazakhstan, HIV self‐testing (HST), a user‐controlled method, may increase consistent HIV testing, but it is a new option for WESUS and interventions are needed to promote and increase uptake.

**Methods: **AEGIDA is a four‐session intervention designed for WESUS to support consistent HIV testing via HST training and PrEP awareness. To create AEGIDA, we conducted formative research (30 interviews/4 focus groups) identifying preferences/barriers/facilitators of HST and applied modified intervention mapping to adapt an evidence‐based intervention (TRUST). To evaluate AEGIDA, we screened 305 women (47% eligible) and enrolled 90 women (six transgender women), who completed baseline self‐interviews and were randomized in a 2:1 assignment to active (AEGIDA) versus a time‐attention control (didactic self‐screening) conditions with 6‐month follow‐ups. AEGIDA's theoretically based intervention sessions include evidence‐based techniques (motivational interviewing, peer education, harm reduction, cognitive reframing and self‐compassion) to reduce internalized stigma and build HST skills (via videos created by sex worker advocates and hands‐on practice with facilitator) to increase consistent HIV testing. Sessions were delivered face‐to‐face and via videoconference, with a closed Instagram page for active condition participants.

**Results: **At baseline, average participant age was 39 (SD = 8.8); 37% completed ninth grade or less. Nearly half were homeless and two‐thirds were food insecure in the 3 months before enrolment. STIs were common (13 syphilis; 4 gonorrhoea; 17 trichomonas; 5 chlamydia). About half reported not using condoms with paying partners and getting extra money for it in the past 3 months. Just half had HIV tested in 6 months before enrolment. Preliminary analyses find that AEGIDA is acceptable and feasible to deliver. Session attendance was 100%, 92%, 87% and 80%; median number of days to complete all four sessions was 18.3 (4−63 days).

**Conclusions: **AEGIDA has the potential to promote HST and PrEP uptake by addressing key stigma‐related barriers to testing and prevention access among WESUS in Kazakhstan. When 6‐month follow‐up is complete in March 2024, we will calculate preliminary efficacy and analyse exit interview data, and disseminate our findings in collaboration with our community and governmental partners.

### OAC1505 Innovations in HIV testing. A story of how we utilized a failed grasshopper harvesting season to conduct moonlight HIV testing and counselling among young men in Uganda

A. Nanyonjo ^1,2^, F. Nabaweesi1, B. Kemigisa^2^, R.N. Kikonyogo^3,4^, L.S. Namara^3^, A. Bagarukayo^4,5^

^1^PillPower Uganda, Hoima, Uganda, ^2^DFCU Bank, Kampala, Uganda, ^3^Infectious Diseases Institute, Kampala, Uganda, ^4^Youth Space Uganda, Kyegegwa, Uganda, ^5^Center for Domestic Violence Prevention, Kampala, Uganda

**Background: **Today, young people (15−24) account for 40% of all new adult HIV infections. We utilized a failed business venture to mobilize youth to know their status. Grasshoppers are a delicacy and a very lucrative source of income in Uganda. The seasonal swarms are trapped only in the night using very bright lights and water drums. During the season, most young people employed in the informal sector usually co‐opt this business as an extra income. This business is accompanied with reckless lifestyle of drinking, partying and buying sex workers especially after receiving huge sums of money. This year, the anticipated grasshoppers did not come in time due to changes in weather. This rendered the businessmen idle and ​open to listening.

**Description: **Pill Power Uganda (PPU) utilized this opportunity to conduct HIV campaigns in the town centres where the businessmen had set up. YSU utilized projectors and screens to showcase testimonies from those living with HIV. The counsellors also discussed condom use, PreP and post‐exposure prophylaxis (PEP) and routine testing and counselling. The team then welcomed a Q&A session, followed by a free testing and counselling session.

**Lessons learned: **A total of 2809 young people (2630 males, 179 females) were reached in the HIV counselling and testing services with 32 (21 males, 11 females) testing HIV positive giving a positive rate of 1%. More men (94%) were reached with HIV testing services compared to females at 6%. The moonlight campaigns contributed to 35% of the total number of individuals who received HIV testing services in Kyegegwa district. The businessmen after knowing their status made resolutions to change their behaviour. The unexpected good outcome is that 411 of the most‐at‐risk populations (MARPs), for example commercial sex workers, long distance truck drivers and bar workers, were also attracted by the testimonies.

**Conclusions/Next steps: **Innovation of cost‐efficient approaches with massive reach is essential for continuity of HIV knowledge dissemination programmes. Utilization of “cover of darkness” events to offer HIV services reaches many young men particularly in the urban settings. YSU intends to research and utilize more of the moonlight business opportunities to conduct HIV awareness campaigns and testing.

### OAC1802 Let communities lead the HIV response: sharing experiences from key population civil society organizations in Uganda, 2022−2023

K. Kulu ^1^, L. Babirye^1^, R. Bbosa^1^, D. Wabomba^1^, J. Kigozi^1^, R. Lusimbo^2^, A. G. Fitzmaurice^3^, C. Ajulong^3^, S. Alamo^3^, N. Kalema^1^, F. Namimbi^1^

^1^Infectious Diseases Institute, Makerere University, Health Systems Strengthening, Kampala, Uganda, ^2^Uganda Key Population Consortium, Kampala, Uganda, ^3^U.S. Centres for Disease Control and Prevention, Entebbe, Uganda

**Background: **Community‐led solutions are necessary to mitigate public health threats, including HIV. PEPFAR funded the Infectious Diseases Institute (IDI) to support civil society organizations (CSOs) to create safe spaces in the community for delivery of person‐centred differentiated services to key populations (KPs). We assessed the contribution of 17 CSO safe spaces to HIV service delivery in Kampala and Wakiso districts of Uganda.

**Description: **We identified indigenous KP‐led CSOs and capacitated them into safe spaces, known as drop‐in centres (DICs). DICs were empowered to provide a package of comprehensive stigma‐free health HIV services to KPs using the PEPFAR KP service layering table. We trained, mentored and linked frontline DIC leaders and facility staff to deliver targeted HIV prevention strategies including HIV self‐testing in KP hotspots, linking HIV‐negative KPs to pre‐exposure prophylaxis (PrEP) or KPs with HIV to counselling for initiation or re‐engagement into antiretroviral therapy (ART) and care. All service data are routinely entered in a centralized data system known as the KP Tracker. We analysed data in the KP Tracker to compute the contribution of DICs as proportions to overall KP service provision performance for the period October 2022−September 2023.

**Lessons learned: **A total of 99,014 KPs received a package of at least three HIV services, including (95,229, 96%) who received an HIV test. Of these, only 14% (13,206) tested at CSO DICs. Among KPs identified as living with HIV, nearly half (603/1271, 47%) were tested at CSO DICs, of whom 599/603 (99%) initiated ART. DICs also contributed nearly half (7470/15,526, 48%) of KPs initiating PrEP.

**Conclusions/Next steps: **CSO DICs performed HIV case‐finding and initiated KPs on PrEP more efficiently compared to facility‐based KP programmes in Kampala and Wakiso districts. Well‐supported CSOs can successfully implement peer‐led solutions at community DICs to improve the delivery of HIV prevention and treatment services to KPs.

**Figure**. OAC1802

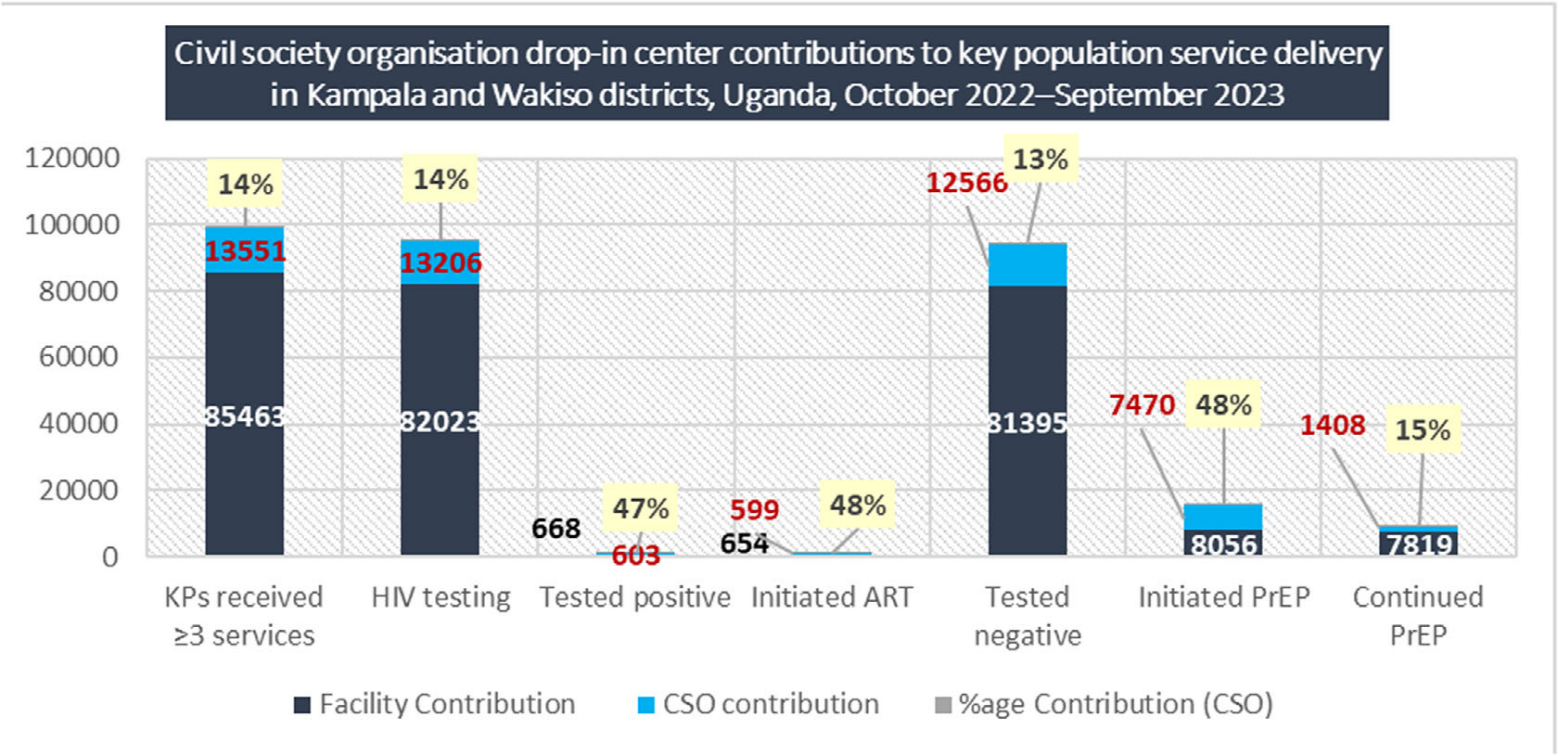

### OAC1803 HIV testing—community‐based peer‐led strategies; HIV testing to support case‐finding

K. Chiyenu ^1^, M. Musheke^2^, C. Siame^2^, A. Samona^2^, B. Ngosa^2^, F. Mwape^2^, S. Shebo^1^, M. Zimba^3^, M. Kabwe^4^, M. Mulenga^5^, C. Kanene^6^, A. Phiri^6^

^1^Centre for Infectious Disease Research in Zambia, Strategic Information, Lusaka, Zambia, ^2^Centre for Infectious Disease Research in Zambia, Implementation Science, Lusaka, Zambia, ^3^Key Population Alliance Zambia, Lusaka, Zambia, ^4^The Lotus Identity, Lusaka, Zambia, ^5^Sustainable Health Advocacy and Dynamic Empowerment Zambia, Lusaka, Zambia, ^6^United States Agency for International Development, Lusaka, Zambia

**Background: **Zambia has made significant progress (89:96:97) towards reaching the 95‐95‐95 targets. However, with 28,000 new HIV acquisition being reported annually, identifying and implementing effective HIV case‐finding strategies has become more imperative. We compare the effectiveness of three different strategies—social network strategy (SNS), mass mobilization and index testing—for case‐finding among key populations in seven districts of Zambia.

**Description: **The USAID‐funded Controlling HIV Epidemic for Key and Underserved Populations (CHEKUP) I is implementing a key populations (KP) programme in seven districts of two provinces of Zambia, targeting female sex workers (FSWs), men who have sex with men (MSM), transgender persons (TGs) and people who inject drugs (PWID). Community‐based KP Wellness Centers linked to government health facilities have been established, run by key populations civil society organizations (KP CSOs), who conduct community outreach, social network testing and index testing through their peer promoters and HIV counsellors.

**Lessons learned: **Utilization of a combination of HIV testing modalities is crucial to mobilizing KP for HIV services. Between December 2021 and September 2023, 25,709 KP were reached: 22,805 through community outreach, 1590 through SNS and 1314 through index testing. Overall, 23% tested HIV positive. Comparing the HIV testing modalities, index testing generated the highest positivity, at 41%, followed by SNS at 31%, with mass mobilization generating a comparatively low HIV positivity, at 20%. Further analysis of KP tested through the three different testing modalities indicated that treatment continuity was highest among those reached through SNS and index testing, at 77% and 70%, respectively, compared to those reached through mass mobilization, at 62%. Treatment continuity among KP reached through SNS and index testing was comparatively higher due to the utility of social networks for tracking and reaching KP.

**Conclusions/Next steps: **While HIV positivity was highest using SNS and index testing, mass mobilization was, by comparison, very effective in reaching a high number of KPs, which is also crucial for combination of HIV prevention. Therefore, as countries strive to achieve epidemic control, utilization of a combination of HIV testing modalities is crucial to reaching hard‐to‐reach population groups, such as KP, for HIV services.

### OAC1804 Exploring pre‐exposure prophylaxis uptake and continuation among gender and sexually diverse populations in Bangladesh: a pilot programme analysis

G. Sarwar ^1^, A. Rahaman^1^, M. Haque^1^, S. Islam Khan^1^

^1^International Centre for Diarrheal Disease Research, Bangladesh (icddr,b), Program for HIV and AIDS, Health Systems and Population Studies Division, Dhaka, Bangladesh

**Background: **Pre‐exposure prophylaxis (PrEP) has emerged as a ground‐breaking biomedical intervention in the fight against HIV. The efficacy of PrEP relies on strict adherence to the prescribed medication regimen, as consistent and correct use is crucial for optimal protection, and suboptimal adherence can diminish its effectiveness. In Bangladesh, there is a growing HIV epidemic, disproportionately affecting men who have sex with men (MSM), male sex workers (MSWs) and transgender women (locally known as hijra). This pilot programme analysis aims to investigate the patterns of PrEP uptake and continuation within these populations (KPs) in Bangladesh.

**Description: **The pilot intervention took place at two drop‐in centres (DICs) located in Dhaka city. From February 2022 to December 2022, a total of 208 participants (70 MSM, 100 MSW and 38 hijra) were enrolled in the PrEP intervention programme. Adhering to the World Health Organization's six‐step cascade, PrEP services were provided. PrEP continuation was prioritized through peer‐led navigation and digital platforms, including text messaging, social media (WhatsApp, Messenger) and audio‐visual counselling. Baseline data on HIV and STIs were collected, with follow‐up assessments at the third‐ and sixth‐month intervals.

**Lessons learned: **At the third‐month follow‐up, 96.2% of participants continued with PrEP, while 3.8% discontinued. By the sixth month, 87.5% of participants completed PrEP follow‐up services, with 12.5% unable to sustain PrEP usage. Prominent reasons for discontinuation included going abroad for job or study purposes (53.84%), relocation from intervention sites (15.38%) and being in a monogamous relationship (11.54%). Regarding STIs, the overall incidence among participants at baseline was 22.6%. From the third‐ to the sixth‐month follow‐up, the incidence of STIs decreased to 16.0% and 5.49%, respectively. No HIV‐positive cases were detected at the third‐ and sixth‐month follow‐up visits.

**Conclusions/Next steps: **The high retention rate at PrEP at the third‐month follow‐up suggests initial success in PrEP uptake, highlighting its potential to curb HIV transmission. However, discontinuation was observed between third and sixth months, emphasizing the need for targeted support and intense PrEP adherence counselling to maintain a good retention rate.

### OAC1805 “I am Manu, your virtual sexual health advisor”: using chatbot technology for innovative approaches and managing demand for HIV prevention among key populations in Central America

C. A. Palma Solórzano ^1^, A. Cabrera^1^, S. Lungo^1^

^1^Pan American Social Marketing Organization (PASMO), Guatemala City, Guatemala

**Background: **In 2023 and under the Prevention Services against HIV project implemented by the Pan American Social Marketing Organization (PASMO) with funds from the United States Agency for International Development (USAID), PASMO developed and launched “Manu,” the first HIV/STI‐related chatbot in Central America. Leveraging chatbot technology, “Manu” was designed to help manage the high online demand for HIV/STI prevention services and information generated under PASMO's cyber‐education and social media outreach strategies targeted to key populations (KPs), especially men who have sex with men (MSM), given that, from October 2022 to September 2023 alone, these strategies helped PASMO reach more than 18,000 at‐risk KPs through online channels and new technologies were needed to help respond to user needs for information and referrals to the project's prevention services.

**Description: **PASMO worked with an external development team to design a comprehensive chatbot decision tree that would allow “Manu” to address information about HIV and STI risks and automatically generate referral coupons to PASMO's free prevention services such as HIV testing, HIV self‐tests and PrEP. The chatbot was designed to operate within one of PASMO's Facebook Fan Page's Messenger apps 24 hours a day, 7 days a week, yet users can also request “human” to interact with a PASMO online outreach worker/“cyber‐educator.” Since its April 2023 launch, Manu reports more than 5000 entries from KPs seeking HIV/STI prevention information and services and more than 700 clients have been referred to project services.

**Lessons learned: **PASMO used audience insight collection tools and innovative eye‐tracking and heat map research to give a name and face to the chatbot which was illustrated in 3D to better interact with users. Chatbot tracking data show that more than 50% of KPs who access “Manu” do so outside of working hours (6 PM to 6 AM), evidencing the importance of 24/7 technology to manage demand and maintain user access to information and referrals.

**Conclusions/Next steps: **The “Manu” chatbot is proving to be an important tool to maintain the access of young and at‐risk KPs to HIV/STI information and service referrals at all times of the day and on weekends.

### OAC2202 Differences in risk factors between high and low vertical HIV transmission settings: Implications for elimination of paediatric HIV

A. Mahumane^1^, A. F. Lwilla^2^, K. Elsbernd ^3,4^, M. Rauscher^5^, B. Meggi^1^, K. Pereira^1^, J. Lequechane^1^, F. Chale^1^, S. Boniface^2^, R. Edom^2^, C. Mudenyanga^6^, M. Mueller^3^, W. C. Buck^7^, N. Taveira^8^, M. Hoelscher^3,5,9,10^, I. Jani^1^, N. E. Ntinginya^2^, A. Kroidl^3,9^, I. Sabi^2^

^1^Instituto Nacional de Saúde, Maputo, Mozambique, ^2^National Institute for Medical Research, Mbeya Medical Research Center, Mbeya, the United Republic of Tanzania, ^3^LMU University Hospital, Division of Infectious Diseases and Tropical Medicine, Munich, Germany, ^4^LMU Munich, Institute for Medical Information Processing, Biometry, and Epidemiology, Munich, Germany, ^5^Fraunhofer Institute for Translational Medicine and Pharmacology ITMP, Immunology, Infection and Pandemic Research, Munich, Germany, ^6^Clinton Health Access Initiative (CHAI), Maputo, Mozambique, ^7^University of California Los Angeles, David Geffen School of Medicine, Los Angeles, United States, ^8^Instituto Universitário Egas Moniz, Almada, Portugal, ^9^German Center for Infection Research (DZIF), Partner site Munich, Munich, Germany, ^10^Helmholtz Center Munich, German Research Center for Environmental Health (HMGU), Unit Global Health, Munich, Germany

**Background: **Roughly, 1.3 million infants are exposed and 150,000 newly diagnosed with HIV annually. Estimates of vertical HIV transmission (VHT) vary by setting. We assessed the risk factors for VHT among infants born to women living with HIV (WLWH) in Tanzania and Mozambique.

**Methods: **Between October 2019 and August 2021, we collected data from pregnant WLWH who participated in the EDCTP‐funded LIFE study at 28 primary health facilities in Tanzania and Mozambique. VHT was assessed up to month 3 for all infants and up to month 18 for a subset of infants. Demographics and clinical characteristics were collected to assess risk factors for VHT, including maternal HIV viral load measurements at baseline and month 3. Additionally, facility‐level programmatic factors including number of staff and annual HIV‐positive deliveries were collected. We used mixed effects models adjusted for health facility clustering to calculate odds ratios (OR) for VHT.

**Results: **In total, 6505 WLWH and their 6602 infants were included in the study with 1296 infants participating in the month 18 subset. VHT up to month 18 was 2.92% (95% CI: 2.42−3.49) in Mozambique, significantly higher than the 0.82% (95% CI: 0.51−1.24) observed in Tanzania (OR: 3.66, 95% CI: 2.31−6.12). On average, Mozambican mothers were significantly younger, attended antenatal care less frequently and had been on antiretroviral treatment for a shorter period. Maternity staff per 100 HIV‐positive deliveries was 9.9 (SD 5.0) in Tanzania and 2.3 (SD 1.0) in Mozambique (*p*<0.0001). After adjusting for these factors, virologic non‐suppression (>1000 copies/ml) at delivery was the principal risk factor for transmission (adjusted OR: 28.3, 95% CI: 15.7−50.9). In Mozambique, 31.0% of mothers were not suppressed at delivery compared to 8.1% in Tanzania; only 10.4% infants who acquired HIV had mothers who were virally suppressed at delivery.

**Conclusions: **We observed a striking difference in VHT between countries. Lack of viral suppression in the early postpartum period was the main risk factor for VHT, and we observed differences in programmatic factors between countries. These results highlight the need for a better understanding of the individual, community and health system factors associated with a lack of viral suppression in pregnant and lactating WLWH.

### OAC2203 Eliminating mother‐to‐child‐transmission of syphilis through the introduction of HIV/syphilis dual tests among pregnant women in Liberia, a national rollout success story

E. Efronson^1^, D. J. Flomo ^2^, M. Silikpoh^1^, M. K. Tobii^2^, A. Konstantanova^3^, K. Gray^2^, E. P. Jimmy^2^, A. Alvarez^3^, A. Forleh^2^, A. Kitchel^3^, C. Faire^1^, D. S. Dunbar^4^, W. K. Zaza^2^, G. M. Jackson^2^

^1^Evidence Action, Monrovia, Liberia, ^2^National AIDS and STI Control Program, Ministry of Health, Monrovia, Liberia, ^3^Evidence Action, Washington, D.C., United States, ^4^Redemption Hospital, ART, Monrovia, Liberia

**Background: **The WHO recommends the use of Dual HIV/Syphilis rapid diagnostic tests for screening pregnant women during antenatal care (ANC). Furthermore, women with HIV‐and‐syphilis‐co‐infection are 2.5 times more likely to transmit HIV to their children. Despite over 95% of pregnant women attending at least one ANC visit—of which 80% are screened for HIV, less than 8% of all pregnant women have been tested for syphilis. In September 2021, the National AIDS and STI Control Program (NACP) began to scale dual‐tests across Liberia to address this gap.

**Description: **Syphilis screening and treatment outcomes were estimated utilizing results from a survey administered across 67 facilities randomly sampled from 567 trained sites in 15 counties. We utilized a triangulation‐approach to estimate screening coverage combining the rates of commodity availability, provider knowledge and adherence to clinical guidelines, and patient consent. Using data from January to August 2023, we estimate that 77% (92,298/120,559) of pregnant women attending first ANC were screened for syphilis and that 2.7% (2529/92,298) were positive of whom 88% (2232/2529) received treatment.

**Lessons learned: **Introduction of dual‐testing increased syphilis screening by nearly 10 times (77%) almost mirroring that of HIV screening (80%), demonstrating that dual‐testing can be easily integrated in national HIV programmes. Empowering local county and district health teams with effective tools and training to lead on‐site training and supervision allows providers to offer quality services that put clients first. Furthermore, high screening paired with high treatment rates improves overall point of care services and health outcomes for pregnant women and their unborn children in a cost‐effective and sustainable manner.

**Conclusions/Next steps: **Other low‐ and‐middle‐income‐countries (LMICs), looking to rollout dual‐tests or new HIV‐testing guidelines, should borrow successes from Liberia's national rollout. Investment in county and district health teams reduce the need for repetitive capacity building at facilities and build upon existing structures to support future HIV‐programmes, ensuring that clients receive high‐quality, comprehensive services. Liberia's ability to achieve significant improvements in syphilis screening and treatment rates demonstrates dual‐testing in ANC settings is feasible and readily adopted by healthcare providers and clients.

### OAC2204 Monitoring Malawi's national integrated testing rates for HIV, syphilis, and hepatitis B and the co‐infection prevalence for pregnant women using routine individual data captured through artificial intelligence technology

T. C. Chirwa ^1,2^, J. Nkhonjera^3^, K. Namachapa^3^

^1^Ministry of Health Malawi, Directorate of HIV, Syphilis and Viral Hepatis, Lilongwe, Guatemala, ^2^International Training and Education Center for Health, Lilongwe, Malawi, ^3^Directorate of HIV, STI and Viral Hepatitis, Lilongwe, Malawi

**Background: **Malawi's 2023 HIV testing programme guidelines have integrated testing for three diseases of HIV, syphilis and hepatitis B virus (HBV) to align with the WHO's triple elimination goal by 2030 among pregnant women. The programme used ScanForm, an innovative tool that captures data from paper registers using Optical Character Recognition (OCR) via a simple smartphone, and further digitizes this information using artificial intelligence (AI). This enhances the granular monitoring of testing rates and co‐infection prevalence for high‐risk groups such as pregnant women. This study aimed to estimate both the testing rates of the three diseases and the co‐infection prevalence rates among pregnant women.

**Methods: **This study analysed testing rates for HIV, syphilis and HBV among pregnant women attending antenatal care (ANC) at 456 out of 657 ANC sites across Malawi, from December 2022 to December 2023. Data were collected via ScanForm, and analysis included computing testing and co‐infection rates, using descriptive statistics and chi‐square tests for age group associations.

**Results: **Among 425,496 women, testing rates were 96.56% for HIV, 81.24% for syphilis and 57.93% for HBV. The highest co‐infection rate (0.2%) was for HIV‐syphilis among 331,683 tested women, particularly in the 25−39 age group. Syphilis‐hepatitis B coinfection was found in 0.1% (298) of 215,897 tested women, mostly in the 30−39 age group. The HIV‐hepatitis B combination had a <1% prevalence, with a higher rate in the 25−39 age group. Triple infections were rare, with only 12 cases found. ​

**Conclusions: **Malawi's integrated testing programme effectively tracks and monitors STI testing and co‐infection rates, crucial for evaluating programme impact and enhancing maternal and child health outcomes. Further analysis on the ScanForm data will focus on multivariate analysis of factors affecting the prevalence of the co‐infections rates.

**Table:** Table. OAC2204

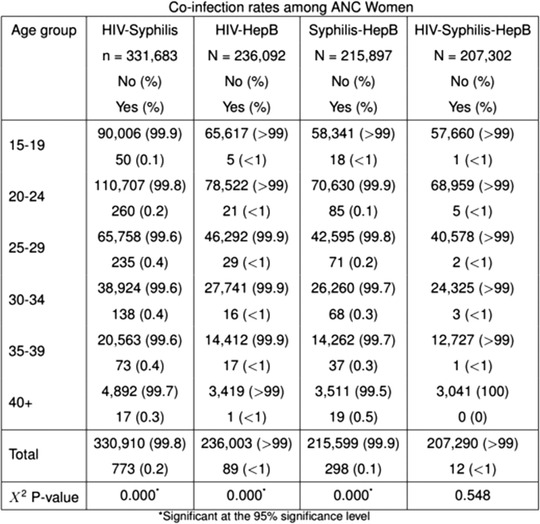

### OAC2205 The impact of intimate partner violence to vertical HIV transmission: A modelling analysis of 42 countries in Sub‐Saharan Africa

S. Kuchukhidze ^1^, M. Walters^2^, D. Panagiotoglou^1^, M.‐C. Boily^2^, S. Diabaté^3,4^, W. A. Russell^1^, H. Stöckl^5^, L. Sardinha^6^, F. Mbofana^7^, R. K. Wanyenze^8^, J. W. Imai‐Eaton^9^, M. Maheu‐Giroux^1^

^1^McGill University, Department of Epidemiology and Biostatistics, Montreal, Canada, ^2^Imperial College London, MRC Centre for Global Infectious Disease Analysis, School of Public Health, London, United Kingdom, ^3^Université Laval, Département de Médecine Sociale et Préventive, Quebec, Canada, ^4^Université Laval, Centre de Recherche du CHU de Québec, Quebecc, Canada, ^5^Ludwig Maximilian University of Munich, Institute for Medical Information Processing, Biometry, and Epidemiology, Medical Faculty, Munich, Germany, ^6^World Health Organization, The UNDP‐UNFPA‐UNICEF‐WHO‐World Bank Special Programme of Research, Development and Research Training in Human Reproduction (HRP), Development and Research Training in Human Reproduction (HRP), Department of Sexual and Reproductive Health and Research, Geneva, Switzerland, ^7^Conselho Nacional de Combate ao HIV/Sida, Maputo, Mozambique, ^8^Makerere University, Department of Disease Control and Environmental Health, School of Public Health, College of Health Sciences, Kampala, Uganda, ^9^Harvard T.H. Chan School of Public Health, Center for Infectious Disease Dynamics, Department of Epidemiology, Boston, United States

**Background: **The 2022 *Global Alliance to End AIDS in Children* highlights addressing gender inequities as key to eliminating mother‐to‐child HIV transmission (MTCT). Women experiencing intimate partner violence (IPV) may be at an increased risk of MTCT due to vulnerability to HIV acquisition and barriers to accessing care. Burden of IPV and new paediatric HIV acquisitions are among the highest in sub‐Saharan Africa. However, the proportion of MTCT attributable to IPV is unknown.

**Methods: **We created a probability tree model for MTCT among women (15−49 years) in 42 sub‐Saharan African countries in 2022. We estimated the proportion of MTCT attributable to past‐year physical and/or sexual IPV, as an age‐standardized population attributable fraction (PAF). We accounted for perinatal and postnatal MTCT among women who acquired HIV before pregnancy, during pregnancy and during breastfeeding. Model parameters included: fertility, HIV prevalence/incidence, ART uptake/retention and breastfeeding duration from UNAIDS’ 2023 Spectrum model; IPV prevalence from the WHO Global Database on Violence Against Women; and effect measures for IPV's impact on model parameters from literature reviews. We derived uncertainty intervals (95% UI) through 1000 Monte Carlo simulations.

**Results: **Across 42 countries, 15% (95% UI: 7−23%) of paediatric HIV acquisitions were attributed to IPV in 2022. PAF ranged from 4% (95% UI: 2−7%) in Niger to 28% (95% UI: 13−42%) in Uganda. PAF was highest among 15‐ to 19‐year‐olds (23%; 95% UI: 9−36%) and lowest among 40‐ to 45‐year‐olds (7%; 95% UI: 4−11%). ART uptake was most correlated with PAF (*R* ^2^ = 0.6; Figure 1). In high ART uptake settings, IPV could lead to a large drop in ART use and subsequent rise in MTCT. Where ART uptake is low, reducing IPV has a smaller impact on preventing MTCT.

**Figure 1:**
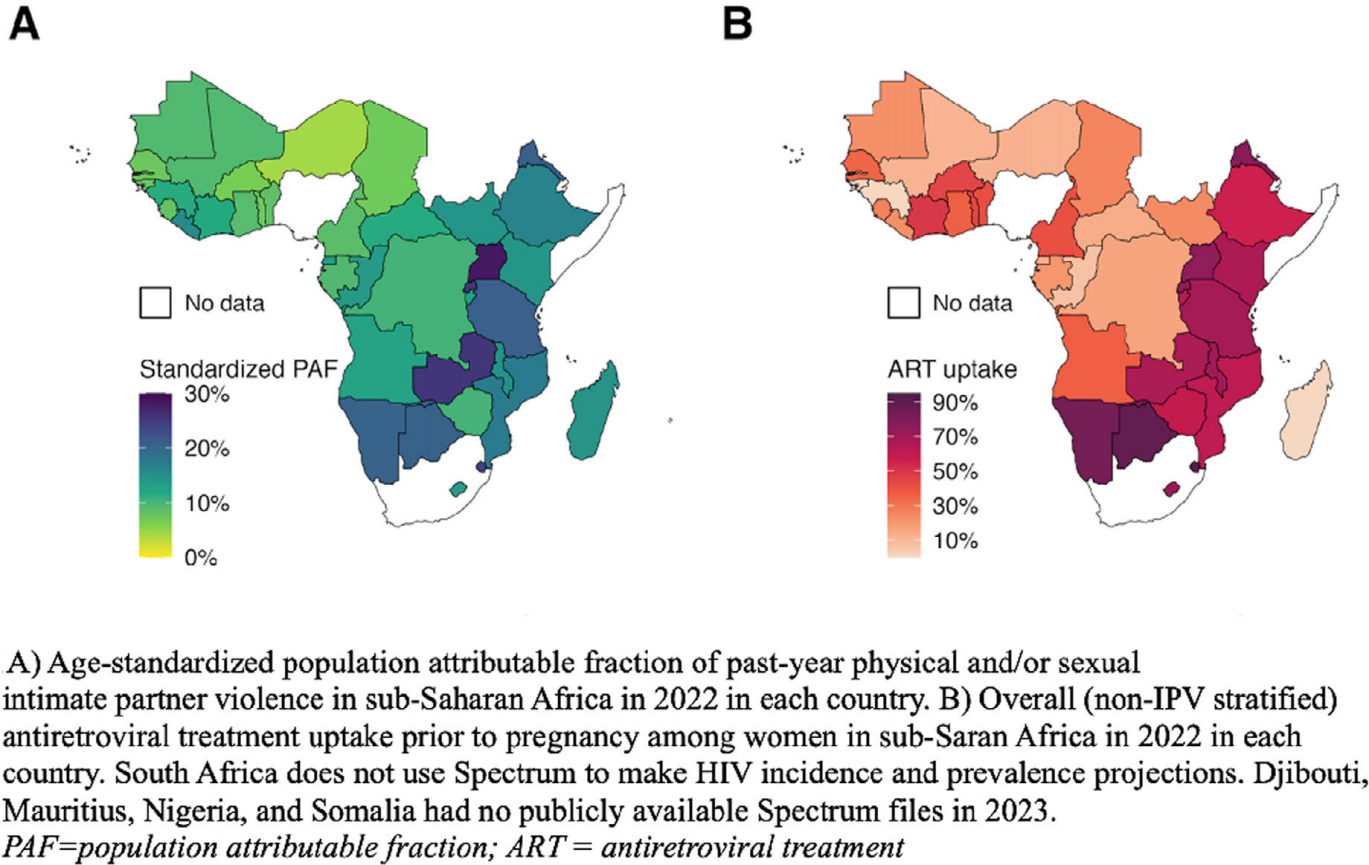
OAC2205

**Conclusions: ​**One in seven new paediatric HIV acquisitions in sub‐Saharan Africa may be due to IPV, rising to one in five among adolescent girls. Ending IPV could accelerate MTCT elimination, especially among young women with highest IPV prevalence and HIV incidence.

### OAC2902 Food insecurity directly impacts adherence to antiretroviral therapy and to pre‐exposure prophylaxis among sexual and gender minorities in Brazil

P. Luz ^1^, T. Torres^1^, V. Matos^1^, B. Hoagland^1^, M. C. Pimenta^1^, M. Benedetti^1^, B. Grinsztejn^1^, V. Veloso^1^

^1^Fundacao Oswaldo Cruz, Rio de Janeiro, Brazil

**Background: **Brazil offers free‐of‐charge antiretroviral therapy (ART) for people living with HIV (PLHIV) as well as pre‐exposure prophylaxis (PrEP) to eligible individuals through its national health system. Adherence to ART and to PrEP is essential to achieving the expected benefits of virologic suppression and prevention of HIV acquisition. We explored whether food insecurity had direct effects on non‐adherence to ART/PrEP.

**Methods: **Cross‐sectional web‐based survey targeting adult sexual and gender minorities living in Brazil (May−September/2021) recruited through dating apps and social media. PLHIV reporting ART use and HIV‐negative individuals reporting daily oral PrEP use were eligible for this analysis. Self‐report of ART adherence was measured by the WebAd‐Q instrument (3‐items/past 7 days) plus a slider question regarding missed doses in the past month (latent outcome). Self‐report of PrEP adherence was measured by the number of days the person took PrEP in the past week (binary outcome: <daily vs. daily). The 7‐item Brazilian Scale of Food Insecurity (EBIA) was used to measure food insecurity (higher scores = more severe food insecurity). We used structural equation modelling to assess the direct and indirect effects of variables on ART/PrEP non‐adherence.

**Results: **In total, 1230 PLHIV were using ART, and 991 HIV‐negative individuals were using daily oral PrEP. Median age of PLHIV was 37 years (HIV negative: 34 years), most were cismen (97%). More PLHIV reported moderate/severe food insecurity 21% (HIV negative: 12%). Self‐report of ART non‐adherence (WebAd‐Q) was 45% (PrEP non‐adherence: 7%). Higher socio‐economic status (latent variable measured by income, education and sex work) had a strong negative effect on food insecurity. Among PLHIV, food insecurity (standardized coefficient [SC]: 0.30, standard error [SE]: 0.07, *p*<0.01), in addition to substance use and binge drinking in the past 6 months, had direct effects on ART non‐adherence. Among HIV‐negative individuals, only food insecurity (SC: 0.31, SE: 0.13, *p* = 0.02) had direct effect on PrEP non‐adherence.

**Conclusions: **Brazil has experienced worsening social inequalities, exacerbated by the COVID‐19 pandemic, leading to increases in food insecurity especially among vulnerable populations. Our findings suggest that providing socio‐economic support could directly help PLHIV by improving their quality of life, vulnerable HIV‐negative individuals by preventing HIV acquisition, and ultimately populations through decreased HIV transmissions.

### OAC2903 The nexus between climate change and HIV/AIDS: a Kenyan perspective

D. Opondo ^1,2^, G. Rutto^1^, S. Arodi^1^, R. Marima^1^

^1^University of Nairobi/USAID Fahari Ya Jamii Project, Infectious Diseases, Nairobi, Kenya, ^2^St. Mary's Mission Hospital, Comprehensive Care Clinic, Nairobi, Kenya

**Background: **Climate change and HIV/AIDS are among the most significant public health challenges in sub‐Saharan Africa in the 21st century. However, there is a limited understanding of the complex relationship between these two challenges, which hinders progress in HIV/AIDS prevention and management within the context of the climate crisis.

**Description: **The framework employed in this systemic review identified five pivotal pathways interlinking climate change and HIV/AIDS, drawing insights from an analysis of 50 studies comprising varied designs, geographical locations and focus areas. The identified pathways included extreme weather events, food insecurity, the spread of infectious diseases, increased migration and pressure on health services, forming the investigation's foundation.

**Lessons learned: **The review's findings provided insights into the dynamics between climate change and HIV/AIDS, with specific regional nuances. Notably, food insecurity emerged as a catalytic factor, intensifying the prevalence of transactional sex with about 60% involving women. Lessons gleaned from regions around Lake Victoria, Makindu, Taita Taveta and Turkana County underscored the vulnerability of established practices among young girls and women who were previously cabbage farmers but are now reliant on handouts to meet their basic needs in the face of climate‐induced disruptions, especially drought.

Moreover, the review highlighted the amplifying effect of extreme weather events on the spread of vector‐borne infectious diseases among individuals living with HIV (PLHIV). Climate‐induced migration in northern Kenya, for example, Turkana, emerged as a destabilizing force, exacerbating economic instability and rendering communities susceptible to transactional sex, sexual violence and exploitation.

Furthermore, the review shed light on the adverse consequences of migration on the accessibility of HIV and sexual health services, as demonstrated by Turkana County with its significant HIV burden. The strain exerted by extreme weather conditions on healthcare systems, including HIV services, acted as a hindrance to the advancement of Universal Health Coverage.

**Conclusions/Next steps: **Collaboration between stakeholders in the fight against HIV/AIDS and the climate emergency is crucial. By deepening our understanding of the complex relationship between climate change and HIV/AIDS, we can develop sustainable strategies and interventions to address these challenges. This knowledge will inform the development of programmes and policies to control the spread of HIV in Kenya.

### OAC2905 Examining community factors associated with interruption in treatment among key population accessing HIV care in Southern Nigeria

B. Edet ^1^, G. Emmanuel^2^, P. Umoh^2^, R. Abang^2^, P. Ameachi^3^, K. Abiye^4^, M. Katbi^4^, O. Bartholomew Boniface^5^

^1^Heartland Alliance Nigeria, Strategic Information Unit, Calabar, Nigeria, ^2^Heartland Alliance Nigeria, Program, Abuja, Nigeria, ^3^Heartland Alliance Nigeria, Strategic Information Unit, Abuja, Nigeria, ^4^United States Agency for International Development, Programs, Abuja, Nigeria, ^5^Heartland Alliance Nigeria, Abuja, Nigeria

**Background: **Interruption in treatment (IIT) has been associated with an increase in new infection and high viral load among key population living with HIV. IIT for these populations can lead to poor health outcomes and increased transmission risk. This study aims to identify community factors that contribute to IIT among KPs in this region.

**Methods: **A mixed‐methods approach was employed, combining quantitative data from KP‐CARE 1 data from medical records to identify rates and patterns of IIT, with qualitative client exit and satisfaction form to explore the experiences and challenges faced by KPs in accessing and adhering to HIV treatment. Data were collected from 14 Heartland Alliance HIV treatment One‐Stop‐Shop across Southern Nigeria.

**Results: **Among the 74,733 clients extracted for this study, 9% of clients were recorded as been interrupted in treatment. Among the clients investigated, 38% were female sex workers, 33% men who have sex with men, 28% people who inject drugs and 1% transgender. The FSW clients had the highest IIT of 51% followed by MSM with 25%, PWID with 23% and transgender with 1%, respectively. Sixty four percent of the clients on IIT were aged 25−39 as at the last ART pickup date with multi‐month dispensing (MMD) of 6 months. Analysis indicates that factors such as transportation barriers (aOR = 0.01), economic hardship (aOR = 0.06), pill burden (aOR = 0.03), self‐stigma (aOR = 0.09) and harassment by enforcement (aOR = 0.09) significantly correlate with higher rates of IIT. Notably, high mobility of KPs from one location to another specifically for FSW community emerged as a critical barrier to consistent treatment.

**Conclusions: **The study highlights the complex interplay of community factors leading to IIT among KPs in Southern Nigeria. Addressing these factors requires multifaceted interventions, including stakeholders engagement, income‐generating programme, stigma reduction programmes, reduction on MMD and continuous providing behaviour change intervention. The findings underscore the need for targeted strategies to support the continuity of HIV care for KPs in the region.

### OAC3302 30 years of advancement and challenges in early HIV diagnosis in Jamaica

D. Perry ^1^, R. Khan‐Francis^2^, S. Beckford Jarrett^3^, J. Lawrence^2^, A. Robb‐Allen^2^, W. McFarland^3^, N. Skyers^2^

^1^South East Regional Health Authority, Kingston, Jamaica, ^2^Ministry of Health and Wellness, Jamaica, Kingston, Jamaica, ^3^University of California San Francisco, San Francisco, United States

**Background: **The first case of HIV in Jamaica was reported in 1982 and antiretrovirals (ARVs) became available at public treatment sites in 2004. This study examines the epidemiology of HIV among persons living with HIV (PLHIV) from 1990 to 2020.

**Methods: **We analysed data from PLHIV first enrolled at HIV treatment sites in Jamaica between January 1990 and December 2020 to identify changes in client demographics, clinical stage and location. The data span three periods: 1990−2003 (P1) pre‐ARV access; 2004−2017 (P2)—public ARV for PLHIV with CD4 <350; and 2018−2020 (P3)—universal test and treat in Jamaica. We used chi‐square and Kruskal‐Wallis H tests to analyse changes across the periods.

**Results: **Twenty‐five thousand nine hundred and fifty‐three PLHIV registered at treatment sites: 731 were in P1, 21,024 in P2 and 4201 in P3. Significant differences emerged in sex distribution (c^2^(4) = 34.18, *p* < 0.001), age (c^2^(6) = 1091.95, *p*< 0.001), area of residence (c^2^(4) = 144.054, *p*<.001) and WHO HIV stage at registration (c^2^(6) = 302.28, *p*< 0.001).

**Conclusions: **The decline in paediatric HIV cases and overall increase in early stage diagnoses are notable public health achievements. However, Jamaica has an ageing cohort of PLHIV entering treatment and targeted testing strategies and behavioural research are needed to reduce HIV transmission and late‐stage diagnoses in older adults.

**Figure**. OAC3302: Changes in (i) age, (ii) sex, (iii) HIV stage at diagnosis and (iv) area of residence among PLHIV enrolling in care in Jamaica, 1990−2020, *N* = 25,953.

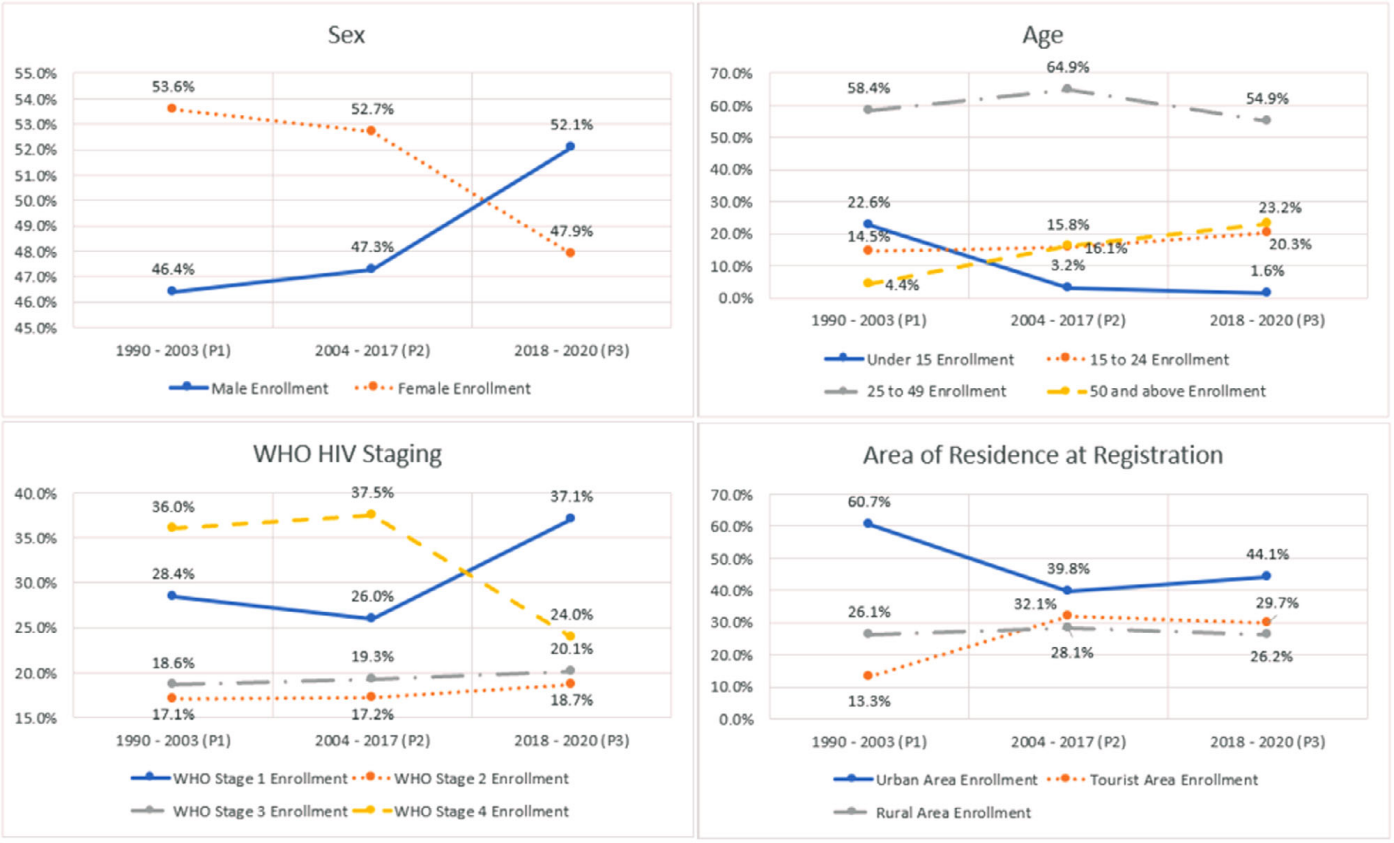

Median age at registration increased from 29 in P1 to 36 in P3; (*H*(2) = 263.68, *p*‐value <0.001). Baseline CD4 counts increased from P1 to P3 (*H*(2) = 343.024; *p*<.001). Stage 4 diagnoses decreased in PLHIV ages under 15, 15−24 and 25−49, but not among PLHIV 50 years and older. Similarly, Stage 1 diagnosis increased across all age groups, except those in the 50 and over cohort.

### OAC3303 The trend for HIV test and treatment and retention on antiretroviral therapy among people living with HIV in Sudan from 2012 to 2020

A. Adam ^1^, A. Mirzazadeh^1,2^, E. Ali^3^, K. Awad^4^, H. Mohamed^4^, M. Elhassan^4^, B. Mugisa^5^, J. Hermez^6^

^1^University of California San Francisco, Institute for Global Health Sciences, San Francisco, United States, ^2^University of California San Francisco, Department of Epidemiology and Biostatistics, San Francisco, United States, ^3^The Joint United Nations Program on HIV/AIDS (UNAIDS), Khartoum, the Sudan, ^4^Federal Ministry of Health, Sudan National HIV Control Program, Khartoum, the Sudan, ^5^United States Agency for International Development, Gauteng, South Africa, ^6^World Health Organization Regional Office for the Eastern Mediterranean, HIV, Hepatitis and STIs, Cairo, Egypt

**Background: **With timely diagnosis of HIV infection, rapid antiretroviral therapy (ART) initiation and good retention on treatment, many countries made significant progress in preventing deaths and further transmission of HIV. We analysed multi‐year data from identified persons living with HIV to assess the timing of diagnosis, ART initiation and retention of treatment in Sudan.

**Methods: **We conducted a retrospective analysis data of 3974 people diagnosed with HIV between 2012 and 2020 in Omdurman ART centre. We reported the distribution of age, sex, stage of disease at diagnosis and geographical locations of residence. We assessed the lag times between HIV diagnosis, ART initiation and last clinical visit, and tested for trends over time.

**Results: **Most people were 30−49 years old (54.9%), male (56.5%), lived in Omdurman (53.8%) and had clinical stage 3 at diagnosis (51.6%) (Table 1). The proportion of people in clinical stage 1 increased from 7.9% in 2012−2014 to 17.4% in 2018−2020 (*p*‐value <0.001). The proportion of people who started ART within 3 months from diagnosis increased from 25.4% in 2012−2014 to 92.3% in 2018−2020 (*p*‐value <0.001). However, among those who started treatment, the proportion who lost to follow‐up after starting ART increased from 55.4% in 2012−2014 to 96.0% in 2018−2020 (*p*‐value <0.001).

**Conclusions: **Our data showed some improvement in early diagnosis of people living with HIV and a significant improvement in timely and rapid ART initiation in Sudan; however, late diagnosis remains predominant. Furthermore, almost all people who started ART had been lost to follow‐up. The late diagnosis and poor retention in treatment threaten losing all the gains made in improving the HIV response in Sudan. Urgent action is needed to remedy those weaknesses.

**Table 1:** OAC3303

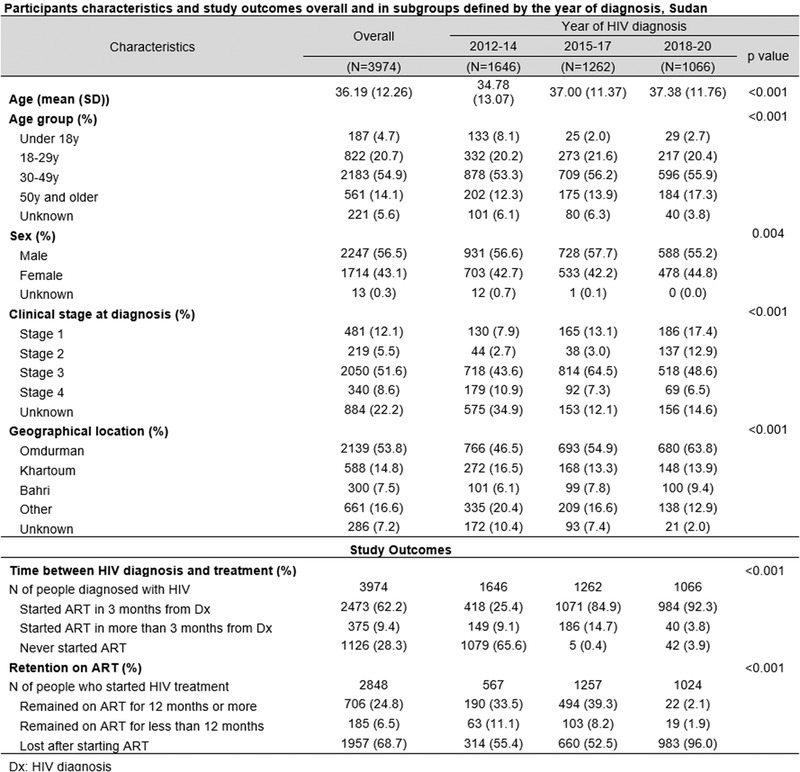

### OAC3304 Changes in early HIV/AIDS mortality rate in people starting antiretroviral treatment between 2013 and 2023: a multicentre survival study in Senegal

B. S. Wembulua ^1^, V. M. P. Cisse^1^, D. Ka^1^, N. F. Ngom^2^, A. Mboup^2^, I. Diao^2^, A. Massaly^3^, C. Sarr^1^, C. S. Ohandza^1^, K. Diallo^4^, M. B. Diallo^1^, M. Diop^5^, N. M. Manga^4^, S. O. Wembonyama^6^, Z. K. Tsongo^6^, M. Seydi^1^

^1^Service des Maladies Infectieuses et Tropicales (SMIT), Fann University Hospital, Dakar, Senegal, ^2^Centre de Traitement Ambulatoire (CTA), Fann University Hospital, Dakar, Senegal, ^3^Pavillon de Traitement Ambulatoire (PTA), Regional Hospital of Kaolack, Kaolack, Senegal, ^4^Department of Infectious Diseases, Hôpital de la Paix, Ziguinchor, Senegal, ^5^Hôpital Principal de Dakar (HPD), Dakar, Senegal, ^6^School of Public Health, University of Goma, Goma, the Democratic Republic of the Congo

**Background: **Studies conducted after the 1996 antiretroviral treatment (ART) access programme in Senegal showed mortality peaks shortly after ART initiation. Considering the national adoption of TATARSEN strategy (Test All, Treat All, and Retain) in 2016 and the scale up of dolutegravir‐based regimens in 2020, we aimed to assess changes in early (6‐month and 1‐year) mortality hazard in Senegalese people starting ART in 2020–2023 and 2017–2019 compared to 2013–2016.

**Methods: **This 10‐year multicentre survival study in Senegal analysed five HIV cohorts from three regions (Dakar, Kaolack and Ziguinchor). Adults (≥18 years) having initiated ART between 2013 and 2023 were considered. Cumulative incidences of death were estimated using the Kalbfleisch‐Prentice method. Loss to follow‐up was accounted as a competing risk. Shared frailty‐based models for competing risk were used to estimate adjusted (for age, sex, WHO Stages and CD4 cell count) early mortality hazard ratios (HRs) in participants who started ART in 2013–2016 (comparator), 2017–2019 and 2020–2023.

**Results: **We enrolled 4006 persons, of whom 2281 (56.9%) were female, and 635 (15.9%) were at WHO‐Stage IV. Median age and CD4 cell count at ART initiation were 40 years (IQR: 31−50) and 188 cells/mm^3^ (IQR: 57−410), respectively. Median follow‐up was 80.4 months (IQR: 48.6−106.7). A total of 463 participants died (4.37 deaths per 100 person‐years), 227 at 6 months and 296 at 1 year, yielding cumulative incidences of 5.7% (95% CI: 5.0−6.4) and 7.4% (95% CI: 6.6−8.2), respectively. Initiation of ART in 2020–2023 was associated with a 38% (adjusted HR [aHR]: 0.62, 95% CI:0.40–0.96) and 40% (aHR: 0.60, 95% CI:0.41–0.48) reduction in 6‐month and 1‐year mortality hazards, respectively, compared to the 2013–2016 period. Predictors of 1‐year mortality were: male sex (aHR: 1.54, 95% CI: 1.20–1.97), age ≥55 years (aHR: 1.98, 95% CI: 1.50–2.60), first CD4 cell count <200 cell/mm^3^ (aHR: 1.45, 95% CI: 1.09–1.93) and WHO Stage IV (aHR: 2.34, 95% CI: 1.77–3.09).

**Conclusions: **Early mortality risk has significantly decreased over time in Senegal. However, AIDS conditions remain significant predictors of mortality. Continued efforts to ensure early diagnosis and prompt linkage to care are needed to further reduce preventable deaths.

### OAC3305 HIV mortality trends and spatial distribution among persons living with HIV, Thailand, 2008–2022

N. Punsuwan ^1^, T. Naiwatanakul^2^, W. Kiatchanon^2^, P. Namahoot^3^, C. Lertpiriyasuwat^1^, S. Aungkulanon^2^, S. Noknoy^1^, P. Wareechai^3^, A. Kanphukiew^2^, S. Northbrook^2^

^1^Division of AIDS and STI, Department of Disease Control, Nonthaburi, Thailand, ^2^Division of Global HIV/TB, U.S. Centers for Disease Control and Prevention, Nonthaburi, Thailand, ^3^National Health Security Office, Bangkok, Thailand

**Background: **Despite notable advancements in healthcare, challenges in health equity remain, specifically in HIV‐related mortality. We compared age‐sex adjusted all‐cause mortality trends among persons living with HIV (PLHIV) registered in the National AIDS Program from 2008 to 2022 in Thailand.

**Methods: **We conducted a retrospective analysis of all reported PLHIV from over 1000 facilities registered in the National AIDS Program (NAP) as of 31 January 2023. Demographics and all‐cause death data were classified according to Thai HIV case surveillance definitions and WHO HIV clinical staging guidelines to calculate age‐sex adjusted mortality rate and standardized mortality ratio (SMR). The average Thai population age and sex structure during 2008−2022 was used as the standard population. Age‐sex adjusted rates were calculated by dividing the number of observed deaths by the population standard and multiplying by 100,000. Standardized mortality ratio was calculated by dividing the number of observed deaths by the number of expected deaths and multiplying by 100. Spatial autocorrelation of SMR was measured by the Moran's *I* coefficient using SAS v 9.4.

**Results: **Of 666,157 diagnosed PLHIV registered in NAP, 157,319 (23.6%) died from all causes. The age‐sex adjusted mortality rate increased from 14.9 per 100,000 in 2008 to 16.8 per 100,000 in 2015 to 18.9 per 100,000 in 2022. Moran's *I* revealed positive values, indicating spatial clustering of high SMR. In 2022, SMR remained high in Bangkok and central provinces near Bangkok, northern Myanmar and Laos border, and eastern seaboard industrial provinces.

**Conclusions: **Increase in age‐sex adjusted mortality rates and geographic disparities in SMR emphasize the need to identify risk factors for death among PLHIV to address gaps. Our findings underscore the importance of ongoing monitoring and a comprehensive approach to address both individual risk factors and regional disparities to reduce mortality among PLHIV in Thailand.

**Figure**. OAC3305

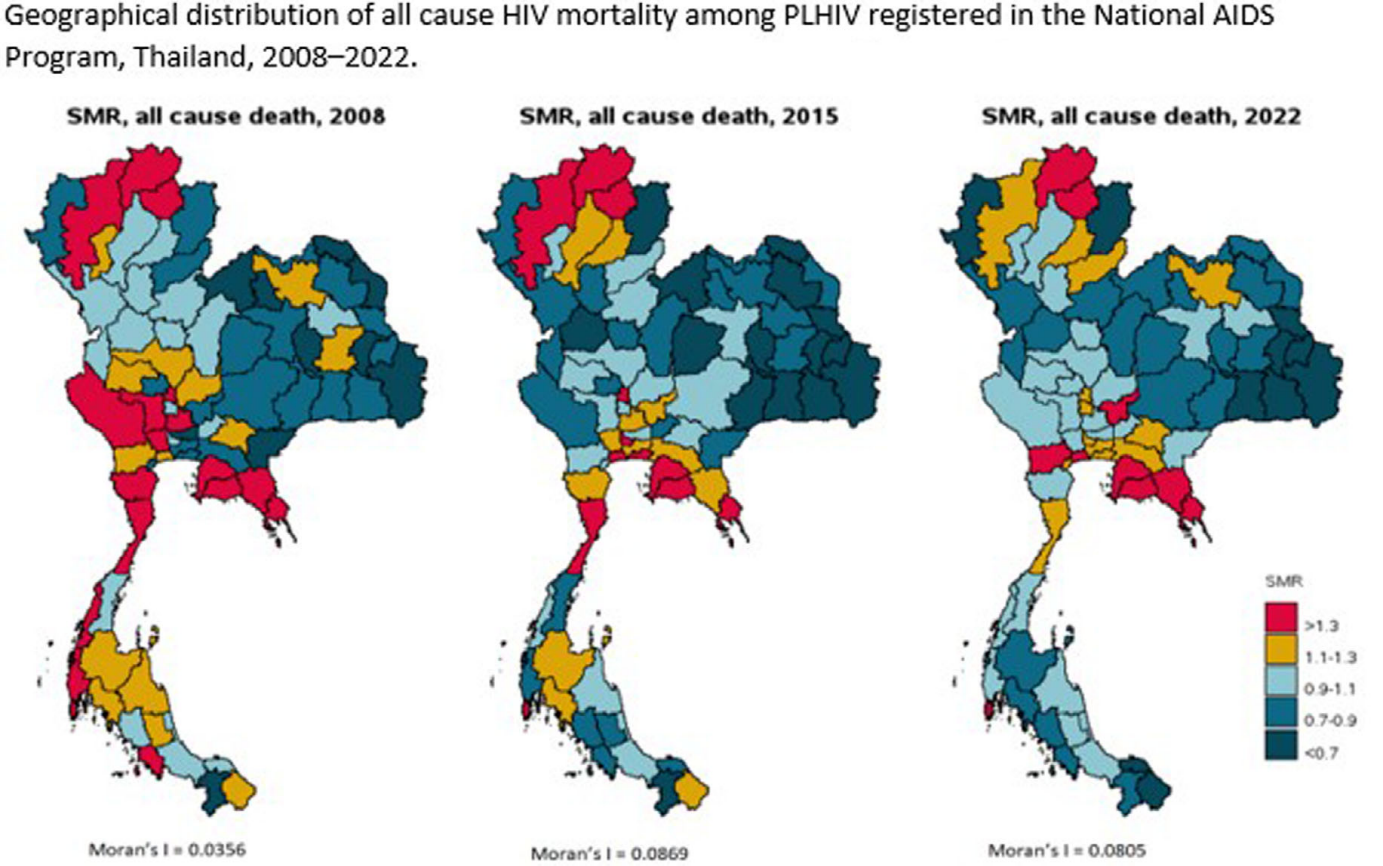

### OAC4002 Transforming cervical cancer screening for women living with HIV using a digital health application: experiences from Tanzania

A. Kibona ^1^, P. Ngalla^2^, J. Mkumbo^1^, F. Haraka^1^, R. Van de Ven^1^, S. Kimambo^1^, S. Yuma^3^, M. Kombe^4^

^1^Elizabeth Glaser Pediatric AIDS Foundation, Technical, Dar es Salaam, the United Republic of Tanzania, ^2^SkyConnect Ink, Program, Dar es Salaam, the United Republic of Tanzania, ^3^Ministry of Health, Technical, Dodoma, the United Republic of Tanzania, ^4^USAID, RMNCH, Dar es Salaam, the United Republic of Tanzania

**Background: **Cervical cancer is a leading cause of cancer morbidity and mortality among women in Tanzania. The World Health Organization recommends visual inspection with acetic acid (VIA) for cervical cancer screening in lower‐resource settings. However, it is a user‐dependent technique vary widely in ascertaining cervical lesions. The Elizabeth Glaser Pediatric AIDS Foundation (EGPAF) rolled out a digital health platform, the smartphone‐enhanced VIA (SEVIA) application, for secured sharing of cervical images, enabling remote supportive supervision of providers and expert verification of diagnosis.

**Methods: **Cross‐sectional analysis of routine programme data from 45 facilities providing cervical cancer screening with SEVIA application services across five supported regions was conducted. Aggregate‐level data were extracted from DHIS2 and the SkyConnect server from April 2022 to October 2023 and were summarized as proportions using Excel software.

**Results: **A total of 33,058 women living with HIV (WLHIV) were screened for cervical cancer; 1057 (3.2%) were found VIA positive. Out of those, 6766 WLHIV (20%) were screened using the SEVIA application and verified by expert. The mismatch of VIA results between the provider and expert decreased from 21% in April−June 2022 to 4% in July−September 2023 (see Figure). Among the 546 mismatch images, 245 images (45%) were reported VIA negative by providers, but as per experts, 176 (72%) were found VIA positive, 24 (10%) were suspected cancer and 45 (18%) had other conditions like polyps and cervicitis. Also, 191 images (35%) were reported VIA positive by providers, but reviewers assessed 185 (97%) of them were VIA negative, four had precancerous lesions and two cervicitis.

**Figure**. OAC4002: Quarterly trend of cervical cancer screening and outcome using the SEVIA App.

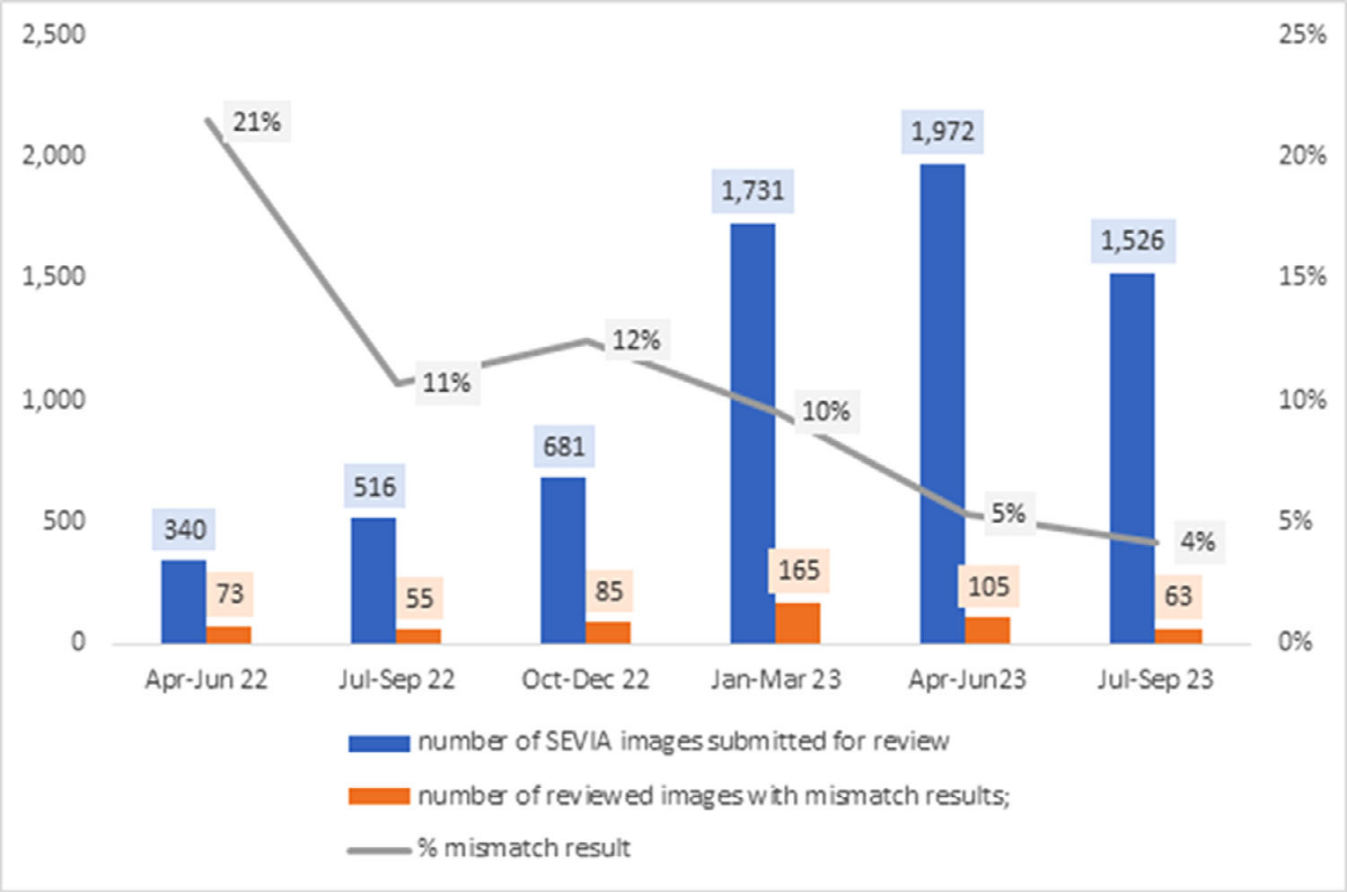

**Conclusions: **The use of digital health platform enabled virtual support to providers, enhancing the quality of cervical cancer screening provided to WLHIV and reducing misdiagnoses. Digital platform will likely improve screening and care of cervical cancer among WLHIV.

### OAC4003 Pilot evaluation of a package of evidence‐based interventions and implementation strategies based on WHO PEN for a cohort of people living with HIV and cardio‐metabolic non‐communicable diseases in Lusaka, Zambia

M. E. Herce^1,2^, J. M. Pry ^1,3^, S. Bosomprah^1,4^, C. Mandyata^1^, M. Siame^1^, C. Mwila^1^, C. Frimpong^1^, A. Mugala^1,5^, P. Sichone^1^, R. Adams^1^, B. Banda^1^, B. Kasenge^1^, L. Chunga^1^, T. Matenga^6^, P. Shankalala^7^, P. Mbewe^1^, N. Nachalwe^1^, M. Vinikoor^8^, J. K. Edwards^9^, W. Mutale^7^

^1^Center for Infectious Disease Research in Zambia (CIDRZ), Implementation Science Unit, Lusaka, Zambia, ^2^University of North Carolina, School of Medicine, Chapel Hill, United States, ^3^University of California Davis, School of Medicine, Sacramento, United States, ^4^University of Ghana, Department of Biostatistics, School of Public Health, Accra, Ghana, ^5^University of Zambia, Ridgeway, Department of Medicine, Division of Infectious Diseases, Lusaka, Zambia, ^6^University of Zambia, Ridgeway, Department of Health Promotion and Education, School of Public Health, Lusaka, Zambia, ^7^University of Zambia, Lusaka, Department of Health Policy and Management, School of Public Health, Lusaka, Zambia, ^8^University of Alabama, School of Medicine, Birmingham, United States, ^9^University of North Carolina, Department of Epidemiology, Chapel Hill, United States

**Background: **With increasing life expectancy for people living with HIV (PLHIV), HIV treatment programmes globally must evolve to address non‐communicable diseases (NCDs). We developed a package of evidence‐based interventions following the WHO package of essential non‐communicable disease (PEN) interventions called “TASKPEN.” TASKPEN includes a one‐stop shop for managing HIV and cardiometabolic NCDs, delivered by a multi‐faceted implementation strategy involving HIV‐NCD service integration, practice facilitation and task‐sharing. We report here the preliminary effects of the “TASKPEN” package on “dual control” of HIV and hypertension for a cohort of PLHIV with co‐morbid cardio‐metabolic NCDs followed prospectively within a pilot stepped‐wedge trial.

**Methods: **PLHIV ≥18 years with a cardiometabolic NCD/risk factor—including hypertension, dyslipidaemia, diabetes mellitus and/or tobacco smoking—identified through a participant screening survey administered at the study clinics from 1 March 2022 to 21 April 2022. For this analysis, we estimated the proportion of participants with suppressed viral load (i.e. <1000 copies/ml) and controlled systolic (<140 mmHg) and diastolic (<90 mmHg) blood pressure before (baseline) and after (follow‐up) TASKPEN introduction using mixed effects regression. Additionally, we described participants’ blood pressure change during the pilot.

**Results: **We enrolled 191 participants, of whom 133 (69.6%) were female, with a median age of 49 years (interquartile range [IQR]: 41−54 years). After a median follow‐up of 231 days (IQR: 221−238 days), the proportion of participants with dual HIV *and* blood pressure control was statistically significant, increasing from 30.9% (95% CI: 24.4%, 38.0%) at baseline to 53.6% (95% confidence interval [CI]: 46.0, 53.6%) at follow‐up (Table). Average systolic and diastolic blood pressure reduced for participants at follow‐up (Figure).

**Figure**. OAC4003

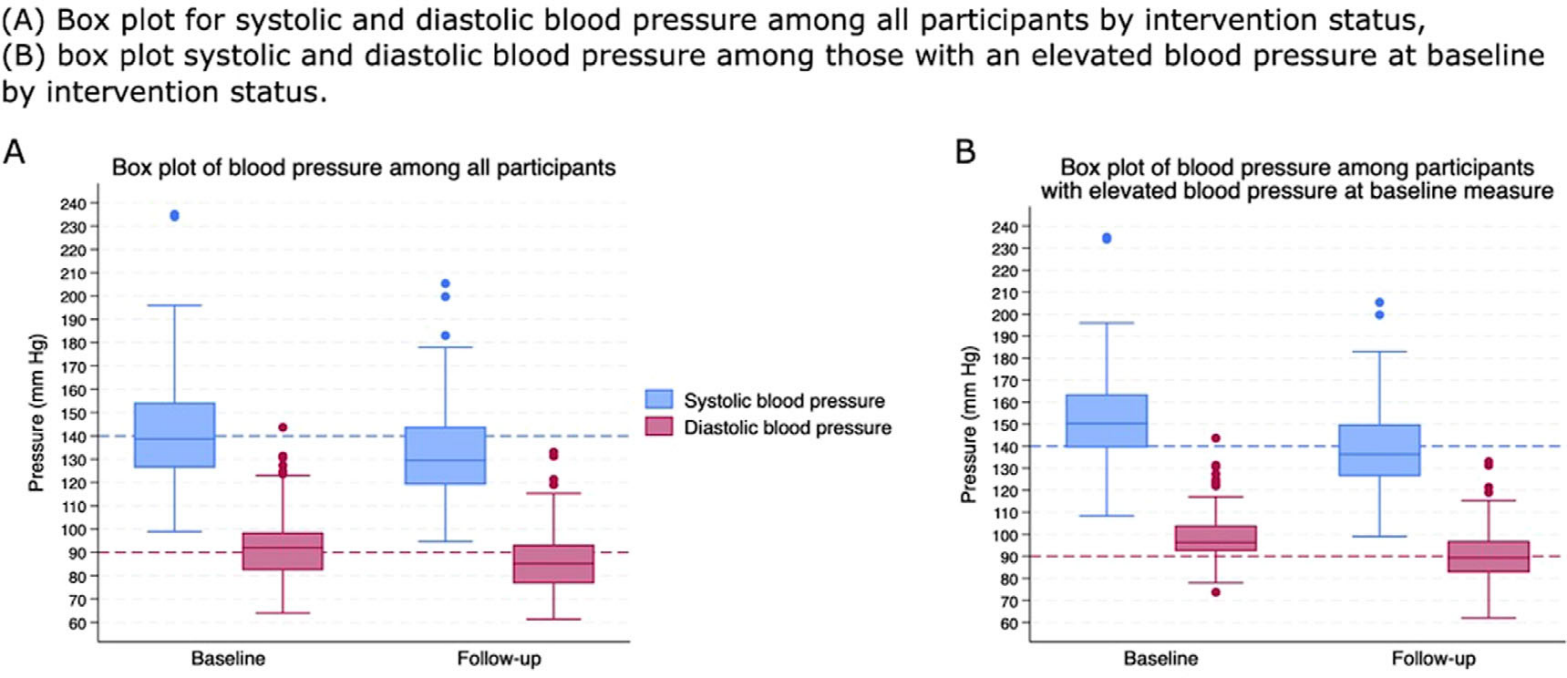

**Table:** Table. OAC4003

	Baseline	Follow‐up
Variable	Proportion (95% CI)	Proportion (95% CI)
Blood pressure [BP] control (i.e. systolic BP <140 mmHg *and* diastolic BP <90 mmHg)	36.1% (29.3, 43.4%)	57.3% (49.7, 64.7%)
Suppressed HIV‐1 viral load (<1000 copies/ml)	84.8% (78.9, 89.6%)	95.3% (91.2, 97.8%)
**Dual control** (i.e. proportion with both viral suppression and BP control)	**30.9% (24.4, 38.0%)**	**53.6% (46.0, 61.6%)**

**Conclusions: **An integrated, WHO‐aligned package of NCDs services can achieve blood pressure control while sustaining, and perhaps improving, viral suppression for PLHIV with co‐morbid cardiometabolic NCDs.

### OAC4004 Persistence of symptoms of depression, anxiety and PTSD among people with HIV aged ≥40 years in low‐ and middle‐income countries in the Sentinel Research Network of IeDEA

M. Stockton ^1^, K. Lancaster^2^, C. Bernard^3,4,5,6^, G. Murenzi^7^, S. Mundhe^8^, J. Mejía‐Castrejón^9^, E. Kwobah^10^, B. Kasimonje^11^, A. Minga^12^, F. Musabyimana^7^, J. Ross^13^, H. Parazzo^14^, H. Byakwaga^15^, F. Mureithi^16^, A. Parcesepe^17^

^1^University of North Carolina at Chapel Hill, Department of Epidemiology, Gillings School of Global Public Health, Chapel Hill, United States, ^2^Wake Forest University, Winston‐Salem, United States, ^3^University of Bordeaux, Bordeaux, France, ^4^National Institute for Health and Medical Research, Bordeaux, France, ^5^Research Institute for Sustainable Development, Bordeaux, France, ^6^Bordeaux Population Health Research Centre, Team GHiGS, Bordeaux, France, ^7^Rwanda Military Hospital and Research for Development, Kigali, Rwanda, ^8^BJ Government Medical College and Sassoon General Hospital, Pune, India, ^9^Instituto Nacional de Ciencias Médicas y Nutrición “Salvador Zubirán”, Departamento de Infectología, Mexico City, Mexico, ^10^Moi Teaching and Referral Hospital, Department of Mental Health, Eldoret, Kenya, ^11^Newlands Clinic, Department of Mental and Social Health, Harare, Zimbabwe, ^12^Centre Médical de Suivi des Donneurs de Sang/ CNTS, Abidjan, Côte d'Ivoire, ^13^The Foundation for AIDS Research, TREAT Asia/amfAR, Bangkok, Thailand, ^14^National Institute of Infectious Diseases Evandro Chagas‐Oswaldo Cruz Foundation, Rio de Janeiro, Brazil, ^15^Mbarara University of Science and Technology, Mbarara, Uganda, ^16^Center for Infectious Disease and Research in Zambia, Lusaka, Zambia, ^17^University of North Carolina at Chapel Hill, Department of Maternal and Child Health, Gillings School of Public Health, Chapel Hill, United States

**Background: **Persistence of symptoms of depression, anxiety and post‐traumatic stress disorder (PTSD) among people with HIV aged >40 years in low‐ and middle‐income countries in the Sentinel Research Network of IeDEA.

**Methods: **We analysed longitudinal data collected between 2020 and 2023 from the International epidemiology Databases to Evaluate AIDS (IeDEA) Sentinel Research Network (SRN) cohort of PWH aged ≥40 on ART at 11 sentinel sites in the Asia‐Pacific, Caribbean, Central and South America, and Central, East, Southern, and West Africa IeDEA regions. We documented the prevalence of symptoms of depression (PHQ‐9 ≥10), anxiety (GAD‐7 ≥10) and PTSD (PCL‐5 ≥33) at enrolment, and 6 and 12 months. Mutually exclusive symptom patterns included: (1) no or mild at all timepoints, (2) persistent or worsening, (3) improved or (4) both worsening and improved.

**Results: **Among 2521 participants, the median age was 51 and 57% were female. The prevalence of depression was 15%, 9% and 9% at enrolment, 6 and 12 months, respectively. For depression, 77% reported no or mild symptoms, 7% reported persistent or worsening symptoms, 11% reported improved symptoms, and 5% reported worsening and improved symptoms. The prevalence of anxiety was 10%, 6% and 7% at enrolment, 6 and 12 months, respectively. For anxiety, 83% of participants reported no or mild symptoms, 6% reported persistent or worsening symptoms, 7% reported improved symptoms and 4% reported worsening and improved symptoms. The prevalence of PTSD was 6%, 4% and 4% at enrolment, 6 and 12 months, respectively. For PTSD, 90% reported no or mild symptoms, 3% reported persistent or worsening symptoms, 5% reported improved symptoms, and 2% reported worsening and improved symptoms. The persistence of symptoms of depression, anxiety and PTSD varied by sex (Table). Females were more likely to report improved symptoms of depression and anxiety.

**Table:** Table. OAC4004: Persistence of symptoms of depression, anxiety and PTSD among PWH in the IeDEA SRN

	Depression			Anxiety			PTSD		
Symptom pattern *N* (%)	Total (*n* = 2330)	Male (*n* = 997)	Female (*n* = 1333)	Total (*n* = 2334)	Male (*n* = 1002)	Female (*n* = 1332)	Total (*n* = 2336)	Male (*n* = 1002)	Female (*n* = 1334)
None or mild	1786 (77)	794 (80)	992 (74)	1937 (83)	838 (84)	1099 (82)	2095 (90)	897 (89)	1198 (90)
Persistent or worsening	172 (7)	83 (8)	89 (7)	140 (6)	62 (6)	78 (6)	69 (3)	35 (4)	34 (2)
Improved	252 (11)	71 (7)	181 (14)	160 (7)	57 (6)	103 (8)	118 (5)	46 (5)	72 (5)
Worsening and improved	120 (5)	49 (5)	71 (5)	97 (4)	45 (4)	52 (4)	54 (2)	24 (2)	30 (2)

**Conclusions: **Symptoms of depression, anxiety and PTSD were common among this cohort of PWH aged ≥40. Future research should examine pathways to improve mental health symptoms and differences by sex.

### OAC4005 Unmet need for HPV vaccination among men who have sex with men living with and without HIV in San Francisco, 2023

P. L. Ramirez ^1^, A. A. Moscatelli^1^, B. Suprasert^2^, M. Tate^2^, M. A. d. S. M. Veras^3^, E. C. Wilson^2,4^, W. McFarland^2,4^

^1^Faculdade de Ciências Médicas da Santa Casa de São Paulo, São Paulo, Brazil, ^2^Center for Public Health Research, San Francisco, Department of Public Health, San Francisco, United States, ^3^Faculdade de Ciências Médicas da Santa Casa de São Paulo, Departamento de Saúde Coletiva, São Paulo, Brazil, ^4^University of California San Francisco, Department of Epidemiology and Biostatistics, San Francisco, United States

**Background: **Men who have sex with men (MSM) are at higher risk for HPV and resulting anal cancer than non‐MSM, with rates highest among older MSM and those living with HIV. Unfortunately, guidelines for adult HPV vaccination are unclear. The objective of our study was to measure the history of HPV vaccination in a community sample of MSM in 2023 in San Francisco, USA.

**Methods: **Data originate from the CDC‐led National HIV Behavioral Surveillance study. Participants are recruited by time‐location sampling wherein participants are intercepted at venues where MSM congregate during randomly selected dates and times. Face‐to‐face interviews collect demographic characteristics, sexual behaviours, sexual health history, access to healthcare and HIV status.

**Results: **Of 497 MSM respondents, 44.9% reported a history of HPV vaccination. The average age of the first HPV vaccine was 29 years (Median = 26, SD = 13). Disparities of HPV vaccination were found by age group (74.2% for 18‐ to 29‐year‐olds vs. 22.6% for 50+ year‐olds, *p*<0.001), educational level (51.0% for postgraduate level vs. 34.3% for some college, *p* = 0.012), seeing a healthcare provider in the past 12 months (47.4% vs. 20.0% among those not seeing a provider, *p*<0.001), having disclosed they have sex with men to their provider (46.2% vs. 23.8%, *p* = 0.047), gender identity (68.8% non‐binary vs. 42.9% for cis‐male vs. 62.5% for or transmasculine, *p* = 0.009) and consistent condom use with anal intercourse in the past 12 months (34.6% consistent vs. 48.8% inconsistent, *p* = 0.005). MSM who used PrEP were more likely to be vaccinated (63% vs. 21.5% not using *p*<0.005). Among MSM living with HIV, the majority (56.3%) were unvaccinated.

**Conclusions: **Despite high risk for HPV and resulting anal cancer, fewer than half of MSM in San Francisco were vaccinated, even among MSM living with HIV. To close the gap, we recommend healthcare providers verify past vaccination history and offer HPV vaccination to unvaccinated men, particularly those who disclose being MSM, who are older and not vaccinated as children, and those who are living with HIV. US and worldwide HPV vaccination policies that specifically address MSM are needed, such as recommending vaccination for MSM at any age.

### OAD0502 AIDS‐orphaned or COVID‐orphaned? Mental health and suicidality across two pandemics

L. Sherr ^1^, T. Mawoyo^2^, R. Hisham^3^, C. Laurenzi^2^, K. Steventon Roberts^4^, M. Tomlinson^2^, L. Cluver^3^

^1^University College London, Global Health, London, United Kingdom, ^2^Stellenbosch University, Institute for Life Course Health Research, Stellenbosch, South Africa, ^3^Oxford University and University of Cape Town, Department of Social Policy and Intervention, Oxford, United Kingdom, ^4^Oxford University, Department of Social Policy and Intervention, Oxford, United Kingdom

**Background: **Parental/caregiver death dramatically affects children directly and indirectly. These are lifelong, and care provided can impact on the future mental health, achievements and behaviour. This study examines the differences and similarities between AIDS orphanhood and COVID orphanhood on children in South Africa with a focus on mental health, suicidal behaviours and psychosocial and environmental risks.

**Methods: **A total of 611 South African children were assessed by trained researchers with ethical approval. Three groups were studied—190 had experience parental/caregiver loss through AIDS, 211 through COVID and a comparison group (*n* = 210) with no loss. Data gathered included demographic variables, mental health, suicidality (mini mental health examination), behaviour, risk behaviours in relation to HIV acquisition, alcohol use, violence, parenting and stigma. Validated scales and established cut‐off points were used for the analysis including analysis of variance and multivariate regression modelling as appropriate.

**Results: **Comparisons between groups showed similar levels of exposure to poverty and turbulent home environments. Orphanhood from HIV/AIDS was most likely to be stigmatized and had the highest levels of suicidality. One in 10 children wished they were dead, with AIDS orphanhood five times and COVID orphanhood three times the control group (16.8%, 9%, 3.8% *p*<.001). This pattern held true for self‐harm (12.6%, 9.5%, 3.6% *p* = .006), contemplation of suicide (10.5%, 9%, 4.3% *p* = .05) and suicide attempts (9%, 2.4%, 1.4% *p* = .001). The AIDS affected children were most vulnerable to negative developmental outcomes, but were followed closely by COVID orphanhood children. Sexual risks, however, were more pronounced among the COVID orphanhood group. Multivariate regression results support the existence of a link between poverty, parenting quality, mental health and social risk behaviours as a pathway to cumulative disadvantage. Increasing age of the child was positively associated with every mental health measure.

**Conclusions: **The mental health burden of orphanhood is high and persists as children age. Little appears to have been learned from the AIDS‐pandemic to inform the COVID‐pandemic which is following closely in terms of mental health burden, stigma, poverty, violence and social risk. The COVID pandemic may have overshadowed the AIDS pandemic with attention and funding diversions, despite a high level of ongoing support needed and little integrated vision.

### OAD0503 Adapting and evaluating a mindfulness and acceptance‐based mental health support programme for adolescents with HIV in a resource‐constrained setting. Evidence from Uganda

K. Musanje ^1,2^, C. Camlin^3^, R. Kasujja^4^, M. Kamya^2^

^1^Makerere University, Psychology, Kampala, Uganda, ^2^Makerere University, Medicine, Kampala, Uganda, ^3^University of California San Francisco, Obstetrics, Gynecology and Reproductive Science, San Francisco, United States, ^4^Makerere, Psychology, Kampala, Uganda

**Background: **Adolescents with HIV (AWH) report the highest mental health burden in Uganda. The dual challenge of living with HIV and negotiating life stage changes compromises AWH's mental health and related clinical outcomes. While several psychosocial strategies are used with AWH, the persistent mental health gap warrants alternative and innovative approaches to complement existing care services. Mindfulness and Acceptance‐based Interventions (MABI) show promise when used to support the mental health of young people. However, their effectiveness has only been tested in high‐income contexts and not with adolescents living with a stigmatized condition such as HIV. We aimed to adapt and test effectiveness of a MABI as an alternative mental health support strategy for AWH in Uganda.

**Methods: **Following two adaptation frameworks (the ecological validity model and the formative method for adapting psychotherapy), we engaged *n* = 30 stakeholders involved in the HIV care cascade to culturally translate a MABI for use in Uganda. Through an open‐label randomized trial (NCT05010317), we further tested the effectiveness of the adapted MABI in reducing depression symptoms, internalized AIDS stigma and anxiety among a sample of 122 older AWH (15−19 years) recruited from an urban clinic in Kampala and used paired sample *t*‐tests and multiple linear regression to compare groups pre‐post.

**Results: **Key adaptations to MABI included: simplifying the language of the manual, adding local practices and slang into therapy, introducing racially congruent visuals and cards representing emotions and reducing therapy sessions from six to four. Furthermore, preliminary results showed that the intervention was associated with a reduction in symptoms of depression (pre‐test = 21.64; post‐test = 12.07, *p* = <0.001), anxiety (pre‐test = 35.47; post‐test = 27.41, *p* = <0.001) and stigma (pre‐test = 3.09; post‐test = 2.07, *p* = 0.002).

**Conclusions: **Results suggest that MABIs have the potential to improve the mental health of AWH in low‐resource settings. Large‐scale trials building on these preliminary results are needed to generate additional evidence to support advocacy inclusion of MABIs into care for adolescents with HIV.

### OAD0504 Nishikilie (Hold on me): providing responsive community centred mental health interventions for LGBTQI communities in anti‐LGBTIQ polarized contexts

S. Mumba ^1^, R. N. Josphine^2,3^, J. Kyana^1^, K. M. Wambua^1^

^1^HAPA Kenya, Programmes, Mombasa, Kenya, ^2^HAPA Kenya, Technical Consultant—Programmes, Mombasa, Kenya, ^3^Community Health Advocacy and Learning Initiative, Public Health Consultant, Kilifi, Kenya

**Background: **HAPA‐Kenya, a key population‐led organization in Mombasa, offers comprehensive health services to 3022 participants, including 140 PLHIV, through community outreach, support groups, drop‐in centres and advocacy. Evidence shows that pervasive violence, stigma and discrimination impact key populations’ mental health, hindering healthcare access. In 2023, the LGBTIQ community faced cycles of anti‐LGBTIQ campaigns, violence and security polarization, exacerbating these challenges.

**Description: **In 2023, a surge in anti‐LGBTIQ campaigns and violence prompted an increased demand for mental healthcare. To address this, a programme was implemented, focusing on mental health screening, psychoeducation, life skills building and group therapy referral. Capacity building included training peer educators and healthcare providers in basic counselling skills and integrated mental health screening. Weekly online check‐ins, a monthly alcohol and substance recovery support group, and KPLHIV support groups were introduced to meet the high demand. The programme emphasized referrals and linkage to psychiatric and social services, with monthly peer educator supervision, microplanning and data review.

**Lessons learned: **In 2023, amid heightened mental healthcare demand due to community‐wide traumatic events, 2010 LGBTIQ individuals engaged in therapist‐guided online check‐ins. Mental health screening of 3094 MSM and transgender persons identified 491 with needs, including 140 PLHIV. Notably, 28% received psychological first aid, 43% underwent individual therapy and 68% participated in group therapy. Ten individuals were referred to Port Riez Hospital in Mombasa for psychiatric treatment, addressing issues like depression (six cases) and psychosis (four cases). This proactive approach, responding to a myriad of challenges, showcases the programme's dedication to supporting diverse mental health needs within the community.

**Conclusions/Next steps: **Anti‐LGBTIQ campaigns and violence contribute to a high prevalence of mental health issues in key populations. This emphasizes the urgency to integrate mental health services into key population programmes, with a focus on community‐level screening, healthcare worker training in CETA and the expansion of online support services.

**Figure**. OAD0504

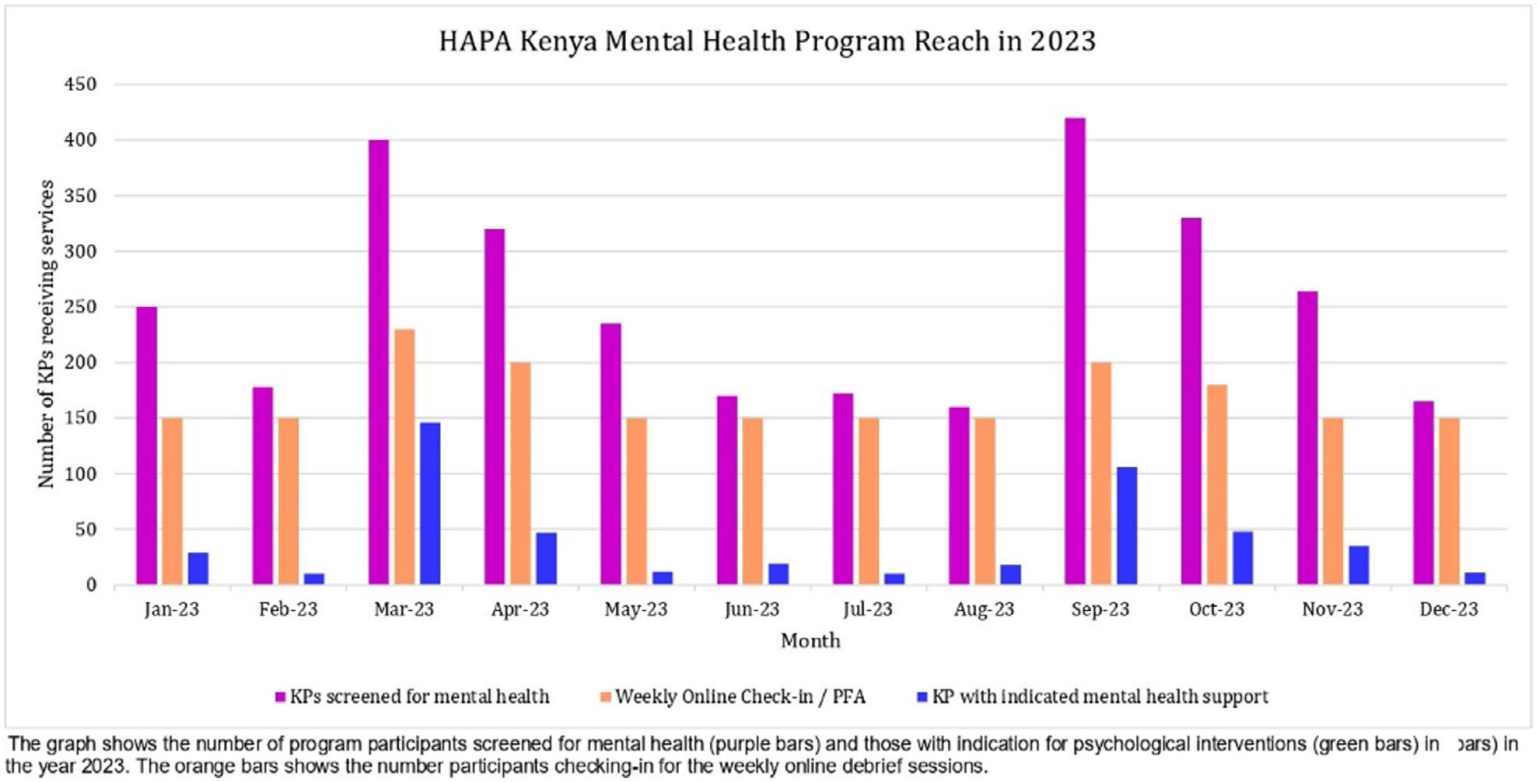

### OAD0505 Peer‐led interventions: exploring the peer group leader experience of delivering a group‐based mental health intervention for adolescents living with HIV in Tanzania

C. Agina ^1^, D. Dow^1,2^, F. Nasuwa^2^, J. Mosha^3^, N. Abdul^3^, E. Sanga^3^, L. Samson^4^, L. Amos Ndelwa^5^, J. Noel Baumgartner^6^

^1^Duke University, Global Health Institute, Durham, United States, ^2^Kilimanjaro Christian Medical Centre, Moshi, the United Republic of Tanzania, ^3^National Institute of Medical Research, Mwanza Research Centre, Mwanza, the United Republic of Tanzania, ^4^Ifakara Health Institute, Ifakara, the United Republic of Tanzania, ^5^Mbeya Zonal Referral Hospital, Mbeya, the United Republic of Tanzania, ^6^University of North Carolina at Chapel Hill, School of Social Work, Chapel Hill, United States

**Background: **Adolescents living with HIV (ALWH) face mental health challenges which negatively influence their adherence to antiretroviral medication and HIV outcomes. In Africa, where the majority of ALWH reside, there are few mental health professionals. Task‐sharing to lay peer leaders may be an effective strategy for delivering mental healthcare. A peer‐led, group‐based mental health intervention called *Sauti ya Vijana* (SYV) was found to be feasible and acceptable to ALWH in Tanzania. This study aims to understand and evaluate peer group leaders’ experiences with SYV.

**Methods: **Twenty‐five peer group leaders (PGLs) aged 23−29 years and living with HIV were trained to deliver SYV which includes 10 group‐based sessions (2 with caregivers) and 2 individual sessions. SYV incorporates three evidence‐based components: cognitive‐behavioural therapy, interpersonal therapy and motivational interviewing, to discuss coping, relationships, stigma, disclosure and value‐guided goal pursuit. IDIs were conducted with PGLs after training and their experience delivering SYV in the pilot study leading into a larger, ongoing clinical trial. IDIs were audio‐recorded, translated from Swahili to English, and analysed using NVivo and 28 deductive codes. Excel was used to summarize and display data for the US‐Tanzania team‐based qualitative data interpretation and identification of themes. Results were presented back to participants for input.

**Results: **PGLs reported a range of motivators and perceived benefits, including a desire to help youth, increased confidence, a sense of shared benefit with the youth and newfound hope for the future. Challenges included concerns about compensation, navigating exposure to difficult life events from the youth that trigger past trauma experience by PGL, maintaining boundaries with the youth and a need for more in‐person supervision. PGLs expressed concerns about job security, particularly around ageing out of the peer role. Recommendations for intervention expansion and sustainability included defining key qualities of future PGLs, continuous training, opportunities for career growth, and integrating male and female youth during sessions.

**Conclusions: **Taking the factors mentioned by PGL into consideration can help enhance the SYV PGL experience and position SYV for sustainability as Tanzania navigates scaling mental healthcare for ALWH.

### OAD0702 Piloting online services for people using new psychoactive substances to assess the prevalence of HIV

C. Imankulova ^1^, N. Shumskaia^2^, N. Kemelbek kyzy^2^

^1^Public Foundation “AFEW”, Program Management, Bishkek, Kyrgyzstan, ^2^Public Foundation “AFEW”, Bishkek, Kyrgyzstan

**Background: **People who use new psychoactive substances (NPS users) are at a significant risk of HIV transmission due to injection and unsafe sex practices. Despite this, harm reduction in Kyrgyzstan primarily targets opioid users, neglecting the diverse groups using NPS. In 2023, a pilot project using the Web‐outreach model has provided services to NPS users, encouraging self‐testing for HIV and serving as a bridge to the HIV centre upon positive results.

**Description: **Operating in high‐drug‐use regions (Bishkek, Osh, Chui), the project studied existing online engagement approaches, trained web‐outreach workers and developed a web‐outreach guide. We reached 1754 NPS users (PWUD 78%, PWID 6%, MSM 11%, TG 2%, SW 3%). Among 6% PWID, 46% used also heroin (72% using sterile equipment). Hundred percent were tested for HIV (27% self‐testing, 73% assisted testing) and received harm reduction kit. Five HIV cases were identified, three confirmed and started ARV treatment. Sixty eight percent of NPS users stated that their sexual activity increased after the use of NPS, 50.5% of beneficiaries had three or more sexual partners in the past month. Twenty five percent used condom during the last sexual encounter.

**Lessons learned: **Of all beneficiaries, 37% were reached online because of their fear and distrust. Therefore, it is important to combine online and offline outreach. NPS users as web‐outreachers are vital for building trust. Continuous training is essential, given different dynamics in offline and online service provision, requiring skills in communication technologies. Consistent input is essential due to the high rotation/attrition rate among peer web‐outreachers. The low detection rate (0.02%) may be an indicator that HIV is just beginning to spread in this group.

**Conclusions/Next steps: **Interim results demonstrate sexual and injection drug use. Without sustained interventions for NPS users, there may be an increase in HIV. The growing interest in NPS among key populations, as well as the shift to injection use of NPS, underscores the urgency of continued interventions to mitigate the potential public health impact.

### OAD0703 “Bluetoothing”: Knowledge, attitudes and behaviour on unsafe drug injection behaviour and its risks to HIV transmission among adolescents and young people in Zimbabwe

H. Choi ^1^, M. Munjoma^1^, N. Kunaka^1^, N. Nhando^1^, K. Chatora^1^, J. Mavudze^1^, T. Moga^1^, B. Mutede^1^, N. Taruberekera^1^

^1^Population Solutions for Health, Harare, Zimbabwe

**Background: **Drug and substance abuse (DSA) is one of the most pressing public health issues in young Zimbabwe populations, and 57% of youths were reported to engage in substance use in 2019. Within various DSA patterns, injection accounts for the second riskiest behaviour for HIV acquisition globally. As DSA becomes more prevalent in high HIV‐burden communities like Zimbabwe, it is important to understand contexts around injection drug use and HIV transmission among adolescents and young people (AYP).

**Methods: **We employed a mixed‐method study in two metropolitan provinces in Zimbabwe from February to March 2023. We administered a questionnaire to randomly selected AYP and 24 in‐depth interviews with purposively selected community‐ and national‐level key stakeholders to assess their knowledge, attitude, and behaviours on adolescent DSA and subsequent HIV risks. We collected quantitative data with KoBo Toolbox and analysed using SPSS Statistics, and we utilized an inductive approach and thematic coding for qualitative analysis.

**Results: **We recruited 770 AYP (410 male, 358 female and 2 transgender) for the survey. 50.5% of males and 36.6% of females responded that they had engaged in DSA within the past 3 months. 3.8% of them had previously used injection as method, and all of them indicated the experience of sharing unsterilized injection equipment with others. Only 26.2% and 10.1% of AYP who use drugs had knowledge of HIV transmission risk through sharing and using non‐sterilized injection equipment, respectively, whereas 97.6% were aware of the risk of unprotected sex. From IDIs, we identified a rise of a new drug injection behaviour called “bluetoothing” among AYP, a direct person‐to‐person injection of blood drawn from an individual who is already intoxicated, mainly due to a lack of financial resources to purchase safe injection supplies and substances.

**Conclusions: **Findings show an intricate dynamic between DSA and potential HIV transmission through a new unsafe drug injection behaviour in Zimbabwe. Lack of resources and attention towards “bluetoothing” facilitates AYP's easier access to substance use and increases their chances of HIV acquisition. As we move to the status neutral approach, it is necessary to develop targeted solutions for unsafe injection behaviours to prevent the transmission among AYP.

### OAD0704 Synergistic effects of exposure to multiple types of violence on non‐fatal drug overdose among women who inject drugs in Indonesia: Implications for broadening the scope of harm reduction services

C. Stoicescu ^1^, B. Medley^2^, E. Wu^2^, N. El‐Bassel^2^, P. Tanjung^3^, L. Gilbert^2^

^1^Monash University, Indonesia, Public Health and Preventive Medicine, Jakarta, Indonesia, ^2^Columbia University, School of Social Work, New York, United States, ^3^Women and Harm Reduction International Network, Jakarta, Indonesia

**Background: **While research has demonstrated associations between violence from intimate and non‐intimate partners and drug overdose among women who inject drugs, existing research focuses predominantly on the Global North and is methodologically limited. Guided by syndemics theory and the risk environment framework, this study examined whether different experiences of gender‐based violence exert independent and interactive effects on non‐fatal drug overdose among women who inject drugs in Indonesia. Findings have implications for broadening the scope and gender‐responsiveness of harm reduction services in Indonesia, which are presently HIV‐focused, to address violence‐ and overdose‐related harms.

**Methods: **We recruited 731 cisgender adult women who injected drugs in the preceding year via respondent‐driven sampling. Multivariate logistic regressions examined associations between intimate partner violence (IPV), police sexual violence and police bribery, and non‐fatal drug overdose, with covariance adjustment for theory‐informed demographic and social factors. To assess whether victimization with multiple types of violence exerts a synergistic effect on overdose, we tested for interaction effects among violence measures by calculating metrics for attributable proportion, relative excess risk due to interaction and synergy index.

**Results: **Lifetime prevalence of non‐fatal overdose was 31.4% (95% CI 27.6, 35.5). Experiencing IPV (AOR 2.38; 95% CI 1.17, 4.83; *p* = 0.016), police bribery (AOR 2.12; 95% CI 1.45, 3.10; *p*≤0.001) and police sexual violence (AOR 3.70; 95% CI 1.47, 9.28; *p* = 0.005) each independently predicted overdose. A significant positive interaction on the additive scale was detected between IPV and police sexual violence on overdose (AP = 0.64, *p* = 0.001; S = 3.76, *p* = 0.015), such that these factors’ joint effect was associated with a fourfold increase in overdose risk.

**Conclusions: **This is the first study to show that women who inject drugs exposed to IPV, police sexual violence and police bribery are significantly more likely to experience overdose, with concurrent experiences of IPV and police sexual violence exerting an amplifying effect on overdose beyond the additive effects of each exposure. Results suggest that eliminating one form of violence when multiple forms of violence are present could magnify the expected reduction in overdose. Expanding HIV‐focused harm reduction services in Indonesia to address concurrent violence and overdose would support more comprehensive and gender‐responsive harm reduction provision.

### OAD0705 Transforming risky behaviours among people who use drugs: a blend of community sexual and reproductive health and harm reduction interventions in Dominican Republic

A. Martin ^1^, A. Arciniegas^2^, M. E. Carbuccia^1^, L. Balham^2,3^, J. C. Jones^2,3^, R. Zeriouh^2^

^1^Centro de Orientación e Investigación Integral (COIN), Santo Domingo, the Dominican Republic, ^2^AIDES, Paris, France, ^3^Coalition Plus, Community‐based Research Laboratory, Pantin, France

**Background: **The PRINCIPE project was implemented from 2020 to 2023 in Haiti and Dominican Republic with the aim of strengthening innovative, community‐based HIV services tailored to the needs of key populations. In the Dominican Republic, the focus was on combined prevention and harm reduction interventions integrated into a sexual health offering for people who use drugs (PWUD).

PWUD in the Dominican Republic have a higher HIV prevalence than the general population (3.2% vs. 0.9 %). The lack of harm reduction interventions, discrimination, violence and criminalization put PWUD at higher risk of contracting HIV.

**Description: **The project featured two main strategies. A comprehensive community approach and the empowerment of PWUD through the strengthening of their activism via a PWUD‐led group called Resiliencia Comunitaria (RC). Outreach was conducted by peer educators (PEs). Each PE was assigned an intervention area to find PWUD, established a mutual trust relationship, and accompanied them in risk reduction by providing education, harm reduction (HR) kits and inviting them to RC.

Health services, including medical, nursing, psychological services, HIV and hepatitis C testing, were provided through a mobile unit positioned near sales or consumption areas. People who tested positive or required additional services were referred to the organization's clinic, onsite and offered accompaniment by a PE.

**Lessons learned: **Over 600 PWUDs were reached. Using PE for delivering HR and sexual, and reproductive health services to the communities proved effective in promoting behaviour change. Sixty two percent of PWUD reported that they had changed their behaviour to reduce the risk of acquiring HIV. Forty two percent reduced the frequency of sharing consumption equipment. Additionally, 57% increased the frequency of condom use, and 71% improved their access to health services. Moreover, the empowerment and advocacy process led by RC enhanced PE's effectiveness in outreach, and authorities increased receptiveness to harm reduction interventions.

**Conclusions/Next steps: **Harm reduction services are more effective when paired with empowerment initiatives, further support to RC and the scale‐up of harm reduction are needed. Also, significant gaps were identified in access to tuberculosis services for crack users. Tailored community strategies implemented through a Civil Society Organization‐Government alliance are required to address and tackle this problem.

### OAD1402 Using smartphones and GPS technology in public health and HIV/AIDS research for young men who have sex with men residing in small cities and towns in the United States

B. P. Takenaka ^1,2^, F. J. Griffith^1,2^, R. Barbour^2^, S. Kirklewski^1,2^, C. Tengatenga^3^, E. Nicholson^1,2^, C. K. Lauckner^4^, J. Gibbs^5^, N. B. Hansen^6^, T. Kershaw^1,2^

^1^Yale School of Public Health, Social and Behavioral Sciences, New Haven, United States, ^2^Center for Interdisciplinary Research on AIDS (CIRA), New Haven, United States, ^3^University of Connecticut, School of Medicine, Farmington, United States, ^4^University of Kentucky, College of Medicine, Lexington, United States, ^5^University of Georgia, School of Social Work, Athens, United States, ^6^University of Georgia, College of Public Health, Health Promotion & Behavior, Athens, United States

**Background: **In 2021, 67% (24,107) of HIV incidence in the United States were attributable to male‐to‐male sexual contact, and 39% were among young men. The social‐spatial environments of YMSM shape their HIV risk. Yet, studies primarily use static residential locations on HIV acquisition risk; missing data capturing YMSM's co‐opted social spaces (e.g. bars, parties, clubs). We highlight the innovative opportunities GPS methods and data provide to understanding the social‐spatial influences on YMSM's sexual health.

**Description: **Through the Men's Voices on Mapping, Neighborhoods, and Technology (#MVMNT) study, our app collects individual GPS data continuously, creates heat maps of usage (daily, weekly, monthly levels), movement patterns and facilitates experience sampling (surveys external triggers). To demonstrate the research possibilities, we will show examples of this methodology using our sample of YMSM (18−34) located in non‐metropolitan areas. We used GPS activity space assessments, qualitative interviews and experience sampling to examine influences of social‐spatial environments on HIV‐related behaviours, and to assess areas in need of structural interventions.

**Lessons learned: **From June 2019 to March 2023, 396 YMSM have completed the #MVMNT study. The use of time and place‐based experience sampling provided novel or nuanced insight on MSM's socio‐cultural and environmental interactions. High acceptability, usability, feasibility and overall positive user experiences were shared by YMSM who completed the study. Smartphone usage and participatory mapping of MSM's locations helped examine real‐time objective and subjective neighbourhood experiences, and physical and virtual contexts of YMSM's substance use and sexual behaviours. Our study found high geospatial data validity in trends in YMSM's social and geographic experiences, and these trends shaped HIV risk behaviours. Place‐based sampling ensured real‐time sampling of multiple contextual factors related to substance use and sexual behaviours among YMSM, and risk for HIV acquisition.

**Conclusions/Next steps: **These findings reveal significant gaps in YMSM's interactions within their social‐spatial environments and patterns in sexual behaviours and substance use. Utilizing real‐time data and storytelling provides groundbreaking advantages over previous studies on HIV risk among YMSM. Successful user‐experience supplies critical data for ecological momentary methods utility in future research. Geospatial data can inform needed structural interventions to reduce HIV acquisition risk among YMSM.

### OAD1403 Digitally viral sex‐positive content that targets HIV stigma among MSM in Aotearoa, New Zealand

B. Clotworthy ^1^, R. Olin^2^, C. Butler‐Smith^1^, M. Power^3^, S. Saini^4^, B. Hollingshead^4^, S. Dareth^1^, J. Rich^5^, C. Chalmers^6^, S. Maddox^6^, H. Habgood^6^, J. Spedding^6^, A. Smith^6^, A. Wattie^6^, E. Watkins^6^, K. Bray^6^, M. Bosma^6^, B. Hopkinson^6^, E. Soule^6^, M. Flynn^6^, A. Long^6^, L. Barke^6^, T. Cottle^6^, Burnett Foundation Aotearoa and YoungShand

^1^Burnett Foundation Aotearoa, Social Marketing, Auckland, New Zealand, ^2^Burnett Foundation Aotearoa, Services and Outreach, Auckland, New Zealand, ^3^Burnett Foundation Aotearoa, Marketing, Communications and Fundraising, Auckland, New Zealand, ^4^Burnett Foundation Aotearoa, Policy, Advocacy, and Science, Auckland, New Zealand, ^5^Burnett Foundation Aotearoa, Senior Leadership, Auckland, New Zealand, ^6^YoungShand, Auckland, New Zealand

**Background: **It is common on sexual networking apps (e.g. Grindr) for men who have sex with men (MSM) to use the word “clean” to describe someone's HIV status. However, this perpetuates the stigmatizing idea that people living with HIV are “dirty,” leading to social exclusion and isolation. HIV stigma also creates barriers to accessing testing and treatment, which can lead to late diagnoses and AIDS.

**Description: **Burnett Foundation Aotearoa created a sex‐positive, fun animation and song that allows MSM to recognize that they may be using “clean” to refer to HIV status, perpetuating stigma on HIV. Titled “Don't be a Dick Getting Dick,” the song features colloquialisms and tongue‐in‐check references, with lyrics explaining why “clean” should not be used. Designed for TikTok, the bright and bold animation is a musical style that features in TikTok trends. The content appeared on other digital platforms including YouTube, Instagram, Radio and Podcast platforms, and we used targeted digital advertising placements on Grindr and PornHub. The audience were also directed to our website with resources to change their behaviours. Posters and condom wallets enhanced the content message in physical spaces relevant to MSM.

**Lessons learned: **The campaign was a success with significant reach. Instagram outperformed TikTok with 51,602 plays and a total reach of 39,514. Our targeted digital advertisement on Grindr had 131,227 views, performing well above average industry benchmarks. The virality of the digital performance delivered the content to MSM and wider support networks, ensuring greater reach of anti‐stigma messaging. Community also fed back that they would be reinforcing this messaging with their wider networks. TikTok's strict community guidelines created significant barriers to uploading content, indicating the censorship of sexual health promotion is a challenge to dismantling HIV stigma. Verification on TikTok supported and legitimized our ongoing work on the platform. Instagram earned over 240% engagement compared to our recent content.

**Conclusions/Next steps: **We will continue to create content underpinned by sound public health theory, while being responsive to community need and seeking further opportunities to deliver content through meaningfully involving people living with HIV to ensure that we dismantle HIV stigma in NZ.

### OAD1404 New forms of sex work and prostitution in Senegal: emerging vulnerabilities in the digital age

T. M. Diallo ^1^, N. K. Sow^1^, K. Diop^1^, B. Thiam^2^, S. Thiam^3^

^1^Centre Régional de Recherche et de Formation (CRCF) du CHNU de Fann, Sciences Sociales, Point E, Senegal, ^2^Comité de Veille et d'Alerte, Dakar, Senegal, ^3^Conseil National de Lutte Contre le VIH Sida du Sénégal (CNLS), Dakar, Senegal

**Background: **In Senegal, HIV prevalence among female sex workers (FSWs) remains high (18.5% in 2010 to 5.8% in 2022). This study focuses on the vulnerabilities generated by the emergence of new forms of sex work, strongly influenced by digital technologies.

**Methods: **The study, supervised by the Conseil National de Lutte contre le Sida and financed by the Global Fund, adopted a qualitative approach with 32 individual interviews and 17 focus groups conducted between February and June 2022 in eight Senegalese regions. A total of 156 FSWs and 25 health agents were interviewed, and the data were analysed using an inductive approach and the Dedoose application. The study was approved by the Senegalese Health Research Ethics Committee.

**Results: **The FSWs surveyed, mostly divorced or widowed women aged between 20 and 50, have abandoned traditional solicitation in favour of new methods of contacting customers, mainly via social networks. The emergence of these new practices has led to peer groupings to rent dedicated spaces, often assisted by brokers facilitating interactions with customers online. The adaptation of FSWs to strategies of concealment, normalization and even professionalization is reflected in their reduced visibility in traditional spaces, commonplace dress, and reduced consumption of drugs and alcohol. However, these changes have also created new vulnerabilities. FSWs are now exposed to online risks, such as blackmail from website managers, non‐consensual video capture and the threat of public disclosure. These new forms of prostitution, facilitated by digital technologies, increase the clandestinity of practices, making access to HIV prevention interventions and regular medical care difficult.

**Conclusions: **This study makes a valuable contribution to the HIV programme by highlighting the need to adapt HIV prevention interventions to take account of new forms of prostitution and the vulnerabilities they entail. It is imperative to rethink prevention approaches, increase access to medical care and raise awareness among SWs of the increased risks associated with these developments.

### OAD1405 Tough Talks virtual simulation HIV disclosure intervention for young men who have sex with men living with HIV: results from a randomized controlled trial

B. Stamp ^1^, K. Muessig^2^, K. Powers^1^, Z. Soberano^2^, S. Levintow^1^, M. Rosso^2^, M. Adams Larsen^3^, L. Hightow‐Weidman^2^

^1^University of North Carolina at Chapel Hill, Gillings School of Global Public Health, Department of Epidemiology, Chapel Hill, United States, ^2^Florida State University, Institute on Digital Health and Innovation, College of Nursing, Tallahassee, United States, ^3^Virtually Better Inc., Decatur, United States

**Background: **Disclosing one's HIV serostatus can improve adherence to care or medications and reduce transmission risk behaviours among young men who have sex with men (YMSM) with HIV. *Tough Talks*, a digital health intervention, was designed to build decision‐making skills for HIV status disclosure using artificial intelligence (AI)‐facilitated role‐playing scenarios with digital avatars.

**Methods: **We enrolled 156 YMSM (ages 16−29) into a three‐arm, parallel‐group randomized controlled trial (NCT03414372) from May 2019 to April 2022 and followed each participant for 6 months. Participants were randomized 1:1:1 to either the: (1) *Tough Talks* Participant‐Driven intervention, in which each participant had unlimited digital access to intervention features; (2) *Tough Talks* Staff‐Driven intervention, in which study staff scheduled two in‐person or online meetings for participant intervention use within a 30‐day window, and unlimited self‐directed digital access thereafter; or (3) standard of care (SOC). We conducted binomial regression analyses with inverse probability of treatment weighting (IPTW) to estimate weighted probability differences (PDs) in 6‐month viral suppression (HIV RNA <20 copies/ml) and weighted risk differences (RD) in 6‐month condomless anal intercourse (CAI) with a potentially susceptible partner, defined as ≥1 act of CAI with a partner who was not known to have HIV, comparing each of the intervention arms with the SOC arm.

**Results: **Of the 156 participants, 129 (83%) completed their 6‐month survey, and 119 (76%) had a viral load measured at 6 months. The probability of viral suppression at 6 months was higher among those randomized to the staff‐driven arm versus SOC (PD = 0.072; 95% CI = 0.022, 0.122) and lower in the participant‐driven arm versus SOC (PD = −0.044; 95% CI = −0.094, 0.005). Similarly, 6‐month risk of CAI with a susceptible partner was lower in the staff‐driven arm versus SOC (RD = −0.277; 95% CI = −0.334, −0.220) and higher in the participant‐driven arm versus SOC (RD = 0.077; 95% CI = 0.022, 0.131).

**Conclusions:** *Tough Talks* may work most effectively as a hybrid intervention that allows for human interaction to help improve decision‐making capacity for HIV status disclosure. Recent advances in AI chatbots may also enhance similar interventions by increasing conversational complexity, facilitating greater rapport and increasing intervention efficacy.

### OAD1602 Red Umbrella Academy: empowering sex workers in HIV prevention in Europe—lessons, challenges and advocacy for PrEP access through community‐based participatory research

L. Stevenson ^1^, F. Belizario^2^

^1^European Sex Workers Rights Alliance, Amsterdam, the Netherlands, ^2^University of Coimbra, Coimbra, Portugal

**Background: **Numerous reports highlight the scarcity of consistent data on health services´ access for sex workers (ESWA, 2021; ECDC, 2015). In alignment with the Sex Workers Implementation Tool (SWIT), developed by WHO, UNAIDS and other stakeholders, the European Sex Workers’ Alliance (ESWA) implemented the second edition of the Red Umbrella Academy (RUA) from May 2023 to January 2024. This initiative aimed to enhance sex workers’ knowledge of HIV and combination prevention, document pre‐exposure prophylaxis (PrEP) access, identify challenges in access to combination prevention, and emphasize the role of peer‐workers and community leadership in HIV prevention.

**Description: **RUA engaged 24 sex workers’ rights activists (SWRA) from Armenia, Austria, France, Germany, Netherlands, Poland, Sweden and Turkey, and employed community‐based participatory research (CBPR) to comprehensively analyse the situation of HIV prevention access by sex workers (SWs), particularly to PrEP. Facilitated by SW, the academy activities focused on advocacy, quality of life, community leadership and discrimination. SWRA also facilitated exploratory research on sex workers’ PrEP access, consisted of in‐depth interviews with 44 respondents. Findings will be disseminated by RUA trainees utilizing methodologies acquired during the programme.

**Lessons learned: **The training and the research not only augmented comprehension regarding PrEP but also elucidated the distinct challenges encountered by disparate countries in the implementation of effective HIV prevention services for sex workers. Disparities in PrEP availability, affordability and acceptability underscored the necessity for integrated services, data collection on adherence, SW empowerment and acknowledgement of their specific needs and constraints. Overall, PrEP is not only a source of prevention but is seen as a source of confidence, an opportunity to ensure adherence to health services, educate the community about HIV prevention and treatment and the opportunity to empower the community to educate their surroundings.

**Conclusions/Next steps: **The Red Umbrella Academy serves as a model for CBPR efficacy, addressing data gaps and empowering sex workers as key advocates. The findings stress the importance of sex workers as advocates, establishing a foundation for impactful community outreach and research programmes.

### OAD1603 Co‐design of national information campaign: Unveiling Australia's community‐led HIV Treatment for All campaign

L. R. Melanie ^1^, L. Stackpool‐Moore^2^, S. Finnegan^2^, A. Cogle^3^, B. Clifton^3^, A. Ogier^3^, E. Oxenburgh^3^, D. Reeders^3^, M. Valencia Moreno^4^, C. Afele^5^, J. Y.‐H. Chen^6^, L. Durow^7^, D. Cordner^8^, K. Sinclair^9^, B. Newham^3^

^1^Watipa, Brisbane, Australia, ^2^Watipa, Sydney, Australia, ^3^National Association of People with HIV Australia (NAPWHA), Sydney, Australia, ^4^Peer‐led Community Advisory Group Member, Brisbane, Australia, ^5^Peer‐led Community Advisory Group Member, Hamilton, New Zealand, ^6^Peer‐led Community Advisory Group Member, Sydney, Australia, ^7^Graphic Design Consultant, Sydney, Australia, ^8^Graphic Design Consultant, Melbourne, Australia, ^9^Web Consultant, Sydney, Australia

**Background: **This abstract provides a dual perspective on Australia's ground‐breaking “HIV Treatment for All” awareness campaign, shedding light on both the campaign's transformative policy shift and the collaborative efforts of key stakeholders propelling it forward.

The campaign informs about Australia's pivotal transition from an industry‐sponsored antiretroviral treatment (ART) compassionate access scheme to the Federation Funding Agreement (FFA) model. This shift ensures free HIV testing and treatment for all residents, regardless of eligibility for Medicare, which is Australia's subsidized public healthcare. The policy change was initially announced on World AIDS Day 2021 and came into effect in mid‐2023.

**Description: **The campaign emphasizes timely testing and treatment, particularly for people in Australia without Medicare, who are often people on student, travel and temporary work visas. The campaign explains implementation details across states and territories, such as where the scheme covers medication costs, and in jurisdictions where additional expenses related to care or treatment side effects are not included. A peer‐led community advisory group guided the process throughout and people living with HIV were also involved within the campaign's collaborative project team.

**Lessons learned: **Spearheaded by the National Association of People with HIV Australia (NAPWHA), the campaign models an example of community leadership in co‐designing a national public health promotion campaign. NAPWHA's national influence and expertise in championing the rights of people living with HIV serve as the campaign's driving force. Diverse and inclusive community leadership at every stage ensured authenticity and relevance, supported by expertise in health, community engagement and advocacy. The collaborative effort incorporated insights from community advisory group members in Australia and New Zealand.

**Conclusions/Next steps: **The success of the “HIV Treatment for All” campaign lies in the seamless integration of diverse roles, emphasizing the importance of inclusive, community‐driven initiatives. This multidisciplinary approach paves the way for equitable access to HIV testing and treatment for all in Australia. Interested parties are invited to explore the campaign's implementation details on the website https://napwha.org.au/hiv‐treatment‐for‐all/, promoting transparency, awareness and community engagement.

### OAD1605 Community HIV Index Testing: A case for revitalizing Primary Health Care in improving uptake of Same Day/Fast‐Track ART initiation of newly diagnosed PLHIV in Botswana

Peter Chibatamoto ^1^ Watipa Gaogane^2^, Alice Sejamitlwa^1^ Emmanuel Kanyane^1^, Caroline Nyambe^1^, Lekopanye Difongo^1^, Kgomotso Sibiya^1^, Moses J. Zulu^1^

^1^Humana People to People Botswana (HPP), Plot 823 Pabalelo Way, Extension 2, P O Box AD 595 ADD, Kgale View, Gaborone, Botswana

**Background: **Botswana is implementing layered HIV prevention programs targeting those most‐at‐risk of acquiring HIV. Approaches include community index testing, same‐day initiation of ART (SDART) for all persons diagnosed with HIV and ready to start treatment, and fast‐track initiation within two weeks of HIV diagnosis. The purpose of this study was to assess the extent to which community‐based HIV index testing contributes to same‐day and fast‐track initiation.

**Description: **HPP Botswana (HPPB), a local NGO, provides community‐based HIV index testing and linkage services in eight health districts in Botswana. This study describes routine HIV testing services (HTS) data (March 2021−September 2023) collected by trained HPP community health workers (CHWs). Using a health facility HIV client list, HPP CHWs engaged PLHIV as index clients and elicited their sexual contacts for HIV testing, following Botswana's National HTS guidelines. At community‐level, CHWs screened contacts for HIV using HIV self‐testing kits and confirmed HIV status through diagnostic HIV rapid test kits. Positive clients were offered same‐day initiation (SDART) or fast‐track options.

**Lessons learnt: **HPPB tested 9,702 sexual contacts of index clients for HIV, yielding a 17% positivity rate, 67% SDART rate, 84% fast‐track initiation rate, and a 96% overall initiation rate. Men comprised 57% of the index clients tested. Although there were no noticeable differences in positivity rates and same‐day initiation (SDART) between the sexes, women had slightly higher fast‐track (86%) and overall initiation (98%) rate as compared to their male counterparts (81% and 94% respectively).

**Conclusions/Next steps: **Community‐based HIV index partner testing is effective in HIV case‐finding and timely ART initiation amongst both men and women. Synchronization of community index partner testing with same day/fast track ART initiation is an important treatment as prevention (TasP) intervention in supporting Botswana's goal of ending AIDS by 2030 and may be effective in reaching hidden pockets of unknown HIV cases.

**Figure**. OAD1605

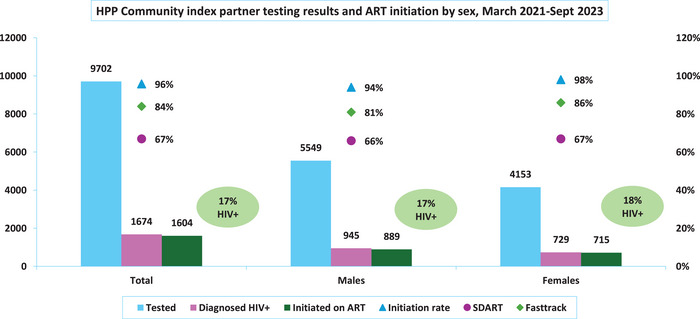

### OAD1902 Comprehensive support and rehabilitation for women and children affected by conflict: a holistic approach to HIV in Ukraine

H. Skipalska^1^, T. Castillo^2^, A. Saienko ^1^, T. Khytryk^1^

^1^Ukrainian Foundation for Public Health, Kyiv, Ukraine, ^2^HealthRight International, New York, United States

**Background: **The prolonged conflict in Ukraine has led to significant challenges for women and children, including displacement, violence and social disintegration, as well as over 12,200 HIV cases in 2022. In response, a holistic programme has been implemented to provide comprehensive support to women and children affected by or at high risk for HIV. This initiative includes the establishment of three Day Centers and three Halfway Houses. The programme utilizes various support strategies and the innovative @TakeCareUA_bot for health education.

**Description: **Our project aimed to address the multifaceted challenges faced by clients affected by conflict, particularly those with HIV. Our comprehensive approach included psychological counselling vocational training for practical skills, and legal support to protect beneficiaries’ rights. The holistic services aimed to foster resilience and empower individuals towards recovery and self‐sufficiency. A robust referral process ensured seamless access to medical care, with 33 clients referred to healthcare facilities, including 17 initiating antiretroviral therapy. In total, 2425 clients received comprehensive care, with a focus on HIV services. Testing services identified 5 syphilis cases, 10 hepatitis C cases and 36 HIV cases. The project's commitment to a seamless referral system reinforced its client‐centred and comprehensive approach.

**Lessons learned: **Our project's success is evident not just in numbers but in the integrated approach and collaboration between Day Centers and Halfway Houses aligned with UN standards for Women and Girls Safe Spaces. This approach ensures comprehensive services, such as group and individual counselling, medical service coordination, document restoration assistance and facilitating children's enrolment in kindergartens. Technology, specifically the @TakeCareUA_bot chatbot, has significantly improved service accessibility and efficiency by providing information, emotional support and directing clients to appropriate services.

**Conclusions/Next steps: **A holistic approach, integrating physical shelter, psychological support, medical care, legal assistance and technological innovation, proves impactful. This not only addresses immediate needs but also fosters long‐term resilience and empowerment for women and children affected by HIV in conflict regions. The success of this model emphasizes the significance of a comprehensive service continuum in tackling multifaceted challenges in such contexts.

### OAD1903 Exploring the impact of digital platforms in increasing access to PREP and mental health support

S. Atuhura ^1^, M. Kimbugwe^1^

^1^Spectrum Uganda Initiatives, Human Rights, Kampala, Uganda

**Background: **The enactment of the Anti‐Homosexuality Act, 2023 in Uganda created significant obstacles for the provision of essential health services to the LGBTIQ community. Access to crucial services like PREP and mental health support has become increasingly challenging. The law imposed heightened scrutiny and constraints on LGBTIQ drop‐in centres, which previously served as safe spaces for healthcare provision. The immediate impact of the law registered the number of young MSM on PREP dropping from 106 to 12 within a month. Access to mental health services for young MSM plummeted from 95 to zero.

**Description: **Spectrum Uganda Initiatives developed an online application that facilitates virtual meetings between young MSM aged 15−24 years and healthcare workers. The application has a directory of anonymous friendly health workers operating across the country. The main objective was for the digital platform to allow clinicians to enrol individuals in virtual adherence clubs, where they can freely access comprehensive information on PREP adherence and mental health support.

**Lessons learned: **Twenty‐six virtual adherence clubs comprising of 10−12 members were formed. Each member was given a unique referral code which was used for linkage to a key populations’ clinic. The numbers adhering to PREP skyrocketed to 268 young MSM within 6 months. One hundred and fifty young MSM embraced the opportunity to access mental health support through the digital platform.

**Conclusions/Next steps: **This innovative digital solution has proved instrumental in overcoming the challenges imposed by the hostile legal environment in Uganda. By providing a virtual space for healthcare services, the application has effectively bridged the gap in access to essential HIV information, PREP adherence and mental health support for young MSM.

### OAD1904 HIV pre‐exposure prophylaxis awareness and willingness to use among migrants in transit through Central American countries

D. Cerecero‐García ^1,2^, F. Macías González^1^, H. Vermandere^1^, C. Palma^3^, S. Soberanis^3^, S. Lungo^3^, S. Bautista‐Arredondo^1^

^1^National Institute of Public Health, Cuernavaca, Mexico, ^2^Imperial College, Department of Primary Care and Public Health, London, United Kingdom, ^3^Pan American Social Marketing Organization, Guatemala, Guatemala

**Background: **The global movement of people has reached unprecedented levels in recent years, presenting challenges and uncertainties for migrants. One particular risk that migrants in transit often encounter is an increased vulnerability of HIV transmission, emphasizing the necessity for targeted prevention strategies. This study focuses on PrEP awareness and willingness to use it among migrants across five Central American countries.

**Methods: **As part of USAID's Prevention Services against HIV project in Central America, we conducted a study between September 2022 and September 2023, involving 2513 international migrants in transit through Mexico, Guatemala, El Salvador, Honduras and Panama. The study assessed PrEP awareness and willingness to use among the general sample and key populations (i.e. LGBTQ+ migrants, MSM, transgender individuals and sex workers). PrEP eligibility was defined as either having engaged in condomless sex with more than one sexual partner, having had a previous STI diagnosis or having engaged in sex under the influence of drugs during transit. Multivariate logistic regression models were utilized to explore predictors of PrEP awareness and willingness to use.

**Results: **Among key populations, 38.7% of migrants met the eligibility criteria for PrEP, compared to only 6.8% among the general population. PrEP awareness was 29% among key populations versus 8% among the general population. Willingness to use daily (70% vs. 56%), injectable (50% vs. 41%) and implant (48% vs. 36%) PrEP was significantly higher among key populations. Engaging in sex during transit was positively and significantly associated with willingness to use all PrEP schemes. Compared with women, men were less likely to be willing to use injectable (OR = 0.72; 95% CI 0.61−0.96) and implant (0.57; 95% CI 0.48−0.68) PrEP. Having an HIV test in the past 12 months was significantly associated with higher odds of PrEP awareness (OR = 1.64; 95% CI 1.29−2.23).

**Conclusions: **The heightened HIV risk faced by migrants in transit underscores the urgent need for targeted prevention strategies. The high willingness to use in our sample suggests that preventive PrEP should be considered as part of preventive measures offered to migrants, especially among key populations, given their high eligibility rate.

### OAD1905 Exploring uncharted territories: understanding adolescent sexuality, gender and HIV risk among refugee girls in Southwestern Uganda

U. Muhumuza ^1^, E. S. Abay^2^, Z. Nahumuza^3^

^1^Africa CDC, Kampala, Uganda, ^2^Africa CDC, Addis Ababa, Ethiopia, ^3^International University of Africa, Khartoum, the Sudan

**Background: **This groundbreaking research delves into unexplored terrain, seeking to unravel the intricate dynamics of adolescent sexuality, gender and HIV risk among refugee girls in Southwestern Uganda. Distinct from previous studies, this research pioneers an in‐depth examination of this vulnerable demographic that is faced with unique challenges, such as displacement, cultural adaptation and limited access to resources shedding light on nuanced factors that remain understudied. By addressing gaps in existing literature, this study contributes significantly to the high‐level scientific discourse on HIV prevention in marginalized populations.

**Methods: **Conducted over 18 months, this study included a meticulously selected sample of 500 adolescent refugee girls, aged 13−18, from diverse camps in Southwestern Uganda. Employing a mixed‐methods approach, quantitative data were collected through structured surveys, while qualitative insights were gathered through in‐depth interviews and focus group discussions. Rigorous statistical analyses, including regression models and chi‐square tests, were employed to elucidate patterns and associations.

**Results: **The research reveals compelling statistics: 35% of participants reported early marriages, with a staggering 45% lacking comprehensive knowledge of HIV prevention methods. Gender disparities were evident, with 60% of girls facing limited access to healthcare resources. Notably, older adolescents (16−18 years) exhibited a 25% higher vulnerability to HIV risk factors compared to their younger counterparts. These findings underscore the urgency for tailored interventions.

**Conclusions: **The statistically significant disparities underscore the urgency for targeted interventions. Age differentials highlight the need for age‐specific strategies, while gender disparities necessitate tailored healthcare and educational initiatives. The 35% prevalence of early marriages emphasizes the critical role of empowering adolescent girls to make informed decisions. The 45% knowledge gap in HIV prevention mandates a comprehensive and accessible education strategy. The 25% increased vulnerability among older adolescents underscores the need for transition‐focused interventions. This research, with its high‐level statistical insights, not only unveils the complexities of HIV risk among adolescent refugee girls but also provides a roadmap for evidence‐based interventions and policy initiatives, setting a new standard for scientific inquiry in this critical field.

### OAD2402 Exploring the impact of climate‐induced migration on HIV vulnerability in Africa

O. Ekerin ^1,2^, D. O. Shomuyiwa^3,4^, O. Sonaike^5^, R. Mojeed^6,7^, I. Gbajumo^5,8^, A. Ahmed^6,9^, A. Ogunsanya^6^

^1^School of Public Health, University of Port Harcourt, Port Harcourt, Nigeria, ^2^State AIDS and STIs Control Program, Department of Public Health, Kaduna State Ministry of Health, Kaduna, Nigeria, ^3^Faculty of Pharmacy, University of Lagos, Lagos, Nigeria, ^4^Global Health Focus, Lagos, Nigeria, ^5^Lagos State University, Lagos, Nigeria, ^6^University of Port Harcourt, Port Harcourt, Nigeria, ^7^State AIDS and STIs Control Program, Department of Public Health, Ekiti State Ministry of Health, Ekiti, Nigeria, ^8^Clafiya, Lagos, Nigeria, ^9^State AIDS and STIs Control Program, Department of Public Health, Kwara State Ministry of Health, Kwara, Nigeria

**Background: **Rising global temperatures and environmental changes have prompted unprecedented migration patterns in Africa, with profound implications for public health. This study delves into the relationship between climate‐induced migration and the heightened vulnerability of populations to HIV infection in Africa. As the number of environmental migrants continues to rise, understanding the intersectionality of climate‐induced migration and HIV risk becomes imperative for targeted intervention strategies. This research seeks to identify the specific factors linking climate‐induced migration to increased susceptibility to HIV, considering the socio‐economic, geographical and demographic dimensions of affected populations.

**Methods: **A comprehensive literature review was conducted, synthesizing findings from epidemiological studies, migration reports and public health databases. The analysis focused on identifying patterns and trends related to climate‐induced migration and HIV vulnerability in Africa. Peer‐reviewed articles, reports from international organizations and demographic health surveys were systematically reviewed to extract relevant data on the prevalence of HIV among climate‐induced migrants and the specific factors contributing to heightened vulnerability. The research also examined the impact of climate‐induced migration on healthcare infrastructure, access to prevention programmes and the social determinants influencing HIV transmission dynamics.

**Results: **The review identified a limited number of studies specifically addressing the intersection of climate‐induced migration and HIV vulnerability in Africa. Preliminary analysis of available data revealed that regions experiencing climate‐related stressors, such as droughts and floods, often witness increased migration rates. However, the impact of these migrations on HIV prevalence and vulnerability is complex and varies across different contexts. Socio‐economic factors, including access to healthcare and HIV prevention resources, play a crucial role in shaping the overall risk landscape for migrants.

**Conclusions: **The findings highlight the urgent need for more targeted research on the relationship between climate‐induced migration and HIV vulnerability in Africa. Addressing this gap will require interdisciplinary collaborations between environmental scientists, healthcare professionals and policymakers to develop comprehensive strategies that integrate HIV prevention and treatment services into climate‐related migration policies. By understanding the nuanced dynamics at play, interventions can be tailored to mitigate HIV risk among migrant populations, contributing to both public health and climate adaptation efforts.

### OAD2403 Climate change‐related factors and HIV vulnerabilities among very young adolescents in Kenya: multi‐method qualitative findings

C. Logie ^1^, J. Kagunda^2^, H. Evelia^3^, C. Gachoki^2^, B. Omondi^3^, L. Gittings^4^, A. Hasham^1^, I. Sternthal^1^, S. Van Borek^1^, L. Taing^5^, M. Narasimhan^6^

^1^University of Toronto, Factor‐Inwentash Faculty of Social Work, Toronto, Canada, ^2^Elim Trust, Nairobi, Kenya, ^3^Centre for Study of Adolescence, Nairobi, Kenya, ^4^Western University, London, Canada, ^5^United Nations University Institute for Water, Environment and Health, Hamilton, Canada, ^6^World Health Organization, Geneva, Switzerland

**Background: **There is growing evidence of linkages between climate change‐related factors, such as water insecurity, and HIV vulnerabilities. Yet, knowledge gaps persist regarding climate change and HIV prevention needs, and climate experiences among very young adolescents (VYA) ages 10−14. We conducted a multi‐method study to explore climate change‐related factors and linkages with HIV vulnerabilities among VYA in climate‐affected Kenyan regions.

**Methods: **This multi‐method study in six sites (Nairobi, Naivasha, Kisumu, Isiolo, Kilifi, Kalobeyei refugee settlement) involved: *n* = 12 focus groups (FGs) with elders, *n* = 60 VYA walk‐along interviews and *n* = 12 two‐day VYA participatory mapping workshops. We conducted thematic analysis informed by the resource insecurity framework, and integration with a mixed‐methods matrix.

**Results: **Participants (*N* = 297) included: elders (*n* = 119; mean age: 60.6 years, standard deviation [SD]: 7.9; men: 48.7%, women: 51.3%), youth walk‐along interviewees (*n* = 60; mean age: 13.4, SD: 1.5; boys: 51.4%, girls: 48.6%) and youth participatory mapping participants (*n* = 118; mean age: 12.1, SD: 1.33; boys: 50.8%, girls: 49.2%). Participant narratives revealed climate‐related changes increased food insecurity, water insecurity and sanitation insecurity. Each resource insecurity was linked with unique and overlapping HIV vulnerabilities. Food insecurity was associated with youth running away, and transactional sex and exploitative relationships for food, which contributed to early pregnancy. Water insecurity was associated with: menstruation hygiene management challenges; sexual violence risks travelling far/at night for water; and transactional sex and exploitative relationships for water. Sanitation insecurity was associated with: sexual violence risks accessing showers, toilets and garbage disposal sites; and transactional sex for menstruation products, which elevated early pregnancy risks. Heavy rains and floods were raised as particularly dangerous for young women/girls due to infrastructure damage and subsequent sexual violence exposure. While sanitation insecurity was common across sites, water and food insecurity were raised most frequently in Kalobeyei (refugee settlement) and Isiolo (nomadic and pastoralist community).

**Conclusions: **Together, findings signal water, food and sanitation insecurity are social drivers of HIV vulnerability among VYA in Kenya. Climate‐informed interventions can consider seasonality influences, contextual differences and target mechanistic pathways at interpersonal (e.g. transactional sex/exploitative relationships), community (e.g. gender inequitable norms) and structural (e.g. sanitation infrastructure, poverty) levels to advance HIV prevention with VYA in Kenya.

### OAD2404 HIV and general health status of displaced populations receiving medical care at mobile clinics after severe flooding in Mulanje district, Malawi

J. Njala ^1^, H. Chimbaka^1^, A. Makwaya^1^, T. Banda^1^, M. Mtulutsa^1^, D. Smith^1^, L. Njikho^2^, J. Chikonde^3^, B. Tauzie^4^, J. Songo^1^, M. Mphande^1^, K. Balakasi^1^, K. Phiri^1^, J. J. Van Oosterhout^1^, S. Phiri^1^

^1^Partners In Hope, Lilongwe, Malawi, ^2^Ministry of Health, Mulanje, Malawi, ^3^UNICEF, Blantyre, Malawi, ^4^UNICEF, Lilongwe, Malawi

**Background: **Due to the rising impact of climate change, Malawi has experienced increased extreme weather events. These include devastating floods and mudslides that resulted in large‐scale displacement of vulnerable populations. In response, Ministry of Health and partners have conducted outreach clinics close to camps for displaced persons. However, little is known about health status and needs of the affected populations.

**Methods: **In November 2023, Partners In Hope (PIH) conducted a cross‐sectional survey of individuals aged ≥18 years utilizing health services at mobile outreach clinics conducted at seven campsites in Mulanje district, set up after flooding caused by Tropical Cyclone Freddy. We describe demographic characteristics, prevalence of self‐reported acute and chronic conditions, depression (PHQ score 1−6) and intimate partner violence (IPV) and health service satisfaction, stratified by residence status (displaced/non‐displaced).

**Results: **Of 341 participants surveyed, median age was 32 years (IQR 23−47 years), and 80% were female. Fifty‐eight percent (197/341) were displaced persons, the rest resided close to the camps (non‐displaced). Displaced participants more frequently had: no formal education (32.5% vs.15.3%; *p*<0.001), worse self‐reported health (41.6% vs. 23.6%; *p*<0.001), respiratory illness (23.8% vs. 13.4%; *p* = 0.02), COVID‐19 symptoms (19.6% vs. 11.2%; *p* = 0.04), HIV testing need (23.4% vs. 14.6%; *p* = 0.04) and under‐5 service need (14.7% vs. 7.6%; *p* = 0.04). The displaced had similar chronic disease prevalence (42% vs. 38%; *p* = 0.389) and unknown HIV status prevalence (1.0% vs. 4.2%; *p* = 0.163) as residents. All PLHIV in both groups were engaged in HIV care (registered on ART 18.8% vs. 18.8%; *p* = 0.300). Rating of mobile services as good was near‐universal in both groups (98%). Similar proportions in both groups screened positive for depression (53.5% vs. 56.3%, *p* = 0.598; 91% was minimal/mild depression overall) and IPV (47.2% vs. 54.6%, *p* = 0.289). Overall prevalence of physical IPV was 16.3% and sexual IPV 15.3%, similar between groups.

**Conclusions: **After severe flooding, mobile outreach clinics were frequented by displaced persons in camps and nearby residents, unable to reach their regular health facility. Given high rates of acute illnesses, chronic conditions (including HIV), depression and IPV, mobile clinics in these settings require multidisciplinary teams with diverse skills to meet the health needs of the attending client population.

### OAD2405 Climate change vulnerability assessment for Orphaned and Vulnerable Children: results from FACT Zimbabwe and Pact. Manicaland and Masvingo provinces, Zimbabwe

D. Chimedza ^1^, T. Mugoni^1^, M. Manditsera^1^, D. Bonnardeaux^2^, E. Olais^2^, T. Chimbidzikai^1^, T. Takaidza^1^, G. Shumba^1^, J. Tavengerwei^1^, E. Nyakanda^1^, T. Nyatsanza^1^, V. Mubaiwa^1^, G. Nyahuma^3^, M. Mukuzunga^4^, T. Nyafesa^5^, B. Mukome^1^

^1^Family AIDS Caring Trust, Safeguarding and Sustainable Livelihoods, Mutare, Zimbabwe, ^2^Pact, Environment, Climate, Energy, Washington, DC, United States, ^3^USAID, Harare, Zimbabwe, ^4^Ministry of Health and Child Care, PMD, Mutare, Zimbabwe, ^5^Ministry of Health and Child Care, Maternal and Neonatal Child Health, Mutare, Zimbabwe

**Background: **FACT Zimbabwe is conducting its Orphans and Vulnerable Children (OVC) programme in nine districts within the provinces of Manicaland and Masvingo, with funding from PERPFAR/USAID. The project aims to contribute to the HIV epidemic by providing support to children who are HIV positive and their caregivers. Climate change (CC) is one of the cross‐cutting components covered in the OVC for 0−17 years. Zimbabwe has recently seen several natural disasters, such as cyclones and droughts, and children's health, development, nutrition, education and access to healthcare services are negatively impacted. FACT Zimbabwe from March to April 2023 conducted a CC vulnerability assessment to establish the extent at which OVC and their caregivers were affected by CC.

**Description: **Pact's Community‐Based Adaptation Framework was used in Manicaland and Masvingo, selected through bio‐climate clustering to represent all five agroecological zones and urban and rural contexts. Data were collected from daily clocks, vulnerability matrices, impact chains, adaptation pathways and historical timelines. A participatory and qualitative approach was used with primary data obtained from focus group discussions (*n* = 585) and secondary data from key informant interviews (*n* = 51), targeting OVC and community leaders in March—April 2023.

**Lessons learned: **The thoughts of rainy seasons caused mental health disorders among caregivers, adolescents and youths. Hunger due to crop/livestock failure exposed OVC to malnutrition/delayed milestones. Adolescent girls/young women were pushed into transactional sex, teen pregnancy/early marriages, especially those close to transport hubs and borders. Floods affected access to clinics, hindering ART refill/adherence. Innovations included multi‐month ART dispensing, scale‐up of indigenous chicken and planting small grains. With water scarcity, diminishing forests and the destruction of schools, OVC walking longer distances to fetch water and firewood and to school exposed themselves to emotional, physical/sexual abuse. The use of solar‐powered boreholes and stoves, improved transportation such as—bicycles for OVC and psychosocial support were sustainable strategies. Substance abuse was a challenge as youths tried to cope with stress, and OVC were not spared. Community‐led OVC safety nets were said to be a necessity for stability.

**Conclusions/Next steps: **CC impacts OVC disproportionately because of their vulnerability/exposure to climate stresses/shocks. Exploration of adaptive capacity is required in communities at risk.

### OAD3202 Major intersectional discrimination associated with increased HIV and bacterial STIs among gay, bisexual and other men who have sex with men in the United States

K. Atkins ^1^, J. M. Wiginton^2^, T. Carpino^3^, A. Scheim^4^, S. Murray^5^, T. Sanchez^6^, S. Baral^3^

^1^Johns Hopkins Bloomberg School of Public Health, International Health, Baltimore, United States, ^2^University of California, San Diego, San Diego, United States, ^3^Johns Hopkins Bloomberg School of Public Health, Epidemiology, Baltimore, United States, ^4^Drexel University Dornsife School of Public Health, Epidemiology and Biostatistics, Philadelphia, United States, ^5^Johns Hopkins Bloomberg School of Public Health, Mental Health, Baltimore, United States, ^6^Emory University Rollins School of Public Health, Epidemiology, Atlanta, United States

**Background: **Intersectional discrimination is a critical determinant of health for marginalized communities. We sought to characterize intersectional discrimination as a potential driver of HIV and bacterial STIs among cisgender gay, bisexual and other men who have sex with men (MSM).

**Methods: **A US nationwide online survey of MSM (October 2022−June 2023) assessed social systems exclusion and interpersonal violence, identified via factor analysis as two domains of the Intersectional Discrimination Index (Figure). We assessed prevalences of each form of discrimination and differences by race/ethnicity and HIV status, using modified Poisson regression with robust variance estimation to calculate prevalence ratios (PR) for associations between each form of discrimination and self‐reported HIV status and past‐year bacterial STI diagnoses.

**Results: **Of 4348 MSM, 1322 (27.8%) reported lifetime experiences of major discrimination, 679 in the last year. Past‐year exclusion was twice as prevalent among MSM of colour (i.e. non‐White; *p*<0.001) and 67% more prevalent among MSM with HIV (*p* = 0.01), and past‐year violence was 30% more prevalent among MSM with HIV (*p*<0.01). MSM reporting past‐year exclusion had 51% higher HIV prevalence and 42% higher bacterial STI prevalence, including 55% and 72% higher prevalences of gonorrhoea and syphilis, respectively, than MSM reporting no past‐year exclusion (Figure). Effects were stronger among MSM of colour. Men reporting past‐year violence exposure had 39% higher HIV prevalence, 23% higher bacterial STI prevalence, and over 30% higher prevalences of gonorrhoea, chlamydia and syphilis than those without past‐year violence exposure; effects were more pronounced among MSM of colour for HIV and chlamydia.

**Figure**. OAD3202

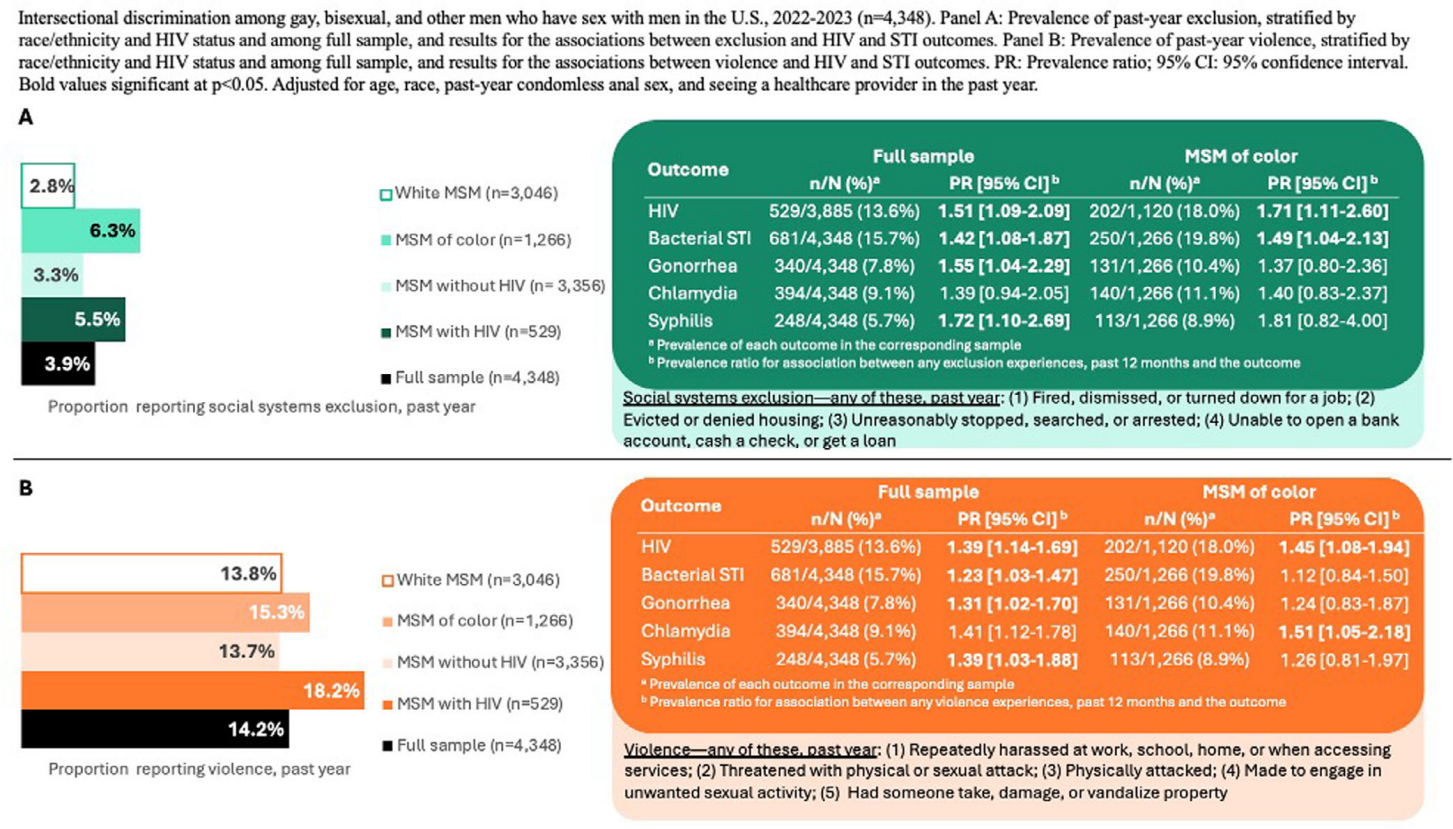

**Conclusions: **Intersectional discrimination via social systems exclusion and violence disproportionately affects men living with HIV and is associated with elevated prevalence of HIV and bacterial STIs, disproportionately so among MSM of colour. Structural interventions are critical to minimizing harms of systemic exclusion and mitigating the health effects of violence among MSM.

### OAD3203 Relationship between discrimination, sexual decision‐making and behaviours that increase the HIV/STI risk among trans and non‐binary people in Germany, a cross‐sectional study

M. Martín‐Sánchez ^1,2^, K. Pöge^3^, A. Hahne^4^, J. Hamm^5^, V. Bremer^1^, U. Koppe^1^, TASG Study Group

^1^Robert Koch Institute, Department of Infectious Disease Epidemiology, Berlin, Germany, ^2^ECDC Fellowship Programme, Field Epidemiology Path (EPIET), European Centre for Disease Prevention and Control (ECDC), Stockholm, Sweden, ^3^Robert Koch Institute, Department of Epidemiology and Health Monitoring, Berlin, Germany, ^4^Freelance Sexual Health Counsellor and Bodyworker, Hamburg, Germany, ^5^Deutsche Aidshilfe, Berlin, Germany

**Background: **Discrimination against trans and non‐binary people has been linked to negative health outcomes, including sexual health. We explored the relationship between discrimination based on gender identity among trans and non‐binary people in Germany and their perceived ability to make decisions to feel as protected as desired from HIV and sexually transmitted infections (STIs). We further assessed whether feeling unable of making decisions to feel protected from HIV/STI was associated with past 12‐month behaviours that increase the HIV/STI risk.

**Methods: **We conducted a cross‐sectional study using data from the TASG online survey, performed using a participatory approach during March−July 2022 among trans and/or non‐binary people aged 18 years and older living in Germany. We described participants characteristics and the frequency of experiencing discrimination based on their gender identity. We calculated prevalence ratios (PR) with 95% confidence intervals (95% CI) for the associations between frequent discrimination and feeling unable of making decisions to feel protected from HIV/STIs, and between feeling unable of making these decisions and past 12‐month sex while under drug influence and condomless penetrative sex with multiple partners without using PrEP.

**Results: **Among 3077 participants, 22.0% identified as female, 21.8% as male, 12.4% as non‐binary female, 12.7% as non‐binary male, 27.0% as non‐binary and 4.0% used other terms. Overall, 22.1% (505/2287) reported frequent discrimination based on their gender identity, and proportions varied across the gender spectrum, with 31.8% (190/597) in non‐binary, 29.3% (76/259) in non‐binary females, 22.0% (69/314) in non‐binary males, 15.9% (79/497) in females and 11.2% (61/547) in males. Participants experiencing frequent discrimination felt more often unable to make decisions to feel protected from HIV/STI (PR 1.4, 95% CI 1.1−1.8). Feeling unable of making decisions to feel protected from HIV/STI was associated with increased prevalence of past 12‐month sex under drug influence (PR 2.9, 95% CI 2.3−3.7) and condomless penetrative sex with multiple partners without PrEP (PR 2.1, 95% CI 1.5−3.0).

**Conclusions: **Feeling unable to make decisions to feel protected from HIV/STI among trans and non‐binary people was associated with both frequent discrimination and behaviours that increase the HIV/STI risk. Strategies for empowering trans and non‐binary people to assert their sexual decision‐making needs should be explored.

### OAD3204 HIV criminalization and enacted stigmas among people living with HIV in countries across Eastern Europe and Central Asia

M. A. Roach ^1^, K. Atkins^2^, G. Turpin^1^, W. Gomez^1^, C. Lyons^1^, H. Moran^1^, A. Rao^1^, O. Syarif^3^, P. Looze^3^, K. Lalak^3^, J. d. D. Anoubissi^3^, F. Chiu^3^, S. Brion^4^, K. Dunaway^4^, L. Sprague^5^, C. Garcia De Leon Moreno^5^, D. Matyushina^5^, A. Leshanok^6^, S. Baral^1^, K. Rucinski^2^

^1^Johns Hopkins University, Department of Epidemiology, Baltimore, United States, ^2^Johns Hopkins University, Department of International Health, Baltimore, United States, ^3^Global Network of People Living with HIV, Amsterdam, the Netherlands, ^4^International Community of Women Living with HIV, Nairobi, Kenya, ^5^UNAIDS, Geneva, Switzerland, ^6^People PLUS, Svetlogorsk, Belarus

**Background: **HIV criminalization includes laws and policies that unjustly penalize people living with HIV (PLHIV). Convictions from HIV criminalization have increased in Eastern Europe and Central Asia (EECA), reinforcing discrimination towards PLHIV and threatening individual health and wellbeing. We examined the relationship between HIV‐related conviction rates and individual experiences of stigma in the legal system, workplace and healthcare across eight countries in EECA.

**Methods: **Country‐level implementation of HIV‐related conviction rates was approximated by the number of people convicted on HIV‐related charges per capita through 2022 per the HIV Justice Network. We measured enacted stigma (Table) using Stigma Index 2.0 cross‐sectional data, collected by networks of PLHIV in Belarus, Georgia, Kazakhstan, Kyrgyzstan, Moldova, Russia, Tajikistan and Ukraine (2020−2023). Multilevel logistic models, clustered at the country level, were used to estimate adjusted odds ratios (aOR) and 95% confidence intervals (CI) for the associations between country‐level HIV‐related conviction rates and enacted legal system, workplace and healthcare stigma.

**Results: **Among 8128 respondents, 50.4% (*n* = 4121) had ever experienced enacted stigma. HIV‐related conviction rates were lower in Kazakhstan, Ukraine, Georgia and Kyrgyzstan; high in Russia and Moldova; and highest in Belarus and Tajikistan. Compared to PLHIV in countries with lower rates of HIV‐related convictions, those in countries with the highest rates of HIV‐related convictions had higher odds of experiencing enacted stigma in the legal system (aOR: 4.88, 95% CI: 2.18−10.95) and the workplace (aOR: 2.12, 95% CI: 1.57−2.86). While not associated with HIV criminalization, almost half of the participants (46.1%, *n* = 3772) reported enacted stigma in the healthcare setting.

**Conclusions: **The relationship between HIV criminalization and enacted stigma among PLHIV reinforces the potentially harmful impact of structural stigma. Changing the trajectory of HIV epidemics in Eastern Europe and Central Asia necessitates community‐led and comprehensive policy and programmatic approaches including addressing punitive laws limiting the impact of HIV services.

### OAD3205 Addressing self‐stigma for young people living with HIV through football in Kampala, Uganda

K. S. Sekabira ^1^, L. Brooks^2^, S. P. Nabude^3^, I. Kusemerirwe^3^

^1^Tackle, Programmes, Kampala, Uganda, ^2^Tackle, Regional Programmes, Mombasa, Kenya, ^3^Tackle, Projects, Kampala, Uganda

**Background: **84.8% of PHIV surveyed showed signs of self‐stigma (People Living with HIV Stigma Index Global Report 2023) affecting their lives negatively. We report the results of a youth‐led, football‐based intervention where learning happens through play, reducing self‐stigma, increasing adherence to ART and improving overall health outcomes for YPLHIV.

**Description: **Over 18 months, 501 (m = 174, f = 327) participants engaged in self‐stigma reduction sessions, delivered by 29 football coaches trained to deliver scenario‐based football games designed to discussed stigma, both through play and during post‐match debriefs. Topics covered during the sessions included identification of self‐stigma, self‐care techniques, U = U, where to find further support for adherence and mental wellbeing, and the importance of speaking about your status and your challenges. Additionally, two football tournaments reached 608 participants where health services were offered alongside football. Sessions took place at ART clinics, health facilities, peer group meets and community hubs. Focus was placed on qualitative data collection. Internalized stigma questions were based on ViiV's self‐stigma index survey.

**Lessons learned: **The programme's endline self‐stigma index surveys (*n* = 335) showed an increase from 54% to 62% willingness to disclose their positive status after the sessions. Those who felt guilty they are HIV positive reduced from 28% to 20%. Feelings of worthlessness due to positive status reduced from 30% to 20%. Those hiding their status reduced from 49% to 45%. Those uncomfortable disclosing their status reduced from 40% to 29%. Qualitative data collection from 33 YPLHIV showed the camaraderie and sense of belonging from playing in a football team, coupled with the fun elements of sport allowed them to relax and open up about their lives to their peer group, seek further support where before they had not, and this led to reduced self‐stigma.

**Conclusions/Next steps: **Tailored, football‐based interventions are an effective mechanism to reduce self‐stigma for YPLHIV, particularly for opening up necessary conversations around identifying self‐stigma, self‐care techniques to reduce it and ART adherence. However, data suggest that more work is needed to shift societal attitudes even as internalized feelings improve.

### OAD3702 Empowering older adults living with HIV: a social support intervention boosts adherence and viral suppression

P. Kakwera ^1^, B. Kwagala^1^, F. Nakyeyune^1^

^1^Wakiso District Network of People Living with HIV (WADNET), Kampala, Uganda

**Background: **As the population ages with HIV, older people living with HIV (OPLHIV) face a unique intersection of vulnerabilities. Age‐related stigma, co‐morbidities and inadequate social support can compromise their health and HIV treatment outcomes. Existing research links stronger social networks to improved HIV control, highlighting the need for targeted interventions.

**Description: **Recognizing the dearth of social support among OPLHIV in Uganda, we implemented a 6‐month social support programme for 60 individuals with poor adherence. Our multi‐pronged approach delivered emotional support through counselling and facilitated family disclosure, informational guidance for informed decision‐making and nutritional support via counselling, kitchen gardens, and food supplements.

**Lessons learned: **The intervention yielded significant improvements in treatment adherence and viral and other key vitals such as weight. Nearly half (46%) of initially unsuppressed individuals achieved viral suppression, while another 54% showed improved viral load control. Additionally, 67% reported enhanced treatment adherence, signifying the programme's effectiveness in addressing critical challenges faced by OPLHIV. Notably, 80% of participants also experienced weight gain, highlighting the programme's impact on overall health and wellbeing.

**Conclusions/Next steps: **Our findings provide compelling evidence for the positive impact of comprehensive social support on OPLHIV outcomes. This model presents a promising, replicable approach for other communities and HIV/AIDS networks seeking to improve OPLHIV's wellbeing and treatment success. Future research should explore the long‐term sustainability of such interventions and their broader applicability in diverse settings.

### OAD3703 Primary outcomes of an intervention employing a hybrid online‐offline approach to address HIV stigma among women living with HIV in Vietnam

C. Lin ^1^, B. D. Nguyen^2^, T. T. Nguyen^2^, H. Dang^2^, L. Li^1^, M. G. Le^2^

^1^Univeristy of California, Los Angeles, Los Angeles, United States, ^2^Hanoi Medical University, Hanoi, Viet Nam

**Background: **There are approximately 80,000 women living with HIV/AIDS (WLHA) in Vietnam. In addition to HIV stigma and gender disparities in employment opportunities, financial power and other social capital, WLHA faced unique challenges stemming from a patriarchal culture in Vietnam. In this context, women are often expected to embody traditional female virtues, maintain a subordinate role to males and shoulder the primary responsibilities of family caregiving. These societal expectations add another layer of complexity to the stigma and delay in healthcare among WLHA in Vietnam.

**Methods: **The study team developed an online‐offline hybrid approach to empower WLHA in Vietnam. This intervention incorporates evidence‐based strategies to boost self‐efficacy, foster positive coping with stigma, motivate service‐seeking and treatment adherence, and engage peer and social support. To allow flexibility, the intervention is designed to be conducted in three modalities: in‐person, Zoom and Zalo (the most popular social media application in Vietnam). The efficacy of this intervention was evaluated through an intervention pilot with a single‐arm design, comparing baseline and 4‐month assessments among 91 WLHA participants recruited in Hanoi, Vietnam.

**Results: **The mean score of WLHA participants’ perceived barrier to access care was significantly reduced from baseline (19.9 ± 0.5) to 4‐month assessment (17.3 ± 0.6; *p*<0.0001). There was significant improvement demonstrated in active cognitive and behavioural coping with HIV (from 58.0 ± 1.0 at baseline to 61.5 ± 1.0 at 4‐month; *p*<0.0001). We also observed a significant reduction in overall stigma score (consisting of awareness of stigma, agreement with stigma and application of stigma subscales) (from 69.9 ± 1.5 at baseline to 65.2 ±1.6 at 4‐month; *p*<0.0001). This reduction was also observed in the HIV stigma attached to females, which declined from 70.9 ± 1.7 at baseline to 64.9 ± 1.7 at the 4‐month assessment (*p*<0.0001).

**Conclusions: **These findings underscore the potential of tailored, flexible interventions that leverage both interpersonal support and social media platforms to empower WLHA and engage them in healthcare services. The study offers valuable insights for similar interventions to reduce the negative impacts of HIV stigma and gender disparities in other similar patriarchal contexts.

### OAD3704 Cognitive trajectories of older adults with and without HIV: a longitudinal population‐based study in rural South Africa

S. Gao^1^, D. Bassil^1^, C. Roberts‐Toler^1^, J. Rohr^1^, R. Wagner^2^, L. Kobayashi^3^, M. Rosenberg^4^, T. Barninghausen^1,5^, K. Kahn^2^, J. Joska^6^, J. Manne‐Goehler ^1,7^

^1^Harvard T.H. Chan School of Public Health, Harvard Center for Population and Development Studies, Boston, United States, ^2^University of the Witwatersrand, MRC/Wits Agincourt Research Unit, Johannesburg, South Africa, ^3^University of Michigan School of Public Health, Department of Epidemiology, Ann Arbor, United States, ^4^Indiana University School of Public Health, Department of Epidemiology and Biostatistics, Bloomington, United States, ^5^Heidelburg Institute of Global Health, Heidelburg, Germany, ^6^University of Cape Town, Cape Town, South Africa, ^7^Brigham and Women's Hospital, Division of Infectious Diseases, Boston, United States

**Background: **Modern antiretroviral therapy has led to ageing of PLWH and a growing prevalence of neurocognitive disorders associated with ageing. However, the relationship between ageing, HIV and cognition remains unclear. We assessed cognitive trajectories among ageing rural South African PLWH compared to people living without HIV (PWOH) overall and by viral suppression status.

**Methods: **We used data from the Health and Aging in Africa: Longitudinal Studies in South Africa (HAALSA), an ongoing population cohort of 5059 adults aged 40 years living in rural South Africa. We conducted linear mixed‐effects models to determine the association between HIV serostatus at baseline and the outcomes of (1) z‐standardized episodic memory score, (2) Activities of Daily Living (ADLs), (3) Instrumental Activities of Daily Living (IADLs) and (4) trail‐making among those with data at three timepoints (*n* = 1603). The episodic memory models were adjusted for practice effects; the remaining models were adjusted for baseline cognition. All models included age, sex, education, wealth index, marital status, country of origin and size of household, a random slope for time, random intercept for individual, and inverse probability weights for attrition and mortality over time.

**Results: **Suppressed PLWH had significantly better episodic memory over time compared to PWOH (b = 0.002, *p*‐value = 0.017). This trend was still significant after adjusting for socio‐demographic characteristics (b = 0.002, *p*‐value = 0.018). There was no significant difference between unsuppressed PLWH and PWOH (b = 0.00, *p*‐value = 0.241). These results were also consistent with the trail‐making test after adjustment (b = 0.03, *p*‐value = 0.044 and b = 0.01, *p*‐value = 0.634, respectively). There were no significant differences in ADL or IADL trend.

**Conclusions: **Suppressed PLWH had better cognitive trajectories in episodic memory and executive function than PWOH, but there was no significant difference between unsuppressed PLWH and PWOH. These results offer further evidence of the importance of viral suppression for healthy cognitive ageing in PLWH.

**Figure**. OAD3704

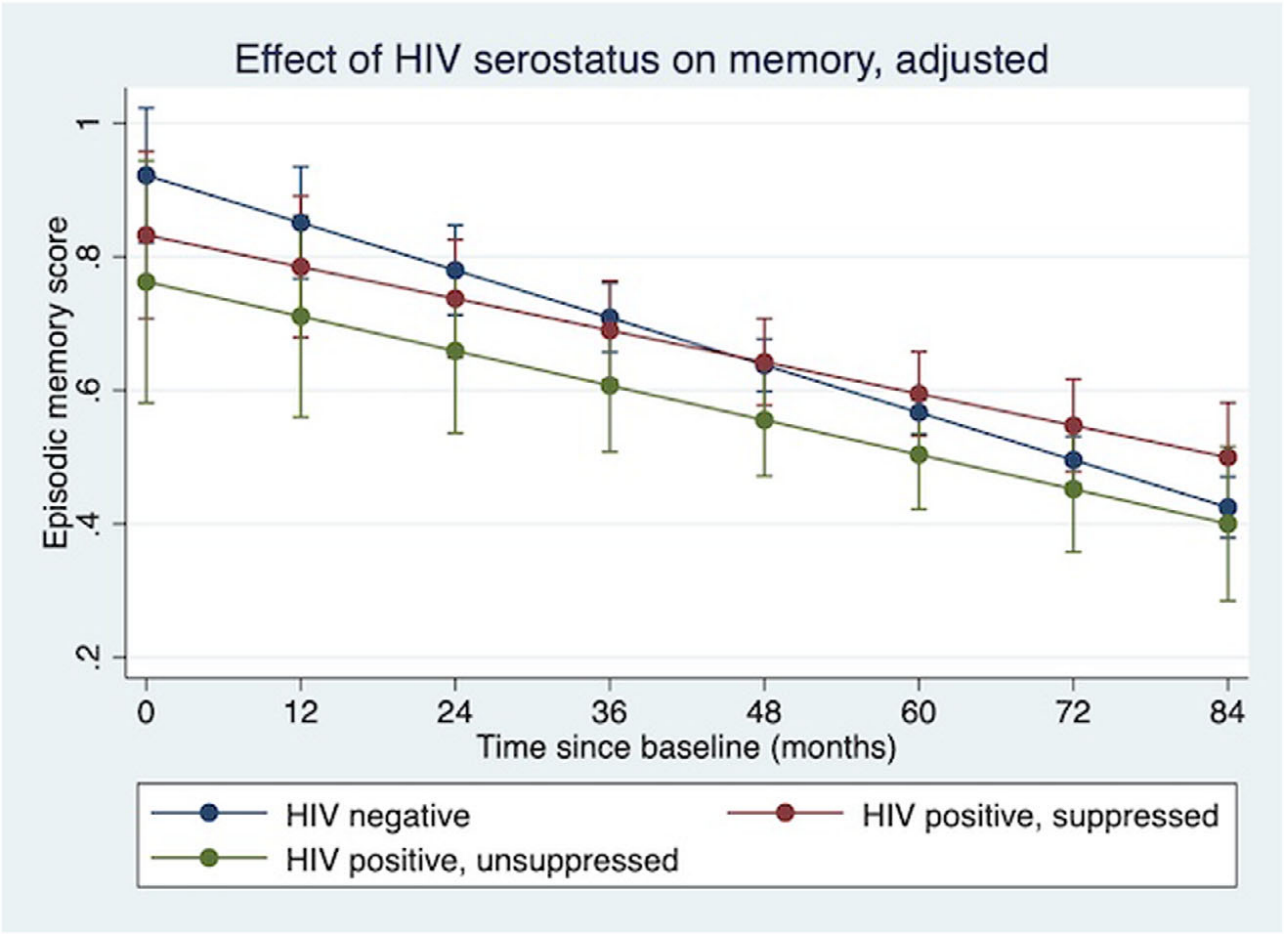

### OAD3705 Experiences and acceptability of Long‐acting Injectable antiretroviral therapy among adolescents enrolled in the Acceptability and Feasibility of long‐acting INjectable ART in Adolescents and Young‐Adults (AFINAty) study in South Africa

M. Atujuna ^1^, L. Jennings^1^, E. Rousseau^1^, R. Panda^1^, N. Mashele^1^, N. Bongo^1^, L.‐G. Bekker^1^, C. Orrell^1^

^1^Desmond Tutu HIV Centre, University of Cape Town, Department of Medicine, Cape Town, South Africa

**Background: **Adolescents living with HIV have struggled to adhere to antiretroviral treatment (ART), with only 10% achieving viral suppression. Long‐acting injectable (LAI) ART in the form of a cabotegravir (CAB) and rilpivirine (RPV) in combination may be a novel solution adolescents find this formulation acceptable.

**Methods: **Within the ongoing AFINAty study investigating the acceptability and feasibility of LAI among adolescents (12−24 years old), we conducted a series of longitudinal in‐depth interviews with a sub‐set of trial‐participants across three cohorts: those with consistent viral suppression on ART; those with evidence of prior poor adherence; and ART‐naïve. Participants had to achieve viral suppression on oral ART before starting LAI. The main areas of inquiry for the qualitative aim included: experience with ART, motivation to start LAI, prior experience with LAI, preference between LAI and oral ART. We analysed data using a thematic and structured framework approach.

**Results: **Using data collected in the first round of interviews, four broad themes emerged: (1) *LAI simplifies life, while pills complicate life,* where the use of LAI reduces the burden of having to plan their lives around their daily pill‐taking schedules; (2) *Living life fully*, where with LAI, participants, for the first time, expressed feeling the freedom of living like individuals who were HIV‐free, as they did not have to worry about anything, only to attend their next appointment; (3) *LAI completely removed the fear of unplanned HIV disclosure* that often occurred among peers and family members caused by carrying or having pills around; (4) *LAI removed adolescents ’ fear of missing pills due to pill fatigue and pill burden*, relieving anxieties and pressure of the consequences of not taking them, improving their general wellbeing and outlook on life. For these adolescents, the pain and minor side effects experienced with injections were inconsequential compared to the burden of taking pills daily.

**Conclusions: **Between oral and LAI ART, adolescents much preferred LAI ART. More research is needed to understand the long‐term use of LAI and what new challenges might emerge, acknowledging that these adolescents had recently started using LAI.

### OAE0402 improving advanced hiv disease identification among clients failing antiretroviral therapy: an implementation partner‐led initiative in Uganda

A. Nuwagira ^1^, R. Kirungi^1^, V. Nabitaka^1^, L. Kabunga^1^, J. Batusa^1^, W. Eigege^2^, I. Amamilo^2^, J. Conroy^2^, C. Amole^2^, P. M. Namuwenge^3^, V. Kasone^4^, J. Kyokushaba^4^

^1^Clinton Health Access Initiative, HIV Care and Treatment, Kampala, Uganda, ^2^Clinton Health Access Initiative, Boston, United States, ^3^Ministry of Health, AHD/TB Program, Kampala, Uganda, ^4^National Health Laboratory and Diagnostic Services, Kampala, Uganda

**Background: **CD4 testing is the gateway to identifying advanced HIV disease (AHD) for both newly diagnosed clients with HIV and those failing treatment (non‐suppressed people on antiretroviral therapy). While access to baseline CD4^+^ testing for newly diagnosed people has improved over the years with the expansion of CD4^+^ testing to include VISITECT point‐of‐care testing, CD4^+^ testing among failing clients has remained unacceptably low.

**Description: **Between October 2022 and September 2023, the Uganda Ministry of Health (MOH) with support from partners implemented quality‐improvement initiatives aimed at addressing gaps in the delivery of the AHD package of care implementation among failing clients.

Key interventions implemented included healthcare workers (HCWs) training on the identification and treatment of AHD, data management and reporting, commodity inventory management and formation of facility‐level AHD focal teams. In addition to this, sub‐national implementing partners integrated CD4^+^ testing into targeted community HIV services using a device‐free CD4^+^ testing platform. The cascade was monitored on a bi‐weekly basis to measure the impact on key indicators.

**Lessons learned: **​Access to CD4^+^ testing among the failing clients increased from 42.8% before the intervention, to 60% by the end of the quality improvement initiative.

**Conclusions/Next steps: **AHD screening services among failing people is critical to the reduction of AIDS‐related deaths. However, more effort such as integration of AHD screening services into existing community outreach programmes, HCW capacitation, improved commodity inventory management, and optimized reporting is needed to optimize CD4^+^ testing among failing people.

**Figure**. OAE0402

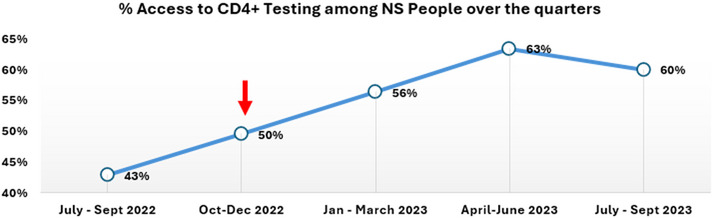

### OAE0403 Cost‐effectiveness simulation of implementing a point‐of‐care test package for opportunistic infections for people with advanced HIV in Mexico City's reference centres: preliminary analysis of the PREVALIO‐CDMX study

A. Camiro‐Zuñiga ^1^, A. Martin Onraet^1^, V. H. Ahumada Topete^2^, Á. López Íñiguez^3^, E. Sienra Iracheta^4^, X. A. Flores Andrade^2^, A. P. Rodríguez Zulueta^4^, M. Aranda Audelo^2^, A. B. Peralta Prado^2^, C. Pérez Jiménez^1^, J. G. Sierra Madero^3^

^1^Instituto Nacional de Cancerologia, Infectious Diseases, Mexico City, Mexico, ^2^Instituto Nacional de Enfermedades Respiratorias, Infectious Diseases, Mexico City, Mexico, ^3^Instituto Nacional de Ciencias Médicas y Nutrición Salvador Zubirán, Infectious Diseases, Mexico City, Mexico, ^4^Hospital General Dr. Manuel Gea Gonzalez, Infectious Diseases, Mexico City, Mexico

**Background: **Opportunistic infections (OIs) remain the main cause of death in people living with HIV in Latin America. Point of care tests (POC) represent an opportunity for timely diagnosis and reduce related costs.

**Methods: **Preliminary analysis of the retrospective data of the PREVALIO‐CDMX study (NCT05685641), which is being conducted in four reference centres for HIV treatment in Mexico City. We included data of all individuals with advanced HIV disease (<200 CD4 or suspected AIDS‐defining disease) that presented to care from September 2022 to September 2023, which underwent standard screening for tuberculosis (TB), disseminated histoplasmosis (DH) and cryptococcal disease (CD) during their routine care. We determined standard packages for related costs of care for the management of these OIs. We calculated time until OI treatment initiation using day 0 as the moment in which the individual presented to the participating centre, care provided during this time lapse was included in cost calculations. The simulated intervention was the centre‐wide implementation of first‐day POC tests on new HIV diagnosis, specifically lateral flow tests for urinary lipoarabinomannan, urinary histoplasma antigen and serum Cryptococcus antigen. We identified potential savings on spared hospitalization days and exempted further tests. We constructed Kaplan‐Meier curves to assess differences in time to OI treatment initiation.

**Results: **We included 282 participants, with a CD4 count median of 35 (17−83.5) cells/ml. Median time to OI treatment initiation was 3 (1−4) days. The simulation predicted a net reduction of costs of 320 USD/person, representing an average reduction of 12.49% and a median reduction in time to treatment initiation of 3 days (*p* = 0.002).

**Conclusions: **Optimal implementation of same‐day POC tests has the potential to reduce attention costs of individuals with advanced HIV disease and reduce hospital stays due to diagnostic delays. The potential impact of this intervention could be exponentiated if applied in the first level of care.

**Figure**. OAE0403

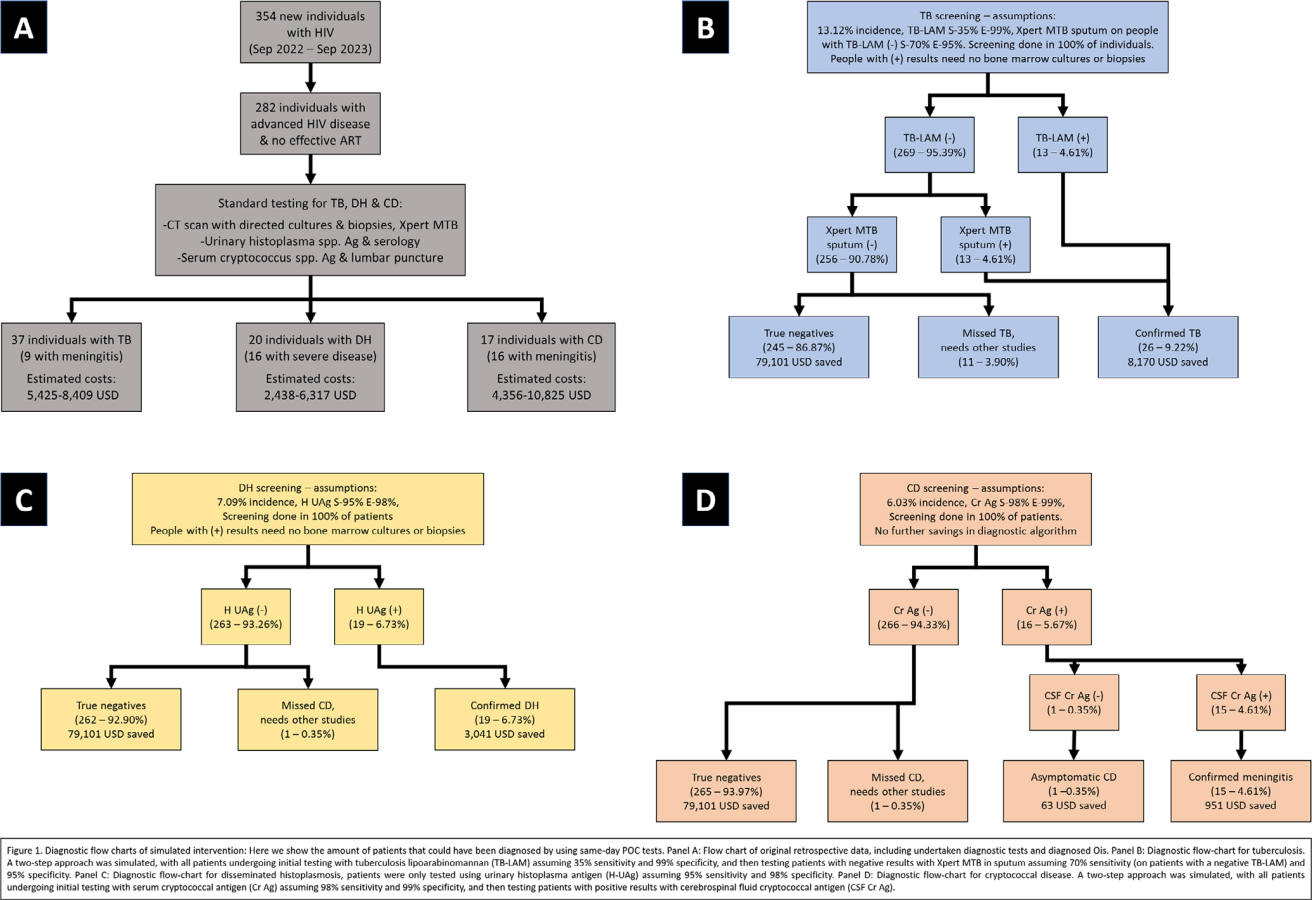

### OAE0404 Clinical impact and cost‐effectiveness of improving access to cryptococcal meningitis diagnostics and treatment in Malawi

M. Feser ^1,2^, T. Maphosa^3^, A. Shroufi^4^, A. Rangaraj^5^, V. R. Talbot^2^, Y. Qian^2^, E. Matiya^3^, N. Ford^5^, R. Nyirenda^6^, A. Phillips^7^, K. A. Freedberg^2,8,9,10^, A. Tiam^11^, E. P. Hyle^2,8,9,12^

^1^University of North Carolina at Chapel Hill, Gillings School of Global Public Health, Chapel Hill, United States, ^2^Massachusetts General Hospital, Medical Practice Evaluation Center, Boston, United States, ^3^Elizabeth Glaser Pediatric AIDS Foundation Malawi, Lilongwe, Malawi, ^4^Global Fund to Fight AIDS, Tuberculosis and Malaria, Geneva, Switzerland, ^5^World Health Organization, Department of Global HIV, Hepatitis, and Sexually Transmitted Diseases, Geneva, Switzerland, ^6^Malawi Ministry of Health, Directorate of HIV, STI and Viral Hepatitis, Lilongwe, Malawi, ^7^University College London, London, United Kingdom, ^8^Massachusetts General Hospital, Department of Medicine, Division of Infectious Diseases, Boston, United States, ^9^Massachusetts General Hospital, Division of General Internal Medicine, Boston, United States, ^10^Harvard T. H. Chan School of Public Health, Department of Health Policy and Management, Boston, United States, ^11^Elizabeth Glaser Pediatric AIDS Foundation, Washington, D.C., United States, ^12^Harvard Medical School, Boston, United States

**Background: **Cryptococcal meningitis (CM) causes 13−20% of deaths among PLHIV globally. In sub‐Saharan Africa, access to WHO‐preferred regimens for everyone in need remains limited. We modelled the cost‐effectiveness of improving access to CM diagnostics and treatment among adult PLHIV initiating HIV care with a positive serum CrAg test result in Malawi.

**Methods: **We used the CEPAC‐I model to evaluate nine strategies singly and in combination: (1) *Status quo* (CM treatment if diagnosed with CM and pre‐emptive fluconazole otherwise); (2) *LP for asymptomatic CrAg+*; (3) *Semi‐quantitative CrAg (CrAgSQ) for asymptomatic CrAg+* to diagnose CM without LP; (4) *Increased 5FC*; and (5) *Liposomal amphotericin B (LAmB) use* (Table 1A). We simulated a cohort with mean (SD) CD4 27/ml (30/ml), age 37 y (10 y), 51% female; 15% had symptomatic CM, 48% asymptomatic CM and 37% asymptomatic cryptococcemia. Model outcomes included 1 y survival, lifetime quality‐adjusted life years (QALYs) and costs, and incremental cost‐effectiveness ratios (ICER, $/QALY); we considered $640/QALY as the cost‐effectiveness threshold. Sensitivity analyses of key parameters were performed.

**Table 1:** OAE0404

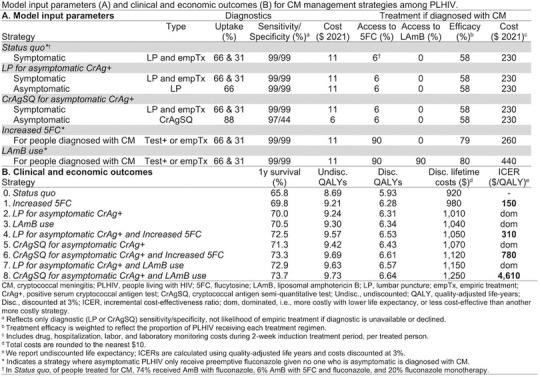

**Results:** *Status quo* would result in 66% 1‐y survival, 8.69 QALYs and $930/person (Table 1B). *LP for asymptomatic CrAg+ and Increased 5FC* would be cost‐effective: +10% 1‐y survival, +0.89 QALYs, +$130/person (ICER, $310/QALY). *CrAgSQ for asymptomatic CrAg+ and Increased 5FC* would increase QALYs but not be cost‐effective (ICER $780/QALY) due to more PLHIV without CM treated for CM given low test specificity. *LAmB use* could be cost‐effective if it were substantially less costly than current prices. Results were sensitive to LP uptake, CrAgSQ specificity, efficacy of pre‐emptive fluconazole and CM treatments, and proportion of asymptomatic PLHIV with CM.

**Conclusions: **Concurrently improving access to CM diagnostics and 5FC for symptomatic and asymptomatic PLHIV with CM would improve clinical outcomes and be cost‐effective in Malawi and similar settings. Improving access to LAmB could further improve outcomes and be cost‐effective at lower prices.

### OAE0405 Link between poverty and HIV viral load non‐suppression among PLHIV

R. W. Mukondwa ^1^, A. Ayer^2^, K. Takarinda^1^, T. Makoni^3^, C. Aviles‐Guaman^2^, M. Hudson^2^, N. West^2^, K. Webb^1^, P. Shete^2^

^1^Organization for Public Health Interventions and Development (OPHID), Programs, Harare, Zimbabwe, ^2^Center for Tuberculosis, University of California San Francisco (UCSF), Division of Pulmonary and Critical Care Medicine, San Francisco, United States, ^3^Zimbabwe National Network of People Living with HIV (ZNNP+), Programs, Harare, Zimbabwe

**Background: **HIV disproportionately impacts the impoverished. Social protection interventions (SPIs) including cash and food transfers may interrupt this cycle of poverty and disease. The objective of this study was to identify the relationship between poverty, social protection interventions and HIV viral load (VL) in 15 districts of Zimbabwe in a programmatic setting.

**Methods: **We conducted an exploratory, sequential mixed methods analysis. Quantitative analyses utilized retrospective cross‐sectional data collected from January to July 2023 from client satisfaction surveys (CSS) among adults living with HIV (PLHIV) (> 18 years) on antiretroviral therapy (ART). We performed descriptive analyses and generalized estimated equations to evaluate relationships between multidimensional poverty, SPIs and VL non‐suppression (>/ = 1000 copies/ml). Between August and September 2023, we conducted semi‐structured in‐depth interviews (IDIs) (*n* = 25) with adults (>18 years) with a history of accessing SPIs. IDIs were audio recorded, transcribed, translated and analysed using the framework method.

**Results: **Among 13,722 PLHIV in ART care completing the CSS, 8971 (65.4%) were female. Median age of the respondents was 44 years (interquartile range [IQR]: 36−52 years). Nearly half (*n* = 6095; 44.4%) of respondents were multidimensionally poor. An additional 3471 (25.3%) were vulnerable to multidimensional poverty, 5894 (43%) lacked food, yet only 2515 (18%) had ever received SPIs. The majority (1283 [51.3%]) of SPI recipients received educational assistance. Poverty was associated with HIV VL non‐suppression (relative risk [RR] = 1.55; 95% confidence interval [CI]: 1.13, 2.13). SPI receipt was also associated with HIV VL non‐suppression (RR = 1.67; [95% CI: 1.07, 2.62]). Qualitative findings showed that despite significant need, PLHIV in ART care had limited information about types of SPIs available and how to apply to the programmes. Further, participants described their experiences of poverty, with SPI providing a fragile support system for access to food and basic needs.

**Conclusions: **SPI receipt is limited among PLHIV in Zimbabwe, despite frequently reported poverty and food insecurity. In this context, SPIs may serve as a surrogate for socio‐economic vulnerability and appear insufficient to alleviate its effects. There may be a mismatch in SPIs (educational assistance) with individual needs (food insecurity) among this population of PLHIV, signalling importance of concordance in SP interventions with vulnerabilities experienced.

### OAE1202 Uptake of HIV post‐exposure prophylaxis in private retail pharmacies in Kenya: early findings from the Pharm PrEP study

P. Ong'wen ^1^, S. Roche^2^, V. Omollo^3^, T. Kaireithi^4^, L. Juma^3^, C. Kiptinness^4^, P. Otieno^3^, G. Rota^3^, M. Anyona^1^, P. Banerjee^2^, M. Asewe^3^, B. Rono^3^, E. Gichuru^4^, K. Harkey^2^, R. C. Malen^2^, E. Irungu^1^, D. Were^1^, E. A. Bukusi^3,5^, K. Ngure^4,5^, K. F. Ortblad^2^, Pharm PrEP cRCT Team

^1^Jhpiego, Nairobi, Kenya, ^2^Fred Hutchinson Cancer Center, Seattle, United States, ^3^Kenya Medical Research Institute, Kisumu, Kenya, ^4^Partners in Health and Research Development, Thika, Kenya, ^5^University of Washington, Seattle, United States

**Background: **HIV post‐exposure prophylaxis (PEP) is an effective strategy to reduce the risk of HIV acquisition after recent exposure, but has been underutilized in many HIV prevention programmes. In Kenya, PEP is freely available in most public health clinics that offer HIV services; however, it is primarily used for occupational HIV exposure. We report early findings on PEP uptake at 45 intervention pharmacies delivering HIV services for the first time as part of the Pharm PrEP cluster‐randomized control trial (cRCT).

**Methods: **As part of the Pharm PrEP cRCT, trained pharmacy providers at 45 private pharmacies in central and western Kenya are offering subsidized PEP and PrEP services to clients seeking sexual health products. Eligible PEP clients are ≥16 years and self‐report: unknown/negative HIV status, a potential HIV exposure (e.g. condom break) in <72 hours and no signs of acute HIV acquisition. Upon enrolment, participants undergo HIV testing and, if negative, are dispensed a 30‐day PEP supply. Pharmacy providers record client demographics, HIV test results and dispensing details in an electronic medical record.

**Results: **From July 2023 to mid‐January 2024, 881 of 1534 clients (57%) seeking pharmacy‐based HIV services initiated PEP (median: 7 PEP clients/pharmacy, IQR 3−13). The median age of PEP clients was 27 years (IQR: 23−32), with 59% (523/881) being male and 60% (531/881) unmarried. The majority (98%, 849/881) of clients identified as the general population and 61% (538/811) reported having casual sex partners. The most common exposures leading to PEP use were condom break/condomless sex (85%, 747/811), sexual assault (3%, 23/881) and shared needles (2%, 20/881). In addition, clients had ongoing HIV risk behaviours that would warrant PrEP use including, inconsistent condom use (64%, 561/881), sexual partners of unknown HIV status (51%, 449/881) and multiple sex partners (48%, 420/881).

**Conclusions: **Early data from the Pharm PrEP cRCT suggest that PEP services are in high demand at private pharmacies in Kenya and could be delivered in partnership with public support. Additionally, our findings suggest that integrating PEP with PrEP services in HIV prevention programmes might help better serve individuals with diverse HIV prevention needs, bringing us closer to ending the AIDS epidemic.

### OAE1203 Knowledge, awareness, feasibility and acceptability of long‐acting Cabotegravir for HIV prevention: results from the SEARCH Dynamic Choice HIV prevention trial

E. Kakande ^1^, L. Balzer^2^, J. Kabami^1^, J. Ayieko^3^, G. Chamie^4^, N. Sutter^4^, H. Sunday^1^, M. Nyabuti^3^, J. Litunya^3^, C. Camlin^4^, J. Johnson‐Peretz^4^, J. Temple^2^, G. Lavoy^1^, C. Koss^4^, M. Bacon^5^, M. Czarnogorski^6^, M. Petersen^2^, M. Kamya^1,7^, D. Havlir^4^, SEARCH Collaboration

^1^Infectious Diseases Research Collaboration, Kampala, Uganda, ^2^University of California, Berkeley, United States, ^3^Kenya Medical Research Institute, Nairobi, Kenya, ^4^University of California, San Francisco, United States, ^5^National Institute of Health, Bethesda, United States, ^6^Viiv Healthcare, Washington, United States, ^7^Makerere University, Kampala, Uganda

**Background: **Injectable Cabotegravir (CAB‐LA) is highly effective for HIV prevention, but real‐world implementation studies among men and women in Africa are lacking. We assessed knowledge, awareness, feasibility and acceptability among participants who used CAB‐LA for prevention in the ongoing SEARCH Dynamic Choice HIV prevention (DCP) randomized implementation study in rural Uganda and Kenya.

**Methods: **The SEARCH DCP study enrolled women and men aged ≥15 years with self‐assessed risk for HIV acquisition. The intervention arm included structured product choice (oral PrEP, PEP or injectable CAB‐LA), with flexibility to switch between products based on changes in participant risk or preferences over 48 weeks of follow‐up. CAB‐LA injections were provided at Ministry of Health clinics. Quantitative surveys were completed by participants who chose and initiated CAB‐LA at the time of initiation and after 24 weeks of CAB‐LA use.

**Results: **Of 487 intervention arm participants, 274 (56%) started CAB‐LA during follow‐up (183 women, 91 men; 79 youth aged 15−24 years); of these, 198 (72%) used CAB‐LA for ≥24 weeks. At initiation, 64% chose CAB‐LA because it was easier to take an injection and 49% because of difficulty remembering to take oral pills. Among youth initiators, 42% chose CAB‐LA because they did not want someone to see them taking pills and 22% because partners/friends would not let them take pills. At CAB‐LA initiation, 99% of participants had basic to no knowledge of CAB‐LA, consistent across gender and age groups. Awareness, acceptability and feasibility were high at 24 weeks (Table).

**Table:** Table. OAE1203: Awareness, acceptability and feasibility of CAB‐LA in SEARCH HIV Dynamic Choice Prevention

	Question	Response	Overall	Women	Men	15−24 years	25+ years
**Awareness** of CAB‐LA at Initiation versus Week‐24	“How many of your friends know about CAB‐LA?”	None of my friends know about CAB‐LA	91% versus 25%	92% versus 28%	89% versus 19%	91% versus 30%	91% versus 23%
**Acceptability** at Week‐24	“What is your level of satisfaction for using CAB‐LA?”	Satisfied to Very Satisfied	97%	98%	96%	100%	96%
**Acceptability** at Week‐24	“What is the likelihood of you recommending CAB‐LA to a friend?”	Likely to Extremely Likely	95%	95%	94%	95%	95%
**Feasibility** at Week‐24	“How easy was it to take CAB‐LA?”	Easy to Very Easy	95%	95%	94%	98%	93%

**Conclusions: **In rural Uganda and Kenya, over half of participants in the SEARCH DCP trial who were offered choice of oral PrEP/PEP or CAB‐LA chose and started CAB‐LA during the first 48 weeks. CAB‐LA was a popular choice for men and women and was feasible to deliver with a high level of satisfaction.

### OAE1204 Pharmacy‐based delivery of long‐acting injectable PrEP in Kenya: provider, client and key stakeholder perspectives on potential challenges and opportunities

S. D. Roche ^1^, K. Kamolloh^2^, N. Thuo^3^, M. Opiyo^2^, V. Ogello^3^, A. Odira^2^, E. Owidi^3^, P. Ochwal^2^, M. Hewa^2^, L. Adiema^2^, F. Mogaka^2^, V. Omollo^2^, R. Malen^1^, K. Harkey^1^, J. Stewart^4^, E. A. Bukusi^2,5^, K. F. Ortblad^1^, K. Ngure^3,6^

^1^Fred Hutchinson Cancer Center, Public Health Sciences, Seattle, United States, ^2^Kenya Medical Research Institute, Kisumu, Kenya, ^3^Partners in Health and Research Development, Thika, Kenya, ^4^University of Minnesota, Minneapolis, United States, ^5^University of Washington, Seattle, United States, ^6^Jomo Kenyatta University of Agriculture and Technology, Nairobi, Kenya

**Background: **Four sub‐Saharan African countries have approved long‐acting injectable PrEP for HIV prevention. As countries decide where to make injectable PrEP available for maximal impact, private pharmacies are one venue under consideration. To understand the potential barriers and facilitators to delivering injectable PrEP via private pharmacies in Kenya, we conducted qualitative formative research with pharmacy providers, clients and key stakeholders.

**Methods: **From July to September 2023, we interviewed pharmacy providers, pharmacy clients and key stakeholders involved in HIV policymaking, regulation and programme implementation in Central and Western Kenya. We purposively sampled pharmacy providers and clients both with and without prior experience delivering or receiving, respectively, oral PrEP services at a pharmacy. Our semi‐structured interview guide was informed by the Updated Consolidated Framework for Implementation Research (CFIR). We analysed transcripts thematically.

**Results: **We interviewed 16 pharmacy providers, 25 pharmacy clients and 9 key stakeholders. Each participant group was ∼50% female, and median age was 25 (IQR 23−29) among clients and 37 (IQR 34−41) among providers and stakeholders. Overall, participants supported the idea of pharmacy‐based injectable PrEP delivery, with some recommending it first be rolled out at pharmacies with medical providers on staff. Providers and key stakeholders called for updated guidelines specifying the circumstances under which pharmacy providers can deliver injectable PrEP (CFIR: policy and law). Across all groups, some participants expressed concern about unqualified providers, and many called for the development of an injectable PrEP certificate programme—possibly modelled after existing courses on injectable contraception delivery—to ensure competency in maintaining the cold chain; counselling; administering injections; managing drug reactions; assessing side effects; and conducting pharmacovigilance (CFIR: access to knowledge and information). To keep injectable PrEP affordable for clients and economically viable for pharmacies, a few recommended price controls or government subsidies (CFIR: innovation cost; financing). Some also noted the need for a shared health information system for client tracking (CFIR: information technology infrastructure) and reliable systems for safe disposal of used sharps (CFIR: compatibility).

**Conclusions: **Stakeholders of injectable PrEP are interested in pharmacy‐based delivery. Additional implementation research is needed to identify and test specific capacity‐building and integration strategies.

### OAE1205 Preferences for services delivering pre‐exposure prophylaxis among sexually active adolescent girls and young women: a discrete choice experiment in Zimbabwe

V. Cambiano^1^, P. P. Indravudh^2^, K. Chidhanguro^3^, W. Murenjekwa^3^, G. Ncube^4^, A. Copas^5^, F. Cowan^3,6^, J. Dirawo^3^, E. Matsikire^3^, A. Mpofu^7^, O. Mugurungi^4^, A. Phillips^1^, I. Taramusi^8^, E. Sibanda ^3,6^

^1^University College London, Institute for Global Health, London, United Kingdom, ^2^London School of Hygiene and Tropical Medicine, London, United Kingdom, ^3^Centre for Sexual Health and HIV/AIDS Research (CeSHHAR) Zimbabwe, Harare, Zimbabwe, ^4^Department of AIDS and TB Unit, Ministry of Health and Child Care, Harare, Zimbabwe, ^5^University College London, Institute for Global Health and the MRC Clinical Trials Unit, London, United Kingdom, ^6^Liverpool School of Tropical Medicine, Department of International Public Health, Liverpool, United Kingdom, ^7^National AIDS Council (NAC), Harare, Zimbabwe, ^8^UNAIDS UCO Zimbabwe, Harare, Zimbabwe

**Background: **Adolescent girls and young women (AGYW) in Zimbabwe are disproportionally affected by HIV. As pre‐exposure prophylaxis (PrEP) is rolled‐out in Zimbabwe, we assessed preferences for services delivering PrEP among sexually active (SA‐AGYW; self‐reported sex in the last year) in Zimbabwe and identify key drivers of demand for services delivering PrEP.

**Methods: **We conducted a discrete choice experiment (DCE) among SA‐AGYW (aged 15−24) recruited using a respondent‐driven sampling survey in six urban and peri‐urban districts in May−July 2023. The DCE was designed based on a literature review and qualitative studies and administered face‐to‐face using pictorial illustrations. Data were analysed using a conditional logit model.

**Results**: Nine hundred AGYW completed the DCE. There was a general preference for PrEP programmes over not receiving PrEP (see Figure). Participants had strong positive preferences for (in order of strength): a programme to support parents having a more positive attitude about sexual and reproductive health services (SRHS); a friendly attitude by dispensing health workers; collecting PrEP from a community health worker (CHW) or the local public sector clinic compared with the pharmacy; and for injectable PrEP compared with oral PrEP. Participants reported negative preferences for: vaginal ring for PrEP (compared with oral PrEP), longer distance to the venue for PrEP collection; higher fees to access PrEP including the consultation; and longer time spent at the PrEP collection venue. There was no evidence of preference for the integration of the PrEP dispending venue.

**Figure**. OAE1205

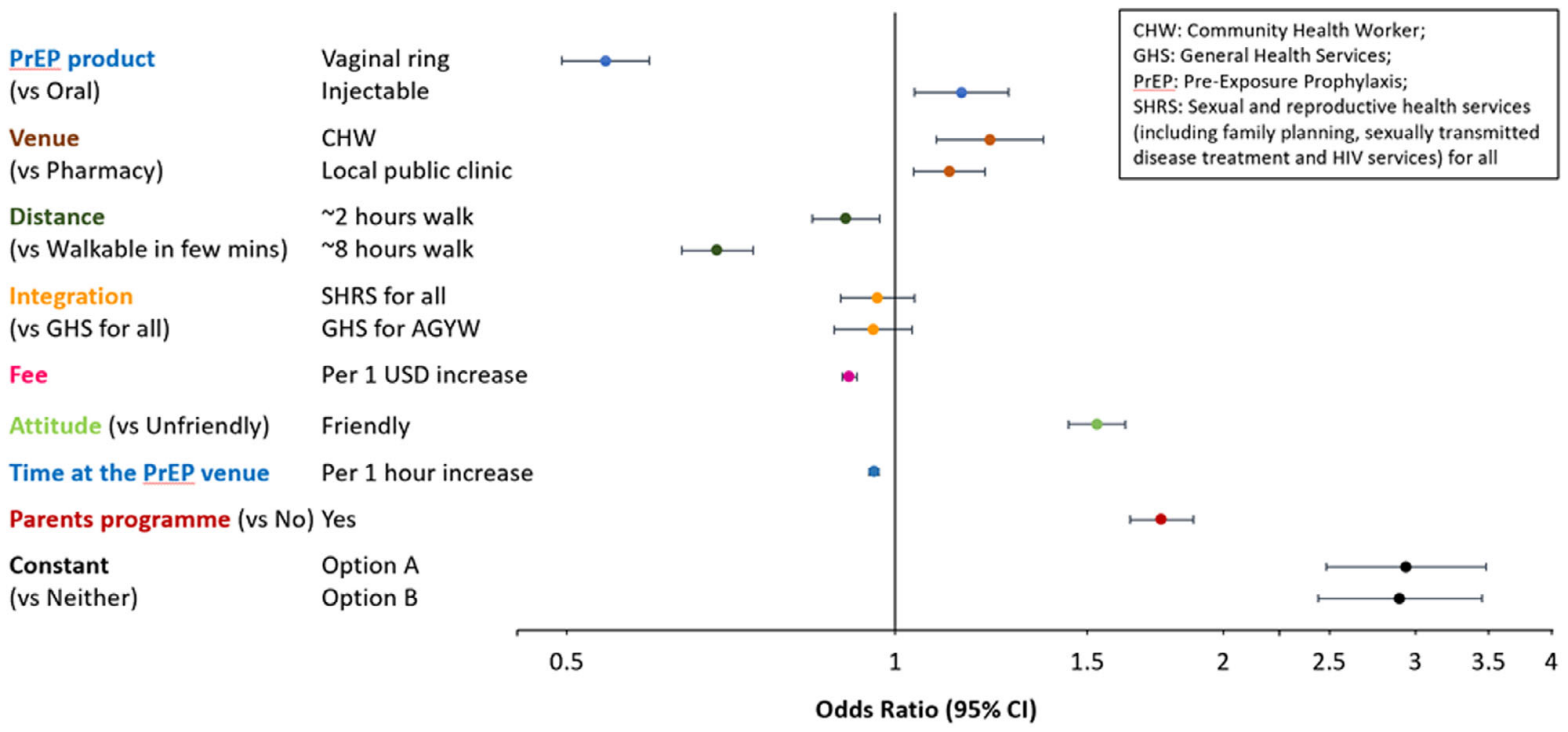

**Conclusions: **PrEP programmes can be optimized to reach sexually active AGYW, if PrEP is provided by friendly CHW or local public sector clinic at low‐cost for the user, at venues within walking distance, with short waiting times and with the choice of injectable PrEP. Programmes should be accompanied by activities for building parental support for SRHS.

### OAE2002 Promoting exploratory community monitoring of the incidence of tuberculosis and TB/HIV comorbidities in health centres with the incorporation of Geographic Information Systems in Caracas, Venezuela

A. Nieves ^1^

^1^Acción Ciudadana Contra el SIDA (ACCSI), Dirección Ejecutiva, Caracas, Venezuela, Republic of Bolivarian

**Background: **The Venezuelan humanitarian crisis caused the collapse of the health system. There is an absence of official reports on the epidemiology of tuberculosis and TB/HIV comorbidities in the COVID‐19 context. Health authorities do not provide information on the situation of these diseases and do not authorize NGOs to monitor medical services that care for persons with TB and comorbidities. From 2019 to the present, the Global Fund pays for 100% of ARV and TB medications and supplies for persons with these diseases.

In response, Acción Ciudadana Contra el SIDA (ACCSI) implemented a project financed by The Global Fund with these OBJECTIVES: (1) Generate information through community monitoring with the participation of PLHIV in TB and comorbidity services in five hospitals in Caracas; (2) Incorporate GIS (ARCGIS Online software) and KoboToolbox to collect data (cell phone use) in health services, to community monitoring; (3) Carry out evidence‐based advocacy to decision‐makers.

**Description: **Between November 2022 and April 2023, community monitoring was carried out in a difficult environment as it was affected by the aforementioned obstacles. However, PLHIV participated in stages of community monitoring (coordination, collection, documentation, systematization, analysis and dissemination) in health services of five hospitals, obtaining information on comorbidity in Caracas.

**Table:** Table. OAE2002

Indicator	Nov 2022	Dec 2022	Jan 2023	Feb 2023	Marc 2023	Apr 2023	TOTAL
Total number of TB cases	123	81	81	90	102	55	532
No. of report TB cases	85	44	40	46	34	15	264
No. new TB cases	38	37	41	44	68	40	286
Total number of TB/HIV comorbidity cases	14	11	13	15	24	17	94
No. HIV people notified of TB	1	7	7	7	1	0	23
No. people with HIV new TB cases	13	4	6	8	23	17	71

**Lessons learned: **The project findings are:

Two dashboards were produced that make visible the results of community monitoring, situation of the last 10 years of TB/HIV comorbidity and georeferencing of 49 TB consultations and pharmacies in 24 Venezuelan states, with public access to persons:

Dashboard 1: https://lsigma.maps.arcgis.com/apps/dashboards/59c1e0c604b54fa4850cacd83fb5f14b

Dashboard 2: https://www.arcgis.com/apps/dashboards/10d8877cd51a45ccb9e6b565fd988138

GIS links health and geography to analyse people's access to health services and effective responses to the Venezuelan health emergency, among the lessons learned.

**Conclusions/Next steps: **The incorporation of GIS in community monitoring professionalized the work of NGOs, by improving the processes of collection, analysis and documentation and of quality data, whose evidence is useful for advocacy to decision‐makers and contribute to the improvement of international cooperation assistance for Venezuela.

### OAE2003 Community‐led monitoring goes beyond evaluating essential health components, it also actively addresses issues of rights and social justice in Indonesia and Manipur, India

G. Khwairakpam ^1^, C. Thomas^2^, E. Lankiewicz^3^, R. Nalinikanta^4^

^1^TREAT Asia/amfAR, Bangkok, Thailand, ^2^Peduli Hati Bangsa, Jakarta, Indonesia, ^3^Andelson Office of Public Policy/amfAR, Washington, D.C., United States, ^4^Community Network for Empowerment, Manipur, India

**Background: **Community‐led monitoring (CLM) is a routine, ongoing cycle of collecting data on community‐designed indicators, identifying gaps, generating actionable evidence and advocating to improve essential components of healthcare services. It is gaining recognition among stakeholders and donors. Our project, CLM in Asia, is one of the first digitalized CLM projects, monitoring the essential components of HIV, hepatitis B (HBV) and hepatitis C (HCV) through care recipients.

**Description: **The project was initiated in 2021 by two community‐based organizations, Peduli Hati in Jakarta, Indonesia and Community Network for Empowerment (CoNE) in Manipur, India, with amfAR's TREAT Asia programme, monitoring 12 health facilities comprised of local district to referral hospitals in select provinces providing public HIV and viral hepatitis services. The indicators developed by the local partners were based on current national guidelines and address the essential components of the accessibility, acceptability, availability, affordability and quality framework covering HIV, HBV and HCV. From April 2023, the project moved to a digitalized platform with publicly available information on the project details, tools and a real‐time data dashboard.

**Lessons learned: **Post digitalization from April to December 2023, we reached out to 1593 care recipients with 76% between 25 and 49 years of age; 71% male, 28% female, 0.56% transgender. Respondents were receiving care for HIV (62%), HBV (3%) and HCV (34%). Besides monitoring essential care components and resolving bottlenecks, we assisted 174 people in HIV treatment monitoring, 19 linked to HBV treatment, 37 for HCV treatment. CLM team members resolved seven episodes of medicine shortages or stock‐outs. Interactions with care recipients resulted in successfully linking 20 children living with or affected by HIV to public education programmes, restoring the right to education of a child living with HIV, home delivery of antiretrovirals for the visually challenged, addressing gender‐based violence and relocating seven people to antiretroviral centres which were more convenient for them.

**Conclusions/Next steps: **CLM enables care recipients to have a say in improving the essential components of healthcare services they receive. It not only addresses care bottlenecks at facilities, but also serves as a mechanism to link people to social security schemes and bring about social justice.

### OAE2004 Improving uptake of tuberculosis testing using urine Lipoarabinomannan among children with advanced HIV disease: outcomes of a quality improvement initiative in southern Nigeria

O. Onwah ^1^, E. Nwanja^1^, O. Toyo^1^, U. Akpan^1^, M. Unimuke^1^, E. Ezieke^1^, C. Okolo^1^, C. Nwangeneh^2^, O. Anika^2^, U. Onwuzuruigbo^1^, B. Oyawola^3^, K. Omo‐Emmanuel^3^, D. Ogundehin^3^, E. James^3^, C. Obiora‐Okafo^3^, A. Idemudia^3^, C. Nwadike^3^, K. Kakanfo^3^, B. Pius^3^, B. Onimode^3^, O. Asaolu^3^, A. Bashorun^4^, A. Gambo^5^, J. Pius^3^, O. Oyelaran^3^, R. Goldstein^3^, O. Onyedinachi^1^, A. Adegboye^1^, A. Eyo^1^

^1^Excellence Community Education Welfare Scheme, HIV Prevention Care and Treatment, Uyo, Nigeria, ^2^Family Health International (FHI 360), Abuja, Nigeria, ^3^Office of HIV/AIDS and TB, United States Agency for International Development (USAID), Abuja, Nigeria, ^4^National AIDS, Sexually Transmitted Infections Control and Hepatitis Programme (NASCP), Federal Ministry of Health, Abuja, Nigeria, ^5^National Agency for the Control of AIDS (NACA), Abuja, Nigeria

**Background: **Diagnosis of tuberculosis (TB) in children living with HIV (CLHIV) can be challenging using conventional diagnostics. Lateral flow urine lipoarabinomannan (LF‐LAM) assay is recommended for TB diagnosis among CLHIV with advanced HIV disease (AHD) in Nigeria, with simultaneous GeneXpert mycobacterium TB/rifampicin (MTB/RIF) tests when sputum or stool samples can be produced. The PEPFAR/USAID‐funded ECEWS ACE‐5 project implemented this recommendation using a quality implementation framework that included the development of a simplified algorithm for TB testing using LF‐LAM, inventory optimization for LF‐LAM and weekly data reviews. This study assessed the outcomes of this quality implementation approach on the uptake of TB tests using LF‐LAM among children with AHD in Southern Nigeria.

**Methods: **This was a retrospective cohort analysis using electronic medical records of ART‐naïve children (<15 years old) diagnosed with AHD using the WHO criteria, from October 2022 to March 2023 across 153 health facilities in Akwa Ibom and Cross River States, Nigeria. Results were disaggregated by age and sex. Uptake of LF‐LAM tests (proportion of children with AHD who were tested using LF‐LAM) and proportion of LF‐LAM tests that were positive were compared before (October−December 2022 [Period‐1]) and during (January−March 2023 [Period‐2]) the intervention, using chi‐square.

**Results: **In total, 215 children had AHD (M: 111, F: 104), with 60% (129/215) identified in Period‐2. Median age was 2 years (IQR 1−3). Overall, 45.6% (98/215) of children with AHD were tested for TB using LF‐LAM, with 18% (18/98) testing positive. Of those who tested positive via LF‐LAM, 88.9% (16/18) were in Period‐2. Uptake of LF‐LAM tests was significantly higher in Period‐2 compared to Period‐1 (69.0% [89/129] vs. 10.5% [9/86] *p*<0.01), and the proportion of positive LF‐LAM tests was lower in Period‐2 than in Period‐1 (18.0% [16/89] vs. 22.2% [2/9] *p* = 0.75).

**Conclusions: **The uptake of TB testing using LF‐LAM for children with AHD improved in our setting following a quality implementation approach. Further investigation of factors affecting the uptake of TB testing using LF‐LAM in this sub‐population is recommended.

### OAE2005 Integrating hepatitis C virus self‐testing into HIV and harm reduction services as an approach towards HCV micro‐elimination among key populations and people living with HIV in Vietnam

B. Vu ^1^, K. Green^1^, M. Tran^2^, H. Nguyen^1^, K. Do^1^, L. Tran^1^, A. Tran^1^, G. Le^2^, C. Pham^1^, K. Granger^3^, H. Phan^4^, K. Nguyen^5^, L. Doan^4^, P. Cao^5^

^1^PATH, Hanoi, Viet Nam, ^2^CCIHP, Hanoi, Viet Nam, ^3^PATH, Washington DC, United States, ^4^Vietnam Administration of HIV/AIDS Control (VAAC), Ministry of Health, Hanoi, Viet Nam, ^5^Vietnam Administration of Medical Services (VAMS), Ministry of Health, Hanoi, Viet Nam

**Background: **Hepatitis C virus self‐testing (HCVST) is an innovative approach to accelerate progress towards HCV elimination goals. We conducted a cross‐sectional observational study to assess the acceptability and effectiveness of HCVST compared to routine HCV testing among key populations (KPs) and people living with HIV (PLHIV) in Hanoi and Ho Chi Minh City, Vietnam.

**Methods: **From September 2023 to January 2024, we engaged eight community‐based organizations (CBOs), six anti‐retroviral therapy and methadone maintenance treatment (MMT) public clinics, and four KP‐led private clinics in implementing community‐based, facility‐based, online and secondary distribution. Clients were offered the choice of an oral fluid‐based HCVST, or rapid HCV testing provided by CBOs and clinic staff (PL‐HCVT). Individuals with a positive HCV test were referred or linked to designated public and private clinics for HCV confirmatory testing and treatment initiation. Acceptability of HCVST was defined as the proportion of first‐time HCV testers utilizing the service, and effectiveness was measured by HCV positivity and treatment initiation rates.

**Results: **Of 2882 individuals recruited, 1834 opted for HCVST and 1048 opted for PL‐HCVT. The proportion of first‐time testers was significantly higher in HCVST compared to PL‐HCVT (67.6% vs. 59,1%), and particularly high in secondary distribution and community‐based HCVST compared to online and facility‐based HCVST (91.4% and 83.8% vs. 48.9% and 36.8%, respectively). Overall, HCV sero‐positivity rate was lower in HCVST than in PL‐HCVT (11.2% vs. 18.4%); however, it was higher in community‐based and facility‐based HCVST compared to secondary distribution and online HCVST (18.1% and 16.8% vs. 3.6% and 1.5%, respectively). HCV positivity rate was highest among PLHIV (27.6%), followed by MMT clients (26,4%), people who inject drugs (17.9%), sex partners (3.9%), female sex workers (2.8%) and men who have sex with men (0.3%). Of 399 HCV sero‐positive individuals detected, 206 were from HCVST, of which 92.7% received confirmatory testing, and 98.5% of those eligible initiated HCV treatment.

**Conclusions: **HCVST is an additional effective approach to increase uptake of HCV testing and treatment among KPs and PLHIV. HCVST is most effective in reaching unreached people and linkage to care through community‐based model, followed by facility‐based, secondary distribution and online.

### OAE2302 Impacts of community‐led monitoring on HIV service improvements: implementation in three provinces in Thailand

S. Sittikarn ^1^, N. Ketsuriyong^2^, P. Paoboonprung^3^, P. Rattakittvijun Na Nakorn^4^, A. Poonwanasatien^5^

^1^Caremat Foundation, Chiang Mai, Thailand, ^2^Stella Maris, Songkhla, Thailand, ^3^Saichon Network, Chonburi, Thailand, ^4^USAID, Bangkok, Thailand, ^5^FHI 360, Bangkok, Thailand

**Background: **The implementation of community‐led monitoring (CLM) by key populations and people living with HIV communities is still new in the Asia region and has few examples of successful implementation and improvements. We represent here CLM processes and impacts among stakeholders collaborating with the Thailand Ministry of Public Health and implemented in three provinces.

**Description: **Key‐population‐led health service providers involved in the CLM monitoring cycle engaged in data collection and analysis, stakeholder presentations, advocacy for service quality improvements and outcome monitoring. Between October 2022 and September 2023, 3248 responses were gathered online.

**Table:** Table. OAE2302

FINDINGS (% of responses)	IMPROVEMENT HIGHLIGHTS
**Chiangmai**	
50% lacked information on undetectable = untransmissible (U = U)	Provincial Health Office (PHO) promoted U = U messages, reducing stigma toward PLHIV at provincial media outlets
91.84% indicated limited number of PrEP‐offering facilities	PHO assisted all hospitals in Chiangmai to register as PrEP service facilities under National Health Security Office in 2024
**Chonburi**	
12.23% mentioned ARV clinics lacked privacy	Extended ARV clinic area and separated counselling room
21.58% mentioned no client queuing system in place	Implemented a queuing system at ARV clinic
**Chiangmai, Chonburi and Songkhla**	
18.9% mentioned providers lacked understanding of gender sensitivity and stigma and discrimination	Conducted a series of gender sensitivity training and outcome monitoring for hospital staff

**Lessons learned: **Lessons learned from the provincial CLM implementations included:

- Sharing CLM success stories and challenges among provinces promoted knowledge exchange, problem‐solving and continuous improvement of health service provision.
- CLM working groups are best positioned to drive positive changes in HIV service delivery, policy reforms, stigma reduction, capacity enhancement and access to accurate information within the HIV response.
- The launch of the CLM official Facebook page successfully promoted knowledge sharing and community engagement among implementers, thereby enhancing the programme's effectiveness and impact.

**Conclusions/Next steps: **CLM led by KP and PLHIV communities can enhance the quality of health services. In 2024, CLM will be expanded to three additional provinces: Chiang Rai, Ubonratchathani and Phitsanulok.

### OAE2303 Post‐hospitalization community home visit intervention to decrease mortality among adults with advanced or unsuppressed HIV in urban Zambia: pilot evaluation

C. Claassen ^1,2,3^, G. Muchanga^2^, M. Mujansi^2^, L. Mwango^4^, C. Bwalya^2,5^, K. Stoebenau^5^, C. Baumhart^1^, D. Malama^2^, J. Mukuka^2^, T. Daka^2^, B. Lindsay^1^, M. Mwitumwa^3^, N. Mbewe^3^, W. Mutale^6^, M. Vinikoor^3,7^

^1^University of Maryland Baltimore, Institute of Human Virology, Center for International Health, Education, and Biosecurity (Ciheb), Baltimore, United States, ^2^MGIC‐Zambia, Lusaka, Zambia, ^3^University Teaching Hospital, Lusaka, Zambia, ^4^Ciheb Zambia, Lusaka, Zambia, ^5^University of Maryland School of Public Health, Department of Behavioral and Community Health, College Park, United States, ^6^University of Zambia, Lusaka, Zambia, ^7^University of Alabama at Birmingham, Birmingham, United States

**Background: **HIV‐related mortality remains high in Zambia. Hospitalization often precedes death; post‐discharge mortality among PLWH reaches 20−40%. We evaluated a community health worker (CHW) model to provide post‐discharge support with the ultimate goal of mortality reduction.

**Methods: **We conducted a quasi‐experimental feasibility and acceptability study at two tertiary hospitals in Lusaka, Zambia, using the PRISM implementation science framework. Hospitalized PLWH with either CD4 <200 (i.e. advanced HIV) and/or HIV RNA >60 copies/ml, regardless of treatment history, were enrolled and followed for 6 months post‐discharge. Participants received a novel community intervention, based on formative qualitative work, consisting of a discharge summary card, CHW home visits within 7 days of discharge, and repeated every 2−4 weeks thereafter, and screening and referral for depression and unhealthy alcohol use. CHW visits were overseen by a physician‐clinical liaison officer team based at the discharging hospital. During visits, CHWs provided psychosocial and medication counselling, checked vital signs and made reminders for outpatient follow‐up.

**Results: **From 18 August 2023 to 22 January 2024, 100 study participants (median age, 39 years; 47% women; median CD4, 118 cells/mm^3^) were enrolled. To date, 86 (86%) received at least one home visit (31 within 1 week of discharge); of these, 36 (42%) had two or more visits; 32 (37%) received a discharge summary card; and 86 (100%) were screened for behavioural health problems. At 1 month, 86 (86%) were alive, 14 (14%) had died; 20 (20%) were readmitted based on concerns found during CHW home visits. Acceptability of CHW home visits among participants and caregivers was high. When available, 6‐month mortality data will be compared to a historical control group from the same hospitals.

**Conclusions: **A novel discharge model of care, involving enhanced discharge instructions, CHW home visits, and screening and referral for behavioural health problems, proved feasible and acceptable in urban Zambia. As post‐hospital mortality is so high and minimal/no transition of care programmes exist in African settings, CHW visits have the potential to reduce post‐discharge mortality among PLWH. Focusing on the peri‐discharge period can strengthen health systems as countries move into HIV epidemic control.

### OAE2304 Improvement on quality of healthcare services offered to people living with HIV at health facility in Cameroon through the community‐led monitoring mechanism

E. Sangong Rose ^1^, M. N. Mireille^1^, L. H. Shey^1^, A. Fabrice Yannick^1^, N. Gildas^1^, R. G. Bissai^2^, A. Ngong^2^, Z. Z. Akiy^2^, A. D. Nzaddi^2^, L. Ebiama^2^, J. Tchofa^2^

^1^Reseau Camerounais des Associations des Personnes vivant avec le VIH (RECAP+), Yaounde, Cameroon, ^2^USAID Cameroon, Yaounde, Cameroon

**Background: **Limited ownership of health policies by affected communities in sub‐Saharan Africa in general and Cameroon in particular remains a challenge in successful implementation of the health sector national strategic plan. With support from USAID, the Cameroon Network of Associations of PLHIV (RECAP+) is currently rolling out a CLM mechanism in Cameroon with the overall objective of improving access to free quality HIV/AIDS service offered at facility level while supporting the client‐centred approach.

**Description: **The CLM is being rolled out nationwide, in 326 health facilities by 38‐member organizations of RECAP+ and 169 rigorously trained site monitors who are mainly PLHIV. Activities include monthly data collection using two main questionnaires at facility level to inform 13 main indicators, community sensitization on HIV prevention and treatment access, psychosocial counselling and evidence‐based advocacy for change. The indicators monitored focus on treatment access barriers such as user fees elimination, stigma and discrimination, quality of psychosocial support and other HIV care‐related variables.

**Lessons learned: **Contributions of the CLM led to the Ministry of public health developing an HIV CLM guide in 2023 for use by organizations. The CLM also helped in improving community engagement in healthcare; in fiscal year 2023, monitors were able to sensitize 14,891 peers at facility‐level and 45,293 persons in the community on their rights, bringing back 41 lost to follow‐up (LTFU) peers to care. Confidentiality issues were resolved in eight health facilities and involved the creation of appropriate reception space for clients and serious warning to some health personnel, HIV commodities supplied in 16 facilities, 37 persons linked to the facility for HIV testing among which 8 new cases placed on treatment. Improvement in wait time in 10 facilities from 2 hours to 30 minutes and less involving actions such as the delocalization of viral load testing services and the use of customized tickets for ARV pickups. Thanks to the CLM, some health facilities received equipment such as centrifuges, microscopes and fridges from mayors to improve the quality of care.

**Conclusions/Next steps: **CLM remains one effective mechanism that governments can put in place to better engage the community and strengthen the health system.

### OAE2305 Bringing evidence to the table: community‐led monitoring to reduce human rights violations and violence against sex workers

L. Gumpo^1^, L. S. Rajabo^2^, J. Vilanculos^2^, A. Zandamela ^3^, S. A. Balsamo^4^, S. Bouwmeester^4^, I. van Beekum^4^

^1^Southern African Sex Workers Alliance (SASWA), Lusaka, Zambia, ^2^National Platform for Sex Workers' Rights, Maputo, Mozambique, ^3^Pathfinder International, Maputo, Mozambique, ^4^Aidsfonds‐Soa Aids Nederlands, Amsterdam, the Netherlands

**Background: **Since 2014, the Hands Off programme works to reduce violence against sex workers in Southern Africa, to support a decrease in HIV acquisition among this key population. When the programme began, little to no data regarding the prevalence of human rights violations against sex workers was available in the region, meaning previous interventions and advocacy efforts did not focus on the problems that the community itself prioritized.

**Description: **Sex worker‐led organizations in Botswana, Mozambique, South Africa and Zimbabwe were trained on the use of Ona, a secure, free and open‐source data collection tool to capture human rights violations in real time. Organizations set up varied peer‐monitoring systems by training additional sex workers in their communities to respond to human rights violations and capture data regarding demographics of the sex worker, the kind of violence that occurred, who perpetrated the violence and so on. The data are securely stored via Ona, owned by the sex worker‐led organizations, and available for the organizations and the Hands Off team to access and use. Since 2021, these data have formed the basis of an annual human rights violations report published by Hands Off partners.

**Lessons learned: **Since 2021, at least 1500 human rights violations against sex workers have been recorded each year. When data collection is driven and owned by community organizations, we find these organizations take initiative to use the data in effective and innovative ways. In Mozambique, sex workers use the evidence collected in data‐driven advocacy for stronger protections for sex workers on community, regional and national levels. They also use the data collection system as an accountability mechanism to follow up on cases of violence reported to law enforcement. Sex workers in Mozambique report stronger relationships with police and greater safety in their work as a result.

**Conclusions/Next steps: **Community‐driven data collection for sex workers is vital for real‐time understanding the nature and prevalence of violence in their communities. Their ownership over both the collection process and data itself has created a stronger understanding of high‐risk hot spots and stakeholders to target for sensitization, and builds a strong evidence base for data‐driven advocacy to support violence prevention.

### OAE2502 Country HIV response sustainability roadmaps: where are key populations in the conversations?

R. Ochanda ^1^, J. MacWilliam^2^, I. Tendolkar^2^, A. Gaudino^2^, K. Chapola^3^, M. Amalumilo^4^, S. Wambua^5^, A. Mitha^6^, F. Tingiba^7^, S. Malunga^8^, K. Mutale^8^, M. Mutongore^9^, C. Aviah^9^, O. Mussa^10^, A. Enema^11^

^1^AVAC, Policy Advocacy, Nairobi, Kenya, ^2^AVAC, Policy Advocacy, New York, United States, ^3^CHERA, Advocacy, Lilongwe, Malawi, ^4^Nigeria Key Population Health and Rights Network, MSM and Transgender Network, Enugu, Nigeria, ^5^Kenya Key Population Consortium, Leadership, Nairobi, Kenya, ^6^Malawi Diversity Forum, Leadership, Lilongwe, Malawi, ^7^South Sudan Key and Vulnerable Populations Forum, Leadership, Juba, South Sudan, ^8^Key Population Trans National Collaboration/Zambia Key Population Consortium, Secretariat, Lusaka, Zambia, ^9^Tanzania Key and Vulnerable Populations Forum, Leadership, Dar es Salaam, the United Republic of Tanzania, ^10^Zanzibar Key and Vulnerable Populations Forum, Leadership, Pemba, the United Republic of Tanzania, ^11^Nigeria Key Population Health and Rights Network, KP Secretariat, Lagos, Nigeria

**Background: **The 2022 PEPFAR Strategic Plan encourages recipient governments to develop sustainability roadmaps. PEPFAR strategy further stipulates that a sustainable response must place people and communities at its centre.

**Description**: A rapid, fact‐finding study conducted by the Key Population Trans‐National Collaboration (KP‐TNC) in six countries (Kenya, Tanzania, Zambia, Nigeria, Malawi and South Sudan) reveals a notable absence of the KP community in the sustainability discussions. The KP community is not involved in the sustainability discussions yet some of these countries have created Multi‐stakeholder Sustainability Technical Working Groups, KP Technical Working Groups and representative apex KP bodies (commonly known as KP‐Consortia) that connect KPs to different HIV response platforms and stakeholders.

**Lessons learned: **According to the fact‐finding study, key reasons for not including the KP community in the sustainability discussions include: cultural and religious beliefs that stigmatize KPs; push from some conservative policymakers for non‐inclusive integration of KP programmes; existence of punitive laws towards KPs; lack of frameworks providing guidelines on how the KP community will be included in the sustainability framework; and lack of political will stemming from lack of understanding of KP needs in relation to HIV programming. This is a signal that some or all KP HIV response needs within countries are likely not to be considered in the final sustainability roadmaps in case the KP community will keep being left out of the ongoing conversations.

**Conclusions/Next steps: **The lack of involvement of KP networks and representatives in the sustainability roadmap discussions is quite concerning and signals that countries are likely not to support KP programmes once they assume the full burden of the HIV response. This being the case, it would mean that the current gains that have been made in HIV response among KPs and general populations are likely to be reversed. PEPFAR, Global Fund, UNAIDS and other stakeholders, therefore, need to assist countries to develop a framework for KP engagements in the sustainability roadmap discussions.

### OAE2503 Social contracting of NGOs/CBOs to reach the last mile key populations under National AIDS Control Programme in India: learnings from the implementation of targeted intervention projects

S. Purohit ^1^, S. Rajan^1^, S. Bhavsar^1^, G. Pandey^2^, S. Kumar^2^

^1^National AIDS Control Organisation, New Delhi, India, ^2^PATH, New Delhi, India

**Background: **National AIDS Control Organisation (NACO) in India has partnered with civil society organizations (CSOs) primarily non‐governmental organizations (NGOs) and community‐based organizations (CBOs) through State AIDS Control Societies (SACS) under the social contracting mechanism to expand the reach and provide prevention services to the key populations. The social contracting model not only includes grant‐making by the Government of India but also ensures legislative, policy and programmatic initiatives to strengthen the service delivery mechanisms.

**Description: **NACO implements the Targeted Intervention (TI) projects as a major prevention initiative through CSOs (NGOs/CBOs) to reach out to the key populations. Peer‐led outreach model is adopted to provide a basic package of services tailored to the demands of key populations. The services include counselling for behaviour change, screening for HIV/STI, distribution of commodities (condoms, needle/syringes, lubes, etc.), linkages to treatment, as well as mobilization to reach the last mile. The engagement of CSOs in the projects goes through a robust selection process followed by financing, recruitment of staff, monitoring, supportive supervision, capacity building and evaluation of projects through third parties in a participatory process. TI project coverage of the last 6 years (2017−2018 to 2022−2023) is analysed and presented below to see the impact.

**Table:** Table. OAE2503

Year	Number of TI projects	Total coverage of key population (in lakh)	Total HIV test conducted (in lakh)	% of Sero‐HIV‐positive case detected	% linked to ART
2017−2018	1450	60.67	21.74	0.25	81.5
2018−2019	1443	66.62	23.45	0.25	83.0
2019−2020	1426	86.61	27.41	0.23	86.7
2020−2021	1472	64.77	23.24	0.20	85.0
2021−2022	1512	90.56	30.62	0.20	80.4
2022−2023	1543	100.88	34.57	0.25	82.7

The above table indicates that the coverage of KP under TI has increased over the year with the increase in HIV testing and linkages to ART.

**Lessons learned: **Peer‐led outreach combined with participatory evaluation, digital reporting and capacity building of CSOs has resulted in an increase in the coverage of KP under the projects.

**Conclusions/Next steps**: India's social contracting model has contributed to expanding reach with services, increasing community ownership, improved governance, accountability and partnership between the Government and CSOs. The social contracting model can be replicated in other disease prevention and control programmes in the larger health system to fast‐track results and achievements.

### OAE2504 From outreach provider to primary healthcare clinic for key populations in Thailand: how a community‐based organization expanded its mission and moved to sustainable financing

P. Patpeerapong ^1^, S. Santayakul^2^, S. Mills^2^, P. Rattakittvijun Na Nakorn^3^

^1^Mplus Foundation, Chiang Mai, Thailand, ^2^Family Health International (FHI360), Bangkok, Thailand, ^3^USAID, Bangkok, Thailand

**Background: **MPLUS Foundation is a community‐based organization delivering comprehensive HIV, STI and primary healthcare services through a key‐population‐led health service (KPLHS) approach. Over two decades, MPLUS evolved from conducting only HIV outreach to now serving as a government‐certified clinic, extending its services to include HIV‐adjacent areas like mental health, stigma and discrimination, and anti‐bullying. This expansion is supported by diverse funding sources, including USAID, the Global Fund, domestic health financing, grants and social enterprise incubation.

**Description: **Based on clients’ needs, strategic planning and its evolving mission, MPLUS expanded its services over time to offer increasingly comprehensive HIV/STI services and primary healthcare. This now includes HIV testing, PrEP and referral to ART services, all recognized over time by the Thai government as reimbursable services by the Thailand National Health Security Office (NHSO). In 2019, MPLUS became the first KP‐led organization certified as an HIV testing facility and gained ART provider certification in 2023. This certification led to increased domestic financing, reducing MPLUS’ dependence on international donors. These sources supported 18% of total operational costs in 2019, increasing to 56% in 2023. Furthermore, a fee‐based social enterprise service was added in 2023, accounting for 2% of total revenue in 2023.

**Lessons learned: **Challenges remain to full sustainability. In January 2023, the Thai government restricted HIV prevention services for non‐universal coverage (UC) clients, challenging MPLUS's financial viability. As a certified health facility, MPLUS was more resilient than others in continuing core HIV services through a fee‐based model for certain HIV services, utilizing this revenue as a revolving fund to sustain essential HIV services for key populations whose health benefits are not registered under the UC scheme and those with economic challenges. MPLUS accessed the domestic health fund to explore government reimbursement for adjacent diseases like mental health and telemedicine for non‐communicable diseases to promote wellbeing of older key populations and other groups in need.

**Conclusions/Next steps: **With careful strategic planning and in environments with universal health coverage, KP‐led organizations can transform into primary care facilities to sustainably serve their targeted populations through a one‐stop shop model supported by diversified funding, including domestic health financing, grants and social enterprises.

### OAE2505 Key population community perspectives on sustainable HIV services

M. Ighodaro ^1^, A. Mehrotra^2^, R. MacInnis^3^, A. Lambert^2^, D. Wendt^2^, M. Schiff^3^, C. Akolo^2^

^1^Global Black Gay Men Connect Corporation, New York, United States, ^2^FHI 360, United States, ^3^The Palladium Group, United States

**Background: **Global funding for HIV programming has decreased, and more funders are prioritizing “transition” or “sustainability” strategies. Continued HIV services for key population (KP) communities under alternative forms of financing require thoughtful consideration prior to transition and inclusion of KP perspectives to ensure continued service utilization and funding.

**Description: **The EpiC Project and Global Black Gay Men Connect (GBGMC) conducted five virtual focus group discussions between August and October 2023. Participants represented a diverse range of perspectives including 84 members of KP communities and organizations serving KP communities from 27 countries in Africa, Asia and the Caribbean. Participants shared viewpoints in a pre‐consultation survey (*n* = 75), a 2‐hour discussion (*n* = 84) and a post‐consultation survey (*n* = 37). One consultation was held in French and four in English. Anonymized pre‐consultation survey results were presented during focus groups. Focus groups were transcribed, excluding identifying details, and notes analysed for prevalent themes.

**Lessons learned: **Consistently, participants highlighted the importance of meaningful involvement of KP communities, including engagement in design, delivery and evaluation of services paired with capacity strengthening support, to ensure communities are prepared to lead service delivery. Access to client‐centred HIV services tailored to KP communities (preferably at KP‐led facilities) free of stigma and discrimination was noted as critical. Adequate financing for services including access to innovative financing structures such as social contracting or social enterprise to meet needs not funded by governments or other donors was also a theme.

Regarding current services, participants said: “The involvement of key population groups in providing sustainable services is essential … In [my country], the proportion of clients coming to key population‐led facilities is 3–4 times higher than public facilities. And for the clients who come to public facilities … they usually go with a key population supporter.”

In the post‐consultation survey, participants requested in‐person consultations to have a more nuanced exploration of local financing options, and review models of effective engagement and capacity strengthening for KP communities.

**Conclusions/Next steps: **As sustainability‐related conversations with funders and governments move forward, further dialogue and engagement of key population communities is needed to ensure equitable access to services in the future and sustain current gains in service delivery.

### OAE3002 Mental health outcomes of transgender women in Brazil: a narrative review and a call to action for public health policies

D. R. Araujo Coelho ^1^, W. Fernando Vieira^2^, C. Coutinho^3^, S. L. Reisner^1,4,5^, V. G. Veloso^3^, B. G. Grinsztejn^3^

^1^Harvard T. H. Chan School of Public Health, Department of Epidemiology, Boston, United States, ^2^Universidade de São Paulo, Instituto de Ciências Biomédicas, São Paulo, Brazil, ^3^Instituto Nacional de Infectologia Evandro Chagas, Fundação Oswaldo Cruz (INI‐Fiocruz), Rio de Janeiro, Brazil, ^4^Fenway Health, The Fenway Institute, Boston, United States, ^5^University of Michigan, Department of Epidemiology, Ann Arbor, United States

**Background: **In Brazil, transgender women (TGW) face several mental health challenges, often exacerbated by societal discrimination, physical and sexual violence, economic struggles and HIV healthcare. This study reviews these mental health challenges and suggests public health interventions for this vulnerable population.

**Methods: **A narrative review on PubMed was conducted in December 2023, using a combination of Mesh terms and title/abstract keywords: “TGW,” “mental health outcomes,” “socio‐demographic factors” and “Brazil.” Observational studies on mental health outcomes of TGW in Brazil, regardless of HIV status, were included. Exclusions were made for studies not published in English or Portuguese, case reports, case series and abstracts.

**Results: **The review revealed high rates of anxiety (26.5%−70.1%), depression (19.1%−69.6%), substance abuse (21.5%−56.6%) and suicidality (25%−47.25%) among TGW in Brazil. These issues are intensified by several societal factors, as illustrated in Table 1. We propose an integrated policy framework, focusing on: (1) Expanding gender‐affirming care in Brazil's Unified Health System (SUS), training health professionals in transgender health and broadening access to mental health services; (2) Promoting the Dandarah app, a tool for reporting harassment and violence; (3) Enhancing job training and placement through non‐governmental organizations (NGOs) like Transempregos; (4) Implementing affirmative university policies for TGW; (5) Improving access to HIV testing, PrEP, PEP and highly active antiretroviral therapy (HAART). Key stakeholders include SUS, the Ministry of Health, healthcare professionals, tech developers, law enforcement, legal aid organizations, employment agencies, private sector, educational institutions, schools and NGOs.

**Conclusions: **TGW in Brazil experience significant mental health issues, influenced by various factors. A holistic approach, encompassing healthcare, safety, employment, education and HIV care, is essential. Effective public health policies must be inclusive, responsive and tailored to TGW's needs, promoting their wellbeing and dignity.

**Table 1:** OAE3002

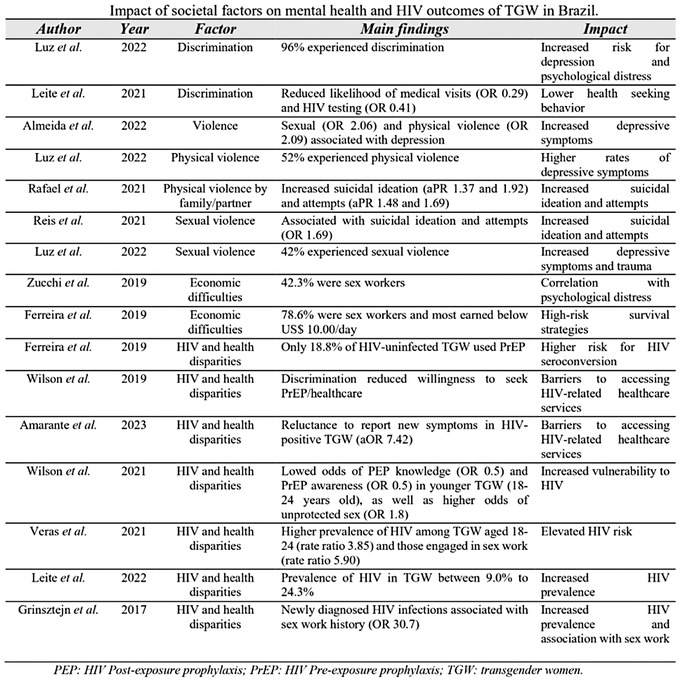

### OAE3003 Role of psychosocial counselling in harm reduction programme among people who inject drugs: Bangladesh experience

M. G. Rozario ^1^, S. Islam^2^, M. Akhtaruzzaman^3^, M.‐U. Rashid^1^, A. Chowdhury^2^, F. Afroz^4^

^1^CARE Bangladesh, Health and Nutrition Unit, Dhaka, Bangladesh, ^2^Save the Children in Bangladesh, HIV/AIDS Program Health and Nutrition Sector, Dhaka, Bangladesh, ^3^National AIDS/STD Control Program Bangladesh, DGHS, Dhaka, Bangladesh, ^4^CARE Bangladesh, Health and Nutrition Unit, Dhaka, Bangladesh

**Background: **Since 1995, Bangladesh has implemented the harm reduction programme to address the needs of the people who inject drugs (PWID). From 2021 to 2023, CARE Bangladesh as an implementer of the Global Fund grant is providing essential harm reduction services to 15,989 PWID of 13 districts. Bio‐medical and psychosocial interventions were integrated into the programme strategies to minimize the spread of HIV among PWID.

**Description: **Drug addiction's impact on the body, mind and nervous system, with associated mental health issues, underscores the importance of psychosocial support in successfully integrating drug users into harm reduction services. Twenty percent PWID in Bangladesh enrolled in harm reduction services have a history of over 10 years of drug injection, often accompanied by mental illness. The harm reduction package offered includes a range of services, from needle‐syringe distribution to condom promotion and treatment for various health issues, addressing the complex needs of this population.

Twenty five percent PWID have received case management, brief intervention, motivational interviewing, cognitive behaviour therapy and social support group development which have decrease drop‐out from 9.7% (2021) to 2.2% (2023) and adherence increased from 90% (2021) to more than 95% (2023) to antiretroviral therapy (ART), opioid substitution therapy, management of HIV‐HCV.

**Lessons learned: **Mental health support emerges as a cornerstone in addressing drug use disorders, teaching individuals coping mechanisms, replacing negative thoughts with positive ones, emphasizing treatment benefits, and involving family and social support systems. Despite the challenges faced by counsellors in conducting sessions with PWID, varied methods, including case management, have been employed to ensure effective counselling sessions through different psychosocial interventions.

**Conclusions/Next steps: **Programmatic data for 2022 showcase retention rates on methadone therapy increase from 63% (2021) to 89% (2023), increase adherence to ART more than 95% and management of HIV‐HCV among 681 PLHIV client. These positive outcomes underscore the critical impact of psychosocial interventions in maintaining the wellbeing and social integration of individual drug users.

### OAE3004 iTHRIVE 365 reduces the negative psychological impact of daily intersection stigma for black same gender loving men living with HIV

L. Scott‐Walker ^1^, J. Smith^1^, D. English^2^

^1^Rutgers University, Camden, United States, ^2^Rutgers University, New Brunswick, United States

**Background: **While emerging evidence shows health inequities among Black gay, bisexual, other same gender loving men living with HIV (SGLMLWH) are driven by intersectional stigma, there is a lack of interventions designed to combat stigma and promote their mental health. In light of this, our Black SGLM‐led multisectoral team developed iTHRIVE 365, a multicomponent mHealth intervention designed to combat the negative effects of stigma by increasing access to health information and motivation, cultivating Black SGLM social support, and facilitating connections to culturally affirming healthcare and economic resources. This study examined whether this intervention moderated the daily effects of intersectional stigma on depressive symptoms, anxiety symptoms and emotion dysregulation among a sample of Black SGLMliving with HIV.

**Methods: **We conducted a 14‐day diary study with 32 Black SGLMLWH in Atlanta, GA. Daily surveys assessed intersectional stigma, depressive symptoms, anxiety symptoms and emotion regulation difficulties. Participants spent 7 days without app access and 7 days with app access. Dynamic structural equation modelling assessed longitudinal associations from intersectional stigma to next‐day psychological health. We tested for intervention moderation by comparing these associations between non‐access and app‐access days.

**Results: **Intersectional stigma was positively associated with next‐day depressive symptoms (𝛾 = 0.21, 95% CI = [.10, .34]), anxiety symptoms (𝛾 = 0.26, 95% CI = [.13, .37]) and emotion dysregulation (𝛾 = 0.09, 95% CI = [.01, .19]). iTHRIVE365 significantly moderated the associations with anxiety symptoms and emotion dysregulation, such that there were significant associations with stigma on non‐access days, but not app‐access days.

**Conclusions: **Findings suggest that iTHRIVE365 may be an effective tool in reducing the negative mental health impacts of daily intersectional stigma for Black SGLMLWH.

**Table:** Table. OAE3004: Grouped dynamic structural equation results

Group	Outcome	Parameter	Estimate	95% CI	Std. Effect
No T365 Access	Depressive Sx	Stigma, Fixed Effect	0.18*	[.02, .33]	0.23
	Anxiety Sx	Stigma, Fixed Effect	0.32*	[.16, .50]	0.37
	Emotion Dys	Stigma, Fixed Effect	0.15*	[.02, .28]	0.26
T365 Access	Depressive Sx	Stigma, Fixed Effect	0.19*	[.03, .41]	0.22
	Anxiety Sx	Stigma, Fixed Effect	0.19	[−.01, .40]	0.20
	Emotion Dys	Stigma, Fixed Effect	0.02	[−.10, .15]	0.03

Abbreviations: CI, credible interval using the highest posterior density method; Dys, dysregulation; Estimate, median of the posterior distribution; Std. Effect, standardization using the average of within‐person standardization method; Sx, symptoms; T365, iTHRIVE365 intervention.

*indicates that 0 is not in the 95% credible interval of the parameter and is analogous to being statistically significant in a frequentist inference.

### OAE3005 Evaluating the impact of Prime Time Sister Circles on select health outcomes for a diverse cohort of Black women with HIV in urban United States

H. Kwakwa ^1^, A. Dongala^1^, M. Dorsainvil^1^, T. Stokes^1^

^1^Philadelphia Department of Public Health, Ambulatory Health Services, Philadelphia, United States

**Background: **Prime time sister circles (PTSC) is a community‐based, culturally relevant intervention designed to help Black women improve cardiovascular health. With US Black women disproportionately impacted by cardiovascular risk, interventions to improve cardiovascular health in Black women with HIV are urgently needed. In one of 12 projects funded by health resources and services administration (HRSA), we implemented PTSC with two other interventions (Red Carpet Care and Trauma‐informed Care) bundled for Black women with HIV. We report the clinical and access to care impact of PTSC plus the other two (base) interventions, compared with the base interventions only.

**Methods: **This study was conducted in community health centres in Philadelphia between May 2021 and December 2022. Eligibility criteria were: (1) Self‐identification as a Black woman and (2) HIV diagnosis with (3a) Re‐engagement in care or (3b) New diagnosis or (3c) ≥2 Emergency Department (ED) visits or ≥1 hospitalization within 12 months or (3d) ≥2 missed HIV care visits within 6 months. The base interventions were conducted over a 6‐month period, and the PTSC intervention occurred over 12 weeks. A comprehensive survey and laboratory examination were conducted and access‐to‐care data collected at baseline, 6 months and 12 months. We present a descriptive analysis of data collected, comparing the PTSC group to the base intervention only cohort.

**Results: **Forty‐six women enrolled the project, 19 of them opting into and 14 completing the PTSC intervention. Mean age was 51, 19 were born outside the United States, 2 were transgender and participants reported a mean of 6 chronic co‐morbidities. For both groups, improvements were noted in HIV RNA, CD4 cell counts, haemoglobin A1c and LDL as well as in ED utilization and missed HIV primary care visits. These improvements were more pronounced at 6 months than 12 months. Additionally, PTSC participants showed an improvement in blood pressure at 6 months only.

**Conclusions: **Our base evidence‐informed bundled interventions were collectively successful in improving HIV‐related parameters as well as select cardiovascular risk parameters in this diverse cohort of ageing Black women with HIV. The inclusion of PTSC provided added value in improving blood pressure at 6 months. The slight worsening from 6 to 12 months mandates further study of maintenance interventions.

### OAE3902 STI testing rates among PrEP users randomized to 3‐monthly (standard of care) or 6‐monthly monitoring within the EZI‐PrEP trial, the Netherlands: preliminary results

M. L. Groot Bruinderink ^1,2^, E. S. Wijstma^1^, V. W. Jongen^1,3^, L. Blitz^4^, C. van Bokhoven‐Rombouts^5^, J. Woudstra^1^, K. Vermeij^6^, S. Boers^7^, H. M. Gotz^7,8^, F. van Harreveld^2,9^, M. Prins^2,10,11,12^, E. Hoornenborg^1,10,11,12^, U. Davidovich^1,2^, M. Schim van der Loeff^1,10,11,12^

^1^Public Health Service Amsterdam, Department of Infectious Diseases, Amsterdam, the Netherlands, ^2^University of Amsterdam, Department of Psychology, AmsterdamNet, the Netherlands, ^3^HIV Monitoring Foundation, Amsterdam, the Netherlands, ^4^Public Health Service of Haaglanden, Department of Sexual Health, The Hague, the Netherlands, ^5^Public Health Service of Gelderland‐Zuid, Nijmegen, the Netherlands, ^6^Aidsfonds—Soa Aids Nederland, Amsterdam, the Netherlands, ^7^Public Health Service of Rotterdam‐Rijnmond, Department of Infectious Diseases, Rotterdam, the Netherlands, ^8^Erasmus MC, University Medical Center, Department of Public Health, Rotterdam, the Netherlands, ^9^National Institute for Public Health and the Environment (RIVM), Bilthoven, the Netherlands, ^10^Amsterdam UMC, University of Amsterdam, Department of Internal Medicine, Amsterdam, the Netherlands, ^11^Amsterdam Institute for Immunology and Infectious Diseases (AII), AmsterdamNether, the Netherlands, ^12^Amsterdam Public Health Research Institute (APH), Amsterdam, the Netherlands

**Background: **Decreasing PrEP monitoring visits can reduce PrEP use burden. We examined STI testing behaviour and STI positivity among PrEP users randomized to a less frequent 6‐ versus 3‐monthly STI screening (standard‐of‐care) within the EZI‐PrEP study.

**Methods: **EZI‐PrEP is a randomized controlled trial (September 2021−March 2024) on the non‐inferiority of 6‐ versus 3‐monthly, and online versus in‐clinic PrEP monitoring among men‐who‐have‐sex‐with‐men in the Netherlands. Monitoring includes STI screening; additional free‐of‐charge STI testing in‐between monitoring visits is optional. This preliminary analysis includes data from participants with >1 PrEP follow‐up monitoring visit in Amsterdam, Rotterdam, The Hague and Nijmegen until September 2023. Using visit rate ratios and 95% CI, we compared (i) overall visit rates (i.e. number of PrEP visits and additional STI visits per person‐year) and (ii) additional STI visit rates (i.e. number of additional STI visits per person‐year) between 6‐ and 3‐monthly monitoring arms. Using χ^2^ test, we compared (iii) the proportion of all visits with any positive chlamydia, gonorrhoea or infectious syphilis (“any STI”) and (iv) the proportion of all additional STI visits without STI‐related symptoms or partner notification between 6‐ and 3‐monthly monitoring arms.

**Results: **Four hundred and twenty‐eight participants (6‐monthly arm: *n* = 213; 3‐monthly arm: *n* = 215) contributed to 512 person‐years of follow‐up. The overall visit rate in the 6‐monthly arm was lower than the 3‐monthly arm (visit rate ratio = 0.70, 95% CI: 0.64−0.77). The additional STI visit rate in the 6‐monthly arm was higher than the 3‐monthly arm (visit rate ratio = 1.94, 95% CI: 1.58−2.40). We found no difference in STI positivity between arms (6‐monthly arm: 22.3%; 3‐monthly arm: 20.5%, *p* = 0.35), nor in the proportion of additional STI visits without STI‐related symptoms or a partner notification (6‐monthly arm: 52.1%; 3‐monthly arm: 42.8%, *p* = 0.071).

**Conclusions: **Compared to PrEP users monitored every 3 months, PrEP users monitored every 6 months attended more additional STI visits, but had fewer visits overall. STI positivity was comparable between the arms. These preliminary findings suggest that implementing 6‐monthly PrEP monitoring as standard‐of‐care could reduce the total number of visits without resulting in increased STI positivity, leading to cost reductions of PrEP programmes. Further research on the impact of 6‐monthly monitoring on STI transmission is ongoing.

### OAE3903 Long‐term health outcomes of people living with HIV who were enrolled in 6‐month dispensing of antiretroviral treatment for 12−18 months in four provinces of Mozambique

J. Chacha ^1^, O. Canhanga^1^, J. Moiane^2^, M. Prieto^2^

^1^Efficiencies for Clinical HIV Outcomes (ECHO) project, Mozambique/Abt Associates, Maputo, Mozambique, ^2^Efficiencies for Clinical HIV Outcomes (ECHO) project, Mozambique, Maputo, Mozambique

**Background: **Multi‐month dispensing (MMD) of antiretroviral treatment (ART) reduces unnecessary clinic visits, contributes to improve health outcomes among people living with HIV (PLHIV) and improves the efficiency of health systems. 3MMD dispensing has become the standard of care in many clinical and community settings, and now, globally, the focus is to extend MMD up to a period of 6‐months (6MMD). In 2020, Mozambique Ministry of Health adopted a phased approach to facilitate this transition towards 6MMD. USAID‐funded Efficiencies for Clinical HIV Outcomes (ECHO) project is supporting 6MMD implementation in 24 health facilities in four provinces. We analysed long‐term health outcomes of PLHIV who were enrolled in 6MMD for a period of 12−18 months.

**Methods: **This is a quantitative cross‐sectional study of PLHIV enrolled in 6MMD from April to September 2022. Eligibility criteria included: age ≥5 years old, on ART for ≥12 months, with a viral load (VL) <1000 copies/ml, have no current illness and not be on cotrimoxazole and/or tuberculosis prophylaxis (excluding pregnant/breastfeeding women). Data were collected from electronic medical records from 24 health facilities in four Mozambican provinces implementing 6MMD. All individuals were followed through October 2023 to assess VL coverage, VL suppression (defined as VL<1000 copies/ml) and retention in care after ≥12 months of enrolment in 6MMD. Descriptive statistics were used to characterize the study sample and determine long‐term health outcomes.

**Results: **A total of 55,820 PLHIV enrolled in 6MMD were included in the analysis, of whom 65% were female (36,303/55,820). The median age was 39 years (interquartile range [IQR]: 32–47 years). VL coverage was 82% (45,644/55,820) across the cohort, and among those with a VL test result, 98% (44,580/45,644) were virally suppressed. Ninety eight percent (54,954/55,820) of individuals were retained in HIV care after ≥12 months of enrolment in 6MMD.

**Conclusions: **PLHIV enrolled in 6MMD can achieve high rates of VL suppression and retention in care, which supports the case for the expansion of the model to other health facilities. Further research will be needed to assess outcomes for individuals who have a shorter period on ART before enrolment, and for individuals with longer‐term follow‐up.

### OAE3904 Aligning key population HIV prevention service preferences and coverage in Vietnam: findings from a national private sector engagement assessment

K. Green ^1^, H. Phan Thi Thu^2^, L. Tran Khanh^1^, A. Duong Thuy^2^, B. Vu Ngoc^1^, T. Tram Tri^1^, G. Nguyen Hoang^3^, D. Nguyen Anh^4^, T. Ngo Minh^4^, A. Doan Hong^4^, Y. Vu Ngoc^1^, G. Castillo^1^

^1^PATH, Hanoi, Viet Nam, ^2^VAAC/MOH, Hanoi, Viet Nam, ^3^HSPI, Hanoi, Viet Nam, ^4^USAID, Hanoi, Viet Nam

**Background: **The HIV response in Vietnam has seen major successes in PrEP scale‐up through a range of differentiated models. These include private sector services (PSSs) which are responsible for nearly half of PrEP enrolment nation‐wide. While KP access to preferred PSSs is critical to sustainably achieving ending AIDS by 2030 goals, the delta between KP service preferences, willingness to pay (WTP) and actual coverage of desired services is unknown. Such information is critical to understand as donor funding for HIV prevention steadily declines.

**Methods: **We conducted a mixed‐method assessment involving an HIV private‐sector engagement (PSE) readiness and coverage benchmarking (58/63 provinces) and a services use, preferences and WTP study in 11 highest HIV burden provinces among 3060 KP (1800 men who have sex with men, 420 transgender women; 420 people who inject drugs; 420 female sex workers) from May to October 2023. PSSs were defined as general or KP‐led clinics, hospitals or pharmacies, and the coverage assessment included provincial mapping of identified services, inventory of services offered and maximum client load. We also scored provincial Department of Health readiness to engage with or increase coverage of PSSs.

**Results: **Only five of 58 provinces were identified as having high to very high HIV/PHC‐related PSS coverage and 36% of provinces had no PSSs at all. Overall, two provinces were identified as representing combined strong PSE readiness and high coverage of PSS. Related to service preferences, 70.5% and 72.2% of KP preferred KP‐led clinics for PrEP and PEP, respectively, followed by the public sector and then general private clinics or hospitals. 79.2% of KP reported WTP for PrEP and 68.2% for PEP. Of those, 51% of MSM reported being able to pay the average commercial price for PrEP (drugs, tests, exam), while only 6.9% of PWID, FSW and TGW were able to do the same.

**Conclusions: **There is a significant misalignment between KP service preferences, access to PSSs and WTP for preferred services. As donor funding declines, partial‐subsidy models that offer reductions in HIV prevention pricing will be required as parallel efforts are undertaken to reduce cost and ultimate price passed onto clients for commercial services.

### OAE3905 Client experiences of and preferences for HIV care delivery during the first 6 months on antiretroviral therapy in South Africa

N. Mutanda ^1^, A. J. Morgan^2^, L. Sande^1^, M. Maskew^1^, A. Kamanga^3^, V. Ntjikelane^1^, N. Scott^2^, M. Benade^2^, S. Rosen^1,2^

^1^University of Witwatersrand, Health Economics and Epidemiology Research Office, Johannesburg, South Africa, ^2^Boston University, School of Public Health, Massachusetts, United States, ^3^Clinton Health Access Initiative, Lusaka, Zambia

**Background: **Improving retention on antiretroviral therapy (ART) is essential to achieve global HIV goals. Disengagement from care is highest in the early treatment period (<6 months after initiation or re‐initiation), but differentiated service delivery models designed to increase retention generally exclude clients with <6 months on ART. We assessed preferences for HIV service delivery in the early treatment period.

**Methods: **From 9/2022 to 6/2023, we surveyed adult (≥18) clients who were initiating, re‐initiating or on ART for ≤6 months at 18 primary health facilities in South Africa. The survey collected data on experiences with and preferences for HIV treatment delivery. A subset of respondents were re‐contacted ≤12 months later for focus group discussions (FGDs) to further explore preferences.

**Results: **We enrolled 1098 adults (72% female, median age 33): 38% were initiating/re‐initiating ART at study enrolment, 38% had been on ART ≤3 months and 24% had been on ART 3−6 months. While 81% of clients reported actually receiving 1 month of medication at a time, 63% said they would prefer 3‐ or 6‐month dispensing. Sixty one percent preferred 3‐ or 6‐month visit scheduling, while only 13% preferred monthly visits. Seventy nine percent overall would prefer to receive care from a nurse over a doctor (15%), counsellor (5%) or other provider (2%); men (20%) were more likely than women (12%) to prefer a doctor (*p*<0.05). Sixty five percent said they would prefer receiving treatment in community settings (school, church or pharmacy) instead of a clinic. A large majority of participants (93%) had not been offered any choices of service delivery locations or dispensing durations. Fifty percent of participants desired more counselling; FGD participants expressed the need for frequent, intensive and empathetic counselling during the early treatment period. Unlike the quantitative survey results, FGD participants expressed a preference for collecting medication from the clinic rather than at community pickup points to provide an opportunity to ask questions and receive counselling.

**Conclusions: **Even during the first 6 months after ART initiation, a substantial proportion of clients would prefer less frequent clinic visits and longer dispensing intervals, though they also value frequent and high‐quality counselling. New care models for the early treatment period should reflect these preferences.

### OAF0302 Challenging assumptions: rethinking the efficacy of voluntary licensing in medicine access, economic landscapes and policy frameworks

O. Mellouk ^1^, G. Krikorian^2^, S. Kondratuyk^1^

^1^ITPC, Johannesburg, South Africa, ^2^Independent Consultant, Paris, France

**Background: **Voluntary licensing is a prevalent practice, particularly in sectors where patents are numerous like pharmaceuticals. During recent years, voluntary licences (VLs) emerged as a predominant solution to ensure access to treatment in resource‐limited settings to the detriment of other compulsory strategies. The research delves into the multifaceted impacts of VLs on access to medicines, the economic dynamics of the pharmaceutical market and the broader discourse surrounding access to health.

**Description: **Drawing insights from an extensive review of bibliographic resources, licence agreements, and consultations with legal experts and stakeholders involved in HIV treatment and other medicine policies, this study conducted two analyses. Firstly, an assessment of the provisions within licence agreements was undertaken. Secondly, the research documented the diverse effects and impacts that these agreements can exert on policies and stakeholders.

**Lessons learned: **The study uncovered a spectrum of potential effects stemming from voluntary licences. It explores both positive impacts, such as enhanced access for specific populations in certain countries, and negative features within agreements related to target countries, transparency, knowledge transfer, active ingredient usage, pricing, grant‐back of rights and more. The research examined how licence deals influence the conduct and business practices of pharmaceutical companies, including partnerships between previously competitive entities, risk assessment for engaging in compulsory licensing and potential repercussions for small generic manufacturers. Moreover, it scrutinized their impact on the access to medicines movement, the utilization of TRIPS flexibilities, and their broader influence on access politics and policy debates. The study concludes with a series of recommendations aimed at improving licence agreements, enhancing their monitoring and regulation, and mitigating the inadvertent creation of new barriers to access to medicines.

**Conclusions/Next steps: **The findings contribute to a comprehensive understanding of industrial dynamics and relationships among various pharmaceutical entities on global markets. They highlight the indirect role that voluntary licensing plays in shaping policies and the access to medicines landscape. These insights are crucial for formulating informed policies to improve access to HIV medicines and guiding stakeholders in negotiations aligned with this overarching objective.

### OAF0303 Popularize The Use Of TRIPS flexibilities in MENA countries to improve access to ARVs

O. Marrakchi ^1^

^1^International Treatment Preparedness Coalition for Middle East and North Africa, Marraekech, Morocco

**Background: **In the MENA region, the majority of countries encounter challenges in accessing high‐quality antiretroviral drugs (ARVs). The latest generation treatments are often patented, and many countries in the region, classified as “developing countries,” are increasingly excluded from voluntary licences granted by pharmaceutical laboratories. In response to this issue, a solution exists—using the TRIPS flexibilities provided by the WTO. However, these flexibilities are relatively unknown in the region, despite their potential to save millions of lives.

**Description: **The UNITAID Project, led by ITPC Global, is implemented across several countries worldwide. ITPC MENA, in its role as SSR, has been responsible for implementing the project in Morocco and the MENA region since 2018. The project aims to promote the use of flexibilities to enhance access to ARVs as well as treatments for co‐infections, such as TB, hepatitis and even Covid‐19.

**Lessons learned: **For over 6 years, ITPC MENA has undertaken extensive evidence collection efforts, conducting numerous studies to substantiate our advocacy and expose certain barriers hindering the use of these flexibilities. These barriers include international agreements and validation agreements signed by some countries in the region with the European Patent Office (EPO), demonstrating their impact on access to medicines. Subsequently, based on this evidence, extensive awareness campaigns were conducted targeting civil society, key populations, decision‐makers, local generic manufacturers and patent offices. Finally, more tangible actions were taken, such as patent oppositions (e.g. TAF for HIV, Baricitinib for Covid‐19). Following this approach (data collection, raising‐awareness and concrete actions), we successfully conducted a robust advocacy campaign grounded in evidence, convincing the majority of stakeholders who did not hesitate to express their support.

**Conclusions/Next steps: **Today, we can affirm that the programme has borne fruit. For the first time, TRIPS flexibilities are being utilized in Morocco, thanks to successful advocacy and awareness efforts targeting decision‐makers. The next step is to replicate the same journey for Morocco in other countries within the region, starting with Tunisia as early as this year.

### OAF0304 National Health insurance coverage of HIV benefits in five PEPFAR‐supported countries: a scoping review

A. Verani ^1^, M. Ruffner^1^, F. Riako Anam^2^, N. Tavanxhi^3^, I. Semini^4^

^1^State Department, Bureau of Global Health Security and Diplomacy, U.S. President's Emergency Plan for AIDS Relief (PEPFAR), Washington, DC, United States, ^2^Global Network of People Living with HIV (GNP+), Amsterdam, the Netherlands, ^3^Global Fund to Fight AIDS, Tuberculosis, and Malaria, Geneva, Switzerland, ^4^UNAIDS, Geneva, Switzerland

**Background: **Integration of health benefits such as HIV testing and anti‐retroviral therapy (ART) into National Health Insurance (NHI), previously accomplished by Thailand and Vietnam with support from the U.S. President's Emergency Plan for AIDS Relief (PEPFAR), may help sustain the HIV response and improve the health of people living with HIV (PLHIV). African nations have also established NHI benefits.

**Methods: **To discuss key findings from this body of literature and identify HIV‐related benefits in NHI, we purposively selected four PEPFAR partner countries with NHI (Ghana, Kenya, Nigeria and Rwanda) and one PEPFAR partner country planning to initiate NHI (South Africa), then searched peer‐reviewed literature in PubMed using terms “health insurance AND HIV AND country name” for each country selected and included 31 of 292 articles that: (a) addressed publicly financed health insurance and HIV; (b) in the selected countries; and (c) published between 1 January 2019 and 31 December 2023. We identified which HIV benefits (testing, ART) and other benefits addressing PLHIV co‐morbidities were included in NHI benefits, through review of the peer‐reviewed literature and official NHI websites.

**Results: **HIV and non‐HIV benefits relevant to PLHIV are included in NHI packages of Ghana, Kenya, and Nigeria and planned for inclusion in South Africa's. Whether Rwanda's package covers these benefits is unclear, as benefits are stated generally (“drugs and medical services”). Our scoping review found: (1) *Having NHI is associated with*: higher rates of health‐seeking behaviour, HIV testing and antenatal screening; increased treatment of OIs in PLHIV; improved obstetric health among PLHIV; chemotherapy for PLHIV with Kaposi's sarcoma; higher cervical cancer survival rates; comprehensive knowledge about HIV/AIDS; and awareness of PrEP, and (2) *Not having NHI is associated with*: HIV acquisition; catastrophic health expenditures for PLHIV; cardiovascular risk factors among PLHIV; and being on ART.

**Conclusions: **Most countries reviewed include (and South Africa plans to include) multiple HIV‐related benefits in their NHI. Having NHI is associated with greater awareness, access to and use of healthcare by PLHIV (except ART). Integration of HIV services and other related health benefits into NHI is ongoing.

### OAF0305 Community advocacy to reduce IP barriers to access to affordable medicine: lessons learned from the Bedaquiline Patent Opposition in Indonesia

B. Larasati ^1^

^1^Indonesia AIDS Coalition, DKI Jakarta, Indonesia

**Background: **Trade Related Aspects on Intellectual Property Rights (TRIPS) poses a threat to public access to health commodities, especially in developing countries. TRIPS grants inventors monopoly rights, which results in high prices. However, there are cases when companies attempt to extend the protection period by registering new patents for minor modifications to existing products. To prevent such frivolous patents, the filing of Patent Opposition (PO) is needed.

**Description: **From November 2022 to November 2023, the Indonesia AIDS Coalition (IAC) filed a PO against Bedaquiline, part of the first‐line treatment regimen for Drug‐Resistant TB (DR‐TB), dispersible tablet formulations. Overall, Indonesia ranked second in the world for TB cases, with a total of 969,000 cases, of which an estimated 28,000 are DR‐TB. PLHIVs are especially vulnerable. In 2021, TB caused the deaths of 6500 PLHIV, or 29.5% of the total TB‐HIV cases. The PO process lasted for 10 rounds, from presenting written arguments to witness examinations. The arguments put forward are that CSOs are interested parties under the Patent Law and can file POs; the steps for creating dispersible tablets are already known; and patent applications for new uses of known substances without an increase in efficacy are not eligible for patents.

**Lessons learned: **(1) There are still differences in views between the judiciary panel and CSOs regarding CSOs’ legal standing, which have roots in ambiguous sentences in the Patent Law. This highlights the importance of strengthening the arguments by referring to the organization's constitution and track record, as well as the need to advocate for more clarity; (2) Difficulty in finding local pharmaceutical expert witnesses. This highlights the importance of building rapport with academic circles and the role of international networks; (3) Difficulty finding law firms that possess a community perspective; and (4) Difficulties in rallying support from TB groups due to the media ban during court proceedings.

**Conclusions/Next steps: **The Bedaquiline case marks the first time a CSO has conducted a PO in Indonesia. Not only for drugs but also for product patents. The lessons learned from this case will inform future PO efforts, specifically on strengthening the argument on CSOs’ legal standing.

### OAF1102 A scoping review of factors associated with HIV acquisition in the context of humanitarian crises

D. Harsono ^1^, S. Atre^2^, K. Nyhan^3,4^, D. Garmroudi^5^, J. L. Davis^1,6,7^, W. Ho^8^, K. Khoshnood^1,6^

^1^Center for Interdisciplinary Research on AIDS at Yale University, New Haven, United States, ^2^Yale School of Public Health, Department of Chronic Disease Epidemiology, New Haven, United States, ^3^Yale University, Harvey Cushing/John Hay Whitney Medical Library, New Haven, United States, ^4^Yale School of Public Health, Department of Environmental Health Sciences, New Haven, United States, ^5^Yale University, New Haven, United States, ^6^Yale School of Public Health, Department of Epidemiology of Microbial Diseases, New Haven, United States, ^7^Yale School of Medicine, Department of Pulmonary, Critical Care and Sleep Medicine, New Haven, United States, ^8^Yale School of Public Health, Department of Social and Behavioral Sciences, New Haven, United States

**Background: **Humanitarian crises—natural or human‐made events that can threaten communities’ health, safety, security and wellbeing—may affect the HIV epidemic dynamics and risk for HIV acquisition.

**Methods: **We conducted a scoping review of literature published in English between January 1990 and March 2022 to characterize the global evidence of modifiable and non‐modifiable factors for HIV acquisition in the context of humanitarian crises. We systematically searched, screened and synthesized literature from MEDLINE, Embase, Global Health (all accessed via Ovid) and Scopus, and sought out grey literature through websites of humanitarian agencies and relevant non‐government organizations, the International AIDS Society's abstract databases and Google Scholar. We considered studies presenting empirical data on HIV acquisition in humanitarian crises‐affected populations, including refugees, asylum seekers and internally displaced persons.

**Results: **Forty‐nine studies met the inclusion criteria. The majority of the studies were quantitative (*n* = 43, 87.8%) and cross‐sectional (*n* = 30, 61.2%) in nature. Most of the research was a single‐country study (*n* = 43, 87.8%) and conducted in sub‐Saharan Africa (*n* = 31, 63.3%). We identified five non‐modifiable factors associated with HIV acquisition (i.e. age, gender, location, place of birth or origin and ethnicity) and 60 modifiable factors that we classified into five categories, namely, 18 policy and structural, 9 socio‐cultural, 11 health and mental health, 16 sexual practices and 6 crisis‐related traumatic event factors. Within these modifiable categories, factors that were most often investigated were education level, marital status, sexually transmitted infection diagnosis, condom use and experience of rape or sexual trauma, respectively. Informed by the findings, we applied the social ecological framework to map the multidimensional factors associated with HIV acquisition in humanitarian settings at the individual‐, social and sexual networks‐, community‐, and public policy‐levels.

**Conclusions: **The current review provides a comprehensive, global analysis of evidence on factors associated with HIV acquisition in humanitarian settings and implications for future programmes and research. Future research should consider longitudinal and mixed methods designs to understand the potential causal linkages between non‐modifiable and modifiable factors and HIV acquisition and explore the lived experiences of crises‐affected populations. Such research will generate actionable evidence to inform ethical and culturally appropriate HIV prevention interventions in humanitarian settings.

### OAF1103 Assessing the disruption of HIV testing and treatment in Nepal during the COVID‐19 pandemic: an interrupted time series analysis

K. Deuba ^1,2^, S. Bhandari^3^, L. R. Pandey^4^, R. S. Kunwar^3^, M. B. Kc^4^

^1^Public Health and Environment Research Centre (PERC), Lalitpur, Nepal, ^2^Karolinska Institutet, Department of Global Public Health, Stockholm, Sweden, ^3^Save the Children, Kathmandu, Nepal, ^4^National Centre for AIDS & STD Control, Ministry of Health and Population, Kathmandu, Nepal

**Background: **Nepal's response to COVID‐19, involving public gathering bans and nationwide lockdowns, significantly impacted routine health services. This study evaluates the effect of these measures on HIV testing and treatment services.

**Methods: **We conducted an interrupted time series analysis using national routine health facility data from all HIV testing and treatment centres in Nepal, spanning January 2019−December 2021. The study period includes the pre‐pandemic phase (January 2019−March 2020) and the pandemic period marked by three COVID‐19 waves (December 2020−December 2021). We analysed monthly data on HIV testing, positive diagnoses and new antiretroviral therapy (ART) enrolments. The Poisson regression model was applied to compare rates pre‐ and post‐COVID‐19 outbreak, yielding relative risk (RR) estimates with 95% confidence intervals (CI).

**Results: **Throughout the study period, 675,939 individuals were tested for HIV, 7926 diagnosed with HIV and 7189 commenced ART. The pandemic correlated with a 34% reduction in HIV testing (RR: 0.657; 95% CI: 0.651−0.663; *p* < 0.001), a 47% decrease in positive diagnoses (RR: 0.529; 95% CI: 0.482−0.579; *p* < 0.001) and a 36% decline in new ART enrolments (RR: 0.641; 95% CI: 0.585−0.705; *p* < 0.001).

**Conclusions: **The COVID‐19 pandemic significantly disrupted HIV testing and treatment in Nepal. These findings highlight the urgent need for resilient health systems in low‐ and middle‐income countries to sustain HIV control efforts amidst pandemic‐related disruptions. Proactive measures are crucial to safeguard the gains made in HIV prevention and care against future health crises.

**Figure**. OAF1103

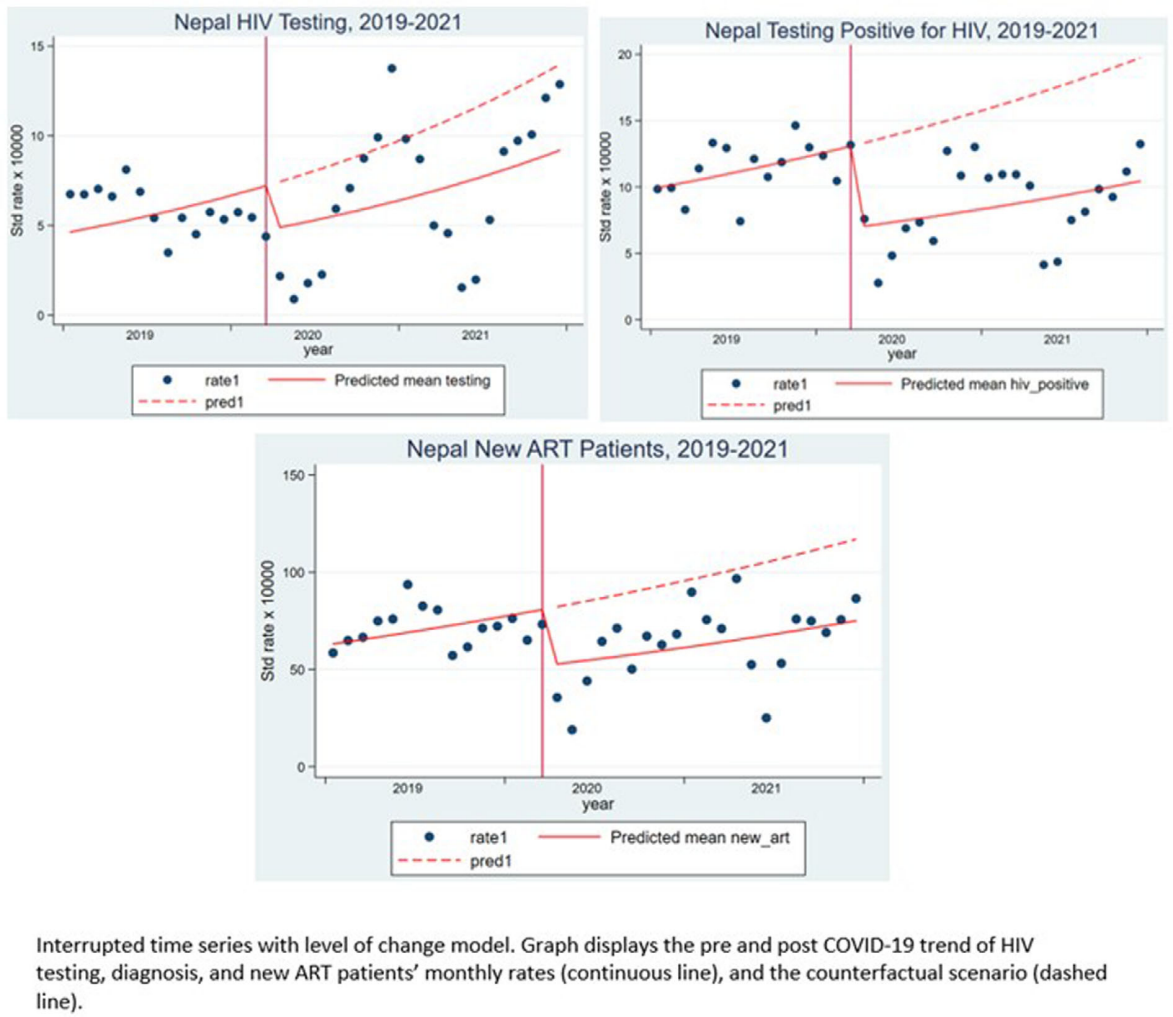

### OAF1104 Scaling up and drafting of risk mitigation strategies for key population programmes in the context of complex and changing legal, social and political environment in Kenya

R. N. Josphine ^1,2^, J. Kyana^3^, E. Amenya^4^

^1^Community Health Advocacy and Learning Initiative, Public Health Consultant, Kilifi, Kenya, ^2^USAID Stawisha Pwani, LVCT Health, Prevention and Key Population Program, Kilifi, Kenya, ^3^HAPA KENYA, Programs, Mombasa, Kenya, ^4^WACHA Health, Programmes, Mombasa, Kenya

**Background**: In a concerning trend of violence against Kenya's LGBTQ community, activists Joash Mosoti, Sheila Lumumba and Edwin Chiloba were brutally murdered between May 2021 and January 2023. This surge in hatred coincided with a growing regional anti‐right movement in East Africa fuelling an anti‐LGBTIQ discourse. The situation reached a tipping point when the Supreme court of Kenya delivered a landmark verdict on 24th March 2023 supporting legal registration of an LGBTIQ group. This further convoked protests, violence incitement towards LGBTIQ individuals, organizations and allies and introduction of anti‐LGBTIQ legislations in parliament.

**Description**: In March 2023, LGBTQ and sex workers’ leaders in Kenya's coast region addressed challenges amid the anti‐LGBTIQ campaign. Topics included rhetoric, legal gaps, evictions, violence and mental health. Proposed solutions: engaging sensitized religious leaders, mapping legal advisors, safe spaces, community sensitization and a crisis response framework. Strategies for mental health, evictions and asylum information were outlined. Funding from UHAI, Hivos, The Global Fund, USAID‐Stawisha Pwani, embassies and NGLHRC, KELIN and Defenders Coalition aims to protect LGBTQ rights amid a hostile environment.

**Lessons learned: **Despite security risks, essential services like ARV and PrEP distribution persisted amid anti‐LGBTIQ campaigns, utilizing COVID‐19 care models like tele‐consultation and online support. Community‐led programmes monitored campaigns online/offline. Limited donor support hindered organizations without a regional plan. Engagements with police, boda boda riders, religious leaders and health officials provided sensitization opportunities. Challenges included funding shortages for emergencies and structural interventions. Nevertheless, improved relationships with law enforcement, boda boda riders and health committees emerged through collaborative efforts amid adversity.

**Conclusions/Next steps: **Community leaders highlight inadequate understanding of key populations, escalating violence and protests against supporting key population programmes. To ensure HIV response sustainability, crucial factors include supporting and funding community‐led organizations for engaging stakeholders, challenging anti‐rights narratives and advocating for inclusion.

**Figure**. OAF1104

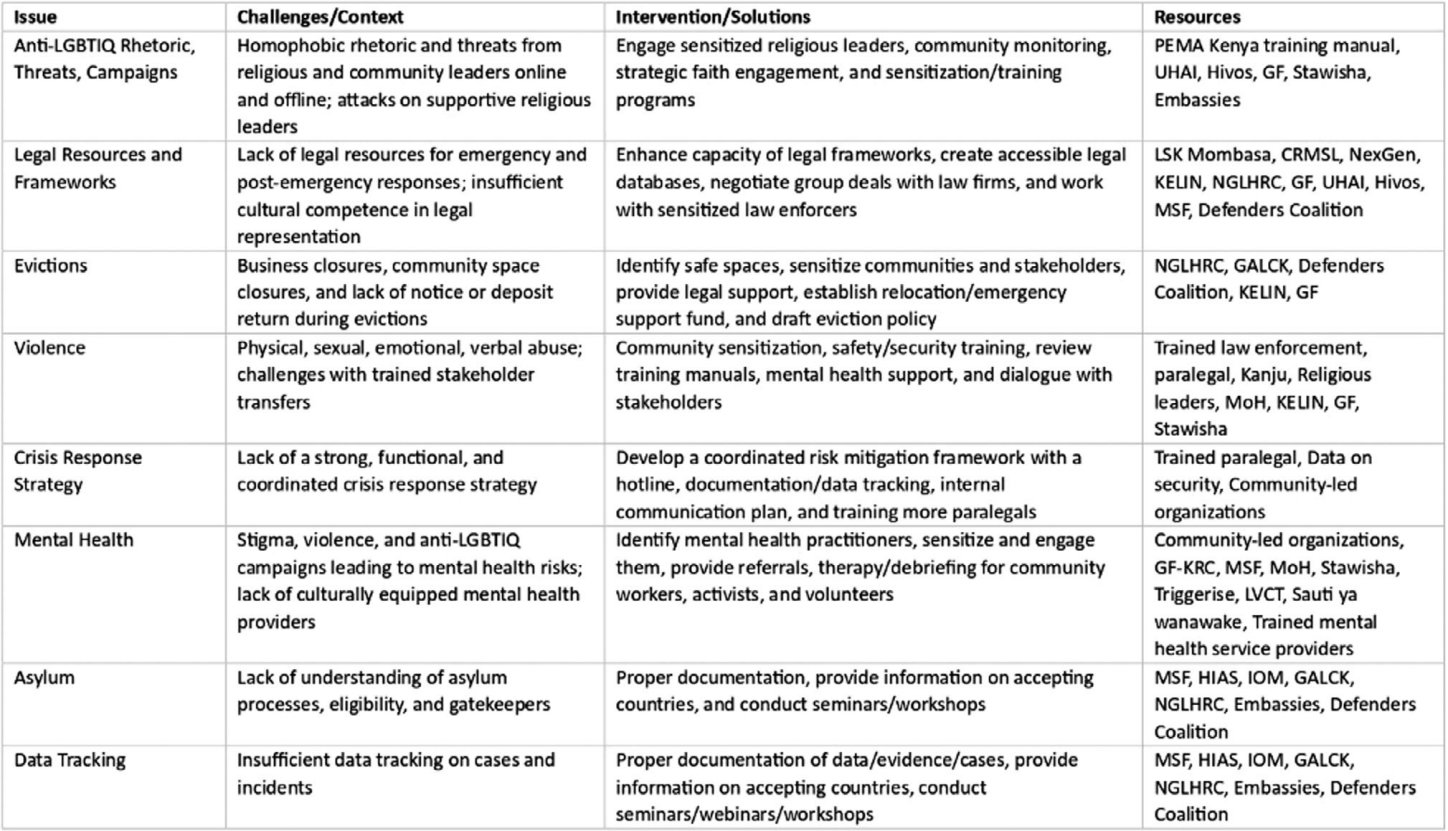

### OAF1105 Providing legal information through the chatbot “Legal Bot 100% Life” during full‐scale war and martial law on the territory of Ukraine

M. Ignatushyna^1^, K. Rivera ^1^

^1^CO “100 PERCENT LIFE”, Kyiv, Ukraine

**Background: **With the beginning of a full‐scale war on the territory of Ukraine, providing services for the prevention, testing and treatment of HIV became complicated. Two thousand four hundred and sixty‐four cases of AIDS were during 1 January 2023−1 October 2023 according to statistics by the Public Health Center of the MOH of Ukraine. The temporary loss of administrative control over part of the territory makes it impossible to obtain complete information about the level of damage to the entire population right now.

Many people with HIV and tuberculosis and representatives of vulnerable groups (hereinafter the Clients) do not have access to services, medicines and information due to hostilities.

**Methods: **A chatbot “Legal Bot 100% LIFE” (Chatbot) has been implemented by CO “100 PERCENT LIFE,” so the Clients can get legal information about the nearest legal offices and organizations providing social services. The Chatbot consists of 10 sections of answers now, including: HIV positive, How to get HIV treatment; Pregnant women and women in labour; Social issues; Privacy; Replacement therapy; Tuberculosis; Discrimination; Viral hepatitis and Martial Law (the last two was supplemented in response to the challenges of war in 2022−2023).

The Legal Bot became a comfortable way to obtain legal advice and useful information which helped clients to reduce the negative impact of hostilities and martial law.

**Results: **Clients have gotten 1928 consultations through it in 2022−2023. The consultations provided by it concerning issues of violation of rights to access to medical care, services, military service, treatment of HIV, hepatitis, tuberculosis and so on. The Chatbot is popular because it shows its effectiveness during limited access to information and services of human rights defenders in wartime conditions for key groups.

**Conclusions: **The Chatbot provides sustainability of legal services and online information about the possibility of receiving other services for the Clients using modern digital technologies during military operations and martial law since the situation in Ukraine requires the introduction of new digital solutions to overcome new challenges.

### OAF2702 Promoting access to HIV services among men‐who‐have‐sex‐with‐men under a harsh environment in the wake of the anti‐LGBTQ+ debate in Ghana

Y. C. Mensah ^1^, S. E. Owusu^2^, S. Owiredu Hanson^3^

^1^Heart for Help Foundation, Programs, Accra, Ghana, ^2^Maritime Life Precious Foundation, Kumasi, Ghana, ^3^CEPEHRG, Technical Coordinator, Accra, Ghana

**Background: **Men‐who‐have‐sex‐with‐men (MSM) in Ghana often face challenges accessing HIV services for reasons including stigma and discrimination. The introduction of the proposed anti‐LGBTQ+ bill by the Ghanaian parliament has further facilitated an environment where MSM constantly experience societal homophobia, fearmongering and human‐rights abuses which affect uptake and delivery of HIV services for MSM. Maintaining uninterrupted access to essential HIV services for MSM in the wake of the anti‐LGBTQ+ debate requires using integrated and community‐based strategies.

**Description: **Various community‐based approaches to HIV service delivery for MSM were introduced during the wake of anti‐LGBTQ+ debate in three districts.

Peer Educators halted conventional small and large group outreach and engaged peers with interpersonal one‐on‐one meetings to reduce public visibility. M‐Friends/M‐Watchers were deployed to facilitate access to needed legal and protective services; and address HIV‐related stigma and human rights abuses directed at MSM.

Flexible community‐based strategies and cross‐cutting initiatives were implemented to preserve access to HIV services and promote the safety of KP implementers and MSM during the period: (1) social media platforms were used to engage peers for HTS and support PLHIVs through virtual case management; (2) HTS and treatment took place at homes and safe locations identified and agreed to by peers at their own convenience; (3) condoms, lubricants and HIV self‐test kits were made available at community‐led DICs and outlets for easy access; (4) the programme promoted multi‐month dispensing of ART and PrEP to eliminate clinic visits.

**Lessons learned: **Introduction of community‐based strategies reached out to more MSM and increased HIV‐positive yield across the three districts. During the period of anti‐LGBTQ+ debate from September 2022 to February 2023, 690 new MSM were reached and tested for HIV; 49 were diagnosed positive. After the introduction of community‐based strategies, from March to August 2023, 834 new MSM were reached and tested; 141 were diagnosed positive.

**Conclusions/Next steps: **Community‐based approaches to HIV service delivery proved to be effective within the period of the anti‐LGBTQ+ debate. Nevertheless, the influence of the anti‐LGBTQ+ bill on HIV&AIDS programming is enormous affecting MSM and organizations offering services to them. We, therefore, call for a high‐level stakeholder advocacy on the effect of the anti‐LGBTQ+ bill against sexual minorities in the fight against HIV&AIDS.

### OAF2703 Strategies for HIV service continuity and risk mitigation for outlawed key populations in a restrictive legal environment

L. Oucul ^1^, E. Kamakune^2^, M. Nakimuli^3^

^1^The AIDS Support Organization (TASO), Programs and Capacity Development Directorate, Kampala, Uganda, ^2^The AIDS Support Organization, Grants Management Unit, Kampala, Uganda, ^3^Most At Risk Population Initiative (MARPI), Programs, Kampala, Uganda

**Background: **On 26th May 2023, the President of Uganda assented to the Anti‐Homosexual Act, which subsequently came into force on 30th May 2023. Within 2 months of the enactment of the law, The AIDS Support Organization (TASO) sub‐recipient, Human Rights Awareness and Promotion Forum (HRAPF) handled 141 cases involving LGBTIQ persons. 64.5% of these cases involved violence that targeted LGBTIQ person, 28 cases of evictions, and 15 cases of arrests (HRAPF report 23/June/2023). TASO is the second principle recipient of the Global Fund and we share strategies used for risk mitigation to ensure continuity of HIV services to the affected key populations (KPs).

**Description: **TASO and its Sub‐Recipients (SR) partnered with Ministry of Health (MoH) and Uganda AIDS Commission to implement an adaptation plan to enable continuity of HIV services in the midst of the restrictive legal environment. This involved sensitization of the key stakeholders at three levels (national, sub‐national and facility levels) on the impact of AHA on HIV services. At the national level, sensitization targeted parliament's HIV/AIDS committee. The sub‐national level targeted district and city leaders, while facility level targeted health workers, KP peers and local leaders. To assure health workers’ safety, the Ministry of Health was engaged to issue a circular to guide health workers. Joint meetings to strengthen linkage between HIV service providers and legal support agencies were conducted. Safety and security training workshops for civil society organizations and health workers were held. Standard operating procedures for client follow up and data collection were reviewed.

**Lessons learned: **HRAPF report shows 21% reduction of cases handled in October−December quarter compared to April−June quarter. The Director of Public Prosecutions issued a circular to guide all prosecutors on management of cases with charges preferred under the AHA. Strong linkage between HIV service providers and legal service providers greatly reduces the risk of arrests. The next steps will involve rolling out safe integrated key population information system managed by the key population consortium.

**Conclusions/Next steps: **It is possible to continue providing HIV services even in restrictive legal environment by mitigating security risks by engaging key stakeholders at all levels.

### OAF2704 Ensuring the right to breastfeeding for women and pregnant people with undetectable HIV viral load in 13 Latin American countries

M. Iacono ^1^, A. Luna^2^, K. Dunaway^3^

^1^National University of Lanus, Social Work (UNLA), Lanus, Argentina, ^2^National Autonomous University of Mexico, Historia, Mexico, Argentina, ^3^University of Buenos Aires, Social Studies, Buenos Aires, Argentina

**Background: **In 2010, the Pan American Health Organization, (PAHO) committed to eliminating mother‐to‐child transmission (EMTCT) of HIV and syphilis, establishing targets for 2015. This commitment persisted through the 2017 ETMI Plus initiative. Despite these efforts, breastfeeding for women with HIV remains forbidden and criminalized, needing guidance for decision‐makers on reproductive rights, specifically regarding breastfeeding for those with undetectable viral loads.

**Methods: **Data were collected in 2023 across 13 Latin American countries eligible to the Global Fund grants including Bolivia, Colombia, Costa Rica, Cuba, Ecuador, El Salvador, Honduras, Guatemala, Nicaragua, Paraguay, Peru, Dominican Republic and Venezuela, to understand the integration of national public policies on breastfeeding and HIV. Using stakeholder mapping, semi‐structured interviews with National AIDS Programs and documentary reviews; the study identified actors, processes and limitations within gender equality, reproductive rights and HIV responses.

**Results: **Diverse breastfeeding approaches for women with undetectable viral loads across the 13 countries, emphasizing risks and promoting formula substitution. Despite regional ETMI Plus adherence, no documented breastfeeding cases exist, posing policy challenges. Issues include sustaining pregnancy attention, HIV test result delays and control loss for HIV‐positive pregnant adolescents due to stigma. Economic policies in Venezuela affecting formula underscore PAHO collaboration needs for guideline updates. Recommendations stress enhancing care, addressing stigma, designing guidelines for indigenous women and ensuring comprehensive care, with calls for UN and Global Fund modifications to promote inclusivity. Regarding HIV criminalization laws, seven countries criminalize transmission, non‐disclosure or exposure to HIV, while four do not, relying on general criminal law. Cuba is an exception with no HIV criminal persecution, and one country lacks available data.

**Conclusions: **Ensuring reproductive rights, especially regarding breastfeeding, for women and pregnant individuals with HIV is imperative. The decision to substitute breastfeeding with formula milk, the only 0% HIV transmission risk option, should be stigma‐free. Urgent actions include case registration, advocacy for expanded feeding choices and updating guidelines for those with undetectable viral loads. Prioritizing economic, technical and political support, and placing the needs of women with HIV at the centre of the response, is essential in Latin America.

### OAF2705 Advocacy for drug policy reforms at high political level in the EECA region

O. Kucheruk ^1^

^1^Eastern and Central European and Central Asian Commission on Drug Policy, Mariestad, Sweden

**Background: **An enabling environment is critical for effective HIV response. Protective laws for people living with HIV and key populations contribute to better HIV outcomes, while criminalization of these populations and discriminatory laws undermine success. In the EECA region, drug legislation is primarily repressive and punitive, imposing severe criminal sanctions on people who use drugs. Massive criminalization prevents KPs from accessing health and social services.

**Description: **The Central and Eastern European and Central Asian Commission on Drug Policy (ECECACD) was initiated by Alexander Kwasniewski, former President of Poland and was created in 2021 gathering nine opinion leaders from the region in order to inspire better drug policy based on scientific evidence and human rights. The Commissioners are regional leaders from different professional arenas: former Heads of State, politicians, former members of Parliaments, diplomats, scientists and philanthropists from the region. The core role of the Commission is advocacy for better drug policy at the high political level.

During last 3 years, Commissioners have conducted country missions to Kyrgyzstan, Moldova, Lithuania, Georgia. During these visits, Commissioners have meetings with highest political leaders: Presidents of the states, Prime‐Ministers, Ministers of Health, Ministers of Internal Affairs, Members of the Parliaments, other top‐level authorities. It helped to get political commitment for drug policy reforms.

Currently, EU accession perspectives provide a great window of opportunities for the candidate countries: Moldova, Ukraine and Georgia. ECECACD provides expertise and support to governments to reform drug policies in compliance with EU requirements and standards.

**Lessons learned: **High political level advocacy resulted with political support to progressive reforms: (1) Seimas (Parliament of Lithuania) Committee on Legal Affairs approved amendments to the Administrative and Criminal Code that propose to decriminalize possession of small amounts of cannabis without intent to distribute it; (2) Committee on law enforcement of the Parliament of Kyrgyz Republic approved draft law that eliminate compulsory treatment for people who use drugs. Top politicians from other countries committed to make drug policy reform in their countries.

**Conclusions/Next steps: **The ECECACD aims to inspire an open and honest debate, and promote evidence‐based drug policy approaches across the region.

### OAF3102 Community activism to achieve access to housing, nutrition and transportation assistance for all low‐income people living with HIV

V. Shubert ^1^, C. King^2^

^1^Housing Works, Advocacy, Brooklyn, United States, ^2^Housing Works, Brooklyn, United States

**Background: **Evidence shows that safe, stable housing is essential to support effective antiretroviral treatment (ART) that sustains optimal health for people with HIV and stops ongoing transmission. In New York City (NYC), unstable housing has been found to be the single strongest predictor of poor HIV health outcomes and health disparities. Yet, few jurisdictions invest in social supports as HIV healthcare. As the result of persistent community activism over a 30‐year period, in 2016 NYC became the first jurisdiction to provide housing, nutrition and transportation assistance for every low‐income person with HIV.

**Description: **In the mid‐1980s, New York State (NYS) put in place a government‐funded rental assistance programme for people with HIV‐related illness, and New York City (NYC) supplemented this support with an HIV‐specific nutrition and transportation enhancement to existing cash transfer programmes for low‐income New Yorkers. Initially viewed as palliative care, the supports were limited to people with an AIDS diagnosis. With the advent of effective ART, these social protection programmes were recognized as critical enablers of HIV treatment. Extensive community advocacy over a 30‐year period beginning in 1988, including litigation, legislative lobbying and civil disobedience, gradually expanded the reach of the NYS/NYC‐funded social protection programmes, and in 2016, these social supports were expanded to all low‐income NYC residents with HIV experiencing homelessness and housing instability.

**Lessons learned: **On 31 December 2023, 27,472 New Yorkers with HIV (33% of all diagnosed) received public housing assistance. Among people with HIV in NYC in medical care, 88% were virally suppressed at the end of 2022, and 79% had sustained viral suppression from 2018 through 2022. Evidence‐based community activism is key to addressing HIV health inequities and ending HIV epidemics.

**Conclusions/Next steps: **As the first jurisdiction to provide access to meaningful housing supports for all low‐income persons with HIV, NYC has demonstrated the feasibility and effectiveness of addressing key social determinants of HIV health outcomes, and the vital role of community activism to achieve HIV health equity.

### OAF3103 Navigating opposition: understanding and responding to coordinated resistance against Comprehensive Sexuality Education and Sexual and Reproductive Health Rights

I. Zhukov^1^, E. Faur ^2^, H. McEwen^3^

^1^UNFPA, Sexual and Reproductive Health Branch, New York, United States, ^2^National University of San Martin (UNSAM), Argentina, ^3^University of the Witwatersrand, Wits Centre for Diversity Studies, Johannesburg, South Africa

**Background: **Resistance against comprehensive sexuality education (CSE) and sexual and reproductive health rights (SRHR) is a longstanding challenge, particularly fuelled by conservative opposition intersecting with youth, sexuality, gender and power dynamics. Recently, this opposition has gained traction, both within countries and at multilateral UN political levels. Despite global consensus on the positive impact of CSE, coordinated efforts aim to instigate doubt and distrust. This research explores the politicization of CSE, identifying patterns and trends in opposition, with a focus on countermovements describing themselves as “pro‐family” but labelled “anti‐gender” by concerned activists.

**Methods: **The research was conducted remotely in 2021−2022, engaging professionals working on CSE across the world. Semi‐structured interviews with key informants provided insights into anti‐CSE and anti‐SRHR advocacy. Participants from diverse stakeholder groups were interviewed, offering a comprehensive view. The study covered regions including Eastern and Southern Africa, Asia Pacific, Eastern Europe and Central Asia, Latin America and the Caribbean, Western and Central Africa, and the Arab States/Middle East and North Africa.

**Results: **The study identified “activated opposition” against CSE. Eastern and Southern Africa provides an important case study for observing how a “pro‐family” organization from the global north has mobilized a highly coordinated anti‐CSE regional advocacy campaign in the global south. West and Central Africa witnessed the politicization of CSE, with political leaders leveraging it for their agendas. The Arab States exhibited institutionalized opposition rooted in strong religious views. In Asia Pacific, governments opposed CSE, fearing it contradicted traditional family values. In Latin America, opposition stems from religious and cultural perspectives.

**Conclusions: **The research highlights the urgent need for additional evidence to support CSE, emphasizing the efficacy of its outcomes. Strategies include monitoring and responding to opposition, mapping allies, engaging media positively, mobilizing civil society, and understanding challenges in language and framing. Coordinated efforts between UN agencies, strategic partners, civil society and governments are crucial. The findings emphasize the necessity for a proactive and comprehensive approach to address the evolving landscape of opposition against CSE and SRHR, ensuring the protection and advancement of sexual and reproductive rights globally.

### OAF3104 A successful establishment of paralegals as human rights defenders towards ending HIV in Thailand

J. Siriphan ^1^, P. Duangmala^2^, S. Thunprom^3^, S. Bootchadee^3^, W. Kophaibunsiri^4^

^1^Foundation for AIDS Rights, Director, Bangkok, Thailand, ^2^Foundation for AIDS Rights, Manager, Bangkok, Thailand, ^3^Foundation for AIDS Rights, Coordinator, Bangkok, Thailand, ^4^Foundation for AIDS Rights, Lawyer, Bangkok, Thailand

**Background: **The lack of legal and human rights knowledge among populations with higher risk of being discriminated, including people living with/affected by HIV, is common in Thailand. Legal and rights services provided by the government is limited, often complicated and taking time. We aimed to (i) establish a cadre of paralegals to actively promote, prevent and protect human rights and (ii) document burden of human rights violation and discriminatory actions.

**Description: **The Foundation for AIDS Rights (FAR) focused on paralegals establishment due to clear limitations of dedicated legal professionals and government mechanisms to promote and protect human rights. FAR together with communities with lived experiences, legal and human right experts, developed the “Paralegals as Human Rights Defenders” curriculum. Key contents included (i) Concept of respect in human dignity and diversity, (ii) Knowledge of human rights basics and justice system in Thailand, and (iii) Skills on fact findings and case analysis. Civil society organizations (CSOs) were invited to identify members with potentials to become a paralegal—trusted by communities, ability to access communities and enthusiasm to empower their peers on human rights aspect.

**Lessons learned**:

- *A cadre of paralegals can be successfully established*. During January 2021–May 2023, 64 paralegals were trained. FAR provided technical support to and accepted case referral from these paralegals.
- *Documentation of human rights violations and discriminatory incidents can be made through the country's Crisis Response System (CRS)*. For HIV‐related incidents, 192 reports—mainly compulsory HIV testing in workplace, involuntary HIV status disclosure, denial of health and social services, were filed through trained paralegals.
- *Data were used for policy advocacy and public communications*. Common incidents were used by Thailand's National AIDS Subcommittee on AIDS Rights Protection and Promotion for policy change and public communication design.

**Conclusions/Next steps: **Deprofessionalization of legal and human rights services is crucial to empower people to know their rights, voice their discriminatory experiences and advocate for policy/law reforms. Paralegals can act as human right service providers to be integrated into community‐led health services in Thailand. Curriculum will be expanded and tailored for paralegals serving migrants and people who use drugs.

### OAF3105 Bridging the divide and channelling support for parliamentarians to influence the repealing OF “Bad” laws Africa, a case of the decriminalization of HIV transmission in Zimbabwe 2022

R. Labode ^1^

^1^Parliament of Zimbabwe, Parliamentary Portfolio Committee on Health and Child Care, Harare, Zimbabwe

**Background: **The criminalization of wilful transmission of HIV has been shown to perpetuate gender inequalities, increase stigma among people living with HIV and not effectively decrease HIV infections. Zimbabwe, with one of the highest HIV prevalence rates globally, has been significantly affected by the epidemic. The objective was to implement the recommendations of an HIV legal environment assessment, specifically repealing section 79 of the Zimbabwe Constitution, which criminalizes wilful transmission of HIV, and advocating for the release of prisoners incarcerated under the same law.

**Methods: **Partnerships were formed with parliament, the National AIDS Council, civil society organizations and development agencies such as UNDP and UNAIDS. Strategies included raising awareness through campaigns, conducting research for evidence‐based policies, organizing dialogues, capacity building of parliamentary champions for sexual reproductive health rights and holding public hearings in various provinces. Civil society organizations contributed their expertise and advocacy skills, while affected communities shared their lived experiences.

**Results: **The Minister of Justice successfully embedded the decriminalization of section 79 within a controversial Marriage Bill and presented it to Parliament for debate. Public hearings showed that 71% of attendees supported the motion to repeal the law. Legislators proposed advising the president to consider releasing all prisoners incarcerated under the same law.

**Conclusions: **Through policy advocacy and sustainable development initiatives, parliamentarians, together with development agencies, can collaborate to address HIV‐related issues and drive progress towards inclusive laws that address HIV. Strong partnerships between civil society organizations, the executive and legislators were key to achieving this change. This strategy can be replicated in efforts to change “bad” laws. Support for Zimbabwean parliamentarians in advocating for the release of prisoners incarcerated based on this repealed law is necessary.

### OAF4102 Brazilian network of young people living with HIV/AIDS: we are the answer—what adolescents and young people living with HIV/AIDS think about the access to health services in Brazil

L. Moura da Silva ^1^, M. K. Silva Rodrigues^2^, A. Ribeiro Ferreira^3^, S. Bispo Amaral^4^

^1^Brazilian Network of Adolescents and Young People Living with HIV/AIDS, Brasília, Brazil, ^2^Brazilian Network of Adolescents and Young People Living with HIV/AIDS, São Luís, Brazil, ^3^The Joint United Nations Programme on HIV/AIDS, Brasília, Brazil, ^4^United Nations International Children's Emergency Fund, Brasília, Brazil

**Background**: In Brazil, out of the 43,403 reported HIV cases in 2022, nearly half (41%) affected individuals between 15 and 29 years old, showing the need to prioritize actions to reach this age group. The Brazilian Network of Young People Living with HIV/AIDS (RNAJVHA) closely supported the mobilization and elaboration of the “We Are the Answer” study aiming to understand the obstacles to access healthcare through the experiences of young people living with HIV (YPLHIV) and develop strategies to enhance healthcare services aimed at this population.

**Methods**: The study, a collaboration between UNICEF, UNAIDS, civil society organizations and the private sector, employed a unique approach by integrating both quantitative and qualitative methods for data collection and analysis. A representative sample of YPLHIV from 18 to 29 years old (*n* = 710) participated in online interviews and the qualitative phase included interviews with RNAJVHA leaders (*n* = 7) and round table discussions (*n* = 70) in seven Brazilian capitals. These activities took place between March and April 2023, in which the participants were mobilized through websites and a peer‐to‐peer mobilization strategy. Round table discussions were facilitated by trained YPLHIV mobilizers.

**Results: **A fifth of respondents have experienced situations such as disrespect for privacy, discomfort during healthcare or feelings of guilt or shame for being a person with HIV/AIDS while using the Brazilian healthy system. Findings from the qualitative research align seamlessly with the quantitative findings, emphasizing stigma and discrimination among healthcare professionals as important barriers to treatment adherence. YPLHIV brought several recommendations for the improvement of services, such as: broaden access to information; provision psychological support; expanded and humanized healthcare; create and expand public campaigns to deconstruct the stigma of HIV/AIDS; include and expand the participation of YPLHIV in public policy decisions; and develop a protocol for communicating U = U.

**Conclusions: **The principle of GIPA and the policy of putting people at the centre of the response are some of the alternatives for strengthening public policies on HIV/AIDS. The community mobilization of YPLHIV for the preparation of this study strengthened the local and national leadership of RNAJVHA, which provided subsidies for the construction of public policies on HIV/AIDS in Brazil.

### OAF4103 HIV is not gender neutral: how can we track whether the International AIDS Conference represents the issues women and trans people living with HIV care about?

J. Ouma ^1^, C. Chung^2^, M. Tholanah^3^, K. Dunaway^4^, T. Otieno^5^, S. Petretti^6^, S. Strachan^7^, W. Thamm^7,8^, R. Mbewe^9^, B. Kasadha^10^, N. Policek^11^, L. Kwardem^9^, M. Rattue^9^, F. Murau^9^, L. Wanjiku Njenga^12^, O. Edwards^13^, S. Shahi^14^, M. Vazquez^15^, A. Welbourn^16^, E. Bell^17^, F. Hale^17^, The POWER Group

^1^Global Network of Young People living with HIV (Y+), Machakos, Kenya, ^2^The Transgender Law Center, Oakland, United States, ^3^Making Waves, Harare, United Kingdom, ^4^International Community of Women living with HIV (ICW), Buenos Aires, Argentina, ^5^Athena Network, Seattle, United States, ^6^Positively UK, London, United Kingdom, ^7^The Sophia Forum, London, United Kingdom, ^8^Hillingdon AIDS Response Trust, Hillingdon, United Kingdom, ^9^4M Mentor Mothers Network, London, United Kingdom, ^10^University of Oxford, Oxford, United Kingdom, ^11^European AIDS Treatment Group, Edinburgh, United Kingdom, ^12^Positive Young Women Voices, Dandora, Kenya, ^13^The Jamaican Community of Women living with HIV (JCW+), Kingston, Jamaica, ^14^ICW Asia Pacific, Bangkok, Thailand, ^15^Making Waves, Gijon, Spain, ^16^Salamander Trust, London, United Kingdom, ^17^Making Waves, London, United Kingdom

**Background: **International AIDS Conference (IAC) sets the global HIV agenda—so it is important to monitor whether priority issues of women and trans people living with HIV are covered at the IAC. We also track the use of stigmatizing language, to support the work of community networks and uphold the People First Charter.

**Methods**:

- Key terms were selected reflecting: gendered research on health and HIV treatment, meaningful engagement, psychosocial support, mental health, respectful and comprehensive services including sexual and reproductive health services (SRHS), freedom from violence and discrimination, protective laws, freedom from criminalization and economic justice.
- We searched the AIDS2022 Abstract Book for key terms and compared numbers to previous abstract books from 2016.
- We searched the online portal for abstracts using the same key terms, and IAS+.
- Oral abstracts were read for gendered intention, disaggregation and reflections relevant to women, girls and trans people living with HIV.

**Results: **Coverage of issues:

- There are very few mentions of IPV, GBV, VAWG, SRHR, GIPA, MIWA and (gender) transformative.
- Eighty five percent of oral abstracts were either overwhelmingly gender neutral, or focused on prevention or men.
- Eighteen (15%) oral abstracts had some intention to illuminate issues for women, girls and trans people living with HIV (through research aims or disaggregation) and gendered reflections.

Stigmatizing language:

- The use of “infect*” remains extremely high. Use of “infected” has reduced. Use of the acronym WLHIV has increased from 11 mentions in 2016 to 541 in 2022.

Challenges with tracking:

- The abstract book search does not give the number of abstracts using each term.
- The online portal search does not allow for multiple‐word terms, and does not return more than 200 results.
- Abstract submission keywords are inadequate.
- Manual oral abstract search was time‐consuming, and lacked precision.

**Conclusions**:

- Tracking coverage of issues is problematic, undermining accountability of IAC to women, girls and trans people living with HIV. This should be addressed.
- Our issues are still inadequately covered. Research with clear gendered intentions, results and reflections is rare.
- Stigmatizing language is widely used.
- The alert on abstract submissions for stigmatizing language is a welcome response to our longstanding requests.
- IAS should continue to encourage and prioritize gendered research.

### OAF4104 Facilitating adolescent access to HIV interventions through age of access policy reform

S. Lynch^1^, V. Srivatsan^1^, A. Banda^1^, A. Sharma^1^, M. Kavanagh ^1^

^1^Center for Global Health Policy and Politics, O'Neill Institute for National and Global Health Law, Georgetown University, Washington, DC, United States

**Background: **The science informing the fight against HIV/AIDS is the most advanced it has ever been, and yet adolescent populations continue to remain particularly affected. An enabling national policy environment should include alignment with WHO guidelines on the age of access (AOA) to HIV testing and treatment. WHO recommends eliminating parental consent requirements to access HIV services for adolescents aged 12 years and above.

Here, we examine national AOA policies by evaluating if adolescents can access HIV testing and treatment without parental consent.

**Methods: **The HIV Policy Lab, a collaboration between Georgetown University, UNAIDS and the Global Network of People Living with HIV (GNP+), tracks the adoption status of 33 globally recommended laws and policies for 194 countries. The database evaluates whether adolescents (≥12 years) can access HIV testing and treatment services without parental consent, in alignment with the WHO recommended policy.

**Results: **In our preliminary analysis, we found relevant guidelines for 109 countries. Among them, only 25% (28/109) countries have adopted optimal AOA policies.

Testing policies: Eighteen percent (16/89) countries do not have specific AOA guidelines, 40% (35/89) require parental consent for individuals ≤18 years, 14% (13/89) countries require parental consent for those below 16 years and 14 years, respectively. Twenty‐six countries have created policy exceptions for HIV testing, including demonstrated maturity, pregnancy, emancipated minors and sexual activity.

Treatment policies: 29.5% (21/71) countries lack AOA guidelines, 39.4% (28/71) countries have policies that require parental consent for adolescents ≤18 years, 21.1% (15/71) countries require consent for adolescents ≤16 years and 7% (5/71) countries require consent for adolescents ≤14 years.

In sub‐Saharan Africa, the region with the highest number of children and adolescents living with HIV (CALHIV), only 11 countries have adopted WHO AOA recommendations. Among 10 countries with the largest CALHIV population, only Mozambique and Lesotho have not adopted optimal AOA policies.

**Conclusions: **Many countries are out of step with WHO recommendations on AOA to HIV testing and treatment. Policy reform is needed in these countries to facilitate adolescent‐centric HIV care and enable adolescents to make informed decisions about their health.

### OAF4105 Supporting caregivers to care for children living with HIV in South Africa

N. Heath ^1^, D. Heath^2^

^1^Zoe‐Life, Training, Westville, South Africa, ^2^Zoe‐Life, Training, Durban, South Africa

**Background: **In 2016, 320,000 children aged 0−14 years were living with HIV in South Africa, the majority of whom acquired HIV through vertical transmission. However, 45% of these children were not enrolled in ART as a high number of children are lost to follow up after 12 months of age. HIV disclosure in South Africa is hindered by significant stigma and discrimination associated with HIV, leading to fear of rejection, isolation, and bullying for children and their families. Children and their caregivers often lack accurate knowledge about HIV, making effective disclosure challenging.

**Description: **KidzAlive@Home, implemented by Zoë‐Life SA with support from Aidsfonds (2019−2022), enhances HIV identification, testing, treatment and care retention for children in South Africa. Operating in eThekwini and uMgungundlovu districts, it collaborates with trusted community structures like faith‐based and community‐based organizations. The programme offers caregivers resources like the KidzAdherence curriculum and Talk Tool, simplifying treatment literacy. Healthcare workers utilize these tools for age‐appropriate HIV education, disclosure support and counselling. Child‐friendly spaces within health facilities facilitate learning in a comfortable environment, contributing to improved care for children living with HIV.

**Lessons learned: **Knowledge must be translated into action. Caregivers need support to translate knowledge into action which requires tools that are child‐friendly and easy to understand. Creating comfort in child‐friendly spaces is critical to engaging caregivers and children themselves in their own care. Child‐friendly spaces and household visit create trust and comfort so that healthcare workers can alleviate fears, support disclosure and help to translate knowledge. Kidzadherence clubs help children to understand their diagnosis and reduce fear through connection with other children living with HIV increasing agency in their own care.

**Conclusions/Next steps: **Zoë‐Life's success with the KidzAlive@Home project has led to the government's endorsement of the approach as an intervention to improve children's health outcomes. Zoë‐Life wants to continue to build capacity of community‐based organizations within and outside of South Africa through quality assurance and quality improvement, strengthening the structures that support families so that care can be accessible and effective for the long‐term.

## LATE BREAKING ABSTRACTS

### OAA0206LB Factors influencing time to viral rebound during analytical treatment interruptions in HIV cure trials

V. Klastrup ^1,2^, J.D. Gunst^1,2^, T.A. Rasmussen^1,2^, M. Tolstrup^1,2^, O.S. Soegaard^1,2^

^1^Aarhus University, Department of Clinical Medicine, Aarhus, Denmark, ^2^Aarhus University Hospital, Department of Infectious Diseases, Aarhus, Denmark

**Background: **Achieving sustained control of HIV replication in the absence of ART is an important goal in HIV cure research. To identify factors associated with time to detectable viremia and time to loss of virologic control, we conducted a meta‐analysis among participants in 6 interventional HIV cure trials.

**Methods: **Each of the 6 trials included an analytical treatment interruption (ATI). We determined factors influencing time to detectable viremia (defined as plasma HIV‐1 RNA ≥ 50 copies per ml) and time to loss of virologic control (defined as either restart of ART or two consecutive measurements of plasma HIV‐1 RNA ≥ 5,000 copies per ml) using cox proportional hazard regression to calculate hazard ratios.

**Results: **Among the 114 included participants median age was 47 years (range: 22 to 68 years). The trials investigated the following interventions alone or in combination: broadly neutralizing antibodies (bNAb) (3BNC117, 10–1074), histone deacetylase inhibitors (HDACi) (romidepsin, panobinostat), HIV‐1 peptide vaccine (Vacc‐4x) and toll‐like receptor 9 (TLR9) agonist (MGN1703). We found that pre‐ATI total HIV‐1 DNA ≥ 850 copies as well as intact proviral DNA ≥ 70 copies per 10^6^ CD4+ T cells were associated with shorter time to detectable viremia (HR = 1.94, 95% CI: 1.27, 2.98; HR = 1.75, 95% CI: 1.01, 3.00). Total HIV DNA ≥ 850 copies per 10^6^ CD4+ T cells also predicted shorter time to loss of virologic control; as did time from diagnosis to ART ≥ one year (HR = 1.60, 95% CI: 1.05, 2.45; HR = 1.56, 95% CI: 1.02, 2.39). HDACi treatment seemed to predict shorter time to loss of virologic control, whereas bNAb treatment at ART initiation of individuals harboring 3BNC117‐sensitive viruses was associated with delayed time to loss of virologic control (HR = 1.69, 95% CI: 1.09, 2.62; HR = 0.26, 95% CI: 0.09, 0.73).

**Conclusions: **Our findings shed new light on factors influencing time to viral rebound as well as loss of virologic control. These findings can inform the design of novel cure trials but also highlight the potential impact of early bNAb treatment on virological control during ATI.

### OAA0606LB HIV infection alters the breastmilk virome of mothers living with HIV and the gut virome of related infants through early life

B. Brown ^1,2^, B. Maust^1,2^, C. Feng^2^, A. Happel^3^, S. Minot^4^, A. Varsani^5,3^, H. Jaspan^1,3,2^

^1^University of Washington, Pediatrics, Seattle, United States, ^2^Seattle Children's Hospital, Seattle, United States, ^3^University of Cape Town, Cape Town, South Africa, ^4^Fred Hutch Cancer Center, Seattle, United States, ^5^Arizona State University, Phoenix, United States

**Background: **Infants born to mothers living with HIV that remain uninfected (HEU) experience higher risk of morbidity and mortality, reduced responses to vaccination, and altered gut bacterial microbiota. However, little is known regarding the effect of maternal HIV infection on the establishment and composition of the infant gut virome.

**Methods: **From a cohort of South African mothers living with HIV and related HEU infants (n = 40 pairs) and an age‐matched control group of mothers not living with HIV and their infants (n = 40 pairs), we undertook viral DNA and RNA metagenomic sequencing of purified viral particles. Viral and bacterial communities were analyzed in maternal stool and breast milk and infant stool through the first 36 weeks of life. Vaccine responses were measured via multiparameter flow cytometry and bacterial communities were profiled using 16S rRNA gene sequencing.

**Results: **We find that maternal HIV status is significantly associated with distinct composition of the breast milk (F = 3.28, P < 0.001) and gut viromes of related infants (F = 1.93, P = 0.004), relative to controls. HEU infants also display 11‐fold increased relative abundance of putative *Bifidobacteria* bacteriophages (P < 0.001) and a concomitant 24‐fold reduction in the abundance of *Bifidobacteria* (P < 0.001) in the first week of life, relative to unexposed infants. *Bifidobacterium longum* abundance in the first week of life was significantly positively correlated with later responses to Bacille Calmette‐Guerin (BCG) vaccination (R^2^ = 0.35, P = 0.002), which was contrasted by a significant inverse correlation between the abundance of a eukaryotic DNA virus within the *Smacoviridae* during the first week and BCG responses (R^2^ = 0.12, P = 0.04).

**Conclusions: **Mothers living with HIV have significantly different composition of the breast milk virome, and the gut virome of their related infants also significantly differs compared to infants not exposed to HIV. These shifts include increases in the relative abundance of putatively *Bifidobacteria*‐infecting bacteriophages and reductions in the relative abundance of *Bifidobacteria* in HEU infants relative to unexposed infants. These results provide early insights into the effect of maternal HIV infection on the establishment of the infant gut virome and its relationship to bacterial microbiota and responsiveness to vaccination.

### OAA1306LB Potent and broadly neutralizing HIV‐1 antibodies with improved pharmacokinetics achieved by negative supercharging

Y.D. Kwon ^1^, M. Bender^1^, E.S. Yang^1^, K. McKee^1^, S. O'Dell^1^, E. Tourtellott‐Fogt^1^, I‐T. Teng^1^, D. Wang^1^, A. Pegu^1^, T. Zhou^1^, N. Doria‐Rose^1^, T. Pierson^1^, R. Koup^1^, P. Kwong^1^

^1^National Institutes of Health, Vaccine Research Center, Bethesda, United States

**Background: **Our previous work demonstrated that reducing the net charge of the variable domain of HIV‐1 antibodies diminishes off‐target binding and enhances pharmacokinetics (PK). In this study, we investigated whether reducing the net charge by substituting select Arg or Lys with Gln or Glu in the first constant Ig domain of the heavy chain (CH1) and the constant domain of the light chain (CL), or by incorporating various acidic regions present in human protein molecules into the C‐termini of the heavy and light chains, could enhance the PK of HIV‐1 antibodies.

**Methods: **We engineered a panel of HIV‐1 antibody variants with Arg or Lys residues systematically substituted with Gln or Glu in the CH1 and CL domains in the presence or absence of acidic regions incorporated into the C‐termini of the heavy and light chains and assessed neutralization potency against a 12‐virus panel and PK in Tg32 hFc mice.

**Results: **Our findings revealed that K129E, K210E, and K214E substitutions in the CH1, and K128E, K147E, K190E, and K192E substitutions in the CL improved the PK of VRC01.23LS, VRC07‐523LS, and N6LS while maintaining potency. Antibody variants with their net charge optimized in both the variable and CH1/CL domains displayed significantly extended PK compared to variants with substitutions in either the variable or constant domain alone. Interestingly, incorporating the acidic tail of alpha‐synuclein (ATS) into the C‐termini of the heavy and light chains of HIV‐1 antibodies also enhanced PK without compromising potency. Furthermore, when the acidic tails were added to the charge‐optimized variants, not only did PK further improve, but the potency of VRC01.23LS, VRC07‐523LS, N6LS, PG9LS, PGT128LS, 3BNC117LS, 10–1074LS, and VRC34.01LS_mm28 increased unexpectedly by 6‐ to 10‐fold compared to their wild‐type counterparts.

**Conclusions: **While confirmation is needed regarding whether the enhanced potency observed in *in vitro* pseudovirus assays translates to improved protection in non‐human primates, the conservation of the CH1 and CL domains in IgG1 suggests that these substitutions in the CH1 and CL, along with the addition of acidic tails, have the potential to enhance the PK and potency of various therapeutic antibodies.

### OAA2806LB Spatial characterization and phenotypic profile of macrophage HIV reservoirs in lymph node and gut tissues from subtype C HIV infection

M. Moodley ^1^, C. Chasara^1^, T. Khaba^2^, S. Nxele^1,2^, B. Mahlobo^1^, Z. Ndhlovu^1,2,3^

^1^Africa Health Research Institute (AHRI), University of KwaZulu‐Natal (UKZN), Durban, South Africa, ^2^HIV Pathogenesis Programme (HPP), University of KwaZulu‐Natal (UKZN), Durban, South Africa, ^3^Ragon Institute of MGH, MIT and Harvard, Cambridge, United States

**Background: **HIV Cure is impeded by the incomplete characterization of all sources of rebound‐competent HIV reservoirs to direct targeted eradication therapies. Lymph node (LN) and gut tissues are key sites of HIV reservoir persistence, where most research focuses on follicular helper T cell reservoirs. Emerging evidence reveals that macrophages may also harbour HIV, however there are controversies regarding their role as productively HIV infected reservoirs, partially due to their phagocytic role. This study aimed to characterize macrophage subtype C HIV reservoirs in human lymph node and gut tissues in terms of their phenotype, location, and their potential for sustained productive infection during suppressive antiretroviral therapy (ART).

**Methods: **Formalin‐fixed paraffin‐embedded LN and gut tissues from 20 people living with HIV (subtype C) in South Africa obtained from the FRESH and the HPP lymph node cohorts. Multi‐colour immunofluorescence microscopy combined with in‐situ hybridization RNAscope was employed to characterize and localize macrophage sub‐populations containing HIV protein antigens and viral RNA. High‐resolution oil‐immersion microscopy was used to distinguish between macrophages that were productively infected and those that had ingested infected CD4+ T cells.

**Results: **Lymph node germinal centre (GC) CD68+ macrophages harboured both HIV gag‐p24 protein and HIV gag‐pol RNA. In HIV infection, the frequency of LN CD68+ macrophages was elevated (**p = 0,0039**). The density of GC CD68+P24+ macrophages was higher in late‐treated compared to early‐treated PLWH. There was a strong positive correlation between the density of GC CD68+P24+ macrophages and plasma viral load in late‐treated individuals (**p = 0,0167; r = 1**). High‐resolution imaging techniques revealed that phagocytic macrophages exhibited intracellular staining for CD4+ T cells, and were distinctively localized outside the GCs. In contrast, productively infected macrophages within GCs displayed gag‐p24 co‐localization in the absence of intracellular CD4 ingestion. In the gut, CD68+ macrophages harboured HIV gag‐pol RNA and gag‐p24 protein in the lamina propria and Peyer's Patches.

**Conclusions: **This study reveals that CD68+ macrophages are productively HIV infected tissue reservoirs, capable of contributing to viral rebound. These findings offer significant insights into the spatial distribution and characteristics of macrophage‐associated reservoirs, establishing a basis for developing targeted strategies aimed at eliminating these reservoirs in LN and gut tissues.

### OAA3506LB Natural killer cell in lymph nodes; phenotype, location, and function during acute infection and how HIV modulates their effector functions

M. Mlaba ^1,2,3^, Z. Ndhlovu^4,5,1^, N. Ngema^1^

^1^Africa Health Research Institute (AHRI), Durban, South Africa, ^2^University of KwaZulu‐Natal, Medical Microbiology, Durban, South Africa, ^3^HIV Pathogenesis Programme (HPP), Durban, South Africa, ^4^Havard, Immunology, Cambridge, United States, ^5^Ragon Institute of Mass General, MIT & Havard, Immunology, Cambridge, United States

**Background: **The persistence of HIV reservoirs in lymph node (LN) germinal centres (GC) despite HAART remains a barrier to achieve complete eradication of HIV. There is growing evidence that lymph nodes lack cytolytic responses, that may be linked to poor infiltration by dysfunctional cytolytic T cells. Natural killer (NK) cells represent the cytolytic arm of innate immunity with CXCR5+ NK cells having been correlated to decreased viral burden SIV infected LNs. However, there remains a paucity of information concerning migration and cytotoxicity of NK cells in human LNs. Therefore, our aim was to assess the presence and functional capabilities of these CXCR5+ NK cells during treated HIV infection in the lymph node using immunofluorescent staining assays and flow cytometry.

**Methods: **PBMC and lymph node tissues (formalin‐fixed paraffin‐embedded tissue and dissociated LMC) were obtained from the HIV Pathogenesis Programme (HPP) lymph node study (LNS) and FRESH cohort, Durban, South Africa. Frozen PBMC and LNC were phenotypically and functionally characterised using flow cytometry analysis with standardized surface stain and ICS protocols, while LN tissues were imaged and analysed using Immunofluorescence microscopy.

**Results: **NK cells identified in the tissues of ART‐treated PLWH lack CXCR5 expression required for migration into GC where we have showed HIV persists. Furthermore, there were significantly more CXCR5‐ NK cells (p = 0,0087) when compared to CXCR5+ NK cells in HIV infected individuals. Further, the NK cells were consistently found to localise outside the GCs. These NK cells also exhibited reduced expression of cytolytic markers CD107a and granzyme B. Additionally, while the overall frequency of CD56+ NK cells remained steady during HIV infection, HIV‐ samples displayed a notably higher density of CD56+ CD16+ NK cells (p = 0,0159) compared to infected individuals.

**Conclusions: **The observed localization outside the GC and the reduced expression of cytolytic markers in NK cells are noteworthy and parallels similar observations in CD8+ T cells, suggesting potential common mechanisms contributing to HIV persistence in tissues. This study contributes to a more comprehensive understanding of how HIV‐1 infection impacts the immune system, particularly NK cell biology, during treatment.

### OAB0106LB Efficacy of dolutegravir/lamivudine (DTG/3TC) in adults with HIV‐1 and isolated reactive hepatitis B core antibody (anti‐HBc): results from the phase 3/3b GEMINI‐1/‐2, STAT, TANGO, and SALSA studies

D. Fox ^1^, J. Slim^2,3^, E.T. Overton^1^, A. Doblado‐Maldonado^4^, P. Jeffery^5^, R.A. Grove^5^, C.M. Parry^6^, M. Underwood^1^, B. Jones^6^

^1^ViiV Healthcare, Durham, United States, ^2^New York Medical College, Valhalla, United States, ^3^Saint Michael's Medical Center, Newark, United States, ^4^ViiV Healthcare, Wavre, Belgium, ^5^GSK, Brentford, United Kingdom, ^6^ViiV Healthcare, Brentford, United Kingdom

**Background: **As 2‐drug antiretroviral therapy regimens emerge, clinical management of people living with HIV‐1 and isolated reactive anti‐HBc is important. In real‐world studies of people living with HIV‐1 switching to 2‐drug regimens, reactive anti‐HBc was associated with lower HIV‐1 suppression rates but not elevated liver enzymes or hepatitis B virus (HBV) reactivation. However, further evaluation is warranted. We present outcomes for participants with isolated reactive anti‐HBc receiving DTG/3TC vs comparator regimens in phase 3/3b studies.

**Methods: **This analysis includes individuals with past HBV exposure (reactive anti‐HBc), no evidence of active HBV infection, and non‐reactive HBV surface antibody among treatment‐naive participants in GEMINI‐1/‐2 and STAT and virologically suppressed participants in TANGO and SALSA.

**Results:**

Overall, 46 participants in GEMINI‐1/‐2, 5 in STAT, 13 in TANGO, and 12 in SALSA had isolated reactive anti‐HBc. Proportions of participants with HIV‐1 RNA <50 c/mL or HIV‐1 RNA <40 c/mL and target not detected were generally high and comparable between treatment groups across all studies; few participants experienced HIV‐1 RNA ≥50 c/mL (Figure). Liver function test toxicities were reported in 10/23 (43%) participants receiving DTG + 3TC in GEMINI‐1/‐2 and 3/16 (19%) receiving DTG/3TC in TANGO/SALSA; most were grade 1 or 2. Across studies, 1 participant receiving DTG + 3TC in GEMINI‐1/‐2 had hepatitis E virus infection and liver enzyme elevations that met liver‐stopping criteria, which led to treatment discontinuation at ∼144 weeks and study withdrawal. Adverse events typically associated with HBV were reported in 5/23 (22%) participants receiving DTG + 3TC in GEMINI‐1/‐2 and 1/9 (11%) receiving DTG/3TC in TANGO. No HBV reactivation was reported in any study.

**Figure**. OAB0106LB

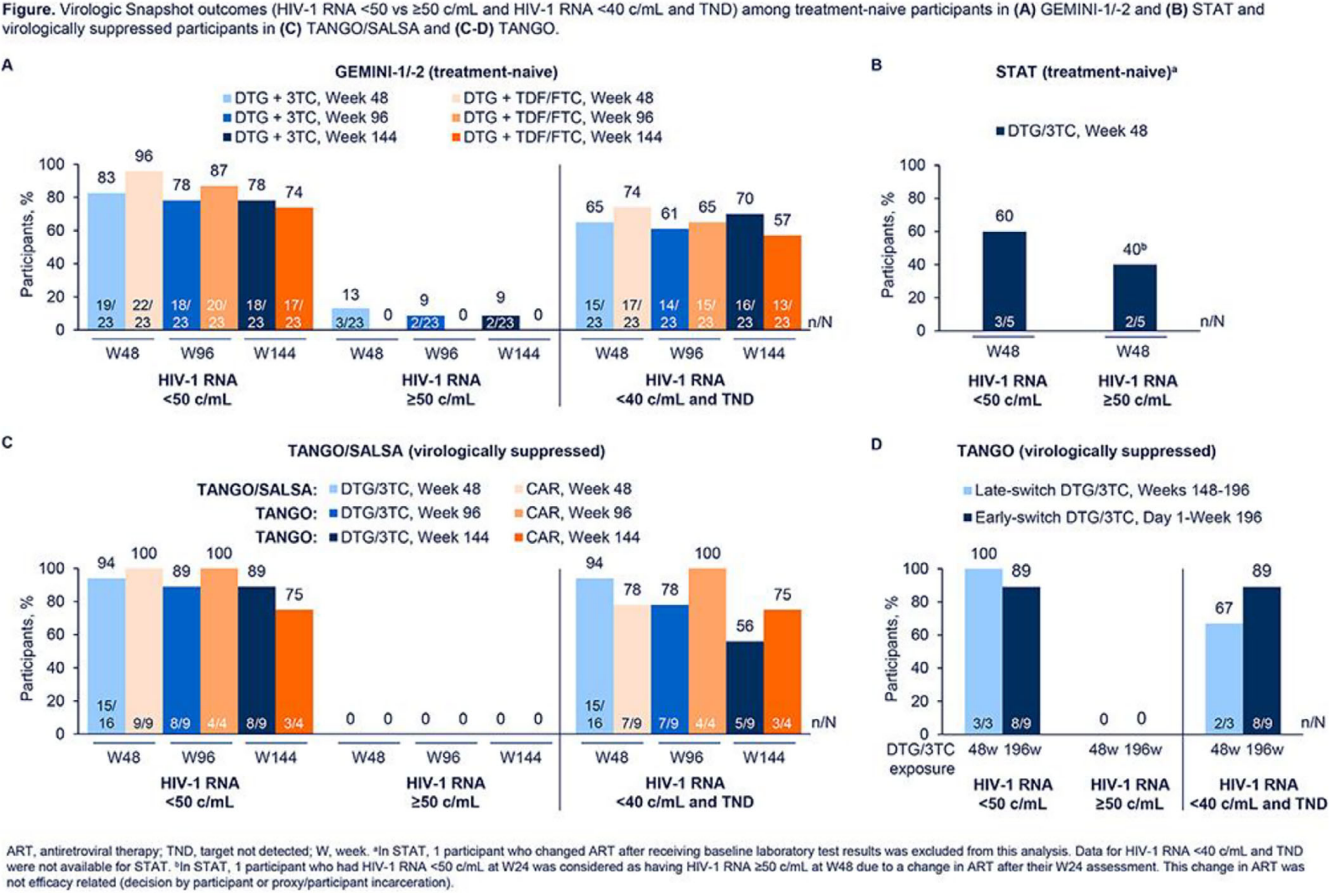

**Conclusions: **Among people living with HIV‐1 with isolated reactive anti‐HBc, DTG/3TC demonstrated high and similar HIV‐1 suppression rates vs 3‐/4‐drug comparator regimens, with few liver enzyme elevations leading to discontinuation and no reports of HBV reactivation.

### OAB1706LB Knowing the true prevalence of tuberculosis (TB) in Nigeria: a call for the bi‐directional diagnostic approach to TB detection

D.S. Adeniyi ^1^, M.D. Agwo^2^, L.K. Biringmiap^3^, G.A. Dapyen^4^, P.C. Okonkwo^1^, S. Wekpe^1^, L.S. Madukaji^5^, F.E. Owolagba^5^, E. Ofuche^5^, I. Onwuatuelo^5^, M. Okoye^6^, J. Samuels^5^, P. Okonkwo^5^

^1^APIN Public Health Initiatives, Directorate of Laboratory Services, Jos, Nigeria, ^2^SMOH, TB Unit, Jos, Nigeria, ^3^PLASU, Medical Laboratory, Bokkos, Nigeria, ^4^SMOH, Epidemiology Laboratory, Jos, Nigeria, ^5^APIN Public Health Initiatives, Laboratory Services, Abuja, Nigeria, ^6^US CDC, Global HIV & TB, Abuja, Nigeria

**Background: **Tuberculosis (TB) is an infection which primarily attacks the lungs. Currently in Nigeria, emphasis is laid on initial diagnosis made on the Cepheid GeneXpert® system. The molecular technologies employed on the GeneXpert® allows for the quick and easy detection of TB infection in sputum samples. However, with Nigeria still leading Africa in TB prevalence, and in the number of undetected cases, diagnosing presumptive TB clients with only the GeneXpert® system becomes inadequate in the presence of Extra‐pulmonary TB (EPTB). This study seeks to highlight the comparative benefits of applying a bi‐directional diagnostic approach to TB screening in Nigeria.

**Methods: **In this cross‐sectional study, a total of 1,415 randomly diagnosed Advanced HIV Disease (AHD = CD4<200 cells/mm^3^) clients in Plateau State, North‐Central Nigeria were tested for the *Mycobacterium tuberculosis (Mtb) lipoarabinomannan glycolipid* using the Urine TB LF‐LAM® test kit. This study took place from October 2022 to December 2023. Clients who were positive with the Urine TB LF‐LAM® further produced sputum samples which were used for the MTB/RIF GeneXpert® testing. Obtained data were analyzed using simple descriptive statistics.

**Results: **From the 1,415 AHD clients in this study, 91/1,415 (6.4%) tested positive with the Urine TB LF‐LAM®; while 17/91 (18.7%) were *Mtb* detected on the GeneXpert® machine. 74/91 (81.3%) EPTB and 17/91 (18.7%) Pulmonary TB (PTB) prevalence were recorded respectively.

**Conclusions: **Considering the current national emphasis on GeneXpert® screening for initial TB diagnosis, results obtained in this study indicates a large number of presumptive TB clients with EPTB are largely left undiagnosed. It is recommended that all presumptive TB clients be bi‐directionally diagnosed using the Urine TB LF‐LAM® and the GeneXpert® screening tests. This double‐pronged approach will help to optimize the TB case detection in Nigeria.

**Key Words**: *TB, EPTB, AHD, Nigeria, Urine TB LF‐LAM®, GeneXpert®*

### OAB2106LB Universal HIV testing of children at 18 months of age in South Africa: a novel policy as the last mile to close the pediatric case finding towards HIV epidemic control

T. Silere‐Maqetseba ^1^, k. Khosa^2^, A. Lenders^3^, D.B. Mugisa^4^, H. Mabasa^4^, O. Mushakarara^4^, K. Kehoe^4^, J. Chehab^4^

^1^National Dept of Health, Child Health, Pretoria, South Africa, ^2^National Dept of Health, Maternal and Neonatal Health, Pretoria, South Africa, ^3^National Dept of Health, HIV/AIDS and TB unit, Pretoria, South Africa, ^4^United States Agency for International Development, South Africa, Paeds HIV, PRETORIA, South Africa

**Background: **South Africa is the first country to adopt a policy on universal HIV testing for all children aged 18 months aligned to the Vertical Transmission Program and the Expanded Programme on Immunization. This policy was adopted in 2019 and is aimed at improving the pediatric HIV case finding in facilities and communities. Based on NAOMI HIV estimates, South Africa has the largest pediatric HIV epidemic globally, with an estimated 152,984 children <15 years living with HIV in 2024^1^.

**Methods: **We conducted a retrospective review of program data from 2018 to 2023, District Health Information System (DHIS II). Data is based on the Government of South Africa financial year (April to March). Data was for children aged 18 months: i) HIV tests, ii) Hexa‐4 vaccinations, iii) live births 18 months prior to the review period, and iv) census estimates for age one year.

**Results: **There was a 48% increase in the number of children tested annually from 238,392 in 2018 to 352,827 in 2023. The proportion of children with a recorded vaccination who were tested for HIV increased from 32% in 2018 to 45% in 2023, while HIV positivity decreased from 0.6% to 0.3% over the same period. Over the five‐year period, 36% (1.35 million) of the 3.8 million children receiving the Hexa‐4 vaccine were tested for HIV. The Hexa‐4 vaccination coverage was 69.7% compared to the estimated population.

**Conclusions: **Our findings indicate scale‐up of integrated EPI and HIV testing services at 18 months of age. However, these data also highlight missed opportunities for universal testing currently. This novel policy is critical to closing the pediatric HIV case finding gaps, key on identifying slow progressors, and children with disadvantaged backgrounds who do not present to the healthcare facilities.

### OAB2606LB Long‐acting cabotegravir (CAB) plus rilpivirine (RPV) in the first, virologically‐suppressed adolescents with HIV‐1 to receive an every 8‐week, all‐injectable regimen in a multicenter, multinational Study: IMPAACT 2017 week 48 outcomes

A. Gaur ^1^, K. Baltrusaitis^2^, E. Capparelli^3^, J. Moye^4^, D. Yin^5^, A. Violari^6^, B. Heckman^7^, S. Buisson^8^, R. Van Solingen‐Ristea^9^, C. Harrington^10^, M. Marzinke^11^, E. Lowenthal^12^, S. Ward^13^, R. Milligan^7^, B. Best^3^, E. Townley^5^, A. Agwu^11^, C. McCoig^14^, G. Roberts^15^, J. Huang^16^, A. Cheung^17^, H. Crauwels^9^, V. Van Eygen^9^, C. Krotje^7^, S. Zabih^18^, G. Masheto^19^, P. Ounchanum^20^, L. Aurpibul^21^, V. Korutaro^22^, C. Bolton Moore^23^

^1^St. Jude Children's Research Hospital, Infectious Diseases, Memphis, United States, ^2^Center for Biostatistics in AIDS Research, Harvard T.H. Chan School of Public Health, Boston, United States, ^3^University of California San Diego, La Jolla, United States, ^4^Eunice Kennedy Shriver National Institute of Child Health and Human Development (NICHD), Bethesda, United States, ^5^National Institute of Allergy and Infectious Diseases, National Institute of Health, Rockville, United States, ^6^Baragwanath Academic Hospital, Johannesburg, South Africa, ^7^Frontier Science Foundation, Amherst, United States, ^8^FHI 360, Durham, United States, ^9^Janssen Research and Development, Beerse, Belgium, ^10^ViiV Healthcare, Research Triangle Park, United States, ^11^Johns Hopkins University School of Medicine, Baltimore, United States, ^12^University of Pennsylvania Perelman School of Medicine, Children's Hospital of Philadelphia, Philadelphia, United States, ^13^Frontier Science Foundation, Brookline, United States, ^14^ViiV Healthcare, Madrid, Spain, ^15^SMG Pharma Safety GlaxoSmithKline, Middlesex, United Kingdom, ^16^GlaxoSmithKline, Mississauga, Canada, ^17^Certara, Princeton, United States, ^18^University of California Los Angeles, Los Angeles, United States, ^19^Botswana Harvard AIDS Institute Partnership, Gaborone, Botswana, ^20^Chiangrai Prachanukroh Hospital, Chiang Rai, Thailand, ^21^Research Institute for Health Sciences, Chiang Mai University, Chiang Mai, Thailand, ^22^Baylor College of Medicine Children's Foundation, Kampala, Uganda, ^23^Centre for Infectious Disease Research, Lusaka, Zambia

**Background: **Long‐acting (LA), intramuscular (IM) cabotegravir+rilpivirine is the first LA combination antiretroviral treatment (ART) regimen. IMPAACT 2017 evaluates the safety, acceptability, tolerability, and pharmacokinetics (PK) of this combination in virologically‐suppressed (HIV‐1 RNA <50 c/mL) adolescents. Data through Week‐48 are presented.

**Methods: **In this Phase I/II trial, virologically‐suppressed adolescents (12‐<18 years; ≥35 kg) with HIV‐1 switched from their pre‐study ART to at least 4 weeks of daily oral CAB+RPV followed by 600 mg CAB‐LA + 900 mg RPV‐LA IM (3‐mL each) in the contralateral gluteus medius at Week 4 and 8, and then every 8‐weeks.

**Results: **Eighteen centers in 5 countries enrolled 144 participants: median (range) age 15 years (12‐17), body mass index 19.5 kg/m^2^ (16.0‐34.3), weight 48 kg (35‐101), 51% female, 74% Black and 92% vertically acquired infection. Most participants received ≥1 injection (142/144), completed Week‐48 (140/144) and received the number of injections expected (140/144). Fifty‐six (39%) participants experienced a drug‐related adverse event (AE); three (2%) were ≥ Grade 3 AE (injection site [IS] pain and abscess [n = 1]; IS abscess [n = 1]; anaphylaxis leading to study drug discontinuation [n = 1]). Most common drug‐related non‐IS AEs were rash (n = 4), headache (n = 3) and nausea (n = 2). Fifty‐two participants (36%) experienced a drug‐related IS AE; mostly Grade 1 (92%) resolving within 7‐days (86%). No confirmed virologic failures occurred through Week‐48. Median (Q1‐Q3) Week‐48 observed pre‐dose concentrations for CAB (2.77 µg/mL[1.99‐3.55]) and RPV (67.9 ng/mL[52.8‐82.4]) approximated those in adults and were well above the respective protein‐adjusted IC_90_(Figure). While 44% and 93% of CAB‐LA and RPV‐LA recipients reported some pain during injection, all reported preferring LA injections to daily oral treatment at Week‐48 (140/140).

**Figure**. OAB2606LB

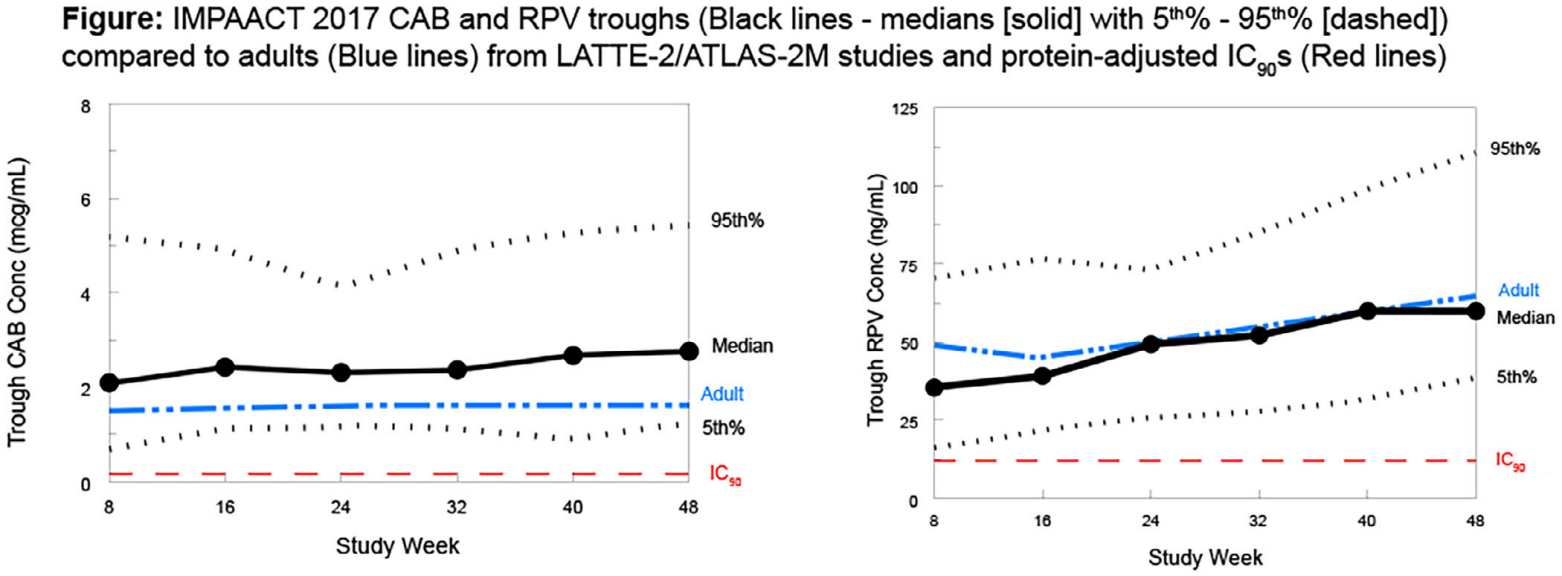

**Conclusions: **Week‐48 multinational data from the first, virologically‐suppressed adolescents living with HIV‐1 who switched from daily oral to injectable CAB‐LA + RPV‐LA every 8‐weeks demonstrate favorable safety/PK profile, strong participant preference and inform clinical use and regulatory submissions.

### OAB3406LB Abacavir is associated with elevated risk for cardiovascular events in the REPRIEVE trial

C.J. Fichtenbaum ^1^, C.D. Malvestutto^2^, M.G. Watanabe^3^, E.D. Smith^4^, H.J. Ribaudo^4^, S. McCallum^4^, K.V. Fitch^5^, J.S. Currier^6^, M. Diggs^5^, J.A. Aberg^7^, M.T. Lu^8^, J. Valencia^9^, C. Gomez‐Ayerbe^10^, I. Brar^11^, J.V. Madruga^12^, G.S. Bloomfield^13^, P.S. Douglas^14^, M.V. Zanni^5^, S.K. Grinspoon^5^, REPRIEVE Investigators

^1^University of Cincinnati, Internal Medicine‐Infectious Diseases, Cincinnati, United States, ^2^Ohio State University Medical Center, Columbus, United States, ^3^Center for Biostatistics in AIDS Research, Harvard T.H. Chan School of Public Health, Boston, United States, ^4^Harvard T.H. Chan School of Public Health, Center for Biostatistics in AIDS Research, Boston, United States, ^5^Harvard Medical School, Metabolism Unit, Massachusetts General Hospital, Boston, United States, ^6^David Geffen School of Medicine University of California Los Angeles, Los Angeles, United States, ^7^Icahn School of Medicine at Mount Sinai, Division of Infectious Diseases, New York City, United States, ^8^Harvard Medical School, Cardiovascular Imaging Research Center, Massachusetts General Hospital, Boston, United States, ^9^Asociacion Civil Impacta Salud y Educacion, Lima, Peru, ^10^Hospital Universitario Virgen de la Victoria, Malaga, Spain, ^11^Henry Ford Hospital, Detroit, United States, ^12^Centro de Referencia e Treinamento DST/AIDS, Sao Paulo, Brazil, ^13^Duke University School of Medicine, Duke Global Health, Durham, United States, ^14^Duke University School of Medicine, Duke Clinical Research Institute, Durham, United States

**Background: **Major adverse cardiovascular events (MACE) are more common in people with HIV (PWH). In REPRIEVE, pitavastatin reduced MACE by 35% among PWH with low‐to‐moderate traditional risk. We evaluated the role of prior and current use of antiretroviral agents (ART) on the development of MACE.

**Methods: **The trial enrolled PWH age 40–75 years on ART for at least 180 days, with a CD4 count >100 c/mm^3^ and low‐moderate CVD risk. ART history was collected at baseline, including duration of exposure to select agents. Analyses in the REPRIEVE ITT population were performed for first MACE (including MI, TIA/stroke, revascularization, CV death), with median follow‐up of 5.6 years. Cox proportional hazards models stratified by treatment group were used to account for treatment group differences. Effects of ART exposure were estimated in models unadjusted and adjusted for entry risk factors.

**Results: **Among 7,769 participants, 31.1% were natal female and 65.2% non‐White. Median age was 50 years, LDL 108 mg/dL, 10‐year ASCVD risk score 4.5%, CD4 621 cell/mm^3^ (447,826 c/mm^3^) with 88% having an HIV viral load <400 copies/mL. The median duration of ART use was 9.5 years (5.3,14.8 years) and varied by Country. Overall, 22% reported prior exposure to abacavir (ABC), 86% to Tenofovir (TDF), 49% to Thymidine analogs (AZT/d4T), and 47% to protease inhibitors (PIs). At study entry 13% were using ABC, 61% TDF, 10% AZT/d4T, and 26% PIs. Entry regimens included 2 NRTIs plus an NNRTI**–**47%, INSTI**–**25%, or a PI**–**19%. In adjusted analyses including the baseline regimen, both former and current use of ABC was associated with higher incidence of MACE (Figure). Former or current use of other ART agents was not associated with MACE (data not shown).

**Conclusions: **Former and current use of abacavir was associated with a higher incidence of subsequent major adverse cardiovascular events in the REPRIEVE trial.

**Figure**. OAB3406LB

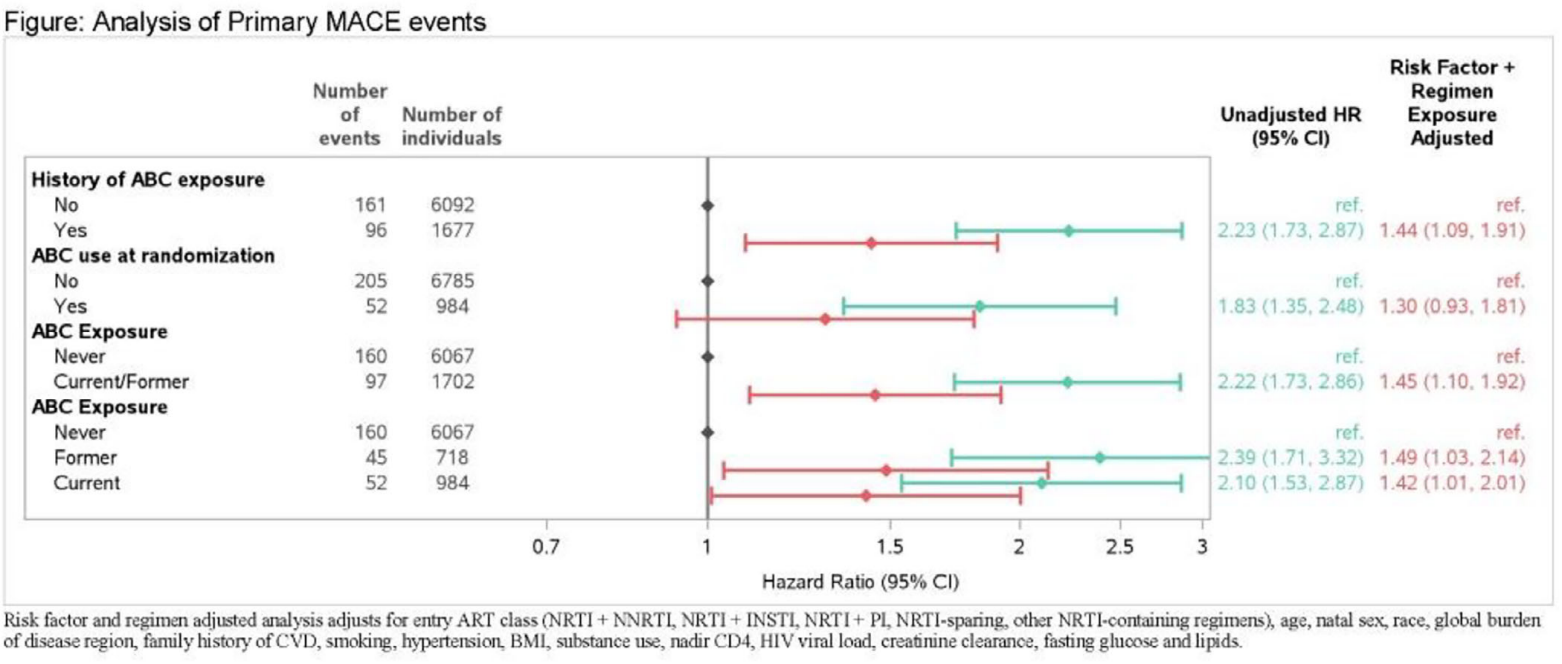

### OAB3606LB Non‐inferior efficacy and less weight gain when switching to DTG/3TC than when switching to BIC/FTC/TAF in virologically suppressed people with HIV (PWH): the PASO‐DOBLE (GeSIDA 11720) randomized clinical trial

P. Ryan^1^, J.L. Blanco^2^, M. Masia^3^, L. Garcia‐Fraile^4^, M.J. Crusells^5^, P. Domingo^6^, A. Curran^7^, R. Guerri‐Fernandez^8^, E. Bernal^9^, J. Bravo^10^, B. Revollo^11^, J. Macias^12^, J.M. Tiraboschi^13^, R. Montejano^14^, C. Amador^15^, M. Torralba^16^, D. Merino^17^, V. Diaz‐Brito^18^, M.J. Galindo^19^, S. Ferra^20^, A. Villoslada^21^, J.E. Losa^22^, F.J. Fanjul^23^, J. Perez‐Stachowski^24^, J. Peraire^25^, J. Portilla^26^, S. de la Fuente^27^, C. Dueñas^28^, M.J. Vazquez^29^, S. Di Gregorio^30^, E. Manzanares^31^, P. Gil^31^, M. de Miguel^31^, B. Alejos^32^, E. Martinez ^2^

^1^Hospital Universitario Infanta Leonor, Madrid, Spain, ^2^Hospital Clínic, Infectious Diseases, Barcelona, Spain, ^3^Hospital General Universitario, Elche, Spain, ^4^Hospital Universitario de la Princesa, Madrid, Spain, ^5^Hospital Clínico Universitario Lozano Blesa, Zaragoza, Spain, ^6^Hospital de la Santa Creu i Sant Pau, Barcelona, Spain, ^7^Hospital Universitari Vall d´Hebron, Barcelona, Spain, ^8^Hospital del Mar, Barcelona, Spain, ^9^Hospital Reina Sofía, Murcia, Spain, ^10^Hospital Morales Meseguer, Murcia, Spain, ^11^Hospital Universitari Germans Trias i Pujol, Badalona, Spain, ^12^Hospital Universitario Virgen de Valme, Sevilla, Spain, ^13^Hospital Universitario de Bellvitge, L'Hospitalet de Llobregat, Spain, ^14^Hospital Universitario La Paz, Madrid, Spain, ^15^Hospital Marina Baixa, Villajoyosa, Spain, ^16^Hospital Universitario, Guadalajara, Spain, ^17^Hospital Juan Ramon Jimenez, Huelva, Spain, ^18^Parc Sanitari Sant Joan de Deu, Sant Boi de Llobregat, Spain, ^19^Hospital Clínico Universitario, Valencia, Spain, ^20^Hospital Universitario Torrecárdenas, Almeria, Spain, ^21^Hospital Universitario Son Llatzer, Palma de Mallorca, Spain, ^22^Hospital Universitario Fundacion Alcorcón, Alcorcon, Spain, ^23^Hospital Universitario Son Espases, Palma de Mallorca, Spain, ^24^Hospital Costa del Sol, Marbella, Spain, ^25^Hospital Universitari Joan XXIII, Tarragona, Spain, ^26^Hospital General Universitario Dr. Balmis, Alicante, Spain, ^27^Hospital Universitario Puerta de Hierro‐Majadahonda, Majadahonda, Spain, ^28^Hospital Clínico Universitario, Valladolid, Spain, ^29^ViiV Healthcare, Tres Cantos, Spain, ^30^CP Endocrinologia i Nutrició S.L., Barcelona, Spain, ^31^Fundación SEIMC‐GeSIDA, Madrid, Spain, ^32^Independent researcher, Madrid, Spain

**Background: **DTG/3TC and BIC/FTC/TAF are preferred regimens in major guidelines, but there are no fully powered trials comparing between them.

**Methods: **PASO‐DOBLE (ClinicalTrials.gov NCT04884139) is a randomized, open‐label trial conducted at 30 sites throughout Spain. Virologically suppressed PWH on regimens containing ≥1 pill/day, boosters, or drugs with cummulative toxicity such as efavirenz or TDF were eligible. Participants were randomized (1:1) to switch stratifying by TAF in the regimen discontinued and sex. Primary endpoint was the proportion of PWH with RNA ≥50 copies/mL at 48 weeks (FDA snapshot, 4% non‐inferiority margin) in the exposed intention‐to‐treat population. Weight changes were also evaluated.

**Results: **Between 14‐July‐2021 and 24‐March‐2023, 553 PWH initiated DTG/3TC (n = 277) or BIC/FTC/TAF (n = 276), including 155 (28%) with TAF in the regimen discontinued and 147 (27%) women. At 48 weeks, DTG/3TC was non‐inferior to BIC/FTC/TAF [risk difference between DTG/3TC (2.2%) minus BIC/FTC/TAF (0.7%) 1.4%, 95%CI ‐0.5 to 3.4] (Figure A). HIV RNA levels were low (≤282 copies/mL) in those showing detectable viral load. Mean adjusted weight increased significantly more with BIC/FTC/TAF (1.81kg, 95%CI 1.28‐2.34) than with DTG/3TC (0.89kg, 95%CI 0.37‐1.41) [difference 0.92kg, 95%CI 0.17‐1.66]. The proportion of participants with weight gain >5% at 48 weeks was 29.9% for BIC/FTC/TAF vs. 20% for DTG/3TC (adjusted OR 1.81, 95%CI 1.19‐2.76). While proportions of PWH experiencing >5% weight gain with DTG/3TC were similar irrespective of the nucleos(t)ide reverse transcriptase inhibitor (NRTI) backbone discontinued, proportions of PWH experiencing >5% weight gain with BIC/FTC/TAF were 50% or 100% higher than those with DTG/3TC when switching from abacavir or TDF (Figure B). Weight change in women (OR 1.131, 95% CI: 0.700‐1.826) didn't differ from that in men. There were few discontinuations (DTG/3TC = 1, 0.4%; BIC/FTC/TAF = 2, 0.7%) due to adverse events.

**Figure**. OAB3606LB

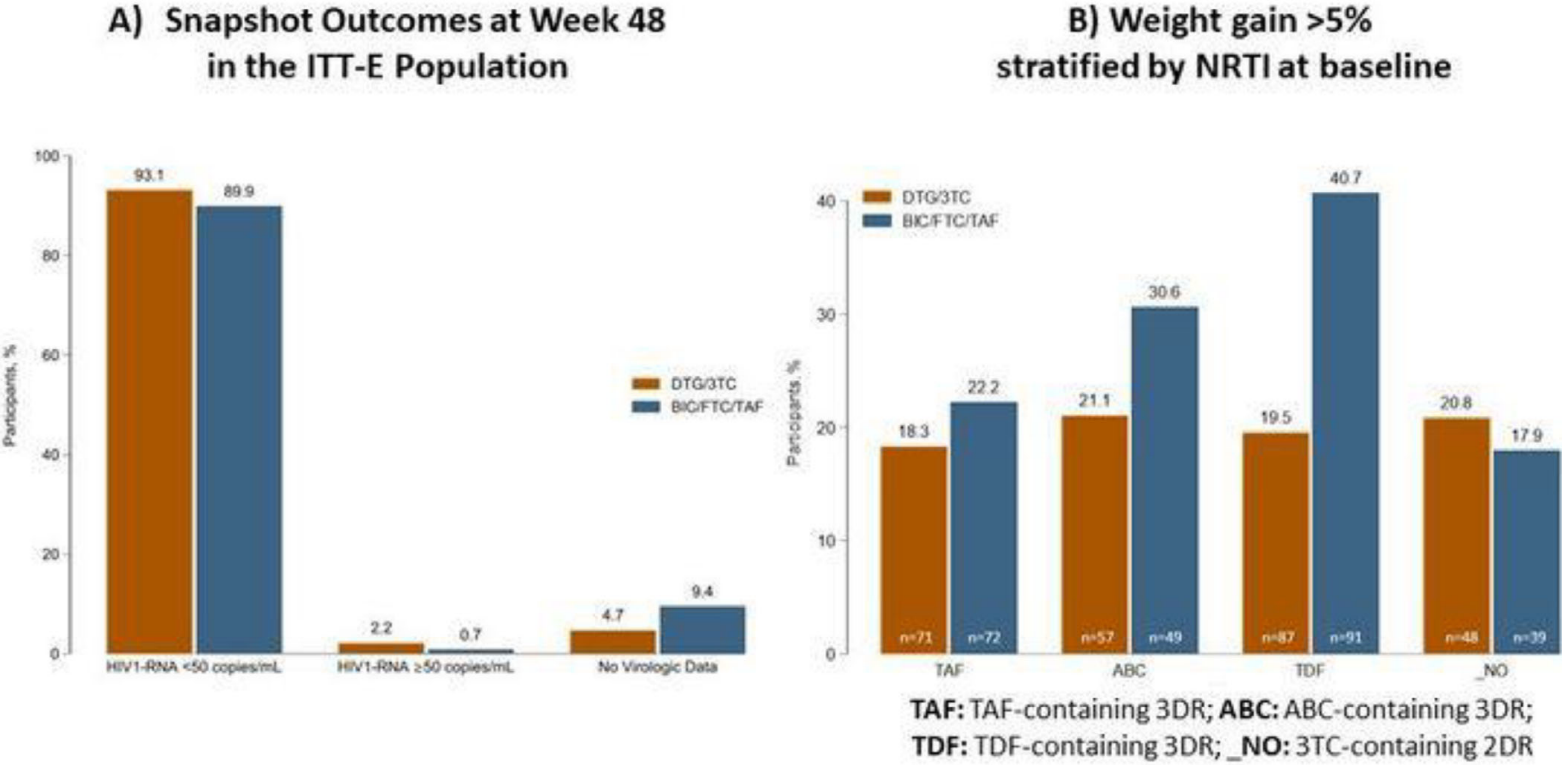

**Conclusions: **Switching to DTG/3TC demonstrated non‐inferior efficacy and resulted in less weight gain than switching to BIC/FTC/TAF at 48 weeks.

### OAB3806LB Dolutegravir with recycled Nucleoside Reverse Transcriptase Inhibitors maintains better viral suppression than Protease Inhibitor based Antiretroviral therapy over 144 weeks: VISEND Trial

S. Sivile ^1,2,3^, S. Fwoloshi^1,2,3^, D. Engamba^1,4^, A. Mweemba^4,1^, M. Siwingwa^1,3^, D. Kampamba^1,2^, M. Mbewe^1^, A.P. Kumar^1^, N. Mbewe^1,5^, H. Phiri^2^, A. Shibemba^1^, B.P. Haabanji^1^, E. Mwamba^1^, B. Simons^6^, C.W. Wester^7,8^, L. Chirwa^1^, A. Hill^9^, L. Mulenga^1,3,2,7,8^

^1^University Teaching Hospital, Adult Infectious Diseases Center, Lusaka, Zambia, ^2^Ministry of Health, Division of Infectious Diseases, Lusaka, Zambia, ^3^University of Zambia, School of Medicine, Division of Infectious Diseases, Lusaka, Zambia, ^4^Levy Mwanawasa Medical University, Division of Infectious Diseases, Lusaka, Zambia, ^5^Zambia National Public Health Institute, Lusaka, Zambia, ^6^London School of Economics & Political Science, London, Zambia, ^7^Vanderbilt Institute for Global Health, Nashville, United States, ^8^Vanderbilt University Medical Center, Department of Medicine, Division of Infectious Diseases, Nashville, United States, ^9^Liverpool University, Department of Translational Medicine, Liverpool, United Kingdom

**Background: **Dolutegravir (DTG) is recommended for both first and second line antiretroviral therapy (ART). Despite emerging evidence on positive clinical outcomes among individuals failing NNRTI‐based ART and switched to DTG‐based regimens with maintained NRTIs, there is paucity in data on longer‐term outcomes. We hereby report the 144‐week outcomes of the VISEND trial.

**Methods: **We conducted a 144 week, randomized, open‐label, phase 3 non‐inferiority trial in Zambia. We randomized adults with viral load (VL) < 1000 copies/mL on tenofovir disoproxil fumarate (TDF), lamivudine (3TC) plus efavirenz (EFV) or nevirapine (NVP) to TDF,3TC,DTG (**TLD**) or tenofovir alafenamide fumarate (TAF), emtricitabine (FTC), DTG (**TAFED**) [Arm A]. Participants with VL < 1000 copies/mL were randomized to **TLD** or **TAFED** or standard‐of‐care (SOC) second line comprising Lopinavir/ritonavir (LPV/r) or atazanavir/r (ATV/r), zidovudine (ZDV), 3TC [Arm B]. Primary end point was VL <1,000 copies /mL (viral suppression (VS)), assessed using the FDA snapshot algorithm (intent‐to‐treat (ITT) population). Noninferiority was tested with a margin of 10 percentage points.

**Results: **Of 1,201 participants, 99% in Arm A maintained viral suppression on both TLD and TAFED [difference, ‐0.0%, 95% CI ‐0.02‐ 0.02] at week 144. In Arm B, 98% TLD‐treated adults achieved VS, compared to 96% for TAFED and 89% for 3TC/ZDV/PI/r. Noninferiority of switching to both DTG‐based arms was achieved compared to the SOC (TLD versus ZDV/3TC/PI/r difference, 9.9% [5.9 ‐ 13.9]; TAFED versus ZDV/3TC/PI/r difference, 8.8% [4.7 – 12.9]). The baseline prevalence of tenofovir and lamivudine/emtricitabine associated resistant mutations was 56% (204/363) and 75%, (273/363) respectively. In those randomised to TDF/3TC/DTG and TAF/FTC/DTG, 61% and 52% had no predicted tenofovir activity respectively; whereas 75% had no predicted lamivudine or emtricitabine activity in both groups. There was no major resistant mutations to DTG but 26 to bPI and 32 to NRTIs among those with virologic failure over the study period.

**Conclusions: **In the VISEND trial, HIV‐positive adults with virologic failure to TDF/3TC/NNRTI, had favourable outcomes when switched to DTG with recycled NRTIs compared to those switched to SOC boosted‐PI despite high baseline resistance to NRTIs. No emergent INSTI mutations were reported at week 144. We recommend recycling of TDF(TAF)/3TC with DTG following failure.

### OAC1006LB Explaining the transmission dynamics of mpox in Europe and the Americas between 2022–2024: findings from an online survey in 23 countries

M. Prochazka ^1^, P. Vinti^2^, A. Hoxha^1^, A. Seale^1^, A. Mozalevskis^1^, R. Lewis^1^, R. Mayorga Sagastume^3^, M. Scherzer^2^, L. Dore^1^, M. Doherty^1^

^1^World Health Organization, Geneva, Switzerland, ^2^WHO Regional Office for Europe, Copenhagen, Denmark, ^3^Pan American Health Organization, Washington DC, United States

**Background: **After rapid epidemic growth between May‐August 2022, new mpox diagnoses declined in Europe and the Americas, with low‐level transmission continuing thereafter. Characterising the extent of behavioural adaptation, mpox vaccination, and mpox prevalence across these regions during the first year of the outbreak can support understanding transmission dynamics.

**Methods: **WHO conducted a retrospective online survey in 23 countries in Europe and the Americas between 19–31 May 2023. The survey was advertised via four geospatial dating applications used by gay, bisexual and other men who have sex with men, and trans and gender diverse people. We described and regionally compared the mpox prevalence, mpox vaccination rates (2 doses) and the extent and duration of behavioural adaptation. We estimated crude and adjusted prevalence ratios (PRs) with confidence intervals (CI) for behavioural outcomes using generalised linear models.

**Results: **Of 16,875 participants, 6.4% (1,086) reported having mpox during the outbreak. Vaccination with at least one vaccine dose was reported by 29.6% (4,987/16,875) of participants; 20.8% (3,502/16,875) reported two doses. Complete vaccination in Latin America (3.5%) and in Eastern Europe and Western Balkans (1.6%) was significantly lower than in Western Europe (27.7%) and North America (51.3%, p<0.001). Adaptations to sexual behaviour were reported by 50.9% (8,583/16,875) and across all regions. Among those who made adaptations, 35.5% (3,045/8,583) said they continued adapting their sexual behaviour up to May 2023. In regression models, participants who reported concerns about mpox (58.6%) were more likely to adapt their behaviour (aPR95%CI: 2.43 [2.34‐2.53]), whereas participants who reported vaccination (aPR95%CI: 0.25 [0.28‐0.31] or having had mpox (aPR95%CI: 0.37 [0.30‐0.44]) were less likely to continue adaptations. Participants in Latin America or North America were significantly more likely to adapt their sexual behaviour and to continue with adaptations compared to participants in Western Europe (p<0.001).

**Conclusions: **Adaptations to sexual behaviour due to mpox were widespread and dynamic, and responded to evolving individual risk perceptions. Given stark vaccine inequity during the first year of the global response, but comparable reduction in transmission, we propose that the sudden decline in mpox transmission seen at the end of 2022 occurred as a combination of community‐led behavioural adaptation and naturally‐acquired immunity.

### OAC1506LB Risk factor assessment for HIV, HBV, and HCV in migrants from Central and South America

R. Lara‐Medrano ^1^, V. Baylon‐Valdez^1^, L.G. Castillo‐Reyna^2^, G.M. Aguirre‐García^1^, M.T. Ramírez‐Elizondo^1^, D. Ramonfaur‐Gracia^1^, M.F. Martínez‐Reséndez^1^, F.J. Bosques‐Padilla^1^

^1^School of Medicine and Health Sciences, Instituto Tecnológico y de Estudios Superiores de Monterrey, Monterrey, Mexico, ^2^Consejo Estatal para la Prevención y el Control del SIDA. Secretaría de Salud, Monterrey, Mexico

**Background: **The unprecedented increase of migratory flow in the Americas region, with individuals seeking to reach the United States of America via Mexico, represents a significant challenge for local health authorities. These key populations are exposed to numerous risks, such as sexual violence and drug use, which pose a risk of exposure to HIV and viral Hepatitis. This study aims to assess the risk factors involved in the incidence of these infections among migrants.

**Methods: **Community‐based screening programs were conducted in shelters, refugee centers, and migrant houses in the state of Nuevo Leon, Mexico. Between December 2023 and April 2024, adults from Central and South America, transiting through Nuevo Leon to the United States, were invited to participate. A sociodemographic questionnaire was administered, and screening was performed using rapid tests for HIV, hepatitis B and C viruses, and syphilis.

**Results: **A total of 117 migrants participated, with 59% identifying as males and a mean age of 33 ± 9 years. Predominantly from Honduras (46%), Venezuela (13%), and Guatemala (11%), primary motivations for migration included fleeing violence (46%) and seeking employment (35%), with a mean transit duration of 9 months. Eighty‐seven percent reported engaging in heterosexual intercourse, yet only 38% used contraceptive methods, with condoms being the most common (62%). Drug use was documented in 16% of participants, mainly marijuana (84%). Use of drugs during sexual intercourse was reported in 12%, with marijuana being the most common. Sale of sexual services occurred in 10%, with only 25% of these using condoms. Sexual abuse was reported by 12% of participants. Screening acceptance rates for HIV and syphilis tests were 86%, for Hepatitis B 93%, and for Hepatitis C 85%. Three participants had a positive rapid HIV test, confirmed with HIV viral load; one of them had a positive rapid syphilis test. All were referred for treatment initiation and follow‐up.

**Conclusions: **These findings underscore the critical importance of tailored interventions for HIV prevention, treatment, and care within migrant populations. Highlighting the urgent need for comprehensive public health strategies addressing the intersecting vulnerabilities faced by migrants, including access to sexual health education and interventions for people who use drugs.

### OAC2206LB Early HIV Infection Diagnostic Challenges in Injectable Long‐Acting Cabotegravir Implementation in Routine Public Health PrEP Service in Zambia

L. Mulenga ^1,2,3,4^, C. Phiri^1,2^, T. Chisenga^2^, K. Zyambo^1,2^, S. Sivile^1,3,2^, I. Bwalya^5^, K. Musokotwane^5^, M. Mwansa^5^, M. Siame^2^, S. Fwoloshi^1,3,2^, J. Mzyece^2^, M. Mwitumwa^1,3^, L. Kampilimba‐Mwango^6^, A. Ndhlovu^7^, L. Kawanga^7^, D. Kampamba^1,2^, B. Tambatamba^2^, D. Nsama^2^, M. Musonda^8^, K. Lishimpi^2^, G. Sinyangwe^2^, L. Hachaambwa^1,6,9^, M. Siwingwa^1,3^, J. Mutukwa^2^, L. Chitembo^10^, C. Claassen^1,6,9^

^1^University Teaching Hospital, Adult Infectious Diseases Center, Lusaka, Zambia, ^2^Ministry of Health, Division of Infectious Diseases, Lusaka, Zambia, ^3^University of Zambia, School of Medicine, Division of Infectious Diseases, Lusaka, Zambia, ^4^Vanderbilt University Medical Center, Department of Medicine, Division of Infectious Diseases, Nashville Za, Zambia, ^5^National AIDS Council, Lusaka, Zambia, ^6^Ciheb Zambia, Lusaka, Zambia, ^7^JSI DISCOVER Health, Lusaka, Zambia, ^8^U.S. Agency for International Development, Lusaka, Zambia, ^9^University of Maryland School of Medicine, Division of Infectious Diseases, Baltimore, United States, ^10^World Health Organisation, Lusaka, Zambia

**Background: **To further curb new HIV infections, injectable long‐acting cabotegravir (CAB‐LA) pre‐exposure prophylaxis (PrEP) was introduced routinely in Zambia in February 2024, the second country after the USA to roll out CAB‐LA PrEP outside research settings. It is critical to understand risks of resistance and optimal HIV testing strategies in such real‐world settings. We describe early experience and challenges in identifying acute HIV infection (AHI) in a low‐resource implementation environment.

**Description: **Using clinical screening tools, at‐risk priority and key populations are identified and screened for CAB‐LA PrEP eligibility. Screening includes assessment for AHI, HIV testing using third‐generation rapid diagnostic antibody test (RDT), and HIV nucleic acid amplification test (NAAT), though it has a two‐week turn‐around time. If HIV RDT and AHI screening is negative, CAB‐LA initiation injection 1 is administered; injection 2 is given one month later after confirming continuation eligibility. NAAT is used to confirm eligibility upon receipt of results.

**Lessons learned: **From 9 February to 30 April 2024, we screened 927 individuals for substantial HIV acquisition risk. 853 screened RDT/AHI negative and were initiated on CAB‐LA PrEP. Among them, 4/853 (0.5%) individuals (3 men, 1 woman, ages 21–28) tested NAAT positive. Three of the four (75%) had received dose 1; one (25%), had received both dose 1 and 2 by the time NAAT results were received. HIV‐1 RNA levels among the four NAAT+ persons were <30, <30, 141, and 533,000 copies/mL. Resistance testing on the HIV‐1 RNA >1,000 copies/mL sample showed sensitivity to integrase strand transfer inhibitors. Furthermore, two discordant results (RDT negative and NAAT+) were recorded prior to CAB‐LA initiation. There was better consistency in results with NAAT when a different RDT was used.

**Conclusions/Next steps: **Early experience in CAB‐LA PrEP revealed challenges in identifying AHI in resource‐limited settings using WHO‐recommended RDTs for HIV screening.Two different serological assays showed better concordance to NAAT. Vigilant monitoring and stringent testing protocols are critical for accurate AHI identification and program optimization. We recommend two different serology tests and/or virologic testing to detect AHI among persons initiating CAB‐LA PrEP to prevent resistance. Point of care PCR platforms could reduce NAAT turnaround time and improve early AHI diagnosis.

### OAC2906LB The Impact of Russia's War in Ukraine on Opioid Agonist Treatment (OAT) Services, a Primary HIV Prevention Strategy among People Who Inject Drugs (PWID)

R. Ivasiy ^1^, L.M. Madden^2^, A. Meteliuk^3^, T. Fomenko^3^, E. Machavariani^1^, D.J. Bromberg^4^, M. Filippovych^5^, I. Kharandiuk^5^, S.O. Farnum^2^, Z. Islam^3^, F.L. Altice^1^

^1^Yale University ‐ School of Medicine, AIDS Department, New Haven, United States, ^2^APT Foundation, New Haven, United States, ^3^ICF “Alliance for Public Health”, Kyiv, Ukraine, ^4^Yale University ‐ School of Public Health, AIDS Department, New Haven, United States, ^5^Ukrainian Institute of Public Health Policy, Kyiv, Ukraine

**Background: **Russia's full‐scale war in Ukraine since 24‐Feb‐2022 has greatly disrupted OAT services for PWIDs, especially in high HIV burden southeastern regions, leading to internal displacement. Our study seeks to assess how the war and internal relocation affect OAT retention, critical for HIV prevention among PWIDs.

**Methods: **We conducted a two‐year comparative cohort survival analysis using Ukraine's national OAT registry from 252 clinics across 25 regions. The study compared local OAT patients in conflict‐remote regions with conflict‐affected regions and internally displaced patients. Regions were clustered into frontline, target, and remote based on conflict‐related metrics: numbers of air raids, explosions, artillery attacks, and percentages of internally displaced population. The primary outcome, treatment retention, was assessed considering time‐dependent displacement status, medication type (methadone vs. buprenorphine), OAT dose (optimal (methadone: ≥90mg; buprenorphine: ≥16mg) vs. suboptimal), dispensation strategy (daily vs. THD: take‐home dosing), age, sex, inject drug duration, HIV‐status.

**Results: **The nationwide sample of PWID receiving OAT as of 23‐Feb‐2022 comprised 17,265 individuals, with 4,953 (28.7%) in frontline, 8,786 (50.9%) in target, and 3,526 (20.4%) in remote regions. Most were male (84.6%), averaging 38.9±7.5 years old. From 24‐Feb‐2022 through 01‐Jan‐2024, 519 (0.03%) individuals underwent internal relocation, and 5,040 (29.2%) experienced treatment dropout. Compared to the local patient cohort in remote regions, patients local to target and frontline regions had elevated risk of treatment discontinuation (adjusted hazard ratios (aHR):1.31, 95%CI:1.27‐1.35; aHR:6.92, 95%CI:6.87‐6.97, respectively). Internally displaced patients in target, frontline, and remote regions faced significantly greater dropout risk than local patients in conflict‐distant regions (aHR:5.95, 95%CI:5.80‐6.03; aHR:6.80, 95%CI:6.73‐6.87; aHR:16.79, 95%CI:16.61‐16.95, respectively). The dropout risk for internally displaced patients in remote areas mirrored that of patients in frontline regions. Predictors of higher treatment retention included optimal dosing (aHR:0.79, 95%CI:0.76‐0.82), THD (aHR:0.72, 95%CI:0.68‐0.76), and receiving methadone (aHR:0.88, 95%CI:0.84‐0.92). Females and people with HIV had a heightened risk of treatment discontinuation (aHR:1.17, 95%CI:1.13‐1.21; aHR:1.07, 95%CI:1.04‐1.11).

**Conclusions: **Russia's full‐scale war in Ukraine increased the risk of OAT discontinuation for patients in conflict‐affected regions and all internally displaced patients, undermining HIV prevention efforts among PWIDs. Further research on national OAT service preparedness for disasters is crucial for uninterrupted care for key populations.

### OAC3306LB Tracking progress in HIV control among male prison inmates in North India: findings from HIV Sentinel Surveillance 2019–2023

L. PVM ^1^, A.K. Sethi^1^, P. Kumar^2^, C. Das^2^, S. Biswas^2^

^1^Postgraduate Institute of Medical Education and Research, Department of Community Medicine and School of Public Health, Chandigarh, India, ^2^National AIDS Control Organization, New Delhi, India

**Background: **India's National AIDS and STD Control Programme targets 80% reduction in new infections from 2010 to 2026. Further, 95% of people living with HIV (PLHIV) should know their status, and 95% of those who know their status should receive treatment. Prison inmates are at substantial risk of HIV infection. We therefore aimed to track progress in HIV control from 2019 to 2023 among prison inmates in North India.

**Methods: **We did a secondary analysis of data for male prison inmates in seven Central Prisons across six States / Union Territories in North India. Data were collected during the 2019, 2021 and 2023 rounds of HIV Sentinel Surveillance. We examined the trends across surveillance rounds in HIV seroprevalence, knowledge of HIV status among PLHIV, and uptake of antiretroviral therapy by PLHIV who knew their status, using mixed‐effects logistic models accounting for prison‐level clustering. Additionally, we fitted models adjusting for socio‐demographics, imprisonment characteristics, knowledge of HIV, behavioural risks and HIV testing.

**Results: **We included 8,400 prison inmates, i.e. 2,800 in each round. From 2019 to 2023, HIV seroprevalence increased from 3.8% to 5.7%. There were declines in awareness of HIV status among PLHIV (65.4% in 2019 to 28.1% in 2023) and uptake of antiretroviral therapy among PLHIV who knew their status (94.3% in 2019 to 84.4% in 2023). The trend in HIV seroprevalence was partly explained by injecting drug use, but persisted despite adjustment. The trend in knowledge of HIV status was explained by HIV testing and knowledge of HIV.

**Figure**. OAC3306LB

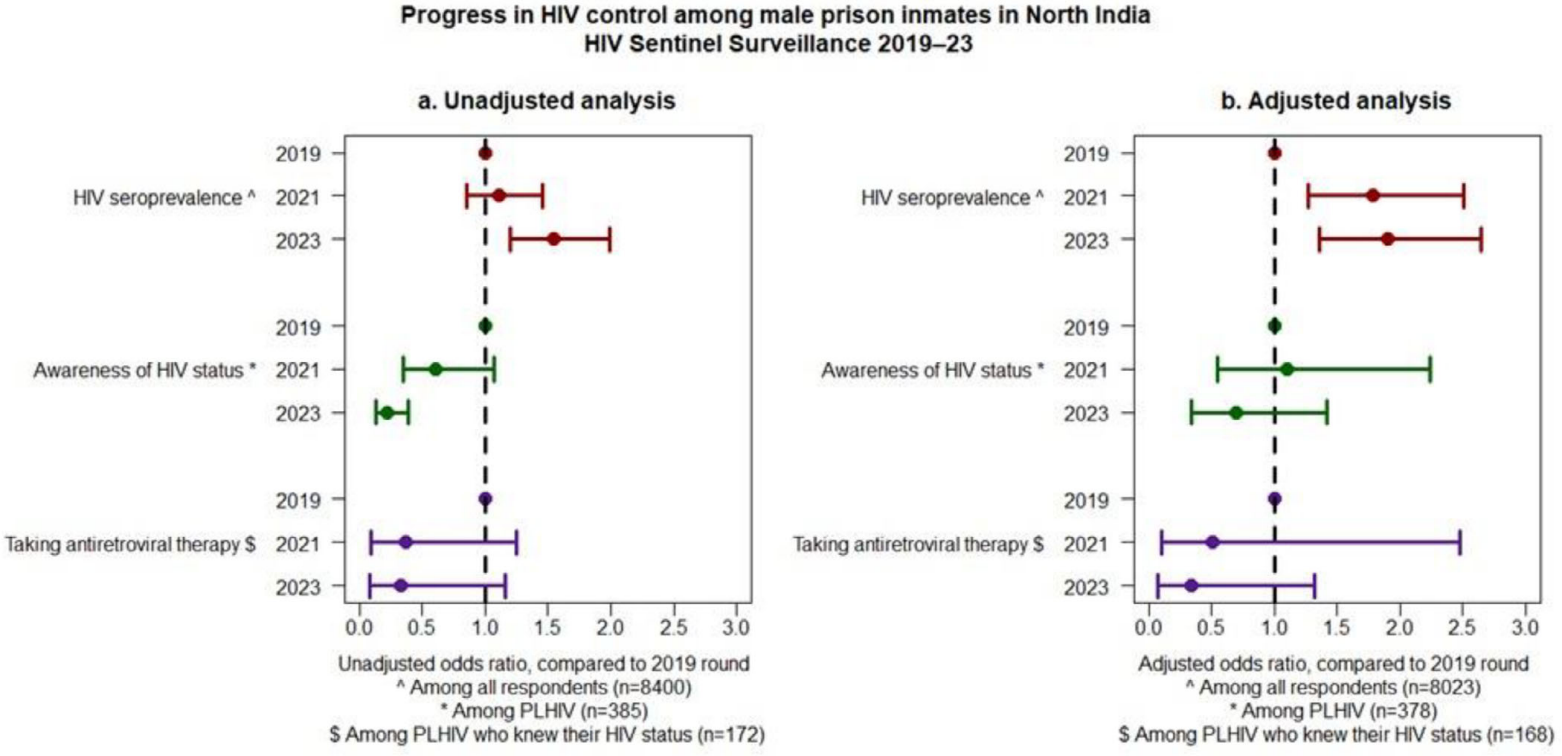

**Conclusions: **Injecting drug use is an important contributor to HIV infection among prison inmates in North India. Further, HIV testing and treatment coverage remain low in this population. Innovations in programme implementation are needed to enhance behaviour change, HIV testing and antiretroviral therapy coverage in prison settings.

### OAD0706LB “*The simplest way to go*:” A mixed methods analysis of why women who inject drugs selected long‐acting injectable cabotegravir instead of daily oral PrEP

A.M. Roth ^1^, A.K. Groves^1^, K.R. Amico^2^, E.C. McDowell^1^, K.M. Ward^1^, E. Forman^1^, S. Palmer^1^, A. Crayton^1^, D.S. Krakower^3^, A. Carrico^4^, K. Mims^5^, T.S. Bartholomew^6^

^1^Drexel University, Department of Community Health & Prevention, Philadelphia, United States, ^2^University of Michigan, Department of Health Behavior and Health Education, Ann Arbor, United States, ^3^Harvard Medical School, Department of Population Medicine, Boston, United States, ^4^Florida International University, Health Promotion and Disease Prevention, Miami, United States, ^5^Prevention Point Philadelphia, Philadelphia, United States, ^6^University of Miami Miller School of Medicine, Department of Public Health Sciences, Miami, United States

**Background: **Women who inject drugs (WWID) have disproportionately high rates of HIV, and thus would benefit from HIV pre‐exposure prophylaxis (PrEP). WWID who struggle with daily adherence may prefer long‐acting injectable cabotegravir (CAB‐LA) however, few studies have assessed product preference, and none have described the process of product selection among WWID who were offered both modalities.

**Methods: **Quantitative data are from 82 English‐speaking, cisgender WWID ≥18 years, who received a PrEP prescription (oral PrEP or CAB‐LA) from a provider integrated within a syringe services program (SSP) in Philadelphia (USA). Qualitative data are from a subset who completed a semi‐structured interview (n = 18 who chose CAB‐LA, n = 7 who chose oral PrEP). All participants are part of an ongoing RCT designed to reduce HIV acquisition risk. We used thematic analysis to explore the process of product selection.

**Results: **Most WWID selected CAB‐LA (75/82). Higher frequency of injection drug use and higher average number of sexual partners were each associated with selecting CAB‐LA (93% daily vs 70% less than daily, *p = 0.056*; 1 vs 0, *p<0.01*, respectively). No matter which product WWID selected, the decision‐making process was similar. Product selection, as described in participant interviews, was informed by their perceived risk for HIV exposure, if/how adherence might be impacted by their individual context (e.g., addiction severity), the brief individual counseling session they received prior to making their selection, and prior experience with oral and/or injectable medications. For WWID selecting CAB‐LA, reducing day‐to‐day action to receive long‐term prevention benefits was important, as few believed they would adhere daily. Those selecting oral PrEP expressed more medical mistrust and dislike of needles and injectable medications. All women felt supported in their product selection by their PrEP providers.

**Conclusions: **Nearly all WWID in our study selected CAB‐LA, suggesting a strong preference for this modality in this group. Women, including those selecting oral PrEP, had strong rationales for their choice. Offering women a wider array of products to choose from will likely increase uptake, as it has for other sexual health tools, like contraceptives.

### OAD1606LB Key strategic actions to improve uptake of and create demand for pre‐exposure prophylaxis (PrEP) among key populations (KPs): qualitative evidence from Cambodia

K. Seang^1^, S. Ky^2^, V. Ouk^2^, S. Samreth^2^, B. Ngauv^2^, P. Ung^3^, V. Saphonn ^4^

^1^University of Health Sciences, Grant Management Office, Phnom Penh, Cambodia, ^2^National Center for HIV/AIDS, Dermatology and STD, Phnom Penh, Cambodia, ^3^UNAIDS, Phnom Penh, Cambodia, ^4^University of Health Sciences, Rectorate, Phnom Penh, Cambodia

**Background: **The majority of new HIV infections were among KPs but the uptake and retention rates of PrEP among MSM, TGW and female entertainment workers (FEW) in Cambodia were ​limited (about half of those previously enrolled in PrEP program discontinued PrEP). Additional evidence and support are required to better understand their suboptimal demand, uptake and retention of PrEP in order to come up with effective improvement strategies.

**Methods: **A qualitative study was conducted among policy‐level stakeholders (n = 9), potential PrEP clients (n = 70) and PrEP providers (n = 26) using key informant interview for the first group and focus group discussions as well as in‐depth interviews for the last two. The data collection took place in six selected PrEP sites across three provinces and Phnom Penh capital. The analysis was based on Braun & Clarke's thematic analysis methodology highlighting contextual, political, sociodemographic and behavioral factors that impact PrEP demand and use.

**Results: **Side effects, stigma/discrimination, misinformation, ineffective risk communication strategies and low HIV risk perception were commonly reported barriers to PrEP demand and uptake. Raising PrEP credibility and accessibility, encouraging involvements from KP community, and other public and private sectors and improving messages surrounding PrEP and PrEP users, should be considered to improve this suboptimal demand and uptake.

**Table:** Table. OAD1606LB

Socio‐demographics	MSM (*n* = 25)	TGW (*n* = 21)	FEW (*n* = 24)
Age (median, IQR)	(26, 9)	(24, 8)	(24, 11)
Education			
None to some or completed primary	1	7	16
Some secondary to ≥ BA (Bachelor) degrees	24	14	8
PrEP use			
Current users (on‐demand or daily)	13	10	11
Non‐users (including never users)	12	11	13

FEW, biological females, exchanged vaginal/oral/anal sex for money, goods, or gifts in past 12 months

IQR, Interquartile range

PrEP non‐users, those who never use PrEP or have stopped using PrEP ≥3 months

**Figure**. OAD1606LB

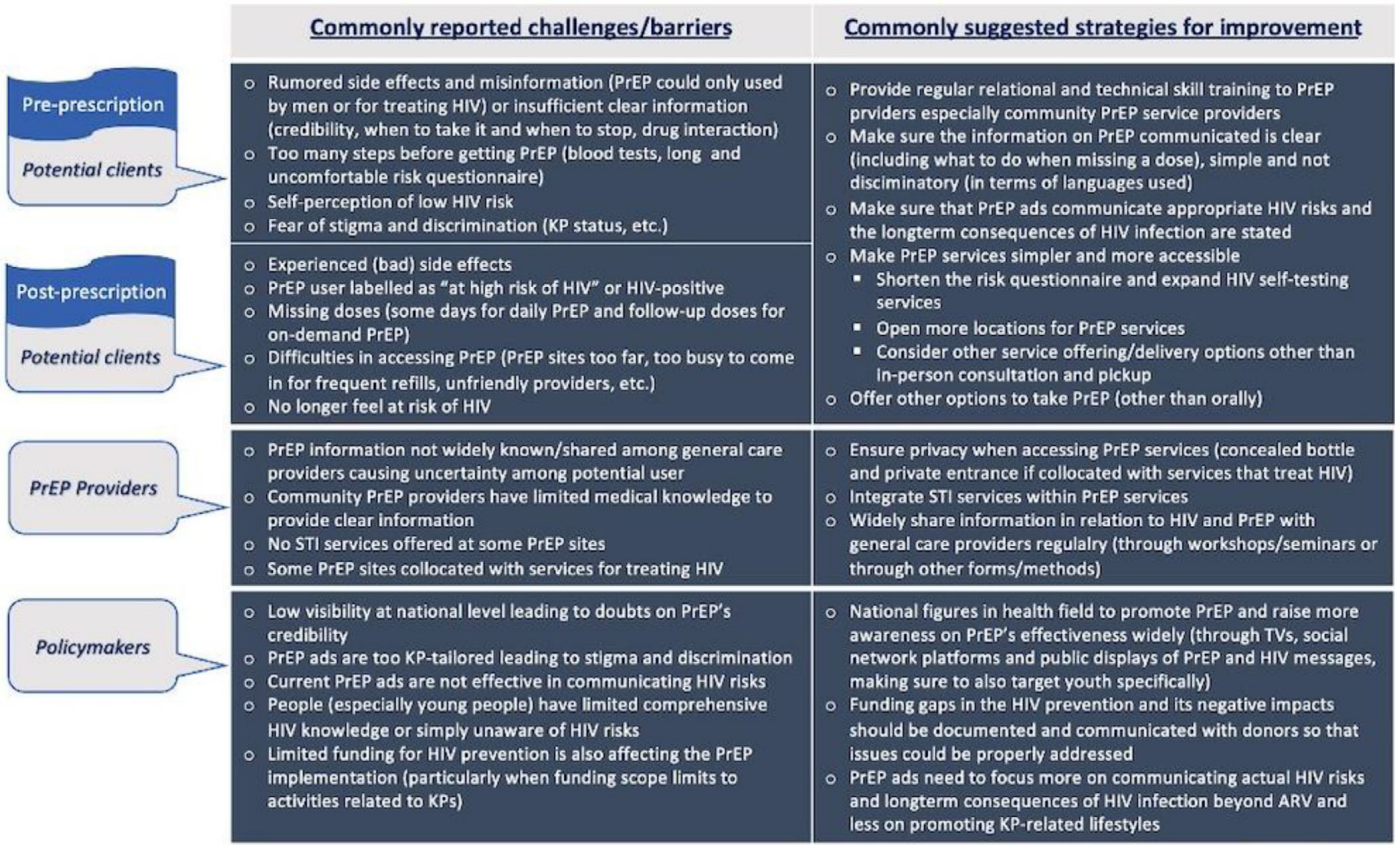

**Conclusions: **KP‐tailored messages to promote PrEP might be more cost‐efficient but could risk inducing stigma and discrimination against this population. Prevention efforts related to PrEP should focus more on effectively communicating HIV risks with correct (factual) information and simple wording rather than on KP populations due to their explicitly stated sexual‐orientation‐and‐gender‐identity‐related HIV risks.

### OAD3206LB Unveiling the hidden truth: HIV‐related stigma and discrimination among European healthcare workers

T. Noori ^1^, A. Sullivan^2^, F. Burns^2^, J. Del Amo^3^, C. Deogan^1^, K. Darling^4^, E. Vaughan^5^, D. Simoes^6^, A. Garner^7^, S. Pasanen^8^, J. Verluyten^9^, E. Martinez^10^, A. Méndez López^11^

^1^European Centre for Disease Prevention and Control (ECDC), Solna, Sweden, ^2^NHS Foundation Trust, London, United Kingdom, ^3^Ministry of Health, Madrid, Spain, ^4^CHUV, Lausanne, Switzerland, ^5^University of Galway, Galway, Ireland, ^6^Grupo de Ativistas em Tratamentos, Lisbon, Portugal, ^7^MPACT Global, Los Angeles, United States, ^8^Positiiviset ry, HivFinland, Helsinki, Finland, ^9^European AIDS Clinical Society, Brussels, Spain, ^10^Hospital Clínic & University of Barcelona, Barcelona, Spain, ^11^Universidad Autónoma de Madrid, Madrid, Spain

**Background: **HIV‐related stigma in healthcare settings is recognised as a barrier to achieving positive health and well‐being and is linked to negative healthcare outcomes. Data on HIV stigma in the healthcare setting has been lacking, and as a result the European Centre for Disease Prevention and Control and the European AIDS Clinical Society partnered to conduct the first ever European‐wide HIV stigma survey.

**Methods: **A survey, translated into 38 languages, was developed and conducted from September 15 to December 5, 2023, targeting clinical and non‐clinical professionals in healthcare settings. A non‐probability sample was recruited via a multi‐channel campaign, leveraging national healthcare professional networks and social media. The survey measured respondents' HIV‐related knowledge and training, personal attitudes and behaviours towards people living with HIV (PLHIV).

**Results: **In total, 18,430 healthcare workers from 54 countries responded to the survey. Most respondents were female (74%) and occupied a variety of healthcare roles, although doctors (44%) and nurses (22%) were the most common. Knowledge about HIV on the concept of ‘undetectable equals untransmittable’ (U = U), post‐exposure prophylaxis (PEP), and pre‐exposure prophylaxis (PrEP) varied across types of professional roles and health facilities, with many (69%) not agreeing with correct statements of HIV transmission and prevention. More than half of respondents would be worried when providing care to PLHIV, including drawing blood (57%) or dressing wounds (53%). Eight percent reported they would avoid physical contact and a quarter (26%) that they would wear double gloves when providing care to PLHIV. Twelve percent of healthcare workers preferred not to provide care to people who inject drugs, while 6% preferred not to provide care to men who have sex with men, sex workers, and transgender persons. Twenty‐two percent reported having witnessed unwillingness to provide care, 19% witnessed disclosure of HIV status without consent, 18% poorer quality of care, and 30% discriminatory remarks or talking badly about PLHIV.

**Conclusions: **This study identifies an urgent need for robust, multifaceted interventions, encompassing education and facility‐levels to eliminate stigma, improve HIV knowledge among healthcare workers, and ensure equitable, non‐stigmatizing care for all PLHIV, ultimately contributing to the global goal of ending the AIDS epidemic by 2030.

### OAD3706LB “It's going to be hell”: Impact of the Ghanaian anti‐LGBT bill on the lives and health of sexual minority men and trans and gender diverse people living with HIV

A.O. Gyamerah ^1^, A. Bello^1^, M.B. Tawiah^2^, N.M. Bawa^2^, P.K. Ayeh^3^, P.J. Anatsui^3^, M. Mensah^2^, N.A. Vanderpuye^3^, M. Lightfoot^4,5^, S.A. Lippman^5^

^1^University at Buffalo, Community Health and Health Behavior, Buffalo, United States, ^2^Independent Scholar, Accra, Ghana, ^3^West Africa AIDS Foundation, Accra, Ghana, ^4^OHSU‐PSU School of Public Health, Portland, United States, ^5^University of California, San Francisco, Division of Prevention Science, San Francisco, United States

**Background: **Criminalization of LGBTQ+ Ghanaians poses a threat to the wellbeing of sexual minority men (SMM) and trans and gender diverse (TGD) people living with HIV (PLHIV), populations whose HIV prevalence are 18.1% and 46.1% respectively. In 2021, an anti‐LGBT bill was introduced in Ghana's Parliament and subsequently passed in 2024. To understand the effects of criminalization on Ghana's key populations, we examined the impact of the bill on the lives and HIV treatment and care of SMM and TGD PLHIV.

**Methods: **Between April 2023‐January 2024, we conducted in‐depth interviews (N = 46) and focus group discussions with a subsample of 12 HIV‐positive SMM and TGD people in Accra, Ghana. Participants were purposefully sampled through HIV clinics/organizations. Eligibility criteria were: assigned male at birth, has sex with men, HIV‐positive, and ≥18 years. Questions explored the impact of criminalization and other stressors on mental health and HIV treatment and care. Interview transcripts were analyzed using thematic analysis.

**Results: **Participant mean age was 28.5 years. Most participants shared that the bill negatively impacted their lives and the LGBTQ+ community. Reported impacts included fear of going outside, dating, or socializing with other LGBTQ+ people and increased personal and community experiences of stigma/discrimination and verbal/physical violence. Participants anticipated experiencing more stressors, losing their jobs, being outed, or facing arrest if the bill becomes law; some planned to leave Ghana. Nearly half indicated that the bill had impacted or will impact their HIV treatment and care and that of other SMM and TGD PLHIV, including avoiding clinics in fear of being stigmatized/reported to police/outed as gay by providers; limited/no access to treatment and care; worsening mental distress from living with HIV; and fear of death. Some stopped going for HIV care or stopped taking ARVs; many shared experiencing psychological distresses such as fear, stress, worry, sadness, and suicide ideation.

**Conclusions: **Ghana's anti‐LGBT bill increased violence/other stressors facing SMM and TGD PLHIV and negatively impacted HIV treatment and care. HIV stakeholders must exert pressure against the signage of the bill into law​ and support local groups to mitigate its effects, including provision of mental health support for SMM and TGD PLHIV.

### OAE0406LB Performance characteristics of HIV RNA screening with long‐acting injectable cabotegravir (CAB‐LA) pre‐exposure prophylaxis (PrEP) in the multicenter global HIV Prevention Trials Network 083 (HPTN 083) Study

R. Landovitz ^1^, F. Gao^2^, J.M. Fogel^3^, B. Hanscom^4^, M. Clement^5^, H.V. Tran^6^, A.H. Gaur^7^, C.J. Fichtenbaum^8^, E. Piwowar‐Manning^3^, A. Moser^3^, M.A. Marzinke^3^, J. Mellors^9^, E.K. Halvas^9^, M. McCauley^10^, K. Gomez‐Feliciano^10^, A. Jennings^10^, L. Soto‐Torres^11^, S. Zwerski^11^, J.F. Rooney^12^, C. Acuipil^13^, A.R. Rinehart^14^, M.S. Cohen^6^, B. Grinsztejn^15^, S.H. Eshleman^3^, For the HPTN 083 Study Team

^1^University of California, Los Angeles, David Geffen School of Medicine, Los Angeles, United States, ^2^Fred Hutchinson Cancer Center, Vaccine and Infectious Disease Division, Seattle, United States, ^3^Johns Hopkins University, School of Medicine, Baltimore, United States, ^4^Fred Hutchinson Cancer Research Center, Seattle, United States, ^5^Louisiana State University, Health Sciences Center, New Orleans, United States, ^6^University of North Carolina at Chapel Hill, Chapel Hill, United States, ^7^St Jude Children's Research Hospital, Infectious Diseases, Memphis, United States, ^8^University of Cincinnati, Cincinnati, United States, ^9^University of Pittsburgh, Department of Medicine, Pittsburgh, United States, ^10^FHI 360, Durham, United States, ^11^National Institute of Allergy and Infectious Disease, Rockville, United States, ^12^Gilead Sciences, Foster City, United States, ^13^ViiV Healthcare, Durham, United States, ^14^ViiV Healthcare, Research Triangle Park, United States, ^15^Instituto de Pesquisa Clinica Evandro Chagas‐Fiocruz, Rio de Janeiro, Brazil

**Background: **Long‐acting cabotegravir (CAB‐LA) is highly effective for HIV pre‐exposure prophylaxis (PrEP) but complicates detection of HIV infection. HIV RNA screening is the most sensitive method for detecting infections in persons using CAB‐LA PrEP, usuallydetecting infections before integrase strand transfer inhibitor resistance emerges. We evaluated the performance HIV RNA screening in the HPTN 083 Open Label Extension study (OLE) among MSM/TGW.

**Methods: **In the OLE, sites performed rapid testing, antigen/antibody (Ag/Ab) testing, and HIV RNA testing at every study visit. HIV status was determined based on‐site testing and retrospective testing at a central laboratory. We calculated the positive predictive value (PPV) and false positive rate (FPR) of isolated positive RNA results and the sensitivity of RNA screening with other tests.

**Results: **This analysis included 27,335 visits conducted for 2,620 participants through 11/30/23. Twenty‐nine participants acquired HIV during the OLE. In 5/29 (17.2%), HIV infection was first identified by an isolated positive RNA test result (true positives, Table); in 2 of these cases, HIV infection was first identified at OLE enrollment. Twenty‐three additional participants had an isolated positive RNA test result (22 HIV negative [false positives], 1 HIV status indeterminant). The PPV for detecting infection by RNA screening for participants with vs. without CAB‐LA in the past 6 months was 9.1% (95% CI 1.6, 30.6) vs. 60% (95% CI 17, 92.7), respectively. The FPR and sensitivity for RNA screening for participants with vs. without CAB‐LA in the past 6 months were: FPR: 0.08 (95% CI 0.05, 0.13) vs. 0.06 (95% CI 0.01, 0.24); sensitivity: 87.5% (95% CI 46.7, 99.3) vs. 100% (95% CI 80, 100), respectively.

**Table:** Table. OAE0406LB: HPTN 083 participants who had a positive HIV RNA screening test with non‐reactive HIV rapid and Ag/Ab tests at the first HIV‐positive visit over 3,893 person‐years of follow‐up

Case number	Original randomized study arm	Case type	Days between the last CAB injection and the 1st HIV‐positive visit	Site HIV RNA result at the 1st HIV‐positive visit (copies/mL)​
1	Cabotegravir	Infection detected at OLE entry, >6 months after the last CAB injection	425	1,597
2	Cabotegravir	Infection detected at OLE entry, >6 months after the last CAB injection	486	493
3	TDF‐FTC	Infection during the oral CAB phase	No CAB injections	1,830
4	TDF‐FTC	Infection with delayed CAB injection	38	124
5	TDF‐FTC	Infection with delayed CAB injection	57	4,120

**Conclusions: **HIV RNA screening performed poorly for detecting HIV infection during CAB‐LA PrEP injections; performance was better immediately prior to CAB‐LA initiation. Although infrequent, most isolated positive RNA test results while on CAB‐LA were false‐positive results. Guidelines for HIV testing algorithms designed to screen for long‐acting PrEP failure should consider these performance characteristics.

### OAE2006LB Integrating HIV, tuberculosis and addiction treatment services in primary care clinics in Ukraine: two‐year outcomes from a randomized controlled trial

E. Machavariani ^1^, K. Dumchev^2^, D. Esserman^3^, I. Pykalo^4^, M. Filippovych^2^, R. Ivasiy^1^, D.J Bromberg^3^, L. Madden^1^, M. Haddad^5^, B. Ahmad^1^, D. Oliveros^1^, S. Dvoriak^2^, F. Altice^1^

^1^Yale School of Medicine, New Haven, United States, ^2^Ukrainian Institute of Public Health, Kyiv, Ukraine, ^3^Yale School of Public Health, New Haven, United States, ^4^European Institute of Public Health, Kyiv, Ukraine, ^5^Community Health Center Inc, Middletown, United States

**Background: **Ukraine's HIV epidemic is concentrated among people who inject drugs (PWID). As Ukraine prioritized primary care (PCC) over specialty addiction centers (SAC), we compared quality health indicators (QHIs) in individuals receiving opioid agonist therapies (OAT) in SAC (standard‐of‐care) and PCCs.

**Methods: **Starting in 2018, we conducted a multi‐phase, Type‐2 hybrid implementation trial comparing QHI outcomes (percentage) in participants prescribed OAT in SACs and PCCs in 13 cities. PCC:SAC allocation was 2:1 and PCC‐allocated participants were stratified in clinics receiving pay‐for‐performance (P4P) or not. QHIs were measured every 6 months over 24 months and included standardized measures for specialty (HIV, TB, OAT) and primary care (i.e., recommended screening for prostate/cervical cancers, etc.) outcomes. To increase confidence in providing specialty services in PCCs, clinicians participated in weekly tele‐education training sessions. QHI outcomes were compared between PCC and SAC using likelihood‐based mixed models with missing at random assumptions.

**Results: **Among 1,459 participants enrolled, there were no differences in sex (male = 83%), age (mean = 39 years), HIV (41.9%) and HCV (57.0%) prevalence in those in SAC (N = 509) or PCC (N = 950) participants. At baseline, composite mean QHIs did not differ between arms, however, mean QHIs were consistently higher at PCC vs SAC at 6 (5.7; 95%CI 1.5‐9.9; *p* = 0.010), 12 (9.1; 95%CI 4.8‐13.4; *p*< 0.001), 18 (10.2; 95%CI 5.9‐14.5; *p* <0.001), and 24 (9.0; 95%CI 4.6‐13.3; p< 0.001) months. The composite QHIs were further divided into primary and specialty care outcomes, with primary care QHIs significantly higher at all follow‐up timepoints and specialty QHI scores higher later at 18 and 24 months. Composite and specialty QHIs were significantly higher for P4P versus non‐P4P PCCs only at 24 months.

**Figure**. OAE2006LB

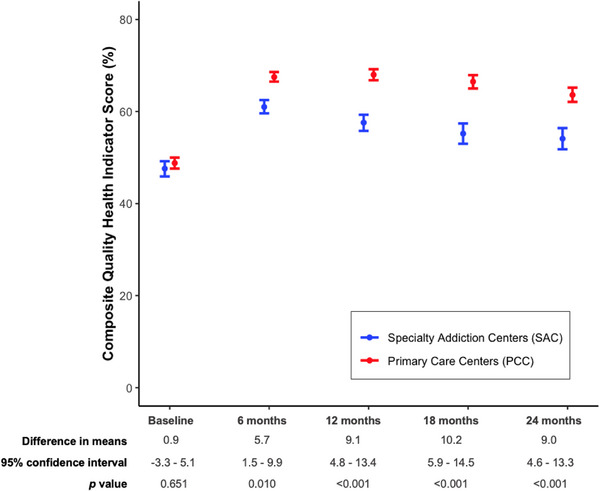

**Conclusions: **Integrating services in PCCs resulted in better comprehensive outcomes relative to SACs for PWID confirming the need to integrate care for PWID with or at high risk for HIV and TB.

### OAE2306LB Differentiated service delivery of PrEP with key population‐ led organizations in Myanmar

T. Yamin Pyone ^1^, L. Htet^1^, L. Aung Thu^1^, Y. Min Thaung^1^, M. Kyaw Kyaw^1^, K. Phyoe Naing^1^, S. Lone Tip^2^

^1^HIV/TB Agency, Information, and Services (AIS) Activity, Community Partners International, Yangon, Myanmar, ^2^United States Agency for International Development (USAID), Yangon, Myanmar

**Background: **In Myanmar, HIV prevalence is significantly high among key populations; 34.9% in PWIDs, 8.8% in MSMs, and 8.3% in FSWs (UNAIDS, 2021) where prevalence among the general population is less than 1%. Pre‐exposure prophylaxis is a proven effective combination prevention globally and is effectively introduced in Myanmar in 2020. Since the reception, HIV/TB Agency, Information, and Services (AIS) Activity, through its two implementing partners, were the first PrEP implementers in Myanmar. In 2022, AIS expanded PrEP with KP‐led CSOs to improve PrEP availability and strengthen the role of KP‐led organizations in HIV service provision.

**Description: **In 2022, AIS collaborated with three KP‐led CSOs and set‐up PrEP distribution points at six KP‐run community outlets, where a medical team from AIS IPs conducted regular mobile visits to the sites. The CSO partners generate the demand, bring clients to the facilities, ensure enrollment in PrEP and other services. AIS IPs provided training to the CSO staff to improve their PrEP knowledge and demand generation capacities to reach the hard‐to‐reach KP. The CSOs partners also take the role in PrEP counseling and baseline investigations for PrEP. After initiating, they also follow‐up the clients for adherence and PrEP refill. In mid‐2023, AIS expanded collaboration with one more CSO for PrEP provision to FSW.

**Lessons learned: **From Feb 2022 to Mar 2024, 1600 clients have been initiated for PrEP through 7 community outlets in Yangon; 1352 MSM, 123 TG, and 125 FSW. This represents around 30% of overall PrEP enrollment of AIS projects. Despite the political instability, KP‐led CSOs improved client recruitment and PrEP service provision through community outlets.

**Conclusions/Next steps: **KP‐led CSO involvement in PrEP service provision improved KP enrollment in PrEP, especially the hidden population. KP‐friendly community outlets provide more options and improve KP access to PrEP. This model improves the capacity of KP CSOs and promotes their role in service provision. Further institutionalization and expansion of this model will improve the PrEP coverage among KP in Myanmar.

### OAE2506LB Incremental uptake of HIV and sexually transmitted infections testing, treatment, and prevention services by integrating gender‐affirming care to sexual health service delivery model: The Tangerine Clinic

B. Sawatwipachai ^1^, S. Youngkong^2^, N. Phanuphak^3^, K. Samitpol^3^, L. Anuratpanich^2^

^1^Faculty of Pharmacy, Srinakharinwirot University, Social and Administrative Pharmacy, Nakhon Nayok, Thailand, ^2^Faculty of Pharmacy, Mahidol University, Bangkok, Thailand, ^3^Institute of HIV Research and Innovation, Bangkok, Thailand

**Background: **The Tangerine Clinic in Bangkok was opened in 2015 to offer a trans‐led health service which integrates gender affirming care with sexual health service. We examined the effectiveness of the Tangerine Clinic model in increasing the utilization of HIV and sexually transmitted infection (STI) testing, treatment, and prevention among transgender clients of the Tangerine Clinic.

**Methods: **The Tangerine Clinic performance outputs in 2021, including number of clients reached, recruited, tested and treated for HIV, syphilis, gonorrhea/chlamydia, post‐exposure prophylaxis (PEP), and pre‐exposure prophylaxis (PrEP), were used for the analysis. We explored the ratio of provider‐initiated HIV testing (conducted when offered as part of gender affirming care) to self‐initiated HIV testing. Then we calculated the hypothetical outputs of four service delivery models: a) solely HIV service, b) integrated hormone and HIV service, c) HIV/STI service, and d) integrated hormone and HIV/STI service.

**Results: **The ratio of provider‐initiated:self‐initiated HIV testing was 0.79:0.21. Integrating gender‐affirming care to HIV service resulted in additional 1,369 HIV testing, 24 clients with HIV diagnoses, 20 starting HIV treatment, 51 starting PEP, and 329 starting PrEP. If gender‐affirming care was integrated into HIV/STI service, 1,594 additional cases would be tested for syphilis, 67 newly diagnosed, and 62 treated. For gonorrhea and chlamydia, 332 additional cases would be tested, 74 newly diagnosed, and 74 treated. When only STI was integrated to HIV service without gender‐affirming care, only 339 syphilis testing were conducted, 14 diagnosed, and 13 treated. Only 71 gonorrhea/chlamydia testing were made, 16 diagnosed, and 16 treated.

**Conclusions: **Integrating gender‐affirming care to sexual health services at the Tangerine Clinic led to an increase uptake of HIV and STI testing and treatment services. PrEP and PEP services can also be scaled up among transgender people through this integrated gender‐affirming and sexual health service model.

### OAE3006LB Moral injury or burnout? The personal impact on health care workers delivering HIV care and treatment in an under‐capacitated health system in Mozambique

C. Audet ^1^, E. Graves^2^, P. Paulo^3^, V. Cumbe^4^, F. Mambo^3^, R. Fernando^3^, T. Oyekunle^2^, C. De Schacht^3^

^1^Vanderbilt University, Health Policy, Nashville, United States, ^2^Vanderbilt University, Institute for Global Health, Nashville, United States, ^3^Friends in Global Health, Maputo, Mozambique, ^4^Central Hospital Beira, Beira, Mozambique

**Background: **People living with HIV enrolled in antiretroviral treatment in Mozambique report being treated with disrespect by healthcare workers (HCW). We have hypothesized that HCWs’ negative behavior towards patients is likely a reflection of burnout, compassion fatigue, and moral injury associated with delivering treatment in resource‐limited settings. A pilot intervention addressing resilience and burnout among health providers to improve provider mental health/wellbeing and clients’ health‐related outcomes began in January 2024 in Zambézia Province, Mozambique.

**Methods: **Four health facilities were randomized to one of four conditions: an anti‐stigma intervention, a resilience intervention, a combination anti‐stigma/resilience intervention, or control. In January and February 2024, we conducted 100 surveys with HCW to measure baseline resilience (decompression and activation factors from the Early Warning Resilience Survey), burnout (Copenhagen Burnout Inventory), and moral injury (MISS‐HF). Data were collected into a secure REDCap database via tablets. Descriptive analysis was performed.

**Results: **Participants were mostly female (63%), married (66%), and median age was 33 years. Forty‐four percent of participants had clinical degrees (e.g., nurses, physicians), 29% were health counselors, and the remainder were in non‐clinical roles. Participants had worked a median of 6.5 years providing health care services. Participants scored very high on the resilience measure (median score 32/40). Few participants reported burnout (1 person scoring above 50, indicating moderate burnout). In contrast, moral injury was identified among 36% of participants (moral injury is identified with a score of 36 or higher). Among the most reported moral injury items, 29% felt guilty they could not prevent someone from dying, 28% acted in a way that violated their own morals, and 21% felt betrayed by other health care professionals. Despite these concerns, only 15% reported that the feelings have caused them significant distress or have impaired their ability to function in relationships, work or in other areas of life that are important to them.

**Conclusions: **With a third of health care workers screening positive for moral injury, delivery of quality HIV services is potentially put at risk. Specific psychosocial support to providers targeted at improving their well‐being could have a positive impact on care delivery.

### OAE3906LB Game‐changing Injectable PrEP: how layering the new HIV prevention method onto existing person‐centered service delivery is supporting client continuation in Zambia

M. Musonda^1^, A. Ndhlovu^2^, L. Kawanga ^2^, D.J. Phiri^2^, J. Musangulule^2^, I. Siluka^2^, N. Chibesakunda^2^, S. Hatchard^2^, D. Mwamba^1^, L. Mulenga^3^

^1^United States Agency for International Development, Health Office, Lusaka, Zambia, ^2^JSI DISCOVER‐Health, Lusaka, Zambia, ^3^The Ministry of Health, Lusaka, Zambia

**Background: **In February 2024, Zambia introduced CAB‐LA, with the USAID DISCOVER‐Health project implementing in six sites in two districts. CAB‐LA has expanded HIV prevention choices for high‐risk individuals, particularly for those who struggle with oral PrEP continuation. Challenges for some include pill burden, lack of privacy and stigma associated with the daily pill.

**Description: **CAB‐LA introduction was layered onto existing oral PrEP service provision, at high performing sites with high PrEP initiation. Best practices from oral PrEP provision were used to address possible adherence issues, including training healthcare workers in comprehensive counseling, and utilizing community health workers as PrEP mentors to generate demand, and provide in‐person psychosocial counseling. To help CAB‐LA clients adhere to appointments, they are provided with appointment cards with the next injection date clearly indicated, coupled with automated reminders and telephone follow‐ups.

**Lessons learned: **From February 9 ‐ March 31, 2024, USAID DISCOVER‐Health initiated 641 clients on CAB‐LA. 208 (32%) were adolescent girls and young women (AGYW), 215 (33%) were adolescent boys and young men (ABYM), 170 (27%) were high‐risk individuals >25 years, 27 (4%) were female sex workers (FSW) and 10 (2%) were men who have sex with men (MSM). As of March 31, 446 clients were due for their one‐month visit (initiation injection two), with 335 receiving the second injection, representing a one‐month continuation rate of 75.1%. When broken down by population type, one‐month continuation was 70%, 82%, 77% and 33% for AGYW, ABYM, FSW and MSM respectively. In the period under review, 631 clients were initiated on oral PrEP at the same sites. 463 clients were due for a one‐month visit, and 345 (74.5%) returned at one‐month.

**Conclusions/Next steps: **Early implementation of CAB‐LA demonstrates minimal difference in continuation between oral PrEP (74.5%) and CAB‐LA (75.1%) at sites with established peer support and strong oral PrEP performance. This demonstrates the timely gains in layering a new HIV prevention option onto a strong existing service delivery platform, with established client trust and skilled staff. Longitudinal tracking of this initial cohort of CAB‐LA clients will be critical to determining CAB‐LA continuation over several months and its possible impact on reducing HIV transmission.

### OAF0306LB Are countries allowing communities to lead? The global landscape of national civil society laws and their association with HIV 95‐95‐95 indicators

S. Mukherjee^1^, V. Srivatsan ^1^, E. Lamontagne^2^, M. Kavanagh^1^

^1^Center for Global Health Policy and Politics, O'Neill Institute for National and Global Health Law, Georgetown University, Washington DC, United States, ^2^The Jointed United Nations Programme on HIV/AIDS (UNAIDS), Johannesburg, South Africa

**Background: **Civil society organizations (CSOs) are essential for the HIV/AIDS response. CSOs mobilize communities, engage with marginalized and key populations, and participate in advocacy efforts to address AIDS‐related stigma and discrimination, and in service delivery by ensuring access to treatment services and treatment adherence support. National laws dictate whether CSOs can register and operate freely and whether CSOs can receive government funds to provide services and are hypothesized to matter in the AIDS response. The actual effect of these laws, however, has not been shown or measured. This study provides an overview of global adoption of civil society related laws and tests their association with HIV 95‐95‐95 targets.

**Methods: **Text of laws and legal information was collected by the HIV Policy Lab through in‐depth legal and policy review for 194 countries from 2017–2023. Laws were coded by legal experts to measure two indicators: i) presence of social contracting policies for financing CSOs and ii) capability of CSOs to register and operate freely under national law. Countries were coded as ‘Adopted’ (both indicators adopted), ‘Partially Adopted’ (one adopted) and ‘Not Adopted’ (neither adopted). Using a cross‐sectional dataset, fractional logistic regression was used to determine associations between the adoption of CSO laws and HIV 95‐95‐95 indicators.

**Results: **The number of countries that adopted and partially adopted Civil Society policies has significantly increased between 2017 (n = 85, 70.8%) and 2023 (n = 90, 84.1%) (χ^2^(1, N = 227) = 4.9, p<0.05). Results from fractional logistic regression show that the odds of PLHIV knowing their HIV status increases by a factor of almost 2 in countries which have adopted civil society freedom policies compared to countries without civil society freedom policies and legislation [OR:1.899, 95%CI:1.42‐2.53,p<0.0001].

**Conclusions: **Legislation and freedom policies for civil society impact national ability to reach AIDS targets. The adoption of national laws supporting both CSO operational freedom and social contracting should be a priority for the global AIDS response. Tracking the national policy environments for CSOs is an important step to ensuring that countries adopt policies that reduce the burden of HIV/AIDS, both nationally and globally.

### OAF1106LB Community‐level HIV stigma and discrimination's impact on HIV testing, treatment uptake, and viral load suppression in 33 African countries: a pooled analysis of 76 nationally representative surveys (2000‐2022)

S. Kuchukhidze ^1^, M.‐C. Boily^2^, S. Niangoran^3^, L. Platt^4^, F. Terris‐Prestholt^5^, K. Dumchev^6^, P. Vickerman^7^, A. Artenie^7^, J. Stone^7^, M. Maheu‐Giroux^1^

^1^McGill University, Department of Epidemiology and Biostatistics, Montreal, Canada, ^2^Imperial College London, MRC Centre for Global Infectious Disease Analysis, School of Public Health, London, United Kingdom, ^3^Programme PAC‐CI/ANRS Research Site, CHU de Treichville, Abidjan, Côte d'Ivoire, ^4^London School of Hygiene and Tropical Medicine, Department of Public Health, Environments and Society, London, United Kingdom, ^5^Warwick Medical School, Coventry, United Kingdom, ^6^Ukrainian Institute on Public Health Policy, Kyiv, Ukraine, ^7^University of Bristol, Bristol Medical School (PHS), Bristol, United Kingdom

**Background: **Stigma and discrimination may hinder reaching the UNAIDS 95‐95‐95 targets for HIV diagnosis, treatment, and viral load suppression (VLS), particularly in high‐burden African countries. Despite global goals of “zero discrimination”, comprehensive, comparable, cross‐country analyses that strengthen the evidence‐base linking stigma to HIV outcomes are lacking.

**Methods: **We pooled individual‐level data from 76 nationally representative surveys with information on HIV stigma, past‐year HIV testing (self‐reported), ART uptake and VLS (both biomarker‐based). We included Demographic and Health Surveys, Population‐based HIV Impact Assessment, and country‐specific surveys. We analyzed three stigma measures:1) discriminatory attitudes towards people living with HIV (PLHIV), 2) shame of associating with PLHIV, and 3) perceived HIV stigma.

We used generalized estimating equations with robust standard errors to estimate adjusted prevalence ratios (aPR) for each stigma measure's association with HIV outcomes. Individual‐level stigma was averaged at a community‐level (survey cluster). Models were adjusted for sex, age group, rural/urban residence, marital status, education, regional HIV prevalence (HIV testing analysis), country and year. We present aPRs for a 50% increase in community stigma prevalence.

**Results: **Data from 842,169 respondents (70,109 PLHIV) across 33 countries were included. Median discriminatory attitudes, shame of associating with PLHIV and perceived stigma were 36% (IQR:19‐64%), 18% (IQR:0‐33%) and 79% (IQR:64‐92%) respectively. All stigma measures were associated with lower past‐year HIV testing (Figure). As community‐level discriminatory attitudes increased by 50%, PLHIV were 17% (95%CI:0.78‐0.87) less likely to be on ART and had 15% lower VLS (95%CI:0.8‐0.9). In communities with greater HIV shame, PLHIV had lower ART uptake (aPR = 0.88; 95%CI:0.81‐0.95) and VLS (aPR = 0.89; 95%CI:0.81‐0.98), with similar results for perceived HIV stigma.

**Figure**. OAF1106LB

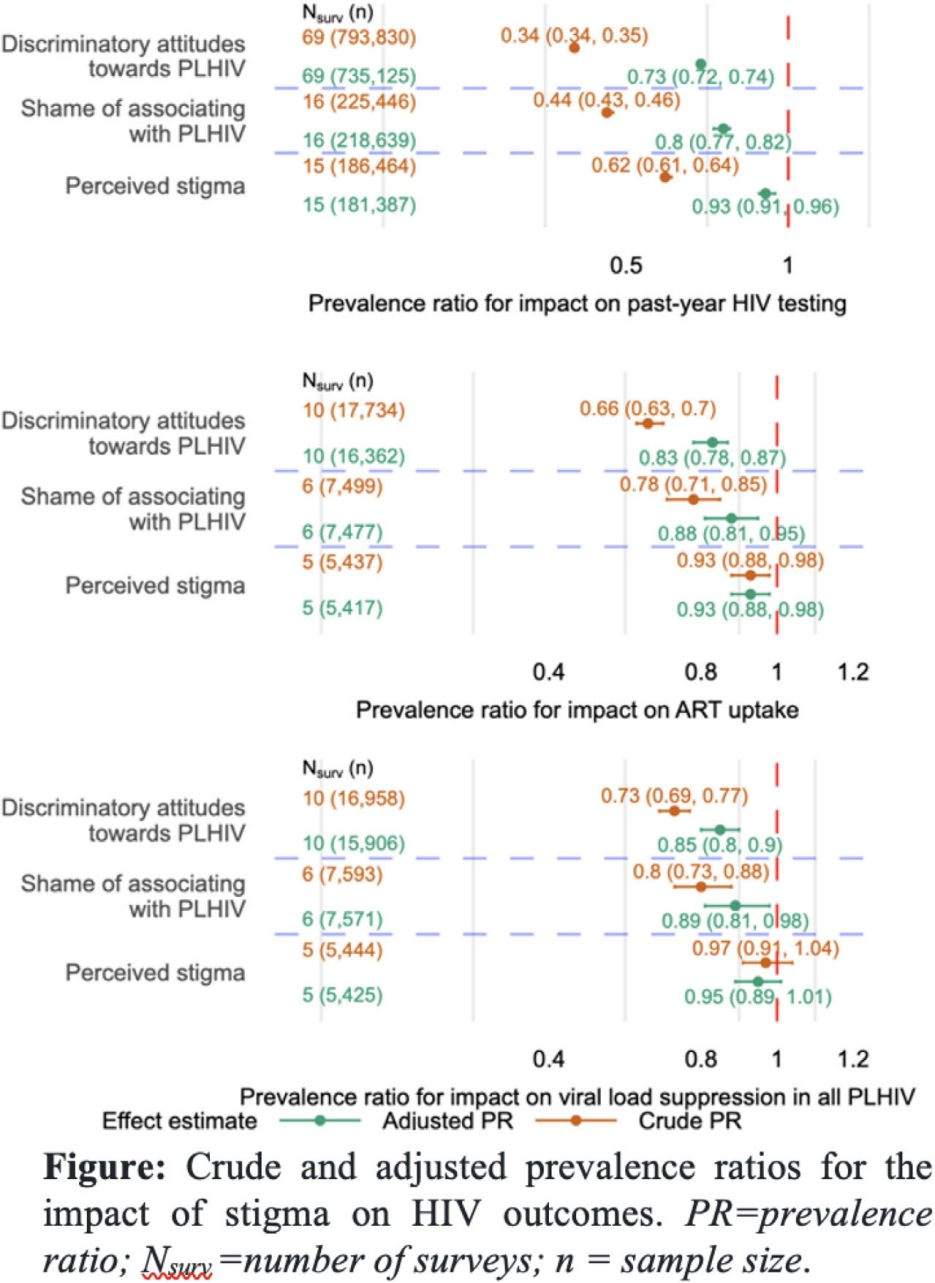

**Conclusions: **In one of the largest studies of this type, stigma was linked with reduced engagement at every stage of HIV care. Addressing stigma and discrimination could strengthen the HIV response and improve treatment outcomes among PLHIV.

### OAF2706LB Sustaining KP service delivery in harsh legal push‐back environments among LGBTQ populations in Uganda amidst the 2023 anti‐homosexuality act (AHA)

F. Engwau ^1^, P. Mudiope^2^, P. Kyambadde^3^, G. Pande^3^, B. Epoku^4^, D. Byamukama^5^, R. Kindyomunda^6^

^1^UNFPA, HIV, Kampala, Uganda, ^2^Ministry of Health, HIVand STI Prevention, Kampala, Uganda, ^3^Ministry of Health, HIV/STI Treatment, Kampala, Uganda, ^4^Ministry of Health, HIV and STI Prevention, Kampala, Uganda, ^5^Uganda AIDS Commission, Prevention, Kampala, Uganda, ^6^UNFPA, HIV/AIDS, Johanesburg, South Africa

**Background: **In 2023, the Ugandan Parliament passed a bill to criminalise homosexuality and proposed harsh penalties to all those that may be convicted of the offence of homosexuality and those abating homosexuality. The social and legal ramifications pushed LGBTQ communities underground, limiting access to HIV prevention services. Although Uganda has made tremendous progress in reducing the prevalence of HIV and AIDS among adults from 18% in 1992 to the current 5.2% (UPHIA 2020), the Ant‐ Homosexuality Act (AHA) threatens these achievements and goals. HIV among men having sex with men is at 13.2%, female sex workers at 31.1%, and people with injecting drug use at 17%.

**Description: **Following the ascent to the AHA 2023, there was a surge in heightened discrimination, violence incidences, arrests, mob justice, services providers fearing to treat LGBTQI, and disruptions to health services utilization. To these consequences, 1) a Response Team (RT) from Ministry of Health, Uganda AIDS Commission, CCM and UNAIDS/UNFPA) was formed to address, 2) the adaptation framework for continuity of services was established, 3) MOH provided a guidance through a circular, on provision of services to all people without discrimination, 4) training of health workers in the provision of KP friendly services in 24 most affected districts. Targeted dialogue meetings reached 180 policymakers, law enforcers and health workers in 24 hotspot districts. The KP community peers were facilitated to conduct client follow up, refills and linkages.

**Lessons learned: **The AHA enactment of 2023 led to increased discrimination, violence, and disruptions in accessing healthcare services for LGBTQ communities in Uganda.

Establishment of a Rapid Response Team (RRT) facilitated coordinated efforts among different partners to address the adverse effects of the legislation on LGBTQ individuals.

Reorientation trainings for service providers and the implementation of adaptation frameworks were effective strategies to ensure continuity of LGBTQ‐friendly services in affected districts.

**Conclusions/Next steps: **The AHA significantly impeded HIV prevention efforts targeting LGBTQ populations, posing a threat to Uganda's goal of ending AIDS as a public health threat by 2030.

Collaborative initiatives involving government agencies, CSOs are vital for mitigating the negative impact of discriminatory laws and promoting access to health services for key populations.

### OAF3106LB Promoting equality in the labor market: a study on the HIV law and discrimination against PLHIV in Brazil

D.A. Calixto ^1^

^1^Fundação Oswaldo Cruz, Public Health, Brasília, Brazil

**Background: **The law 12,984/2014, known as the “HIV Law”, plays a important role in criminalizing discrimination against People Living with HIV (PLHIV) in Brazil, standing out as an strategic tool in combating stigma and discrimination.

**Methods: **The study period covered data from 2015 to 2022, with the setting being Brazil. A retrospective observational study design was employed, analyzing discrimination cases against PLHIV reported to the Federal Public Ministry (MPF). The study population consisted of individuals who faced discrimination due to their HIV status. Data collection involved gathering information from MPF records. Analysis was conducted using Excel, focusing on quantifying, and categorizing discrimination cases by region and type.

**Results: **During the study period, a total of 2,119 discrimination cases against PLHIV were reported in Brazil, with 1,077 cases investigated and resulting in 569 convictions. The Southeast region recorded the highest number of cases (1,012), followed by the Northeast region (520). Primary forms of discrimination included denial of employment (941 cases), dismissal (234 cases), and segregation (103 cases).

**Figure**. OAF3106LB

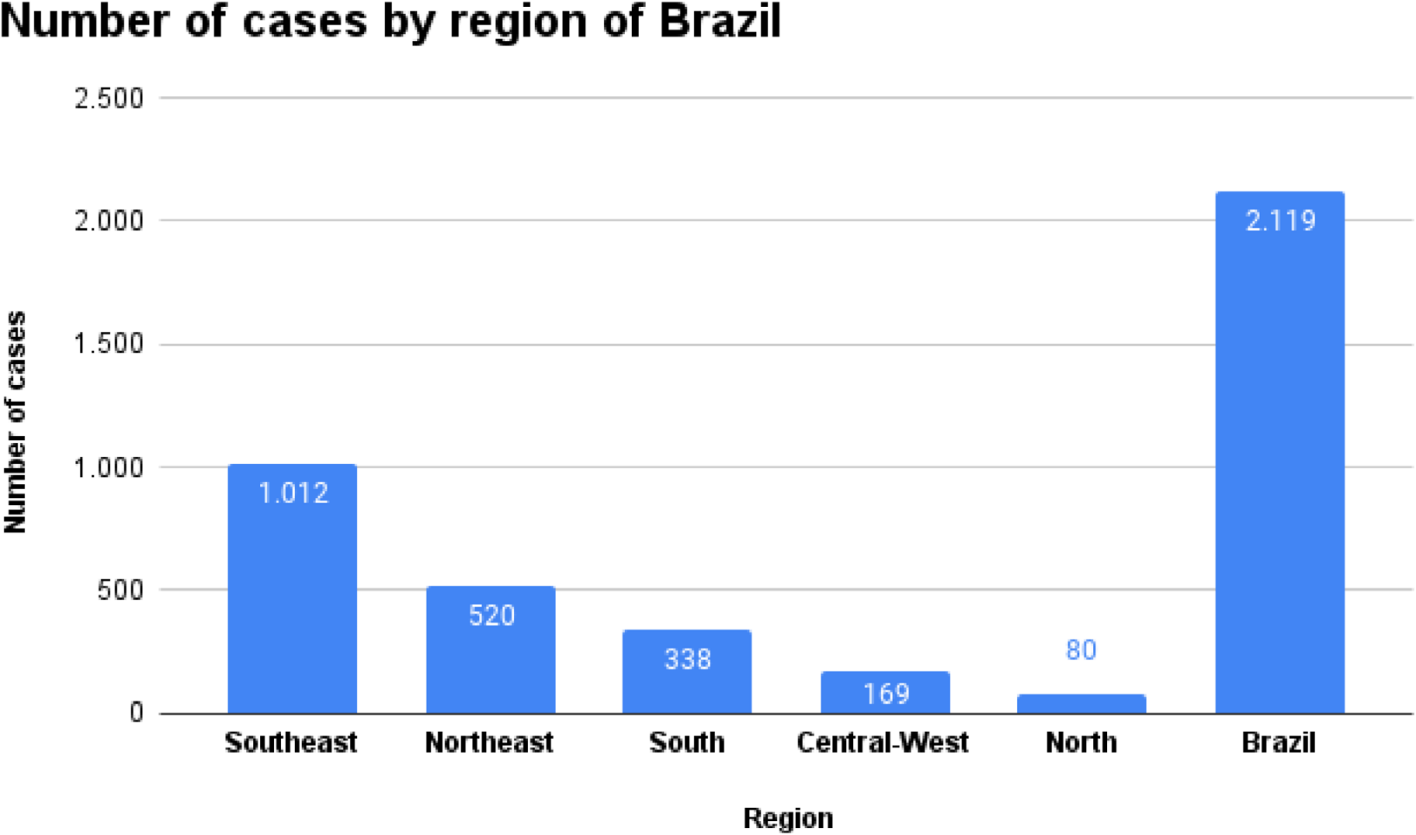

**Conclusions: **Despite advancements in legal frameworks, challenges persist in effective law enforcement, including the need for a greater understanding of legislation and the complexity of proving discrimination, which is often obscured or indirect. The threat of stigma and discrimination undermines fundamental rights at work, highlighting the ongoing necessity to address barriers and foster more inclusive work environments. It is necessary to undertake comprehensive measures to enhance awareness of existing laws, provide robust training for government and organizations of workers and employers, and strengthen mechanisms for reporting and supporting PLHIV who face discrimination.

### OAF4106LB Challenges in access to identification documentation and Protection Services for Unaccompanied Minors and Undocumented Children

D.C. Monare ^1^, P.A. Ncube^1^

^1^University of the Free State, Disaster Management Training and Education Centre for Africa, Bloemfontein, South Africa

**Background: **This research investigates challenges faced by unaccompanied minors and undocumented children in South Africa regarding access to identification documents and protection services. It addresses root causes hindering access, explores interventions, and gathers officials' perceptions from DSD, DOE, and DHA. Lack of documentation jeopardizes access to services, posing risks of statelessness. Climate change exacerbates vulnerabilities. Recommendations include interdepartmental committees, policy changes, temporary documents, and strengthening family tracing efforts to address these complex challenges.

**Methods: **The research delineates a qualitative methodology to explore into accessibility challenges faced by children, particularly regarding identification documents and Child Protection Services (CPS). It involves participants from DHA, DSD, and DOE, employing a multi‐stage sampling technique. Qualitative research, emphasizing depth over quantity, engages 47 respondents through questionnaires, interviews, and workplace observations. Triangulation of content and thematic analysis methods was done to ensure reliability by combining various data sources and analysis techniques, enhancing understanding of challenges and policy practices.

**Results: **The study examines challenges faced by undocumented children and unaccompanied minors accessing basic services, focusing on DOE, DSD, and DHA. DOE challenges are with strict admission policies, leading to dropouts. DSD faces discrepancies in service rendering due to resource constraints. DHA finds it difficult to register undocumented children, compounded by DNA testing requirements. Recommendations include simplifying DHA registration, enhancing interdepartmental collaboration, and improving communication within DOE. These interventions aim to create a more inclusive support system despite persistent challenges, emphasizing streamlined processes and capacity building.

**Conclusions: **In the early 2000s, South Africa faced a surge in HIV/AIDS orphaned children, overburdening the state and family resources. A conference convened to address the crisis, advocated for an intersectoral structure involving government, civil society, and business. This study emphasizes interdepartmental cooperation to support Vulnerable Children (OVC&Y), noting DSD challenges denying services due to lack of documentation. DHA's complex registration, including mandatory DNA testing, which pose financial burdens. Capacity‐building and policy shifts are here‐by urged to streamline services and allocate resources effectively, ensuring coordinated action for OVC&Y protection.

### SS0402LB The next Berlin patient: sustained HIV remission surpassing five years without antiretroviral therapy after heterozygous CCR5 WT/Δ32 allogeneic hematopoietic stem cell transplantation

C. Gaebler ^1^, S. Kor^2^, K. Allers^3^, D. Mwangi^4^, M. Perotti^1^, K. Hanke^5^, K. Meixenberger^5^, V. Corman^6^, T. Burmeister^2^, O. Blau^2^, G. Sürücü^7^, C.G. Schneider^2^, H. Gruell^8^, P. Schommers^9^, F. Klein^8^, L.E. Sander^10^, J. Hofmann^6^, L. Vuong^2^, L. Bullinger^2^, M. Obermeier^4^, I.W. Blau^2^, T. Schneider^3^, O. Penack^2^

^1^Charité – Universitätsmedizin Berlin, corporate member of Freie Universität and Humboldt‐Universität zu Berlin and Berlin Institute of Health, Department of Infectious Diseases and Critical Care Medicine, Berlin, Germany, ^2^Charité – Universitätsmedizin Berlin, corporate member of Freie Universität and Humboldt‐Universität zu Berlin, Department of Hematology, Oncology and Tumor Immunology, Berlin, Germany, ^3^Charité – Universitätsmedizin Berlin, corporate member of Freie Universität and Humboldt‐Universität zu Berlin, Department of Gastroenterology, Infectious Diseases and Rheumatology, Berlin, Germany, ^4^Laboratory MVZ MIB AG, Medical Center for Infectious Diseases, Berlin, Germany, ^5^Robert Koch Institute, Division of Sexually transmitted bacterial Pathogens (STI) and HIV, Berlin, Germany, ^6^Charité – Universitätsmedizin Berlin, corporate member of Freie Universität and Humboldt‐Universität zu Berlin and and Labor Berlin ‐ Charité Vivantes GmbH, Institute of Virology, Berlin, Germany, ^7^Charité – Universitätsmedizin Berlin, corporate member of Freie Universität and Humboldt‐Universität zu Berlin, Institute of Transfusion Medicine, Berlin, Germany, ^8^University of Cologne, Laboratory of Experimental Immunology, Institute of Virology, Faculty of Medicine and University Hospital Cologne, Cologne, Germany, ^9^University of Cologne, Department I of Internal Medicine, Faculty of Medicine and University Hospital Cologne, Cologne, Germany, ^10^Charité – Universitätsmedizin Berlin, corporate member of Freie Universität and Humboldt‐Universität zu Berlin, Department of Infectious Diseases and Critical Care Medicine, Berlin, Germany

**Background: **A scalable cure for HIV remains elusive. Successful cases, including the pioneering cure observed in the so‐called Berlin patient, are limited to individuals receiving allogeneic hematopoietic stem cell transplantations (aHSCT) with homozygous CCR5Δ32/Δ32 allografts that confer resistance to HIV infection. Transplants with functional CCR5 were previously thought to be ineffective for sustaining HIV long‐term remission without antiretroviral therapy (ART). Recently, the case of the Geneva patient demonstrated extended viral control for 18 months after aHSCT from a wild‐type CCR5 donor.

**Methods: **Longitudinal follow‐up analyses of patient samples, including gut biopsies, from 2009 to 2024. Testing for HIV RNA, HIV DNA, viral tropism, CCR5‐expression, viral outgrowth, antiretroviral drug levels and HIV‐specific immune responses.

**Results: **We found prolonged HIV remission exceeding five years without ART following heterozygous CCR5 WT/Δ32 aHSCT for acute myeloid leukemia (AML) in a heterozygous CCR5 WT/Δ32 male. HIV RNA and total HIV DNA were detected pre‐aHSCT with predicted R5 viral tropism. Transplantation from an HLA‐matched (10/10) unrelated donor in October 2015 led to full‐donor chimerism and AML remission. Acute graft‐versus‐host disease (Grade I) was limited to the skin and treated with topical steroids. CD4+ T cell CCR5 expression levels matched CCR5 WT/Δ32 controls. HIV remains undetectable in plasma (LOD 20 copies/ml) 5.5 years after treatment interruption (TI) in September 2018. Repeated HIV DNA measurements were negative in peripheral blood as well as duodenal and ileum biopsies. No viral outgrowth was detected from stimulated CD4+ T cells. Antiretrovirals were undetectable throughout TI, with HIV‐specific antibody levels decreasing and no detectable HIV‐specific T cell responses post‐aHSCT.

**Conclusions: **HIV cure induced by aHSCT is not restricted to the use of homozygous CCR5Δ32/Δ32 donors. Effective reservoir reductions, durable HIV remission and potential cure can also be achieved with functional viral co‐receptors, suggesting that allogeneic immunity fundamentally contributes to HIV eradication.

**Figure**. SS0402LB

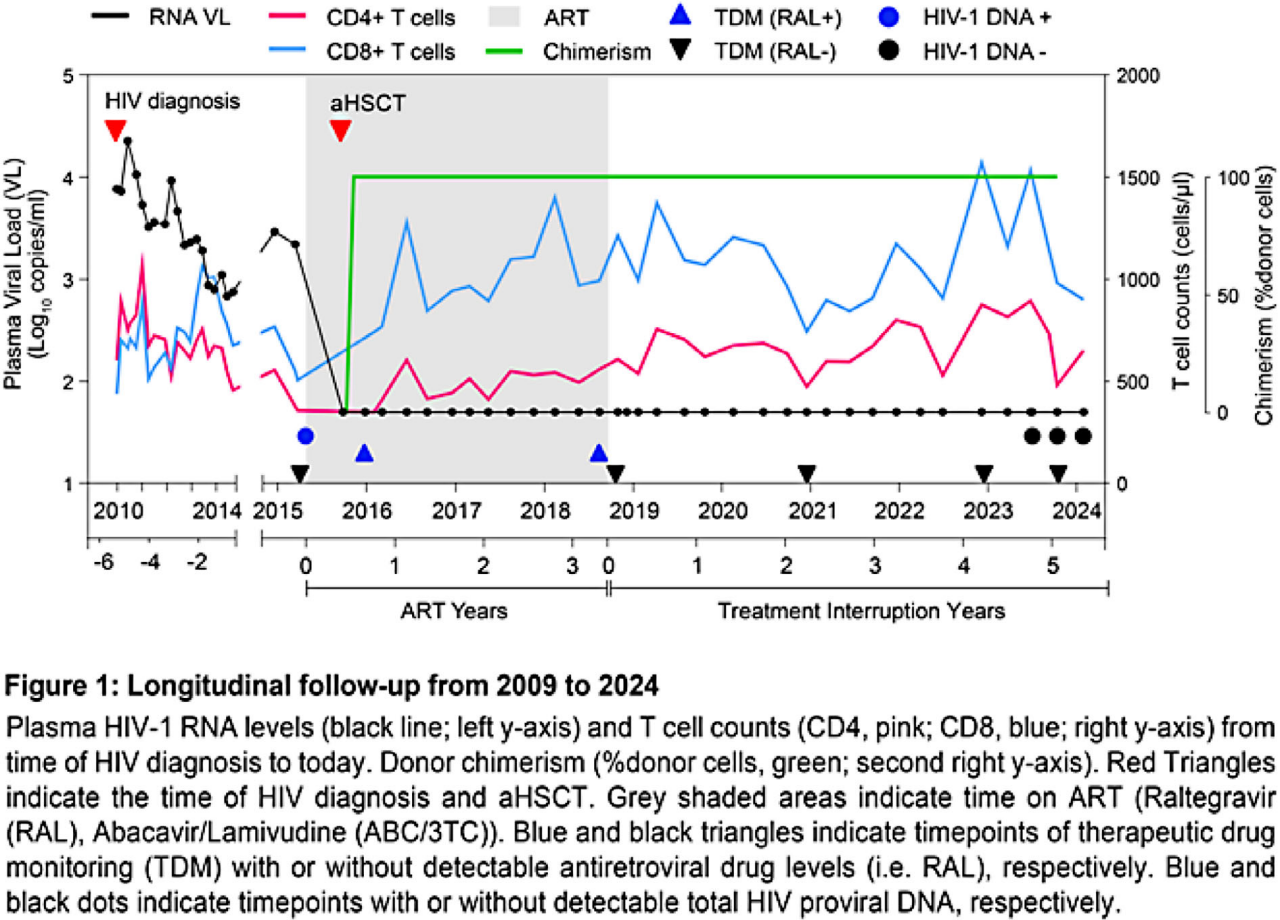

### SS0403LB No confirmed virological failures (CVF) for 144 weeks when switching 2‐/3‐/4‐drug ART to DTG/3TC in heavily treatment‐experienced PLWHA with prior M184V/I and virological failures (VF) in the prospective SOLAR‐3D study

G. Blick ^1^, E. Cerreta‐Dial^1^, G. Mancini^1^, A. Cosenza^1^, L. Fang^2^

^1^Health Care Advocates International, Stratford, United States, ^2^Pharstat, Raleigh, United States

**Background: **DTG/3TC is not approved for stable‐switch in individuals with known history of DTG and/or 3TC resistance or prior VF. The majority of heavily treatment‐experienced(HTE) PLWHA do not qualify for on‐label switch to DTG/3TC. Large prospective studies evaluating DTG/3TC's ability to maintain PCR<50 in virologically suppressed individuals switching ART in the setting of prior/current M184V/I and multiple VF are lacking. SOLAR‐3D, the largest and longest prospective comparative study to‐date, preliminarily demonstrated no CVF through 96‐weeks. Week 144 results will be presented.

**Methods: **SOLAR‐3D, a prospective, open‐label, comparative 144‐week study, evaluates switching 2‐/3‐/4‐drug ART to STR DTG/3TC in HIV‐1 HTE adults with multiple prior VF, prior/current M184V/I, virologic suppression for ≥6mos, and ≥2 prior ART.

Participants were consented/enrolled from 5/2/2019‐10/16/2020 at the NGO, HCAI, in Stratford CT(USA).

There were no exclusions for prior INSTI use, any CD4, prior M184V/I or K65R, or 3TC‐associated mutations detected at BL by Proviral DNA NGS.

Week‐144 results were analyzed by ITT‐E and PP using FDA snapshot analyses.

**Results: **N = 100 participants switched to DTG/3TC, n = 50 with historical/prior M184V/I (37% with current M184V/I by Proviral DNA NGS) and n = 50 without prior M184V/I. Participants with prior M184V/I had significantly greater median prior VF (n[IQR]: 9[7‐13] vs 4[3‐5], p<0.001), longer duration HIV (28.4 vs 15.5yrs, p<0.001), longer ART duration and duration PCR<50c/mL, and were older with lower nadir CD4. Median time on DTG/3TC was 192‐weeks for both groups.

Through Week 144, no difference in efficacy was observed between those with vs without prior M184V/I:

• Primary Endpoint: PCR≥50, n[%]: 2[4%, 2 of 2 re‐suppressed] vs 3[6%, 2 of 3 re‐suppressed], by ITT‐E (5.1% vs 7.9%, PP);

Secondary Endpoint: PCR<50, n[%]: 37[74.0%] vs 36[72.0%], by ITT‐E (94.9 vs 92.3%, PP);

• No CVFs, treatment‐emergent resistance were observed, nor differences regarding PCR TND(<20), viral blips, AEs, or treatment discontinuations.

**Conclusions: **SOLAR‐3D is the largest prospective trial to demonstrate neither prior nor current M184V/I impact the the efficacy and durability of switching virologically suppressed PLWHA with prior VF to DTG/3TC through 144‐weeks. Switching to DTG/3TC must be explored in economically‐developing countries where economic/safety advantages of discontinuing TDF could be consequential.

### SS0404LB Antimicrobial resistance in *Neisseria gonorrhoeae* infections among MSM on Doxycycline post‐exposure prophylaxis

B. Bercot ^1^, L. Assoumou^2^, F. Camelena^1^, C. Voitchouck^3^, M. Mainardis^3^, A. Braille^3^, M. Merimeche^3^, M. Ouattara^2^, E. Rubenstein^4^, A.D. Kaba^2^, G. Pialloux^5^, C. Katlama^6^, L. Surgers^7^, L. Slama^8^, J. Pavie^8^, C. Duvivier^9^, C. Bebear^10^, V. Petrov‐Sanchez^11^, J. Ghosn^12^, D. Costagliola^2^, J.‐M. Molina^4^, ANRS174 DOXYVAC Study Group

^1^IAME UMR1137, Assistance‐Publique Hôpitaux de Paris, University of Paris Cité, Department of Bacteriology and the Associated laboratory of French National Center for Bacterial Sexually Transmitted Infections, St Louis Hospital, Paris 10, France, ^2^Institut Pierre Louis d'Epidemiology et de Santé Publique INSERM UMRS 1136, Sorbonne University, Paris, France, ^3^Assistance‐Publique Hôpitaux de Paris, Department of Bacteriology and the Associated laboratory of French National Center for Bacterial Sexually Transmitted Infections, St Louis Hospital, Paris 10, France, ^4^Hospitals Saint‐Louis and Lariboisière, INSERM U944, Assistance‐Publique Hôpitaux de Paris, University of Paris Cité,, Department of Infectious Diseases, Hospitals Saint‐Louis and Lariboisière, Paris, France, ^5^Sorbonne Université, Department of Infectious Diseases, Hôpital Tenon, Paris, France, ^6^Sorbonne Université, Department of Infectious Diseases, APHP, Hôpital Pitié Salpetrière, Paris, France, ^7^Sorbonne University, INSERM, Institut Pierre Louis d’Épidémiologie et de Santé Publique, Department of Infectious Diseases, Saint‐Antoine Hospital, Paris, France, ^8^Paris Cité university, Department of Clinical Immunology, Hotel‐Dieu Hospital, Paris, France, ^9^Paris Cité university, Department of Infectious Diseases, Hotel‐Dieu Hospital, Paris, France, ^10^Bordeaux University Hospital Armyne Team, UMR CNRS 5234, MFP, University of Bordeaux, Department of Bacteriology, French National Center for Bacterial Sexually Transmitted Infections, CHU de Bordeaux, Bordeaux, France, ^11^ANRS/MIE research agency (National Agency for Research on AIDS and Emerging Infectious Diseases), Research Department, Paris, France, ^12^IAME UMR1137, Assistance‐Publique Hôpitaux de Paris, University of Paris Cité, Department of infectious Disease, Bicaht Hospital, Paris, France

**Background: **Prevention of bacterial STIs with post‐exposure doxycycline (Doxy‐PEP) in MSM raised concerns regarding antimicrobial resistance (AMR). We studied the impact of Doxy‐PEP on *Neisseria gonorrhoeae* AMR in the ANRS DOXYVAC trial.

**Methods: **545 MSM on HIV PrEP were randomized to Doxy‐PEP (n = 362) or No‐PEP (n = 183) and followed for a median of 14 months. Participants were tested at baseline and every 3 months by nucleic acid amplification technic (NAAT) using the Cobas 6800 (Roche) and culture for GC detection in urine, oro‐pharyngeal and anal samples. Etest (Biomerieux) determined MICs and EUCAST guidelines were used for interpretation. Molecular analysis was performed by Whole Genome Sequencing on GC isolates and on NAAT‐positive samples in search of molecular determinants of resistance (*tetM* gene, V57M substitution in S10 protein, MtrR and its promotor modification, mutations in the genes coding for 23S rRNA, S91F substitution in GyrA protein, *penA* mosaic gene). P‐values were calculated using Fisher's exact test.

**Results: **From January 2021 to February 2023, 450 samples (278 patients) were GC‐positive by NAAT. Seventy‐eight GC obtained in cultures (7 at baseline, 40 No‐PEP group, 31 Doxy‐PEP group) and 231 GC‐NAAT‐positive samples (38 at baseline, 99 No‐PEP group, 94 Doxy‐PEP group) were retained for molecular testing.

MICs of ceftriaxone, fluoroquinolones and aminoglycosides were similar in the Doxy‐PEP and No‐PEP groups and no significant change in genetic determinants for these antibiotics was found. Only TEM‐1 genes associated with penicillin resistance were more frequently observed in the doxyPEP group than in the no‐PEP group (40.4% *vs* 17.5% of cases respectively, p = 0.04).

All GC isolates were resistant to tetracycline, with a significant increase in rate of high‐level tetracycline resistance in the Doxy‐PEP *vs*. No‐PEP group (35.5 vs 12.5%, respectively p = 0.04). In addition, the genetic determinant *tetM* was significantly more frequent in the Doxy‐PEP group as compared to the No‐PEP group (55/94 = 59.1% *vs* 23/99 = 23.7%), respectively (p<0.0001). No mutation in the gene encoding 23S rRNA was observed.

**Conclusions: **All GC were resistant to tetracycline but rate of high‐level resistance mediated by the *tetM* gene were higher with Doxy‐PEP. No impact of Doxy‐PEP on Ceftriaxone susceptibility was found.

### SS0405LB Tenofovir‐diphosphate concentrations and viral suppression following monthly point‐of‐care urine tenofovir testing among adults initiating antiretroviral therapy: primary outcome of the randomised controlled STREAM HIV trial

N. Garrett ^1^, K.K. Thomas^2^, J. Dorward^3^, Y. Sookrajh^4^, E. Hill^2^, A. Bardon^5^, M. Khanyile^1^, U. Singh^1^, P. Munatsi^1^, N. Samsunder^1^, M. Wang^2^, P. Belaunzaran‐Zamudio^6^, N. Naicker^1^, D. Donnell^2^, C. Celum^2^, M. Gandhi^7^, P. Moodley^8^, T.R. Cressey^9^, P.K. Drain^2^

^1^Centre for the AIDS Programme of Research in South Africa, Durban, South Africa, ^2^University of Washington, Department of Global Health, Seattle, United States, ^3^University of Oxford, Nuffield Department of Primary Care Health Sciences, Oxford, United Kingdom, ^4^eThekwini Municipality Health Unit, Durban, South Africa, ^5^Washington University, St. Louis, United States, ^6^Division of AIDS, National Institute of Allergy & Infectious Diseases, Rockville, United States, ^7^University of California San Francisco, Department of Medicine, San Francisco, United States, ^8^University of KwaZulu‐Natal, Discipline of Virology, Durban, South Africa, ^9^Chiang Mai University, AMS‐PHPT, Faculty of Associated Medical Sciences, Chiang Mai, Thailand

**Background: **Point‐of‐care urine tenofovir (TFV) tests may improve HIV treatment outcomes and need to be assessed in randomized trials. The STREAM HIV trial evaluated whether monthly urine TFV testing among people with HIV (PWH) initiating dolutegravir‐based antiretroviral therapy (ART) improved adherence.

**Methods: **We recruited PWH initiating first‐line ART at three public clinics in South Africa and randomised participants 1:1 to the intervention (monthly point‐of‐care urine TFV testing [UCSF/Abbott] with adherence counselling) or control arm (monthly adherence counselling without urine TFV testing). The primary outcome was adherence at 24 weeks, assessed by intracellular TFV‐diphosphate concentrations in dried blood spots using mass spectrometry. Secondary outcomes were retention‐in‐care and viral suppression (VS <200 copies/ml). We compared log_10_ TFV‐diphosphate concentrations using t‐tests and estimated risk ratios (RR) using modified Poisson regression.

**Results: **539 participants (58% female, mean age 33 years, CD4 count 393 cells/µl, median viral load 38,163 copies/ml, 5.8% active TB) were initiated on ART between 02/2021‐06/2023. At 24 weeks, 242/270 intervention and 234/269 control arm participants had TFV‐diphosphate results for analyses. Geometric mean TFV‐diphosphate concentrations were similar among intervention and control participants (1,253 versus 1,198 fmol/punch, p = 0.510). However, the proportion with detectable TFV‐diphosphate (≥200 fmol/punch) was higher in the intervention than control arm (98.8% versus 92.7%, RR = 1.06, 95%CI 1.02‐1.11, p = 0.001), Figure 1. Retention‐in‐care (88.5% versus 86.2%, RR = 1.03, 0.96‐1.09), VS (93.8% versus 91.4%, RR = 1.03, 0.97‐1.08) and Retained+VS (82.2% versus 78.8%, RR = 1.04, 0.96‐1.13) were slightly higher in the intervention than control arm, but not significantly different.[Fig jia226279-fig-0035]

**Figure 1 jia226279-fig-0035:**
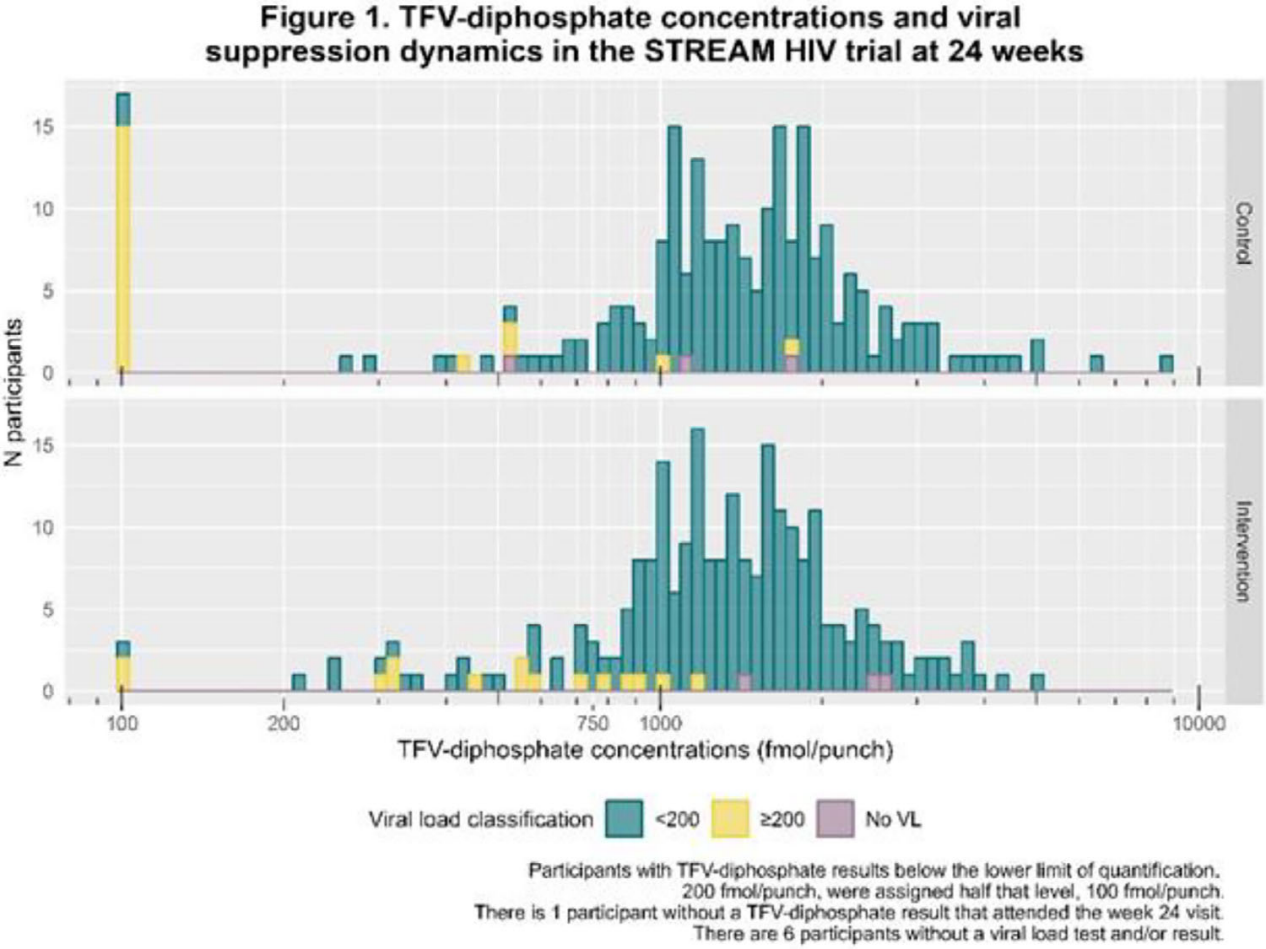
SS0405LB

**Conclusions: **In this South African cohort initiating dolutegravir‐based ART, overall adherence and VS were high at 24 weeks. Monthly point‐of‐care urine TFV testing led to more participants achieving detectable TFV‐diphosphate concentrations, but did not produce higher drug concentrations or more VS. The impact of urine TFV testing in combination with point‐of‐care viral load testing will be assessed at 72 weeks.

### SS0406LB Automated 24/7 dispensing of HIV prevention goods and self‐tests via vending machines

R. Poverga ^1^

^1^Public Association “Positive Initiative”, Chisinau, Moldova, the Republic of

**Background: **To overcome barriers in accessing HIV prevention products and self‐tests—such as reducing dependency on physical interactions with staff, operating hours, and geographic limitations of service providers—in 2023, a network of vending machines (VMs) was launched in Moldova as part of enhanced HIV/AIDS response measures.

**Description: **The VM network was launched in 2023, it consisted of 25 vending machines that provide free, round‐the‐clock, automated dispensing of preventive goods and self‐testing kits for HIV. These were located in 14 major cities across Moldova and are maintained by nine CSOs providing HIV prevention services. Utilizing RFID cards, clients can access goods in accordance with limits and sets based on their affiliation with one or more KG. The VMs are integrated into a digital ecosystem and provide real‐time information on the utilization of goods linked to the client's de‐personalized demographic profile.

**Lessons learned: **From 1 June 2023 to 29 April 2024, the VMs provided access to preventive goods and HIV self‐tests 29,836 times to 3,513 representatives of key groups. Considering that a social worker typically spends 3 to 7 minutes per transaction for the distribution and registration of preventive goods, the automated dispensing saved over 300 workdays, which were reallocated to addressing complex cases in providing HIV prevention, care, and support services. The service was most popular among PWUD (3,015 of 3,513), followed by SW (619), MSM (104), and PLHIV (131), noting that one individual may belong to multiple key groups. 26.56% of users were female and 73.44% were male.

**Conclusions/Next steps: **The 24/7 automated dispensing of HIV preventive goods is essential for broadening service access. It eliminates barriers like discrimination and geographic limitations by allowing non‐stop access without direct human contact, enhancing service perception through initiatives like prize draws. The pilot's success demonstrates potential for expansion, particularly to regions like penal systems with restricted access. Digital tracking enables targeted, data‐driven resource allocation by providing insights into user trends and needs.

## AUTHOR INDEX

### A

Abang, R. OAC2905

Abay, E.S. OAD1905

Abba, M. OAB0103

Abdul, N. OAD0505

Abe, S. OAC0803

Aberg, J.A. OAB3406LB

Abimiku, A. OAA0605

Abiye, K. OAC2905

Acuipil, C. OAB2604, OAE0406LB

Adam, A. OAC3303

Adams Larsen, M. OAD1405

Adams, R. OAC4003

Adegboye, A. OAE2004

Adeniyi, D.S. OAB1706LB

Adiema, L. OAE1204

Afele, C. OAD1603

Afroz, F. OAE3003

Agina, C. OAD0505

Aguirre‐García, G.M. OAC1506LB

Agwo, M.D. OAB1706LB

Agwu, A. OAB2606LB

Ahmad, B. OAE2006LB

Ahmad, N. OAB3604

Ahmed, A. OAD2402

Ahumada Topete, V.H. OAE0403

Ajulong, C. OAC1802

Ake, J. OAB3404

Ake, J.A. OAB2103

Akhtaruzzaman, M. OAC1502, OAE3003

Akiy, Z.Z. OAE2304

Akolo, C. OAE2505

Akpan, U. OAE2004

Alamo, S. OAC1802

Alan, V. OAC1002

Aldrovandi, G. OAA0602

Alejos, B. OAB3606LB

Alford, K. OAB3605

Ali, E. OAC3303

Allen, M.A. OAA1305

Allers, K. SS0402LB

Alles, M.A. OAA0603

Alter, G. OAA0602

Altice, F. OAE2006LB

Altice, F.L. OAC2906LB

Alvarez, A. OAC2203

Amador, C. OAB3606LB

Amalumilo, M. OAE2502

Amamilo, I. OAE0402

Ameachi, P. OAC2905

Amenya, E. OAF1104

Amico, K.R. OAD0706LB

Amole, C. OAE0402

Amorim, L. OAC0805

Amos Ndelwa, L. OAD0505

Anatsui, P.J. OAD3706LB

Ancuta, P. OAA3502

Anderson (Melunsky), K. OAB1703

Anderson, D. OAB3604

Anderson, J.L. OAA1308

Andkhoie, M. OAC1003

Ando, N. OAC0803

Andrew, A. OAA2802

Anika, O. OAE2004

Anoubissi, J.d.D. OAD3204

Anuratpanich, L. OAE2506LB

Anyebe, V. OAB2103, OAB3404

Anyona, M. OAE1202

Apornpong, T. OAB3603

Araínga, M. OAA2804

Aranda Audelo, M. OAE0403

Araujo Coelho, D.R. OAE3002

Arciniegas, A. OAD0705

Arif, M.S. OAA2804

Arodi, S. OAC2903

Arsolino, C. OAC1004

Artenie, A. OAF1106LB

Arts, E. OAB3802

Asaolu, O. OAE2004

Asare, K. OAB3403

Asewe, M. OAE1202

Asif, M. OAC1503

Asowata, O. OAA2803

Assoumou, L. SS0404LB

Atkins, K. OAD3202, OAD3204

Atre, S. OAF1102

Atuhura, S. OAD1903

Atujuna, M. OAD3705

Atyeo, C. OAA0602

Audet, C. OAE3006LB

Aung Thu, L. OAE2306LB

Aungkulanon, S. OAC3305

Aurpibul, L. OAB2104, OAB2606LB

Avettand‐Fenoël, V. OAA0205

Aviah, C. OAE2502

Avihingsanon, A. OAB1702, OAB3603

Aviles‐Guaman, C. OAE0405

Awad, K. OAC3303

Ayebare, P. OAB1704

Ayeh, P.K. OAD3706LB

Ayer, A. OAE0405

Ayieko, J. OAB2105, OAE1203

Ayitewala, A. OAB3802

### B

Babirye, L. OAC1802

Bacon, M. OAE1203

Baeten, J.M. OAC0802

Bagarukayo, A. OAC1505

Bahemana, E. OAB2103, OAB3404

Bailin, S. OAA3505

Balakasi, K. OAD2404

Balham, L. OAD0705

Balla, S. OAB2605

Balsamo, S.A. OAE2305

Baltrusaitis, K. OAB2606LB

Balzer, L. OAE1203

Balzer, L.B. OAB2105

Banda, A. OAF4104

Banda, B. OAC4003

Banda, T. OAD2404

Banda‐Mabuda, H. OAB2102

Bandason, T. OAB2102

Banerjee, P. OAE1202

Baral, S. OAD3202, OAD3204

Barbour, R. OAD1402

Bardon, A. SS0405LB

Barke, L. OAD1403

Barninghausen, T. OAD3704

Bartholomew Boniface, O. OAC2905

Bartholomew, T.S. OAD0706LB

Bashorun, A. OAE2004

Bassil, D. OAD3704

Basu, S. OAA1304

Batrakova, E.V. OAA2805

Batusa, J. OAE0402

Bauer, N. OAB3602

Baumhart, C. OAE2303

Bautista‐Arredondo, S. OAD1904

Bawa, N.M. OAD3706LB

Baylon‐Valdez, V. OAC1506LB

Bbosa, R. OAC1802

Bebear, C. SS0404LB

Becerra Flores, M. OAA1304

Beckford Jarrett, S. OAC3302

Bekker, L.‐G. OAD3705

Belaunzaran‐Zamudio, P. SS0405LB

Belizario, F. OAD1602

Bell, E. OAF4103

Belli, R. OAA1303

Bello, A. OAD3706LB

Belo, C. OAC1005

Benade, M. OAE3905

Bender, M. OAA1306LB

Benedetti, M. OAC2902

Bercot, B. SS0404LB

Bernadin, G.R. OAB3805

Bernal Morell, E. OAB2604

Bernal, E. OAB3606LB

Bernard, C. OAC4004

Bertoni, N. OAC1004

Berzofsky, J.A. OAA1304

Best, B. OAB2606LB

Bethony, J. OAA1308

Bhandari, S. OAF1103

Bhavsar, S. OAE2503

Bhengu, T. OAB2605

Biringmiap, L.K. OAB1706LB

Bispo Amaral, S. OAF4102

Bissa, M. OAA1304

Bissai, R.G. OAE2304

Biswas, K. OAC1503

Biswas, P. OAB2605

Biswas, S. OAA3504, OAC3306LB

Black, D. OAB2105

Blanco, J.L. OAB3606LB

Blau, I.W. SS0402LB

Blau, O. SS0402LB

Blick, G. SS0403LB

Blitz, L. OAE3902

Bloch, M. OAB2602

Bloomfield, G.S. OAB3406LB

Boers, S. OAE3902

Boily, M.‐C. OAC2205, OAF1106LB

Bolton Moore, C. OAB2606LB

Bongo, N. OAD3705

Boniface, S. OAC2202

Bonnardeaux, D. OAD2405

Bootchadee, S. OAF3104

Borah, B. OAC1503

Bosma, M. OAD1403

Bosomprah, S. OAC4003

Bosques‐Padilla, F.J. OAC1506LB

Bottomley, C. OAB3403

Boulle, A. OAB1703

Boulware, D.R. OAB1704

Bouwmeester, S. OAE2305

Braille, A. SS0404LB

Brar, I. OAB3406LB

Braun, D. OAA0204

Bravo, J. OAB3606LB

Bray, K. OAD1403

Bremer, V. OAD3203

Brion, S. OAD3204

Bromberg, D.J. OAC2906LB

Brooks, L. OAD3205

Brown, B. OAA0606LB

Bryant, D. OAC1003

Buck, W.C. OAC2202

Buisson, S. OAB2606LB

Bukusi, E. OAB2105

Bukusi, E.A. OAC0802, OAE1202, OAE1204

Bullinger, L. SS0402LB

Burmeister, T. SS0402LB

Burns, F. OAD3206LB

Burwitz, B. OAA3504

Busza, A. OAB3605

Butler‐Smith, C. OAD1403

Bwalya, C. OAE2303

Bwalya, I. OAC2206LB

Byakwaga, H. OAC4004

Byamukama, D. OAF2706LB

Byrne, S. OAB2603

### C

Cabrera, A. OAC1805

Cafaro, A. OAA1303

Cafun‐Naidoo, T. OAB2605

Cahn, P. OAB0103

Calixto, D.A. OAF3106LB

Calvet‐Mirabent, M. OAA2802

Cambiano, V. OAE1205

Camelena, F. SS0404LB

Cameron, C. OAA0603

Cameron, M. OAA0603

Camiro‐Zuñiga, A. OAE0403

Camlin, C. OAB2105, OAD0503, OAE1203

Campagna, M. OAA1303

Canhanga, O. OAE3903

Cao, P. OAE2005

Cao, R. OAA1308

Capparelli, E. OAB2606LB

Carbuccia, M.E. OAD0705

Cardoso, S.W. OAC1004

Cardozo, T. OAA1304

Carpino, T. OAD3202

Carrico, A. OAD0706LB

Castillo, G. OAE3904

Castillo, T. OAD1902

Castillo‐Mancilla, J.R. OAB2603

Castillo‐Reyna, L.G. OAC1506LB

Cattin, A. OAA3502

Cavanaugh, J.S. OAB2103, OAB3404

Cazaboun, Y. OAB2605

Celum, C. SS0405LB

Cerecero‐García, D. OAD1904

Ceres, M. OAC0804

Cermakian, N. OAA3502

Cerreta‐Dial, E. SS0403LB

Cesar, C. OAB0103

Chacha, J. OAE3903

Chaillon, A. OAA0205, OAA2802

Chale, F. OAC2202

Chalmers, C. OAD1403

Chamie, G. OAB2105, OAE1203

Chang, J.J. OAA1308

Chang, M. OAC1504

Chang, S.‐Y. OAB0105

Chapola, K. OAE2502

Chappell, E. OAB3803

Charre, C. OAA0205

Chasara, C. OAA2806LB

Chatora, K. OAD0703

Chatterjee, D. OAA3502

Chehab, J. OAB2106LB

Chen, J.Y.‐H. OAD1603

Chen, T. OAA0602

Chen, Y.‐K. OAB1705

Chetchotisakd, P. OAB1702

Chetty, K. OAA2803

Chetty, T. OAB2605

Cheung, A. OAB2606LB

Chibesakunda, N. OAE3906LB

Chidhanguro, K. OAE1205

Chikonde, J. OAD2404

Chikowore, T.J.B. OAA0203

Chimbaka, H. OAD2404

Chimbidzikai, T. OAD2405

Chimedza, D. OAD2405

Ching‐Wen L, W. OAA0603

Chirwa, L. OAB3806LB

Chirwa, T.C. OAC2204

Chisenga, M. OAB2102

Chisenga, T. OAC2206LB

Chitembo, L. OAC2206LB

Chiu, C. OAA1308

Chiu, F. OAD3204

Chiyenu, K. OAC1803

Choi, H. OAD0703

Chokephaibulkit, K. OAB2104

Chowdhury, A. OAE3003

Chuaypen, N. OAB3603

Chung, C. OAF4103

Chunga, L. OAC4003

Cisse, V.M.P. OAC3304

Claass, J. OAC0804

Claassen, C. OAC2206LB, OAE2303

Clement, M. OAE0406LB

Clifton, B. OAD1603

Clotworthy, B. OAD1403

Cluver, L. OAD0502

Co Chukwu, C. OAA0605

Cogle, A. OAD1603

Cohen, M.S. OAE0406LB

Collie, S. OAB3403

Collins, I.J. OAB3803

Collins, S.E. OAB3805

Conroy, J. OAE0402

Copas, A. OAE1205

Cordner, D. OAD1603

Corey, L. OAA1305

Corman, V. SS0402LB

Cosenza, A. SS0403LB

Costagliola, D. SS0404LB

Cote, G. OAC1003

Cottle, T. OAD1403

Coutinho, C. OAC1004, OAE3002

Cowan, F. OAE1205

Crank, H. OAA3504

Crauwels, H. OAB2604, OAB2606LB

Crayton, A. OAD0706LB

Cressey, T.R. SS0405LB

Crichton, S. OAB3803

Cronin, S. OAA0202

Crowell, T. OAB3404

Crowell, T.A. OAB2103

Crusells, M.J. OAB3606LB

Culver, D.A. OAA1305

Cumbe, V. OAE3006LB

Curran, A. OAB3606LB

Currier, J.S. OAB3406LB

Czarnogorski, M. OAE1203

### D

D'Amico, R. OAB2604

D.J Bromberg, OAE2006LB

Dai, B. OAB1704

Daka, T. OAE2303

Dang, H. OAD3703

Daniels, B. OAB2605

Danpornprasert, D. OAB1702

Dapyen, G.A. OAB1706LB

Dareth, S. OAD1403

Darisheva, M. OAC1504

Darling, K. OAD3206LB

Das, C. OAC3306LB

Dassaye, R. OAB2605

Davenport, M. OAA1308

Davidovich, U. OAE3902

Davies, M.‐A. OAB1703

Davis, J.L. OAF1102

de la Fuente, S. OAB3606LB

de Miguel, M. OAB3606LB

De Rosa, S.C. OAA1305

De Schacht, C. OAC1005, OAE3006LB

Dear, N. OAB2103, OAB3404

Deeks, S.G. OAA0202

Del Amo, J. OAD3206LB

Delgado, P. OAB0103

DeMoor, R. OAB2604

Deogan, C. OAD3206LB

Deshmukh, A. OAB0102

Desjardins, D. OAA0205

Deuba, K. OAF1103

Di Gregorio, S. OAB3606LB

Diabaté, S. OAC2205

Diallo, K. OAC3304

Diallo, M.B. OAC3304

Diallo, T.M. OAD1404

Diao, I. OAC3304

Diaz‐Brito, V. OAB3606LB

Difongo, L. OAD1605

Diggs, M. OAB3406LB

Diop, K. OAD1404

Diop, M. OAC3304

Dirajlal‐Fargo, S. OAA0603

Dirawo, J. OAE1205

Dispinseri, S. OAB2605

Do, K. OAE2005

Doan Hong, A. OAE3904

Doan, L. OAE2005

Doblado‐Maldonado, A. OAB0106LB

Doherty, M. OAC1006LB

Domingo, P. OAB3606LB

Dong, K. OAA0203

Dongala, A. OAE3005

Donnell, D. SS0405LB

Dore, L. OAC1006LB

Doria‐Rose, N. OAA1306LB

Dorsainvil, M. OAE3005

Dorward, J. OAB3403, SS0405LB

Doster, M. OAA1304

Douglas, P.S. OAB3406LB

Dourado, I. OAC0805

Dow, D. OAD0505

Doyle, N. OAB3605

Drain, P.K. SS0405LB

Duangmala, P. OAF3104

Dueñas, C. OAB3606LB

Duette, G. OAA0202

Dumchev, K. OAE2006LB, OAF1106LB

Dumont, E. OAB3805

Dunaway, K. OAD3204, OAF2704, OAF4103

Dunbar, D.S. OAC2203

Duong Thuy, A. OAE3904

Durand, C. OAA1308

Durow, L. OAD1603

Duvivier, C. SS0404LB

Dvoriak, S. OAE2006LB

Dzavakwa, N. OAB2102

### E

Ebiama, L. OAE2304

Edet, B. OAC2905

Edom, R. OAC2202

Edupuganti, S. OAA1305

Edwards, J.K. OAC4003

Edwards, O. OAF4103

Efronson, E. OAC2203

Eigege, W. OAE0402

Ekerin, O. OAD2402

El‐Bassel, N. OAC1504, OAD0704

Eley, B.S. OAB1703

Elhassan, M. OAC3303

Elsbernd, K. OAC2202

Elvstam, O. OAB3402

Emmanuel, G. OAC2905

Ene, L. OAB3803

Enema, A. OAE2502

Engamba, D. OAB3806LB

Engelmann, F.A. OAA2804

English, D. OAE3004

Engwau, F. OAF2706LB

Ensoli, B. OAA1303

Epoku, B. OAF2706LB

Eshleman, S.H. OAE0406LB

Esserman, D. OAE2006LB

Evelia, H. OAD2403

Eyo, A. OAE2004

Ezeonwumelu, I. OAA2802

Ezieke, E. OAE2004

### F

Fabrice Yannick, A. OAE2304

Faire, C. OAC2203

Fang, L. SS0403LB

Fanjul, F.J. OAB3606LB

Farnum, S.O. OAC2906LB

Fatch, S. OAB3405

Faur, E. OAF3103

Feng, C. OAA0606LB

Fernando Vieira, W. OAE3002

Fernando, R. OAE3006LB

Ferra, S. OAB3606LB

Ferrand, R. OAB2102

Ferrari, G. OAA1305

Fert, A. OAA3502

Feser, M. OAE0404

Fichtenbaum, C.J. OAB3406LB, OAE0406LB

Figueroa, M.I. OAB0103

Filali‐Mouhim, A. OAA3502

Filippovych, M. OAC2906LB, OAE2006LB

Filteau, S. OAB2102

Fink, V. OAB0103

Finnegan, S. OAD1603

Fisher, K. OAA0202

Fisher, M. OAA3504

Fitch, K.V. OAB3406LB

Fitzmaurice, A.G. OAC1802

Flomo, D.J. OAC2203

Flores Andrade, X.A. OAE0403

Flynn, M. OAD1403

Fogel, J.M. OAE0406LB

Fomenko, T. OAC2906LB

Ford, N. OAE0404

Ford, S.L. OAB2604

Forleh, A. OAC2203

Forman, E. OAD0706LB

Fox, D. OAB0106LB

Francavilla, V. OAA1303

Franchini, G. OAA1304

Frank, I. OAA1305

Freedberg, K.A. OAE0404

Frigati, L. OAB1703

Frimpong, C. OAC4003

Frischknecht, P. OAA0204

Frndak, S. OAB2103, OAB3404

Frouard, J. OAA2802

Frye, V. OAC1504

Funderburg, N. OAA0603

Fwoloshi, S. OAB3806LB, OAC2206LB

### G

Günthard, H.F. OAA0204

Gachoki, C. OAD2403

Gaebler, C. SS0402LB

Gaisa, M. OAB0102

Galindo, M.J. OAB3606LB

Galli, L. OAB2605, OAB3803

Galvão, N. OAC0805

Gama, L. OAB2605

Gambo, A. OAE2004

Gandhi, M. SS0405LB

Gangula, R. OAA3505

Gao, F. OAE0406LB

Gao, S. OAD3704

Gaogane, P.C.W. OAD1605

Garcia De Leon Moreno, C. OAD3204

Garcia, J. OAB0103

Garcia‐Fraile, L. OAB3606LB

Gardiennet, E. OAA0205

Garmroudi, D. OAF1102

Garner, A. OAD3206LB

Garrett, N. OAB3403, SS0405LB

Gartland, M. OAB2603

Gatanaga, H. OAC0803

Gatechompol, S. OAB1702, OAB3603

Gaudino, A. OAE2502

Gaur, A. OAB2606LB

Gaur, A.H. OAE0406LB

Gbajumo, I. OAD2402

George, A.F. OAA2802

Ghosn, J. SS0404LB

Gianella, S. OAA2802

Gibbs, J. OAD1402

Gichuhi, S. OAB0104

Gichuru, E. OAE1202

Gil, P. OAB3606LB

Gilbert, L. OAC1504, OAD0704

Gildas, N. OAE2304

Girouard, J. OAA3502

Gittings, L. OAD2403

Goetghebuer, T. OAB3803

Goga, A. OAB2605

Goldstein, R. OAE2004

Golemba, M. OAB0103

Gomez, W. OAD3204

Gomez‐Ayerbe, C. OAB3406LB

Gomez‐Feliciano, K. OAE0406LB

Gopee, K. OAA0604

Gotz, H.M. OAE3902

Grangeiro, A. OAC0805

Granger, K. OAE2005

Graves, E. OAE3006LB

Gray, K. OAC2203

Greco, D.B. OAC0805

Green, K. OAE2005, OAE3904

Gregson, C. OAB2102

Griffith, F.J. OAD1402

Grigochuk, E. OAC1504

Grinspoon, S.K. OAB3406LB

Grinsztejn, B. OAC1004, OAC2902, OAE0406LB

Grinsztejn, B.G. OAE3002

Groot Bruinderink, M.L. OAE3902

Grove, R.A. OAB0106LB

Groves, A.K. OAD0706LB

Gruell, H. SS0402LB

Grunenberg, N. OAA1305

Gubser, C. OAA1308

Guerri‐Fernandez, R. OAB3606LB

Gumpo, L. OAE2305

Gun, A. OAB0103

Gunasena, M. OAA0603

Gunst, J.D. OAA0206LB

Gutowska, A. OAA1304

Gyamerah, A.O. OAD3706LB

### H

Haabanji, B.P. OAB3806LB

Habgood, H. OAD1403

Hachaambwa, L. OAC2206LB

Haddad, M. OAE2006LB

Hagins, D. OAB3604

Hahn, J. OAB2105

Hahne, A. OAD3203

Hale, F. OAF4103

Halliday, F. OAB2603

Halvas, E.K. OAE0406LB

Hamm, J. OAD3203

Han, K. OAB2604

Han, W.M. OAB3603

Hanan, N. OAB2603

Haney, M.J. OAA2805

Hanke, K. SS0402LB

Hanscom, B. OAE0406LB

Hansen, N.B. OAD1402

Happel, A. OAA0606LB

Haque, M. OAC1804

Haque, M.R. OAA2804

Haraka, F. OAC4002

Hariharan, V. OAA3503

Harkey, K. OAE1202, OAE1204

Harrington, C. OAB2604, OAB2606LB

Harsono, D. OAF1102

Hasham, A. OAD2403

Hasson, J.M. OAA2804

Hatchard, S. OAE3906LB

Havlir, D. OAE1203

Havlir, D.V. OAB2105

Heath, D. OAF4105

Heath, N. OAF4105

Heckman, B. OAB2606LB

Hedgcock, M. OAB2602

Heekes, A. OAB1703

Henderson, M. OAB3605

Henegar, C. OAB3803

Heptinstall, J.R. OAA1305

Herce, M.E. OAC4003

Hermez, J. OAC3303

Hewa, M. OAE1204

Hightow‐Weidman, L. OAD1405

Hill, A. OAB3806LB

Hill, E. SS0405LB

Hillier, S. OAB3602

Hiransuthikul, A. OAB3603

Hisham, R. OAD0502

Ho, W. OAF1102

Hoagland, B. OAC2902

Hoang, T. OAA1304

Hoelscher, M. OAC2202

Hoffmann, T.U. OAB3803

Hofmann, J. SS0402LB

Hollingshead, B. OAD1403

Homeus, F. OAB3805

Hoornenborg, E. OAE3902

Hope, T.J. OAA2804

Hopkinson, B. OAD1403

Horton, J. OAB2603

Howe, S.E. OAA1304

Hoxha, A. OAC1006LB

Hsiao, J. OAA0602

Hsu, T.‐H. OAB3604

Htet, L. OAE2306LB

Huang, J. OAB2606LB

Huang, K. OAB3802

Huang, Y.‐S. OAB0105

Hudson, M. OAE0405

Hultquist, J. OAA2804

Hung, C.‐C. OAB0105

Hyle, E.P. OAE0404

### I

Iacono, M. OAF2704

Idemudia, A. OAE2004

Ighodaro, M. OAE2505

Ignatushyna, M. OAF1105

Imai‐Eaton, J.W. OAC2205

Imamdin, R. OAB2605

Imankulova, C. OAD0702

Imsanguan, W. OAB1702

Indravudh, P.P. OAE1205

Irungu, E. OAE1202

Islam Khan, S. OAC1804

Islam, S. OAE3003

Islam, Z. OAC2906LB

Israelski, D. OAB3805

Ivasiy, R. OAC2906LB, OAE2006LB

### J

Jörimann, L. OAA0204

Jackson, G.M. OAC2203

Jackson, R. OAB3602

Jacobs, D. OAB3403

Jagoe, L. OAC1003

Jalil, C. OAC1004

Jalil, E. OAC1004

James, E. OAE2004

Jamil, N. OAB2604

Jani, I. OAC2202

Jaspan, H. OAA0606LB

Jean, E. OAB3805

Jeenarain, N. OAB2605

Jeffery, P. OAB0106LB

Jeffrey, J.L. OAB2603

Jennings, A. OAE0406LB

Jennings, L. OAD3705

Jiang, X. OAA1304

Jimmy, E.P. OAC2203

Jirajariyavej, S. OAB1702

John, S. OAC1002

Johnson‐Peretz, J. OAE1203

Jones, B. OAB0106LB

Jones, J.C. OAD0705

Jongen, V.W. OAE3902

Joska, J. OAD3704

Josphine, R.N. OAD0504, OAF1104

Judd, A. OAB3803

Juelg, B. OAA0602

Juma, L. OAE1202

Jung, W. OAA0602

### K

Ka, D. OAC3304

Kaba, A.D. SS0404LB

Kabami, J. OAE1203

Kabunga, L. OAE0402

Kabwe, M. OAC1803

Kagunda, J. OAD2403

Kahl, L. OAB2603

Kahn, K. OAD3704

Kaireithi, T. OAE1202

Kakande, E. OAE1203

Kakanfo, K. OAE2004

Kakwera, P. OAD3702

Kalema, N. OAC1802

Kalitera, L. OAB3405

Kalk, E. OAB1703

Kallies, A. OAA1308

Kamakune, E. OAF2703

Kamanga, A. OAE3905

Kamolloh, K. OAE1204

Kampamba, D. OAB3806LB, OAC2206LB

Kampilimba‐Mwango, L. OAC2206LB

Kamya, M. OAD0503, OAE1203

Kamya, M.R. OAB2105

Kanene, C. OAC1803

Kangethe, J. OAB0104

Kanphukiew, A. OAC3305

Kanyane, A.S.E. OAD1605

Karim, F. OAA0604, OAA2803

Kasadha, B. OAF4103

Kasenge, B. OAC4003

Kasimonje, B. OAC4004

Kasone, V. OAE0402

Kasonka, L. OAB2102

Kasujja, R. OAD0503

Katbi, M. OAC2905

Katlama, C. SS0404LB

Kaufmann, D.E. OAA3502

Kavanagh, M. OAF0306LB, OAF4104

Kawanga, L. OAC2206LB, OAE3906LB

Kawashima, A. OAC0803

Kawichai, S. OAB2104

Kawonga Dunga, S. OAB3405

Kc, M.B. OAF1103

Keefer, M.C. OAA1305

Kehoe, K. OAB2106LB

Kelleher, A.D. OAA0202

Kemelbek kyzy, N. OAD0702

Kemigisa, B. OAC1505

Kerr, S. OAB3603

Kerr, S.J. OAB1702

Kershaw, T. OAD1402

Ketsuriyong, N. OAE2302

Key, S. OAC1003

Khaba, T. OAA2806LB

Khader, S. OAA2803

Khan, I. OAC1003

Khan‐Francis, R. OAC3302

Khanyile, M. SS0405LB

Kharandiuk, I. OAC2906LB

Khatun, F. OAC1502

Khosa, k. OAB2106LB

Khoshnood, K. OAF1102

Khwairakpam, G. OAE2003

Khytryk, T. OAD1902

Kiatchanon, W. OAC3305

Kibona, A. OAC4002

Kibuuka, H. OAB2103, OAB3404

Kigozi, J. OAC1802

Kikonyogo, R.N. OAC1505

Kimambo, S. OAC4002

Kimbugwe, M. OAD1903

Kindyomunda, R. OAF2706LB

King, C. OAF3102

Kiptinness, C. OAE1202

Kirklewski, S. OAD1402

Kirumira, P. OAB1704

Kirungi, R. OAE0402

Kitchel, A. OAC2203

Kityo, C. OAA0603

Kløverpris, H. OAA2803

Kløverpris, H.N. OAA0604

Klastrup, V. OAA0206LB

Klein, F. SS0402LB

Kobayashi, L. OAD3704

Koenig, S. OAB3805

Koenigs, C. OAB3803

Koethe, J.R. OAA3505

Kolobova, I. OAB2604

Kombe, M. OAC4002

Kondratuyk, S. OAF0302

Kong, X.‐P. OAA1304

Konstantanova, A. OAC2203

Kophaibunsiri, W. OAF3104

Koppe, U. OAD3203

Kor, S. SS0402LB

Korutaro, V. OAB2606LB

Kosalaraksa, P. OAB2104

Koss, C. OAE1203

Koup, R. OAA1306LB

Kouyos, R.D. OAA0204

Krakower, D.S. OAD0706LB

Kranzer, K. OAB2102

Krikorian, G. OAF0302

Kroch, A. OAB3602

Kroidl, A. OAC2202

Krotje, C. OAB2606LB

Kublin, J.G. OAA1305

Kucheruk, O. OAF2705

Kuchukhidze, S. OAC2205, OAF1106LB

Kuhn, L. OAA0602

Kuhn, W. OAA0604

Kulu, K. OAC1802

Kumar, A.P. OAB3806LB

Kumar, P. OAC3306LB

Kumar, S. OAE2503

Kumarasamy, N. OAB3804

Kunaka, N. OAD0703

Kunwar, R.S. OAF1103

Kusemerirwe, I. OAD3205

Kwach, B. OAC0802

Kwagala, B. OAD3702

Kwakwa, H. OAE3005

Kwardem, L. OAF4103

Kwena, Z. OAC0802

Kwobah, E. OAC4004

Kwon, Y.D. OAA1306LB

Kwong, P. OAA1306LB

Ky, S. OAD1606LB

Kyambadde, P. OAF2706LB

Kyana, J. OAD0504, OAF1104

Kyaw Kyaw, M. OAE2306LB

Kyeyune, F. OAB3802

Kyokushaba, J. OAE0402

### L

López Íñiguez, Á. OAE0403

Labode, R. OAF3105

Lalak, K. OAD3204

Lambert, A. OAE2505

Lamontagne, E. OAF0306LB

Lancaster, K. OAC4004

Landovitz, R. OAE0406LB

Langhave, A. OAA2803

Lankiewicz, E. OAE2003

Lara‐Medrano, R. OAC1506LB

Larasati, B. OAF0305

Lataillade, M. OAB2603

Lauckner, C.K. OAD1402

Laurenzi, C. OAD0502

Lavoy, G. OAE1203

Lawrence, J. OAC3302

Le Grand, R. OAA0205

Le, G. OAE2005

Le, M.G. OAD3703

Lechuga, J. OAC1002

Lee, G.Q. OAA0203

Lee, J. OAB3604

Lenders, A. OAB2106LB

Lequechane, J. OAC2202

Lertpiriyasuwat, C. OAC3305

Leshanok, A. OAD3204

Leslie, A. OAA0604, OAA2803

Levintow, S. OAD1405

Lewin, S.R. OAA1308

Lewis, L. OAB3403

Lewis, R. OAC1006LB

Li, F. OAA0602

Li, L. OAD3703

Li, S. OAA1305, OAA1308

Liautaud, B. OAB3805

Light, L. OAB3602

Lightfoot, M. OAD3706LB

Lin, C. OAD3703

Lin, K.‐Y. OAB0105

Lindsay, B. OAE2303

Lippman, S.A. OAD3706LB

Lishimpi, K. OAC2206LB

Litunya, J. OAE1203

Liu, W.‐C. OAB0105

Liu, W.‐D. OAB0105

Liu, Y. OAB0102

Liyanage, N. OAA0603

Logie, C. OAD2403

Lombaard, J. OAB2604

Lone Tip, S. OAE2306LB

Long, A. OAD1403

Looze, P. OAD3204

Lorenzo‐Redondo, R. OAA2804

Losa, J.E. OAB3606LB

Lowenthal, E. OAB2606LB

Lu, M.T. OAB3406LB

Lu, S. OAA1305

Lu, Y. OAB1705

Ludwig‐Barron, N. OAC1002

Lumbiganon, P. OAB2104

Luna, A. OAF2704

Lungo, S. OAC1805, OAD1904

Luo, C.C. OAA1304

Luo, X. OAA2802

Lusimbo, R. OAC1802

Lutz, S. OAA3504

Luz, P. OAC2902

Lwilla, A.F. OAC2202

Lwin, H.M.S. OAB3603

Lwin, H.S. OAB1702

Lynch, S. OAF4104

Lyons, C. OAD3204

### M

Méndez López, A. OAD3206LB

Ma, T. OAA2802

Mabasa, H. OAB2106LB

Macías González, F. OAD1904

Machavariani, E. OAC2906LB, OAE2006LB

Macias, J. OAB3606LB

MacInnis, R. OAE2505

MacWilliam, J. OAE2502

Madden, L. OAE2006LB

Madden, L.M. OAC2906LB

Maddox, S. OAD1403

Madela, F. OAA2803

Madruga, J.V. OAB3406LB

Madukaji, L.S. OAB1706LB

Madziwa, L. OAA2803

Maggiorella, M.T. OAA1303

Magno, L. OAC0805

Maharajh, K. OAB2605

Maheu‐Giroux, M. OAC2205, OAF1106LB

Mahlobo, B. OAA2806LB

Mahumane, A. OAC2202

Maibaze, G. OAC1005

Maida, A. OAB3405

Mainardis, M. SS0404LB

Makoni, T. OAE0405

Makwaya, A. OAD2404

Malama, D. OAE2303

Malen, R. OAE1204

Malen, R.C. OAE1202

Mallal, S. OAA3505

Malunga, S. OAE2502

Malvestutto, C.D. OAB3406LB

Mambo, F. OAE3006LB

Mancini, F. OAA1303

Mancini, G. SS0403LB

Manditsera, M. OAD2405

Mandyata, C. OAC4003

Manga, N.M. OAC3304

Manne‐Goehler, J. OAD3704

Manzanares, E. OAB3606LB

Manzini, V. OAA2803

Maphosa, T. OAB3405, OAE0404

Marc, J.B. OAB3805

Marczyńska, M. OAB3803

Maria Elena, R. OAC1002

Marima, R. OAC2903

Marrakchi, O. OAF0303

Martín‐Sánchez, M. OAD3203

Martínez‐Reséndez, M.F. OAC1506LB

Martelotto, L. OAA1308

Martin Onraet, A. OAE0403

Martin, A. OAD0705

Martinelli, E. OAA2804

Martinez, E. OAB3606LB, OAD3206LB

Marzinke, M. OAB2606LB

Marzinke, M.A. OAE0406LB

Mashele, N. OAD3705

Masheto, G. OAB2606LB

Masia, M. OAB3606LB

Maskew, M. OAE3905

Masombuka, X. OAB3403

Massaly, A. OAC3304

Maswai, J. OAB2103, OAB3404

Matenga, T. OAC4003

Matiya, E. OAE0404

Matlou, M. OAB2605

Matos, V. OAC2902

Matsikire, E. OAE1205

Matyushina, D. OAD3204

Matz, L. OAC1003

Maust, B. OAA0606LB

Mavudze, J. OAD0703

Mawoyo, T. OAD0502

Mayer, K.H. OAA1305

Mayorga Sagastume, R. OAC1006LB

Mbewe, M. OAB3806LB

Mbewe, N. OAB3806LB, OAE2303

Mbewe, P. OAC4003

Mbewe, R. OAF4103

Mbofana, F. OAC2205

Mboup, A. OAC3304

McArthur, C. OAC1003

McCallum, S. OAB3406LB

McCauley, M. OAE0406LB

McCoig, C. OAB2606LB

McCrimmon, T. OAC1504

McDonnell, W.J. OAA3505

McDowell, E.C. OAD0706LB

McEwen, H. OAF3103

McFarland, W. OAC3302, OAC4005

McKee, K. OAA1306LB

McNamara, R. OAA0602

McRaven, M.D. OAA2804

Medley, B. OAD0704

Meggi, B. OAC2202

Mehra, V.L. OAA1305

Mehrotra, A. OAE2505

Meixenberger, K. SS0402LB

Mejía‐Castrejón, J. OAC4004

Melanie, L.R. OAD1603

Melard, A. OAA0205

Mellors, J. OAE0406LB

Mellouk, O. OAF0302

Mendes, I. OAB2602

Mensah, M. OAD3706LB

Mensah, Y.C. OAF2702

Merimeche, M. SS0404LB

Merino, D. OAB3606LB

Meteliuk, A. OAC2906LB

Metzner, K.J. OAA0204

Meya, D.B. OAB1704

Mfusi, S. OAA0604

Miliotis, S. OAA2802

Milligan, R. OAB2606LB

Mills, S. OAE2504

Mims, K. OAD0706LB

Min Thaung, Y. OAE2306LB

Min, S. OAB2604

Minga, A. OAC4004

Minot, S. OAA0606LB

Mireille, M.N. OAE2304

Mirzazadeh, A. OAC3303

Mitha, A. OAE2502

Mizushima, D. OAC0803

Mkhize, N.N. OAB2605

Mkumbo, J. OAC4002

Mlaba, M. OAA3506LB

Moga, T. OAD0703

Mogaka, F. OAE1204

Mohamed, H. OAC3303

Moiane, J. OAE3903

Mojeed, R. OAD2402

Moles, J.P. OAB2605

Moles, R. OAA1304

Molina, J.‐M. SS0404LB

Monare, D.C. OAF4106LB

Monceaux, V. OAA0205

Monini, P. OAA1303

Montefiori, D.C. OAA1305

Monteiro, L. OAC1004

Montejano, R. OAB3606LB

Montezuma‐Rusca, J.M. OAB2602

Moodley, M. OAA2806LB

Moodley, P. SS0405LB

Moore, P. OAB2605

Moragas, M. OAB0103

Moran, H. OAD3204

Moreira Gabriel, E. OAA3502

Moreira, R. OAC1004

Moretti, S. OAA1303

Morgan, A.J. OAE3905

Morris, D.E. OAA1305

Moscatelli, A.A. OAC4005

Moser, A. OAE0406LB

Mosha, J. OAD0505

Mounzer, K. OAB2602

Moura da Silva, L. OAF4102

Moye, J. OAB2606LB

Mozalevskis, A. OAC1006LB

Mphande, M. OAD2404

Mpofu, A. OAE1205

Mthabela, N. OAA0604, OAA2803

Mtulutsa, M. OAD2404

Mubaiwa, V. OAD2405

Muchanga, G. OAE2303

Mudaly, V. OAB1703

Mudenyanga, C. OAC2202

Mudiope, P. OAF2706LB

Mueller, M. OAC2202

Muessig, K. OAD1405

Mugala, A. OAC4003

Mugisa, B. OAC3303

Mugisa, D.B. OAB2106LB

Mugoni, T. OAD2405

Mugurungi, O. OAE1205

Muhumuza, U. OAD1905

Mujansi, M. OAE2303

Mujuru, H. OAB2102

Mukherjee, S. OAF0306LB

Mukome, B. OAD2405

Mukondwa, R.W. OAE0405

Mukuka, J. OAE2303

Mukuzunga, M. OAD2405

Mulenga, L. OAB3806LB, OAC2206LB,OAE3906LB

Mulenga, M. OAC1803

Muller‐Trutwin, M. OAA0205

Mumba, S. OAD0504

Munatsi, P. SS0405LB

Mundhe, S. OAC4004

Munjoma, M. OAD0703

Murau, F. OAF4103

Mureithi, F. OAC4004

Mureithi, M. OAB0104

Murenjekwa, W. OAE1205

Murenzi, G. OAC4004

Murray, S. OAD3202

Musabyimana, F. OAC4004

Musangulule, J. OAE3906LB

Musanje, K. OAD0503

Mushakarara, O. OAB2106LB

Musheke, M. OAC1803

Musiime, V. OAA0603

Musokotwane, K. OAC2206LB

Musonda, M. OAC2206LB, OAE3906LB

Mussa, O. OAE2502

Mutai, K. OAB0104

Mutale, K. OAE2502

Mutale, W. OAC4003, OAE2303

Mutanda, N. OAE3905

Mutede, B. OAD0703

Mutongore, M. OAE2502

Mutukwa, J. OAC2206LB

Mwamba, D. OAE3906LB

Mwamba, E. OAB3806LB

Mwangi, D. SS0402LB

Mwango, L. OAE2303

Mwangwa, F. OAB2105

Mwansa, M. OAC2206LB

Mwape, F. OAC1803

Mweemba, A. OAB3806LB

Mwila, C. OAC4003

Mwitumwa, M. OAC2206LB, OAE2303

Mzyece, J. OAC2206LB

### N

N'guessan, K.F.N. OAA1304

Nabaweesi1, F. OAC1505

Nabitaka, V. OAE0402

Nabude, S.P. OAD3205

Nachalwe, N. OAC4003

Nagot, N. OAB2605

Nahumuza, Z. OAD1905

Naicker, N. SS0405LB

Naidoo, L. OAB2605

Naipauer, J. OAB0103

Naiwatanakul, T. OAC3305

Nakimuli, M. OAF2703

Nakyeyune, F. OAD3702

Naldiga, S. OAA3504

Nalinikanta, R. OAE2003

Nalintya, E.K. OAB1704

Naluyima, R.D. OAB1704

Namachapa, K. OAC2204

Namahoot, P. OAC3305

Namara, L.S. OAC1505

Namimbi, F. OAC1802

Namuli, T. OAB1704

Namuwenge, P.M. OAE0402

Nankia, I. OAB3802

Nanyonjo, A. OAC1505

Narasimhan, M. OAD2403

Nasuwa, F. OAD0505

Natcha, N. OAB1702

Nava, A. OAB0103

Naver, L. OAB3803

Ncube, G. OAE1205

Ncube, P.A. OAF4106LB

Ndhlovu, A. OAC2206LB, OAE3906LB

Ndhlovu, Z. OAA2806LB, OAA3506LB

Ndung'u, T. OAA0203

Neidleman, J.A. OAA2802

Neumann, K. OAA0204

Newham, B. OAD1603

Ngalla, P. OAC4002

Ngandu, N. OAB2605

Ngassaki‐Yoka, C.‐D. OAA3502

Ngauv, B. OAD1606LB

Ngcobo, N. OAB2605

Ngema, N. OAA3506LB

Ngo Minh, T. OAE3904

Ngom, N.F. OAC3304

Ngong, A. OAE2304

Ngosa, B. OAC1803

Ngure, K. OAE1202, OAE1204

Nguyen Anh, D. OAE3904

Nguyen Hoang, G. OAE3904

Nguyen, B.D. OAD3703

Nguyen, H. OAE2005

Nguyen, K. OAE2005

Nguyen, T.T. OAD3703

Nhando, N. OAD0703

Niangoran, S. OAF1106LB

Nicholson, E. OAD1402

Nieves, A. OAE2002

Njala, J. OAD2404

Njikho, L. OAD2404

Nkhonjera, J. OAC2204

Noel Baumgartner, J. OAD0505

Noguera‐Julian, A. OAB3803

Noknoy, S. OAC3305

Noopetch, P. OAB1702

Noori, T. OAD3206LB

Northbrook, S. OAC3305

Nsama, D. OAC2206LB

Nthenge, K. OAA3505

Ntinginya, N.E. OAC2202

Ntjikelane, V. OAE3905

Nuttall, J. OAB1703

Nuwagira, A. OAE0402

Nwadike, C. OAE2004

Nwangeneh, C. OAE2004

Nwanja, E. OAE2004

Nxele, S. OAA2806LB

Nyabuti, M. OAB2105, OAE1203

Nyafesa, T. OAD2405

Nyahuma, G. OAD2405

Nyakanda, E. OAD2405

Nyambe, C. OAD1605

Nyatsanza, T. OAD2405

Nyhan, K. OAF1102

Nyirenda, R. OAE0404

Nyquist, S. OAA2803

Nzaddi, A.D. OAE2304

Nziza, N. OAA0602

### O

O'Brien, K. OAB3602

O'Rourke, J. OAB3803

O'Dell, S. OAA1306LB

Obare, L.M. OAA3505

Obermeier, M. SS0402LB

Obiora‐Okafo, C. OAE2004

Ochanda, R. OAE2502

Ochwal, P. OAE1204

Odari, E. OAB0104

Odira, A. OAE1204

Odoyo, J.B. OAC0802

Ofuche, E. OAB1706LB

Ogello, V. OAE1204

Ogier, A. OAD1603

Ogundehin, D. OAE2004

Ogunsanya, A. OAD2402

Ohandza, C.S. OAC3304

Oka, S. OAC0803

Okolo, C. OAE2004

Okonkwo, P. OAB1706LB

Okonkwo, P.C. OAB1706LB

Okopi, J.A. OAA0605

Okoye, M. OAB1706LB

Olais, E. OAD2405

Olin, R. OAD1403

Oliveira Leite, B. OAC0805

Oliveros, D. OAE2006LB

Omo‐Emmanuel, K. OAE2004

Omollo, V. OAE1202, OAE1204

Omondi, B. OAD2403

Ong'wen, P. OAE1202

Onimode, B. OAE2004

Onodera, T. OAB2603

Onwah, O. OAE2004

Onwuatuelo, I. OAB1706LB

Onwuzuruigbo, U. OAE2004

Onyedinachi, O. OAE2004

Opiyo, M. OAE1204

Opondo, D. OAC2903

Orlando de Castro Rafael, C. OAC1005

Orrell, C. OAD3705

Ortblad, K.F. OAE1202, OAE1204

Osawe, S. OAA0605

Otieno, P. OAE1202

Otieno, T. OAF4103

Ouattara, M. SS0404LB

Oucul, L. OAF2703

Ouk, V. OAD1606LB

Ouma, J. OAF4103

Ounchanum, P. OAB2104, OAB2606LB

Overton, E.T. OAA1305, OAB0106LB

Oware, K. OAC0802

Owidi, E. OAE1204

Owiredu Hanson, S. OAF2702

Owolagba, F.E. OAB1706LB

Owuoth, J. OAB2103, OAB3404

Owusu, S.E. OAF2702

Oxenburgh, E. OAD1603

Oyawola, B. OAE2004

Oyekunle, T. OAE3006LB

Oyelaran, O. OAE2004

### P

Pérez Jiménez, C. OAE0403

Pöge, K. OAD3203

Paioni, P. OAB3803

Palakawong Na Ayuthaya, T. OAB1702

Palma Solórzano, C.A. OAC1805

Palma, C. OAD1904

Palmer, S. OAA0202, OAD0706LB

Panagiotoglou, D. OAC2205

Panarat, P. OAB1702

Panda, R. OAD3705

Pande, G. OAF2706LB

Pandey, G. OAE2503

Pandey, L.R. OAF1103

Paoboonprung, P. OAE2302

Pape, J.W. OAB3805

Paquin Proulx, D. OAA1304

Parazzo, H. OAC4004

Parcesepe, A. OAC4004

Parker, Z. OAB2103, OAB3404

Parry, C.M. OAB0106LB

Pasanen, S. OAD3206LB

Pascoe, R.D. OAA1308

Pascoe, S.S. OAA2804

Passaes, C. OAA0205

Pastellides, C. OAB3403

Patel, N. OAB2604

Patpeerapong, P. OAE2504

Paul, A. OAA2805

Paulo, P. OAE3006LB

Pavie, J. SS0404LB

Pavone Cossut, M.R. OAA1303

Pegu, A. OAA1306LB

Peixoto, E. OAC1004

Penack, O. SS0402LB

Peng, J. OAB2105

Pensiero, M.N. OAA1305

Peraire, J. OAB3606LB

Peralta Prado, A.B. OAE0403

Pereira, K. OAC2202

Perez, C.F. OAB0103

Perez, G. OAC1002

Perez‐Stachowski, J. OAB3606LB

Perotti, M. SS0402LB

Perry, D. OAC3302

Petersen, M. OAE1203

Petretti, S. OAF4103

Petrov‐Sanchez, V. SS0404LB

Pham, C. OAE2005

Phan Thi Thu, H. OAE3904

Phan, H. OAE2005

Phanuphak, N. OAE2506LB

Phillips, A. OAE0404, OAE1205

Phiri, A. OAC1803

Phiri, C. OAC2206LB

Phiri, D.J. OAE3906LB

Phiri, H. OAB3806LB

Phiri, K. OAD2404

Phiri, S. OAD2404

Phyoe Naing, K. OAE2306LB

Pialloux, G. SS0404LB

Picconi, O. OAA1303

Pierre, S. OAB3805

Pierson, T. OAA1306LB

Pimenta, M.C. OAC2902

Pingsusaen, P. OAB1702

Pinto Junior, J.A. OAC0805

Pintye, J. OAB0104

Pius, B. OAE2004

Pius, J. OAE2004

Piwowar‐Manning, E. OAE0406LB

Planas, D. OAA3502

Platt, L. OAF1106LB

Policek, N. OAF4103

Poonwanasatien, A. OAE2302

Porrachia, M. OAA2802

Porterfield, J.Z. OAA0604

Portilla, J. OAB3606LB

Poverga, R. SS0406LB

Power, M. OAD1403

Powers, K. OAD1405

Prieto, M. OAE3903

Primbetova, S. OAC1504

Prins, M. OAE3902

Probst, U. OAC0804

Prochazka, M. OAC1006LB

Pry, J.M. OAC4003

Puentes, J. OAC1002

Punsuwan, N. OAC3305

Purohit, S. OAE2503

Puryear, S. OAB2105

Puthanakit, T. OAB2104

PVM, L. OAC3306LB

Pykalo, I. OAE2006LB

### Q

Qian, Y. OAE0404

### R

Rabie, H. OAB1703

Rahaman, A. OAC1804

Rahman, M.A. OAA1304

Rajabo, L.S. OAE2305

Rajan, S. OAE2503

Rajasingham, R. OAB1704

Rajdev, L. OAA1308

Ramírez‐Elizondo, M.T. OAC1506LB

Ramgopal, M. OAB2602, OAB3604

Ramirez, P.L. OAC4005

Ramjeth, A. OAB2605

Ramonfaur‐Gracia, D. OAC1506LB

Ramos, J.T. OAB3803

Ramos, M. OAC1004

Ramraj, T. OAB2605

Ramsingh, R. OAC1003

Rana, R. OAC1503

Rangaraj, A. OAE0404

Rao, A. OAD3204

Rao, M. OAA1304

Rashid, M.‐U. OAE3003

Rasmussen, T.A. OAA0206LB, OAA1308

Rattakittvijun Na Nakorn, P. OAE2302, OAE2504

Rattue, M. OAF4103

Rauscher, M. OAC2202

Rawat, A. OAC1503

Raymond Marchand, L. OAA3502

Reddy, K. OAA0203

Reddy, M. OAB2605

Reddy, N. OAA0203

Reddy, T. OAB2605

Reeders, D. OAD1603

Reisner, S.L. OAE3002

Revollo, B. OAB3606LB

Riako Anam, F. OAF0304

Ribaudo, H.J. OAB3406LB

Ribeiro Ferreira, A. OAF4102

Rich, J. OAD1403

Richardson, B. OAA0603

Rinehart, A.R. OAE0406LB

Rivera, K. OAF1105

Rivera, V. OAB3805

Roach, M.A. OAD3204

Roache, C. OAC1003

Roan, N.R. OAA2802

Robb‐Allen, A. OAC3302

Roberts, G. OAB2606LB

Roberts‐Toler, C. OAD3704

Robinson, S.T. OAA1305

Roche, S. OAE1202

Roche, S.D. OAE1204

Rodríguez Zulueta, A.P. OAE0403

Rodrigo, H. OAA2805

Rogg, L. OAB2603

Rohr, J. OAD3704

Romo, M. OAB3404

Rono, B. OAE1202

Rooney, J.F. OAE0406LB

Rosen, S. OAE3905

Rosenberg, M. OAD3704

Ross, J. OAC4004

Ross, J.L. OAB2104

Rosso, M. OAD1405

Rota, G. OAE1202

Roth, A.M. OAD0706LB

Rousseau, E. OAD3705

Routy, J.‐P. OAA3502

Rouzioux, C. OAA0205

Rowland‐Jones, S. OAB2102

Roy, D. OAA2805

Roy, U. OAA2805

Rozario, M.G. OAE3003

Rubenstein, E. SS0404LB

Rucinski, K. OAD3204

Ruel, T. OAB2105

Ruffner, M. OAF0304

Rungmaitree, S. OAB2104

Russell, W.A. OAC2205

Rust, L. OAA3504

Rutto, G. OAC2903

Ryan, P. OAB3606LB

### S

Sürücü, G. SS0402LB

Sabi, I. OAC2202

Sacha, J. OAA3504

Saez‐Cirion, A. OAA0205

Saienko, A. OAD1902

Saini, S. OAD1403

Salas, M.E. OAB0103

Salazar, J. OAC1002

Salusso, D. OAB0103

Samer, S. OAA2804

Samitpol, K. OAE2506LB

Samona, A. OAC1803

Samreth, S. OAD1606LB

Samson, L. OAD0505

Samsunder, N. SS0405LB

Samuels, J. OAB1706LB

Sanchez, T. OAD3202

Sande, L. OAE3905

Sander, L.E. SS0402LB

Sanga, E. OAD0505

Sangong Rose, E. OAE2304

Santana, J. OAB2602

Santayakul, S. OAE2504

Saphonn, V. OAD1606LB

Sardinha, L. OAC2205

Sarkis, S. OAA1304

Sarma, N. OAC0804

Sarr, C. OAC3304

Sarwar, G. OAC1804

Sawatwipachai, B. OAE2506LB

Sax, P.E. OAB3805

Scarlatti, G. OAB2605

Schaible, U. OAB2102

Scheim, A. OAD3202

Scherzer, M. OAC1006LB

Schietroma, I. OAA1303

Schifanella, L. OAA1304

Schiff, M. OAE2505

Schim van der Loeff, M. OAE3902

Schneider, C.G. SS0402LB

Schneider, T. SS0402LB

Schommers, P. SS0402LB

Schroeder, J. OAA1308

Scinto, H. OAA1304

Scott, K. OAB3803

Scott, N. OAE3905

Scott‐Walker, L. OAE3004

Seale, A. OAC1006LB

Seaman, M.S. OAA1305

Seang, K. OAD1606LB

Seaton, K.E. OAA1305

Segal‐Maurer, S. OAB2602

Seixas, S. OAC0805

Sekabira, K.S. OAD3205

Seleme, J. OAC1005

Semini, I. OAF0304

September, Q. OAB2605

Sethi, A.K. OAC3306LB

Severe, P. OAB3805

Seydi, M. OAC3304

Sgadari, C. OAA1303

Shaaban, M.A. OAA2804

Shah, N. OAB2103, OAB3404

Shahi, S. OAF4103

Shaik Abdool, F. OAA0604

Shalek, A. OAA2803

Shankalala, P. OAC4003

Sharma, A. OAF4104

Shebo, S. OAC1803

Shen, X. OAA1304

Sherr, L. OAD0502

Shete, P. OAE0405

Shey, L.H. OAE2304

Shibemba, A. OAB3806LB

Shin​, J.‐G. OAB1702

Shingoose, C. OAC1003

Shiojiri, D. OAC0803

Shomuyiwa, D.O. OAD2402

Short, W.R. OAB3604

Shroufi, A. OAE0404

Shubert, V. OAF3102

Shumba, G. OAD2405

Shumskaia, N. OAD0702

Siame, C. OAC1803

Siame, M. OAC2206LB, OAC4003

Sibanda, E. OAE1205

Sibiya, A.L. OAA0604

Sibiya, K. OAD1605

Sichone, P. OAC4003

Sienra Iracheta, E. OAE0403

Sierra Madero, J.G. OAE0403

Sigel, K. OAB0102

Silere‐Maqetseba, T. OAB2106LB

Siliciano, J. OAA3503

Siliciano, R. OAA3503

Silikpoh, M. OAC2203

Siluka, I. OAE3906LB

Silva de Castro, I. OAA1304

Silva Rodrigues, M.K. OAF4102

Simms, V. OAB2102

Simoes, D. OAD3206LB

Simonetti, F. OAA3503

Simoni, J. OAC0802

Simons, B. OAB3806LB

Simons, L. OAA2804

Simonson, R.B. OAB3604

Sinclair, K. OAD1603

Sing'oei, V. OAB2103, OAB3404

Singh, U. SS0405LB

Singh, Y. OAB2605

Sinyangwe, G. OAC2206LB

Siriphan, J. OAF3104

Sirisegaram, L. OAB3602

Sittikarn, S. OAE2302

Sivile, S. OAB3806LB, OAC2206LB

Siwingwa, M. OAB3806LB, OAC2206LB

Skinner, S. OAC1003

Skipalska, H. OAD1902

Skipper, C.P. OAB1704

Sklar, P. OAB2602

Skyers, N. OAC3302

Slama, L. SS0404LB

Slim, J. OAB0106LB, OAB2602

Smedley, J. OAA3504

Smith, A. OAD1403

Smith, D. OAD2404

Smith, D.M. OAA2802

Smith, E.D. OAB3406LB

Smith, J. OAE3004

Smith, M. OAB1703

Smith, S.A. OAA3505

Soares, F. OAC0805

Soberanis, S. OAD1904

Soberano, Z. OAD1405

Soegaard, O.S. OAA0206LB

Solis Reyes, P. OAB3802

Solomon, A. OAA1308

Sonaike, O. OAD2402

Songo, J. OAD2404

Songtaweesin, W.N. OAB2104, OAB3803

Sookrajh, Y. SS0405LB

Sophonphan, J. OAB1702

Soto‐Torres, L. OAE0406LB

Soule, E. OAD1403

Sow, N.K. OAD1404

Spedding, J. OAD1403

Spoulou, V. OAB3803

Sprague, L. OAD3204

Spreen, W. OAB2604

Srivatsan, V. OAF0306LB, OAF4104

St Clair, M. OAB2604

St Silva, M. OAC1004

Stöckl, H. OAC2205

Stackpool‐Moore, L. OAD1603

Stamp, B. OAD1405

Sternthal, I. OAD2403

Stevenson, L. OAD1602

Steventon Roberts, K. OAD0502

Stewart, J. OAC0802, OAE1204

Stockton, M. OAC4004

Stoebenau, K. OAE2303

Stoicescu, C. OAD0704

Stokes, T. OAE3005

Stone, J. OAF1106LB

Strachan, S. OAF4103

Su, L.‐H. OAB0105

Sudjaritruk, T. OAB2104

Sui, Y. OAA1304

Sullivan, A. OAD3206LB

Sun, H.‐Y. OAB0105

Sun, S. OAA0603

Sundaresan, S. OAB3805

Sunday, H. OAE1203

Suprasert, B. OAC4005

Surgers, L. SS0404LB

Sutter, N. OAE1203

Suwanlerk, T. OAB2104

Syarif, O. OAD3204

### T

Taing, L. OAD2403

Takaidza, T. OAD2405

Takarinda, K. OAE0405

Takenaka, B.P. OAD1402

Talbot, V.R. OAE0404

Talton, J.D. OAA1304

Tambatamba, B. OAC2206LB

Tamilselvan, B. OAA0603

Tangkijvanich, P. OAB3603

Tanjung, P. OAD0704

Tantawarak, N. OAB3803

Taramusi, I. OAE1205

Taruberekera, N. OAD0703

Tate, M. OAC4005

Tauzie, B. OAD2404

Tavanxhi, N. OAF0304

Taveira, N. OAC2202

Tavengerwei, J. OAD2405

Tawiah, M.B. OAD3706LB

Tchofa, J. OAE2304

Temple, J. OAE1203

Tendolkar, I. OAE2502

Teng, I‐T. OAA1306LB

Tengatenga, C. OAD1402

Terlikbayeva, A. OAC1504

Terris‐Prestholt, F. OAF1106LB

Thamm, W. OAF4103

Thammapiwan, S. OAB1702

Thiam, B. OAD1404

Thiam, S. OAD1404

Tholanah, M. OAF4103

Thomas, C. OAE2003

Thomas, K.K. SS0405LB

Thomas, Y. OAA2804

Thunprom, S. OAF3104

Thuo, N. OAE1204

Thuruthiyil, C.T. OAA2804

Tiam, A. OAE0404

Tingiba, F. OAE2502

Tique, J. OAC1005

Tiraboschi, J.M. OAB3606LB

Tobii, M.K. OAC2203

Tobin, N. OAA0602

Tolstrup, M. OAA0206LB

Tomaras, G.D. OAA1304, OAA1305

Tomlinson, M. OAD0502

Torralba, M. OAB3606LB

Torres, T. OAC2902

Tourtellott‐Fogt, E. OAA1306LB

Townley, E. OAB2606LB

Toyo, O. OAE2004

Tram Tri, T. OAE3904

Tran Khanh, L. OAE3904

Tran, A. OAE2005

Tran, H.V. OAE0406LB

Tran, J. OAA3504

Tran, L. OAE2005

Tran, M. OAE2005

Treebupachatsakul, P. OAB1702

Trevisi, L. OAB3805

Tripiciano, A. OAA1303

Tschumi, J. OAA0204

Tsongo, Z.K. OAC3304

Tsoy, E. OAB2103

Tumpach, C. OAA1308

Turkova, A. OAB3803

Turpin, G. OAD3204

Turya, F. OAB1704

Tylleskar, T. OAB2605

### U

Ubolyam, S. OAB3603

Uemura, H. OAC0803

Umoh, P. OAC2905

Underwood, M. OAB0106LB, OAB2603

Ung, P. OAD1606LB

Unimuke, M. OAE2004

Utzschneider, D. OAA1308

### V

Valcour, V. OAB2103

Valencia Moreno, M. OAD1603

Valencia, J. OAB3406LB

van Beekum, I. OAE2305

van Bokhoven‐Rombouts, C. OAE3902

Van Borek, S. OAD2403

Van de Perre, P. OAB2605

Van de Ven, R. OAC4002

van der Molen, J. OAB3403

Van Eygen, V. OAB2606LB

van Harreveld, F. OAE3902

Van Oosterhout, J.J. OAD2404

Van Solingen‐Ristea, R. OAB2604, OAB2606LB

Vanderpuye, N.A. OAD3706LB

Vannappagari, V. OAB3803

Varsani, A. OAA0606LB

Vaughan, E. OAD3206LB

Vazquez, M. OAF4103

Vazquez, M.J. OAB3606LB

Veloso, V. OAC1004, OAC2902

Veloso, V.G. OAE3002

Vera, J. OAB3605

Verani, A. OAF0304

Veras, M.A.d.S.M. OAC4005

Verluyten, J. OAD3206LB

Vermandere, H. OAD1904

Vermeij, K. OAE3902

Vickerman, P. OAF1106LB

Vilanculos, J. OAE2305

Villinger, F. OAA2804

Villoslada, A. OAB3606LB

Vinikoor, M. OAC4003, OAE2303

Vinti, P. OAC1006LB

Violari, A. OAB2606LB

Violette, L. OAC0802

Visuthranukul, J. OAB1702

Voitchouck, C. SS0404LB

Volokha, A. OAB3803

Vu Ngoc, B. OAE3904

Vu Ngoc, Y. OAE3904

Vu, B. OAE2005

Vundavalli, S. OAB3605

Vuong, L. SS0402LB

### W

Wabomba, D. OAC1802

Wagner, R. OAD3704

Walters, M. OAC2205

Walukaga, S. OAB1704

Wambua, K.M. OAD0504

Wambua, S. OAE2502

Wang, D. OAA1306LB

Wang, M. SS0405LB

Wang, S. OAA1305

Wanjalla, C. OAA3505

Wanjiku Njenga, L. OAF4103

Wanyenze, R.K. OAC2205

Ward, K.M. OAD0706LB

Ward, S. OAB2606LB

Wareechai, P. OAC3305

Wargas, T. OAC1004

Watanabe, M.G. OAB3406LB

Watkins, E. OAD1403

Wattie, A. OAD1403

Webb, K. OAE0405

Wekpe, S. OAB1706LB

Welbourn, A. OAF4103

Wele, A. OAB1704

Wembonyama, S.O. OAC3304

Wembulua, B.S. OAC3304

Wendt, D. OAE2505

Were, D. OAE1202

West, B. OAC1504

West, N. OAE0405

Wester, C.W. OAB3806LB, OAC1005

Wiche Salinas, T.R. OAA3502

Wiebe, A. OAC1003

Wiginton, J.M. OAD3202

Wijstma, E.S. OAE3902

Willems, E. OAC0804

Williams, L.D. OAA1304

Wilson, E. OAC1004

Wilson, E.C. OAC4005

Winston, A. OAB3605

Wishnevetski, C. OAC1003

Wiwatrojanagul, S. OAB1702

Woeber, K. OAB2605

Wong, T. OAC1003

Woode, E. OAA1304

Woodworth, B. OAA2802

Woudstra, J. OAE3902

Wu, E. OAD0704

Wu, T.‐Y. OAB0105

### X

Xu, P. OAB3604

### Y

Yamin Pyone, T. OAE2306LB

Yang, E.S. OAA1306LB

Yemane Berhan, A. OAB3405

Yin, D. OAB2606LB

Yin, K. OAA2802

Young, K.C. OAA2802

Young, M. OAB3602

Youngkong, S. OAE2506LB

Yuma, S. OAC4002

Yusova, S. OAA3504

### Z

Zabih, S. OAB2606LB

Zandamela, A. OAE2305

Zanni, M.V. OAB3406LB

Zarei, M.M. OAA2805

Zaza, W.K. OAC2203

Zeeb, M. OAA0204

Zeriouh, R. OAD0705

Zhang, X. OAA3505, OAB2602

Zhang, Y. OAA3502

Zheng, H. OAA1305

Zholnerova, N. OAC1504

Zhou, T. OAA1306LB

Zhukov, I. OAF3103

Zimba, M. OAC1803

Zolla Pazner, S. OAA1304

Zulu, M.J. OAD1605

Zwerski, S. OAE0406LB

Zyambo, K. OAC2206LB

